# Ancestry-based differences in the immune phenotype are associated with lupus activity

**DOI:** 10.1172/jci.insight.169584

**Published:** 2023-08-22

**Authors:** Samantha Slight-Webb, Kevin Thomas, Miles Smith, Catriona A. Wagner, Susan Macwana, Aleksandra Bylinska, Michele Donato, Mai Dvorak, Sarah E. Chang, Alex Kuo, Peggie Cheung, Laurynas Kalesinskas, Ananthakrishnan Ganesan, Denis Dermadi, Carla J. Guthridge, Wade DeJager, Christian Wright, Mariko H. Foecke, Joan T. Merrill, Eliza Chakravarty, Cristina Arriens, Holden T. Maecker, Purvesh Khatri, Paul J. Utz, Judith A. James, Joel M. Guthridge

**Affiliations:** 1 Department of Arthritis and Clinical Immunology, Oklahoma Medical Research Foundation (OMRF), Oklahoma City, Oklahoma, USA.; 2 Departments of Medicine and Pathology, University of Oklahoma Health Sciences Center, Oklahoma City, Oklahoma, USA.; 3 Institute for Immunity, Transplantation and Infection;; 4 Center for Biomedical Informatics Research, Department of Medicine; and; 5 Division of Immunology and Rheumatology, Department of Medicine, Stanford University School of Medicine, Stanford, California, USA.

**Keywords:** Autoimmunity, Immunology, Cytokines, Lupus, Signal transduction

## Abstract

Systemic lupus erythematosus (SLE) affects 1 in 537 Black women, which is >2-fold more than White women. Black patients develop the disease at a younger age, have more severe symptoms, and have a greater chance of early mortality. We used a multiomics approach to uncover ancestry-associated immune alterations in patients with SLE and healthy controls that may contribute biologically to disease disparities. Cell composition, signaling, epigenetics, and proteomics were evaluated by mass cytometry; droplet-based single-cell transcriptomics and proteomics; and bead-based multiplex soluble mediator levels in plasma. We observed altered whole blood frequencies and enhanced activity in CD8^+^ T cells, B cells, monocytes, and DCs in Black patients with more active disease. Epigenetic modifications in CD8^+^ T cells (H3K27ac) could distinguish disease activity level in Black patients and differentiate Black from White patient samples. TLR3/4/7/8/9-related gene expression was elevated in immune cells from Black patients with SLE, and TLR7/8/9 and IFN-α phospho-signaling and cytokine responses were heightened even in immune cells from healthy Black control patients compared with White individuals. TLR stimulation of healthy immune cells recapitulated the ancestry-associated SLE immunophenotypes. This multiomic resource defines ancestry-associated immune phenotypes that differ between Black and White patients with SLE, which may influence the course and severity of SLE and other diseases.

## Graphical abstract

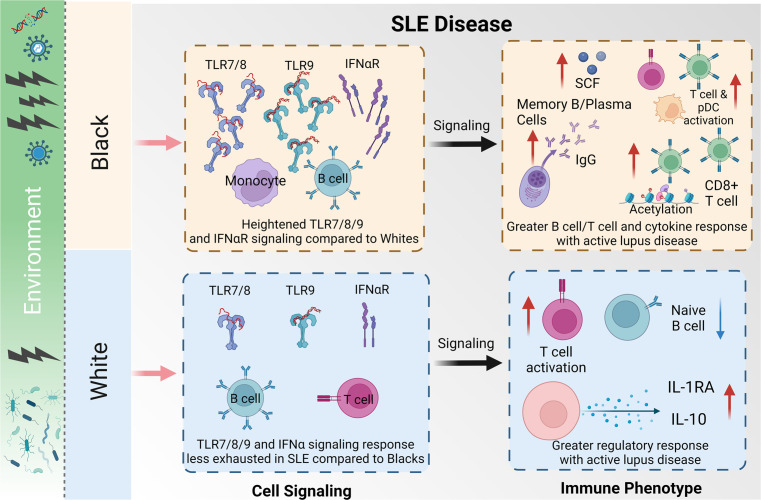

## Introduction

Systemic lupus erythematosus (SLE) is a heterogeneous disease with varied environmental and genetic risk factors that may contribute to onset, organ involvement, and disease course (1, 2). SLE prevalence is 2–3 times higher in Black than White individuals (3). African ancestry is associated with greater disease activity and severity and worse outcomes in SLE, with a higher prevalence of end-stage renal failure and earlier mortality (2). In the United States, SLE remains a top 10 medical cause of death in Black women between 15 and 45 years of age (4), and individuals of African ancestry have a more abrupt and earlier age of SLE onset compared with those of European descent (5). Although racial differences in disease presentation and severity are multifactorial, we focused on immunologic differences that may contribute to autoimmunity-related disease.

TLRs 7 and 9 are pattern recognition receptors that sense single-stranded RNA and DNA, respectively, and can drive type I IFN production and the generation of antiviral immune responses. Activation of TLR7 and TLR9 by recognition of self-nucleic acids is implicated in the pathogenesis and development of autoimmune disease (6). In SLE mouse models, TLR7 is a critical driver of disease, including nephritis and anti-RNA antibody production (7), and TLR9 is important for anti-DNA antibody development (7–9). Human SLE is associated with increased expression and activation of TLR7 and TLR9 on peripheral blood mononuclear cells (PBMCs), and activation of these TLRs induces IFN-α production (10–13). Certain pathways driving SLE pathogenesis are enhanced in Black versus White populations, with greater frequencies of patients with elevated IFN-α, B cell activation markers (CD86), and double negative 2 (DN2) autoantibody-producing B cells (14–16). Patient ancestry impacts whole blood gene expression signatures, which help define the molecular heterogeneity of SLE (17), with B cell–related expression signatures, including plasma cell signatures, more heavily enriched in Black versus White patients (17).

Differences in immune cell frequencies in individuals of different races have also been described, such as benign neutropenia, a greater CD8/CD4 ratio, and higher lymphocyte numbers, primarily of B cells, in Black individuals (18, 19). B cell numbers have also been shown to differ by sex, with the highest cells/mL values reported in Black women (20). Additionally, macrophages isolated from Black individuals display a stronger inflammatory response to pathogens such as *Listeria monocytogenes*, *Salmonella typhimurium*, and influenza A virus in vitro compared with macrophages from White individuals (21, 22).

Given the established role of ancestry-related differences in the immune response of healthy individuals, the current study investigated the influence of ancestry on immune response pathways linked to SLE, including TLR7, TLR9, and IFN-α, using a multiomics systems-level approach. Here, we catalog differences in the immune response in Black and White patients with SLE and healthy controls at the level of cell populations, transcriptomes, epigenomes, and signaling pathways. Notably, Black patients have substantial differences in dominant TLR signaling pathways, evidence of a B cell–skewed immune response, and epigenetic changes in CD8^+^ T cells, all of which may influence SLE disease phenotype and severity in this population.

## Results

### SLE immune phenotypes vary by ancestry and disease activity.

**Figure 1:**
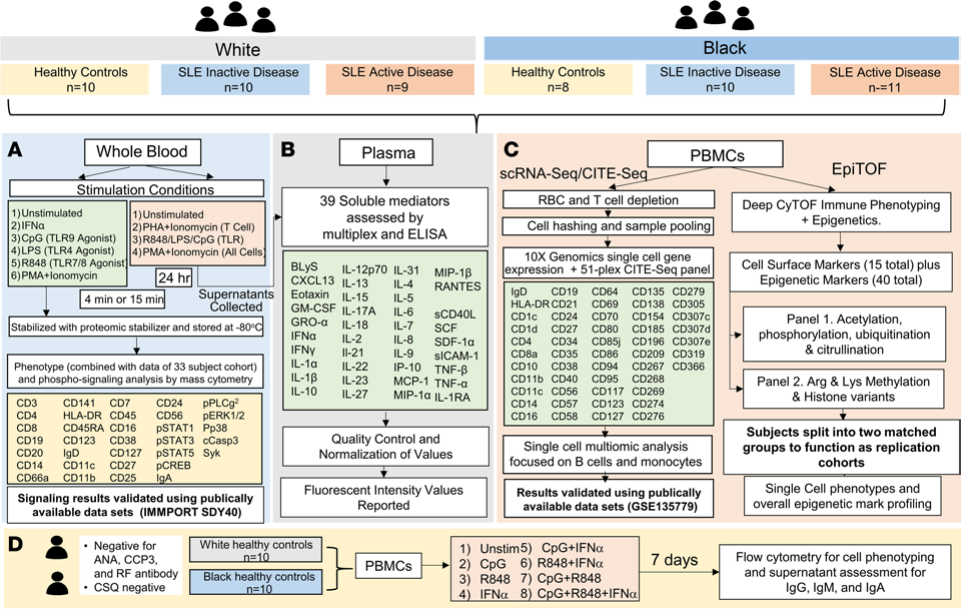
Overview of analysis workflow to catalog ancestry-associated differences in immune phenotypes. A discovery cohort was used for a multiomic systems immunology analysis. Significant results were independently validated either by reusing publicly available data sets or by splitting individuals into 2 matched groups for replication (bold). First, 58 samples, including healthy controls, SLE INACT, and SLE ACT, who self-reported as White or Black, were matched by age, ancestry, and sex. (**A**) Whole blood was collected, left unstimulated or stimulated, and used for immunophenotyping by mass cytometry and signaling analysis by phospho-CyTOF. Similar data from an independently collected and analyzed set of samples, a larger combined analysis of 33 participants, were used to increase the power. (**B**) Plasma and supernatants collected after overnight stimulation of whole blood were used to assess 39 different soluble mediators using multiplex bead-based assays and ELISAs. (**C**) PBMCs were used for droplet-based scRNA-Seq and EpiTOF. For single-cell transcriptomics, PBMCs were washed, depleted of red blood cells and T cells using CD2 depletion to enrich for non–T cell populations, and stained with a 51-plex CITE-Seq panel for dual transcript and protein expression using the 10x Genomics 3′ single-cell droplet methods. These variables were utilized to delineate specific cell lineages, activation, and regulatory markers. (**D**) PBMCs from healthy controls with no autoimmune disease manifestations who self-reported as White or Black were stimulated for 7 days with IFN-α, TLR7/8, or TLR9 agonists, alone or in combination, to assess immune composition and antibody production by flow cytometry and ELISA, respectively. ANA, antinuclear antibody; CCP, cyclic citrullinated peptide; CSQ, connective tissue screening questionnaire; EpiTOF, CyTOF immune phenotyping with epigenetics; scRNA-Seq, sincle-cell RNA sequencing; MCP-1, monocyte chemoattractant protein-1; PHA, phytohemagglutinin; p-STAT, phosphorylated STAT; RF, rheumatoid factor; SCF, stem cell factor.

To examine relationships between immune phenotypes, ancestry, and disease activity, SLE patients with inactive disease (SLE INACT: SLE disease activity index [SLEDAI] < 4), SLE patients with active disease (SLE ACT: SLEDAI ≥ 4), and controls were evaluated using multiomic profiling of blood samples (Figure 1 and Supplemental Figure 1; supplemental material available online with this article; https://doi.org/10.1172/jci.insight.169584DS1). The cohort included 29 individuals each of White or Black ancestry, and patients were matched by disease activity (SLEDAI), sex, and medication use (Supplemental Table 1). Black patients with SLE ACT tended to be slightly younger compared with other disease groups, and no significant differences were found between clinical criteria (Supplemental Table 2). Autoantibody specificities differed slightly, but not significantly, between Black and White patients, with higher frequencies of ribonucleoprotein- (RNP-) (26.3% White vs. 52.4% Black) or Sm/RNP-specific (31.6% White vs. 61.9% Black) autoantibodies in Black patients and higher frequencies of Ro- (52.6% White vs. 23.8% Black) and La-specific (15.8% White vs. 4.8% Black) autoantibodies in White patients (Supplemental Table 3).

**Figure 2:**
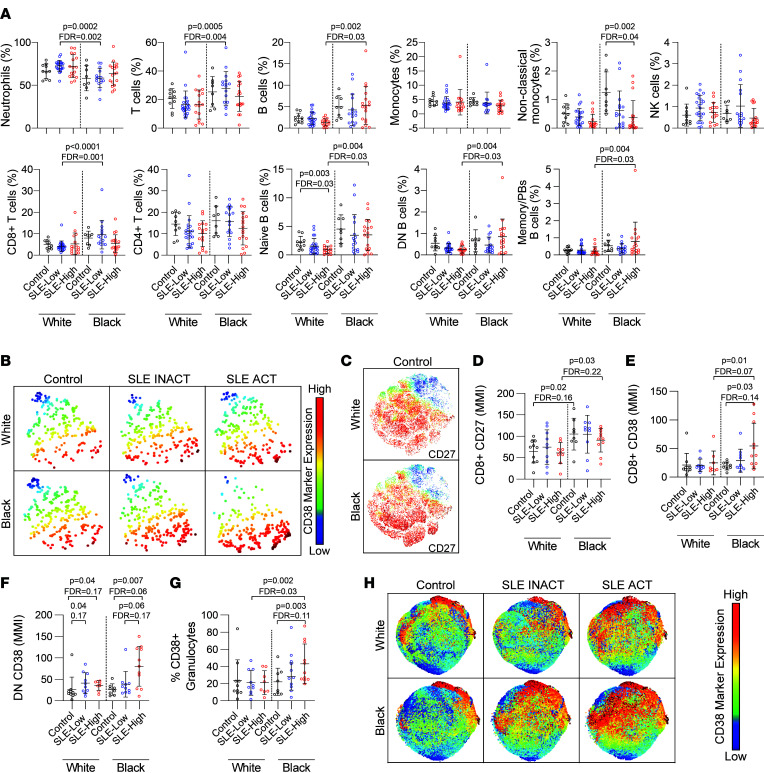
Cell type composition of whole blood from White and Black healthy controls and SLE patients with active and inactive disease. (**A**) The frequencies of major cell lineages in healthy controls, SLE inactive (INACT) patients, and SLE active (ACT) patients, as determined by mass cytometry (*n* = 91). (**B** and **C**) tSNE marker expression plots of CD38 expression on pDCs (**B**) and CD27 expression on T cells (**C**). The mean metal intensity (MMI) of (**D**) CD27 on CD8^+^ T cells and CD38 on (**E**) CD8^+^ T cells and (**F**) DN T cells was assessed from mass cytometry data via Cytobank. The frequency of (**G**) CD38^+^ granulocytes determined by biaxial gating and (**H**) expression of CD38 are shown on granulocytes by disease. Marker values are displayed on a color scale ranging from blue (lowest levels) through yellow (medium levels) to red (highest marker expression). All tSNE plots were derived from cumulative data from 8–11 individuals per group. For all plots, statistical significance was determined using the Kruskal-Wallis test with Benjamini-Hochberg correction for multiple comparison correction, and mean ± SD is shown. pDCs, plasmacytoid DCs.

Mass cytometry was performed to investigate whole blood phenotypes and phospho-signaling (Figure 1A). To assess changes in TLR cell signaling pathways, the mass cytometry panel was designed with 33 metal isotype-tagged monoclonal antibodies specific for cell lineage, phospho-proteins, and activation and homing markers that discern major immune cell subsets and subpopulations (Supplemental Table 4). Single-cell populations were visualized using a high-dimensionality reduction method, t-distributed stochastic neighbor embedding (tSNE), that downsampled the total cell population and unbiasedly clustered cells by similarity. Frequencies were determined by a standard biaxial gating scheme that incorporated all intact, live cells collected (Figure 2A and Supplemental Figure 2). The tSNE analysis included over 1.3 million cells (~17,000 cells/sample) and distinguished 22 phenotypically distinct clusters (Supplemental Figure 3A). Marker expression of gated phenotype clusters is summarized for major lineage markers via tSNE plot (Supplemental Figure 3B) and by median intensity in a heatmap (Supplemental Figure 3C and Supplemental Table 5) (23). Cell numbers from PBMCs and complete blood counts were used to back-calculate cell subset frequencies with respect to whole blood. A second data set consisting of an independent cohort of 32 controls (18 White and 14 Black) and 33 patients with SLE (13 White INACT, 7 White ACT, 6 Black INACT, and 7 Black ACT) matched by age, ancestry, and disease activity was also used (Supplemental Tables 6 and 7).

All individuals in each disease group were used to create tSNE plots, demonstrating that the immune cell composition of whole blood was different in all 6 comparison groups, depending on both ancestry and disease activity scores (Supplemental Figure 3D). For instance, total B cell, naive B cell, DN B cell, and memory/plasmablast frequencies were greater in Black versus White patients with SLE ACT (Figure 2A and Supplemental Tables 8 and 9). T cells, primarily CD8^+^ T cells, were also higher in Black versus White SLE INACT patients. In contrast, neutrophil counts trended lower in Black versus White samples regardless of disease status, as has been previously reported (Figure 2A) (18–20).

When we focused upon disease activity, an increase in the disease activity in White patients was associated with reduced frequencies of naive B cells, resting naive B cells, DN (CD27^–^IgD^–^) B cells, and basophils/eosinophils (Figure 2A, Supplemental Tables 8 and 9, and Supplemental Figure 4). In contrast, in Black samples, increased disease activity was associated with reduced frequencies of nonclassical monocytes and CD8^+^ T cells, as well as increased frequencies of naive B cells (resting and active), DN B cells (DN1/3, DN2), and memory B cells/plasmablasts (Figure 2A, Supplemental Tables 8 and 9, and Supplemental Figure 4). These data begin to illustrate that the whole blood immune composition from Black and White populations with active and inactive SLE is noticeably distinct.

### Enhanced cell activation in Black patients with SLE.

In addition to the changes in immune cell populations, the levels of activation of immune cell populations were assessed. Briefly, we assessed the frequencies and mean metal intensity (MMI) of CD38, HLA-DR, CD11c, CD11b, and CD27 in whole blood cell subsets (Supplemental Tables 10 and 11). We found that higher levels of CD38^+^ (Figure 2B) and CD38^+^HLA-DR^+^ double-positive activated T cells, monocytes, pDCs, and NK cells correlated with increased disease activity in all patients with SLE (Supplemental Figure 5, A–H). The T cell costimulatory molecule CD27 facilitates antigen-specific expansion and supports the survival of activated T cells (24). CD27 was more highly expressed in CD8^+^ T cells (Figure 2, C and D) and CD4^+^ T cells (Supplemental Figure 5I) across Black versus White samples irrespective of disease status. In addition, CD38 was more highly expressed in CD8^+^ T cells, DN T cells, and pDCs in Black versus White patients with SLE ACT (Figure 2, E–H, and Supplemental Figure 5J).

Based on the markers in this panel, we did not observe changes in the frequencies of activated B cells between SLE ACT and SLE INACT samples (Supplemental Tables 10 and 11). However, Black versus White naive B cells expressed higher levels of IgD, even in the absence of disease (Supplemental Figure 5, K and L). The immunoglobulin IgD is elevated in rheumatic disease, has a high proclivity toward binding to nuclear antigens, and induces potent inflammatory responses in myeloid cells (25, 26). In addition, the frequency of activated neutrophils increased with disease activity in Black patients but not in White patients (Figure 2, G and H).

These data together suggest that CD8^+^ T cell, DN T cell, pDC, and neutrophil activations were more common in Black SLE ACT patients compared with White SLE ACT patients. We infer that the activated and inflammatory immune populations in whole blood are enriched in Black versus White populations during SLE disease.

### H3K27ac in CD8^+^ T cells is higher in Black patients and correlates with disease activity.

To investigate a potential relationship between immune cell epigenomes, race, and disease activity, we performed epigenetic landscape profiling using cytometry by time-of-flight (EpiTOF) (27, 28). The global levels of 40 chromatin modifications were measured on a single-cell level in 19 cell populations (Supplemental Figure 6). The cohort was split into 2 biological replicate groups with matching demographic distributions for testing (Supplemental Figure 7).

**Figure 3:**
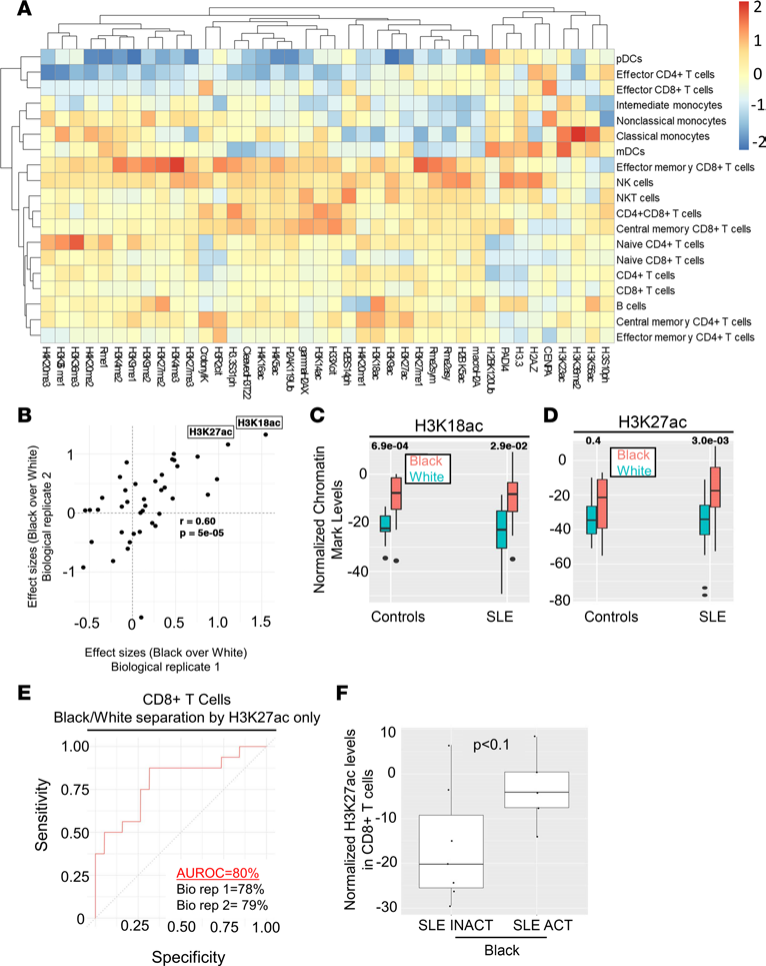
Changes in the CD8^+^ T cell epigenetic landscape distinguish Black from White patients and correlate with higher disease activity. PBMCs (*n* = 53) were used to assess global differences in 40 chromatin modifications in 19 immune cell subsets. (**A**) Average chromatin marker changes for 1 biological replicate comparing overall changes in cell subsets of patients with SLE versus controls. Heatmap depicts increased levels in red, no change in yellow, and decreased levels in blue. (**B**) Scatterplot depicting the effect size comparisons of chromatin marks in CD8^+^ T cells between Black and White patients between 2 biological replicates. Each dot represents the average levels of a chromatin marker. Correlation coefficient and the associated *P* value are shown. (**C** and **D**) Normalized chromatin marker levels of White and Black healthy controls and patients with SLE for (**C**) H3K18ac and (**D**) H3K27ac in CD8^+^ T cells. (**E**) Receiver operating characteristic (ROC) curves depicting the segregation of CD8^+^ T cells from Black patients and from White patients using the variance in Mark 4. Curves summarizing the results from both biological replicates are shown, with area under ROC percentages from independent replicates separately listed. Classification specificity (*x* axis) and sensitivity (*y* axis) are shown for the ROC curve. (**F**) Normalized H3K27ac levels in CD8^+^ T cells from patients with SLE INACT and SLE ACT. Mean ± SD is shown. Wilcoxon’s rank-sum test was used to determine statistical significance, and *P* values corrected for multiple hypotheses using FDR.

In most immune cell populations, the majority of profiled chromatin modifications were elevated in SLE patient cells compared with controls (Figure 3A). However, most chromatin modification levels in pDCs, effector CD4^+^ T cells, and effector CD8^+^ T cells from patients with SLE were reduced. Interestingly, patients with SLE could be distinguished from healthy controls with high sensitivity (>85%) and specificity (>70%) using levels of just 3 chromatin markers as proxies (H3K4me2, H3K4me3, and H4K20me1) (Supplemental Figure 8). These 3 chromatin markers had equal discriminatory capacity in both biological replicate groups.

We compared chromatin markers on 19 immune cell populations between patients with SLE and healthy controls stratified by race (Figure 3B). The levels of H3K18ac (Figure 3C) and H3K27ac (Figure 3D) were higher in CD8^+^ T cells in all Black versus White samples. Indeed, samples from Black patients could be distinguished from White patients with high sensitivity and specificity (AUC = 80%) using H3K27ac levels in CD8^+^ T cells alone (Figure 3E). Further, H3K27ac in CD8^+^ T cells was higher in Black SLE ACT patients compared with SLE INACT patients, but this correlation was not observed for White patients (Figure 3F). This was the only epigenetic marker found to significantly distinguish disease activity in either population. These findings suggest that immune cell epigenomes vary by racial background, both in healthy individuals and with different SLE disease activity states.

### IgG^+^ B cells are more frequent in Black patients with SLE ACT.

To investigate the transcriptional profiles of antigen-presenting cells in Black and White patients with SLE, we performed droplet-based scRNA-Seq (10x Genomics) and protein genomics (CITE-Seq) using CD2-depleted PBMCs from all study participants (Figure 1C). After filtering out cells of low quality and batch normalizing (Supplemental Figures 9 and 10), we obtained transcriptome data sets from about 90,000 cells, with over 30 million unique transcripts across all samples. Among these, 31,082 cells (35.3%) were from controls, 29,838 cells (33.9%) were from patients with SLE INACT, and 27,133 cells (30.8%) were from patients with SLE ACT (Supplemental Figure 11). Using graph-based clustering of uniform manifold approximation and projection (UMAP), we captured the transcriptomes of 8 major cell types or subtypes according to the expression of canonical gene and protein markers (Supplemental Figures 12 and 13).

**Figure 4:**
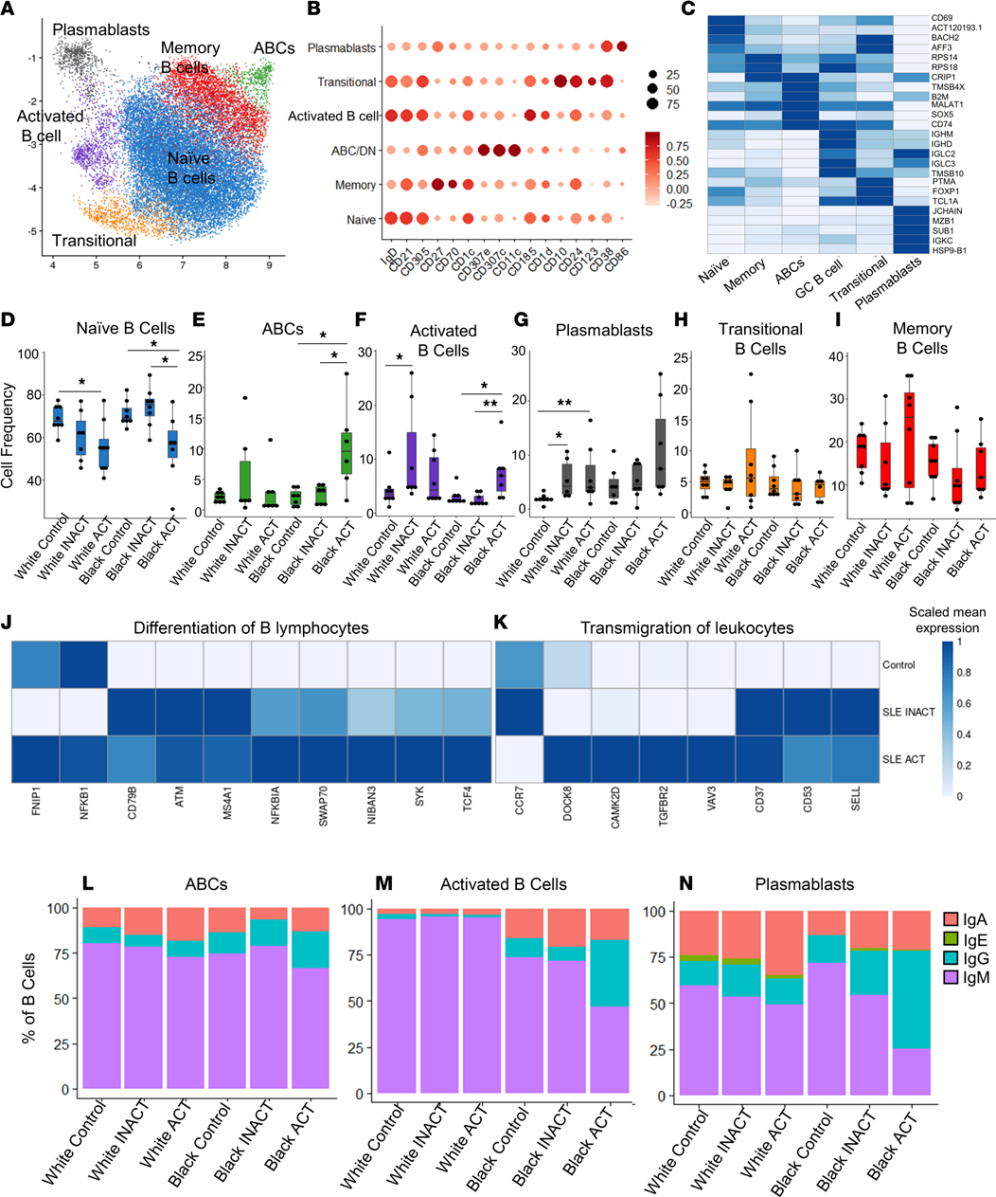
Alterations in the genomic landscape of patients with SLE ACT reveal greater IgG levels in Black patients. PBMCs from 46 controls and patients with SLE were CD2-depleted, followed by droplet-based scRNA-Seq using 10x Genomics. (**A**) UMAP plot representing the 6 B cell clusters across all samples. The putative identity of each cluster was assigned using gene expression and protein expression from CITE-Seq. (**B**) Dot plot representing expression values of selected proteins assessed by CITE-Seq and (**C**) heatmap representing gene expression values of selected genes across each cluster used for cluster annotation. Dot size represents the percentage of cells expressing the marker of interest. Color intensity indicates the mean expression within expressing cells. (**D**–**I**) Box plots comparing the proportion (mean ± SD) of each cell type cluster across the disease groups and ancestries for (**D**) naive B cells, (**E**) age-associated B cells (ABCs), (**F**) activated B cells, (**G**) plasmablasts, (**H**) transitional B cells, and (**I**) memory B cells as defined by scRNA-Seq. Box plots show the interquartile range (box), median (line), and minimum and maximum (whiskers). Ingenuity Pathway Analysis (IPA; QIAGEN) of differentially expressed genes identified differences in (**J**) differentiation of B cells and (**K**) transmigration genes of naive B cells between patients with SLE and controls. Heatmaps shows scaled mean expression of genes in each pathway. (**L**–**N**) The percentages of B cells with high gene expression of class-switched IgA, IgE, IgG, or IgM are shown by B cell subset in (**L**) ABCs, (**M**) activated B cells, and (**N**) plasma cells. *P* values were calculated using pairwise Wilcoxon’s rank-sum tests between disease groups with Benjamini-Hochberg correction for multiple comparisons. *P < 0.05, and **P < 0.01.

We used protein and transcriptome data to subdivide B cells into 6 subsets based on lineage markers (Figure 4, A–C). Independent of ancestry, increased disease activity was associated with a reduced frequency of naive B cells (Figure 4D). An increased frequency of age-associated B cells (ABCs), activated B cells, and plasmablasts (Figure 4, E–G), but not transitional or memory B cells (Figure 4, H and I), was also associated with active disease. The generally reduced frequencies of naive B cells in patients with SLE ACT were also observed by mass cytometry and coincided with increased gene expression of B cell differentiation and migration pathways (Figure 4, J and K). Within these pathways, gene expression of surface markers tended to be highest in patients with inactive disease, while expression of signaling components and transcription factors was highest in patients with SLE ACT. IFN and RNA viral infection gene expression pathways were elevated in plasmablasts from patients with high disease activity (Supplemental Figure 14). Increased levels of class-switching were also evident in patients with SLE ACT (Figure 4, L and M). The frequency of IgG^+^ B cells was higher across ABCs, activated B cells, and memory B cells from Black, but not White, individuals with active compared with inactive disease (Figure 4, L and N, and Supplemental Figure 15). White SLE ACT patients had greater frequencies of IgA^+^ plasmablasts compared with Black SLE ACT patients (Figure 4N). These results suggest that naive B cells may be more likely to differentiate and migrate during periods of heightened disease activity. They also support a model in which increased disease activity of Black versus White patients is associated with an increased frequency of cells producing high-affinity IgG antibodies.

### Myeloid transcriptomes define Black versus White patients’ SLE disease activity.

**Figure 5:**
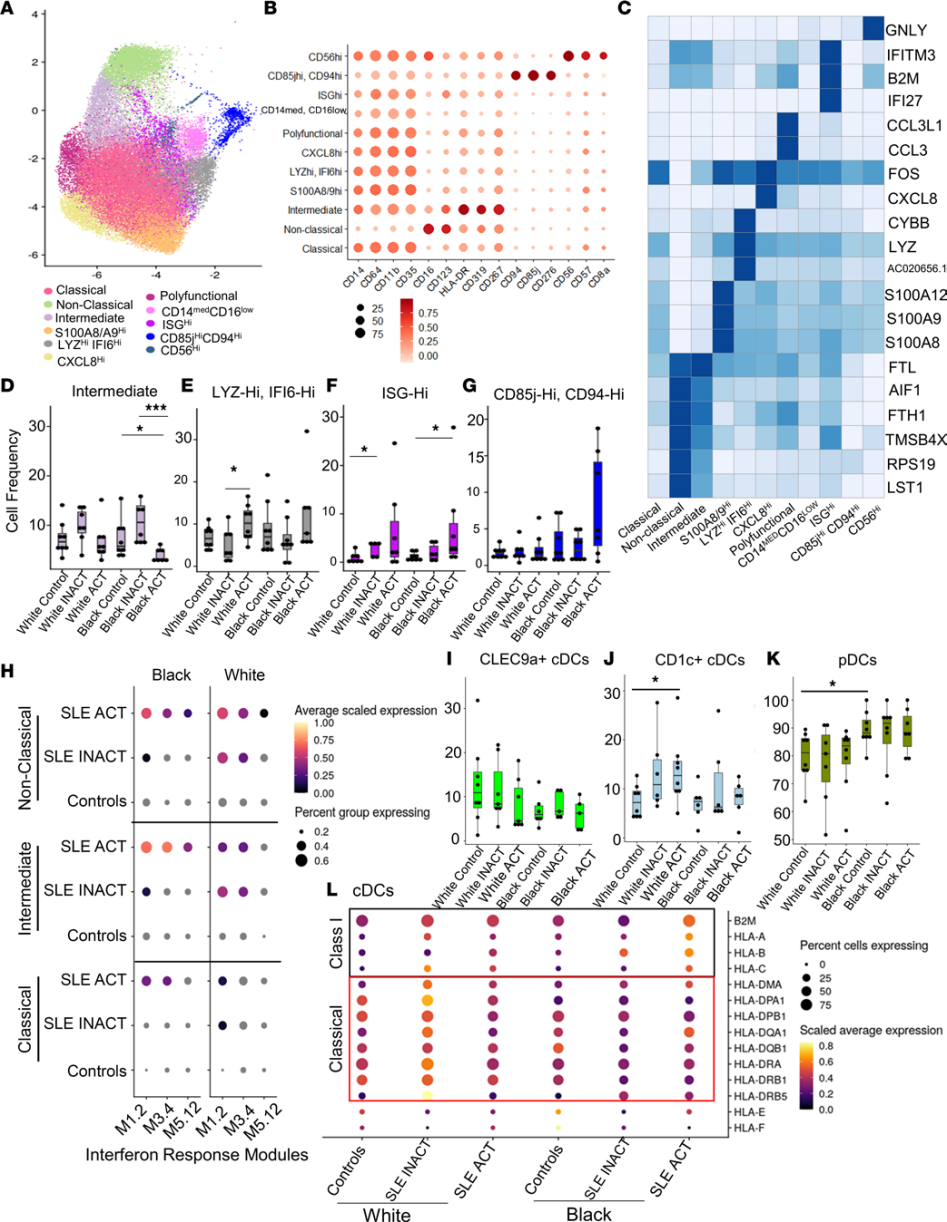
Monocyte ISG^hi^ transcriptional subsets are increased in patients with SLE ACT. PBMCs from 46 controls and patients with SLE were CD2-depleted, and droplet-based scRNA-Seq was performed using a 10x Genomics platform. (**A**) UMAP plot representing the 11 clusters of monocytes. The putative identity of each cluster was assigned using gene expression and protein expression from CITE-Seq. (**B**) Dot plot representing expression values of selected proteins assessed by CITE-Seq and (**C**) heatmap representing gene expression values of selected genes across each cluster used for cluster annotation. Dot size represents the percentage of cells expressing the marker of interest. Color intensity indicates the mean expression within expressing cells. (**D**–**G**) Box plots comparing the proportion of each cluster (mean ± SD) across the disease groups for (**D**) intermediate, (**E**) LYZ^hi^IFI6^hi^, (**F**) ISG^hi^, and (**G**) CD85j^hi^CD94^hi^ monocytes. Box plots show the interquartile range (box), median (line), and minimum and maximum (whiskers). (**H**) Gene expression for specific IFN response modules is shown using a dot plot for nonclassical, intermediate, and classical monocytes by disease group. Dot plots compare the proportion of the 3 different DC populations (mean ± SD) identified by UMAP, (**I**) CLEC9a^+^ cDC1s, (**J**) CD1c^+^ cDC2s, and (**K**) pDCs. (**L**) cDC gene expression of HLA antigen presentation components is shown via dot plot by disease group. HLA class I markers are boxed in black, and HLA class II markers are boxed in red. *P* values were calculated using pairwise Wilcoxon’s rank-sum tests between disease groups with Benjamini-Hochberg correction for multiple comparisons. *P < 0.05, **P < 0.01, and ***P < 0.001. ISG, IFN-stimulated gene.

Monocytes clustered into 11 unique populations based on protein and gene expression markers (Figure 5, A–C). The frequency of intermediate monocytes was reduced in Black SLE ACT patients compared with controls, whereas the frequencies of IFN signature–positive monocyte clusters were greater in White SLE ACT patients (LYZ^hi^IFI6^hi^, ISG^hi^) and Black patients (ISG^hi^) compared with controls (Figure 5, D–F). No significant differences were observed for all other monocyte populations (Figure 5G and Supplemental Figure 16, A–G).

Differences in IFN gene expression signatures in classical, nonclassical, and intermediate cell subsets were assessed using predefined gene expression modules (29, 30). White patients expressed similarly elevated levels of IFN gene expression modules regardless of disease activity (Figure 5H). In contrast, IFN pathway involvement in monocytes increased in Black patients with SLE ACT, particularly in intermediate monocytes (Figure 5H). Apoptosis pathway involvement was reduced in intermediate monocytes from patients with SLE ACT (Supplemental Figure 16H). These data are consistent with the hypothesis that expanded ISG^hi^ monocyte populations contribute to heightened disease activity in both White and Black patients, while greater IFN activation in intermediate monocytes in Black patients with SLE ACT may accentuate activation and increased migration into affected tissues.

Based on the transcriptome, DCs in our data set could be split into 3 distinct subsets of CLEC9a^+^ conventional DCs (cDCs), CD1c^+^ cDCs, and pDCs (Figure 5, I–K, and Supplemental Figure 16, I and J). CD1c^+^ cDCs were elevated with disease activity in White samples, whereas pDCs were globally higher in Black versus White samples independent of disease. HLA class I and II antigen presentation transcripts were differentially expressed in cDCs of patients with SLE (Figure 5L). Black patients with SLE ACT had the highest levels of HLA class I expression compared with all other groups (Figure 5L), whereas White patients with SLE trended toward higher expression of multiple HLA class II transcripts. As pDCs are characterized by IFN production, the increased prevalence of pDCs and elevated IFN levels observed in Black individuals as well as the increased expression of MHC class I in Black patients with high disease activity likely affect both disease course and severity.

### TLR7/9 and IFN-α signaling pathways are more prominent in Black patient immune cells.

**Figure 6:**
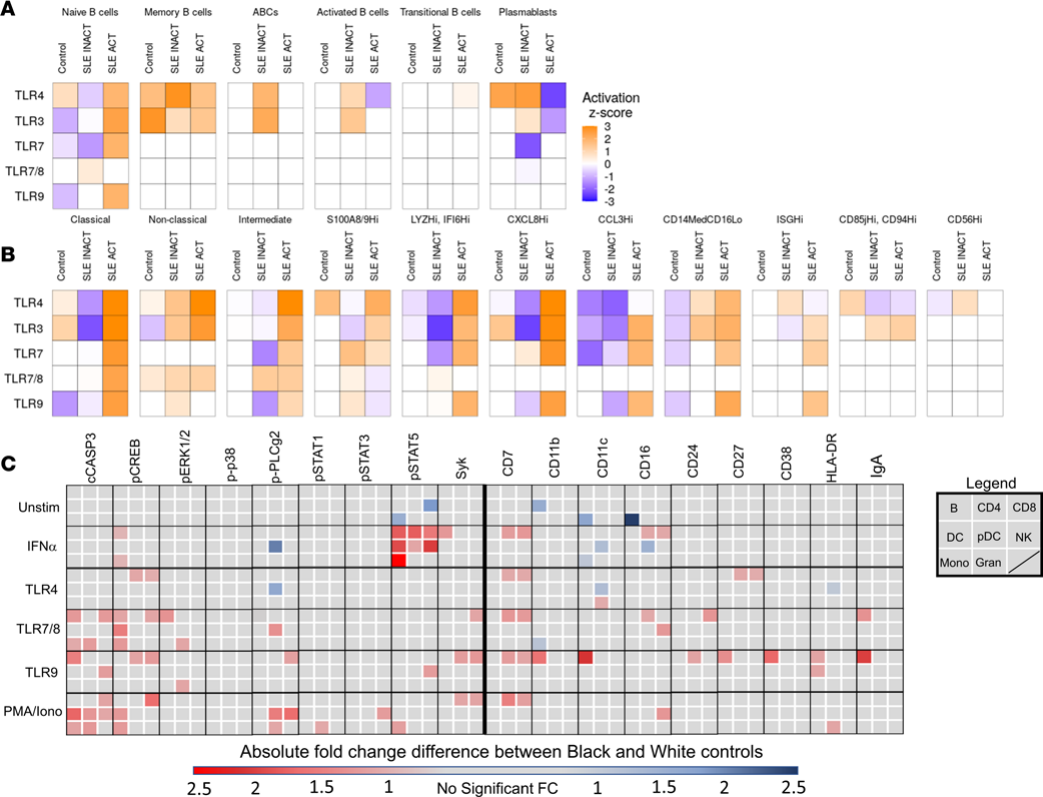
TLR activation pathways are elevated with disease activity in Black patients. Differentially expressed genes between Black and White samples identified by scRNA-Seq (*n* = 46) of (**A**) B cells and (**B**) monocyte cell clusters were assessed by IPA to determine ancestral differences in the activity of TLR pathways by activation *z* scores. Orange indicates increased TLR pathway activity in Black patients, white indicates no difference, and blue indicates increased TLR activity in White patients. (**C**) Peripheral whole blood (*n* = 56) from controls and patients with SLE was stimulated for 4 or 15 minutes with IFN-α and PMA/ionomycin or TLR4, TLR7/8, or TLR9 agonists. The median 95th percentile was used to calculate the fold-change of phospho-signaling and activation markers in Black versus White controls in 8 cell populations (see legend: B cells, CD4^+^ T cells, CD8^+^ T cells, DCs, pDCs, NK cells, monocytes [Mono], and granulocytes [Gran]). Statistical significance was determined using a Mann-Whitney *U* test and FDR *P* < 0.05, and significant changes are noted by a blue box (decrease) or a red box (increase).

Given that TLR7, TLR9, and type I IFN are key drivers of murine autoimmune disease, we investigated whether differences in TLR and IFN signaling pathways may underlie the observed ancestry-based differences in immune phenotypes in health and SLE disease. To assess differences in TLR activity, IPA was first used to evaluate differentially expressed genes in monocyte and B cell clusters identified by UMAP of scRNA-Seq (Figure 6, A and B, and Supplemental Table 12). Within each cluster, IPA calculated an activation *z* score of genes downstream of TLRs and their ligands predicting relative pathway activity in cells from Black patients compared with cells from White patients. TLR gene expression pathways were elevated in naive B cells and memory B cells from SLE ACT Black versus White patients (Figure 6A). TLR gene expression pathways were also elevated in monocytes from Black versus White SLE ACT patients, most notably among more activated/inflammatory monocyte populations: intermediate, CXCL8^hi^, CCL3^hi^, and CD14^lo^CD16^lo^ monocytes (Figure 6B). The TLR4 signaling pathway (LPS) showed the greatest elevation, as previously reported (21), but TLR3, TLR7/8, and TLR9 pathways were all elevated in Black versus White patients with SLE ACT.

To assess TLR signaling, we used phospho-CyTOF to monitor 9 signaling proteins and 8 activation markers, before and after a 4- or 15-minute stimulation of whole blood (Figure 6C, Supplemental Tables 13–20, and Supplemental Figures 17–25), using cell surface markers to discern 8 major cell lineages (Supplemental Figure 17). IFN-α–mediated induction of p-STAT5 was diminished in most cell lineages from Black patients versus Black healthy controls (Supplemental Figures 26 and 27). TLR7/8- and TLR9-induced phospho-signaling markers and activation proteins were also lower in multiple cell lineages from Black SLE patients compared with Black healthy controls (Supplemental Figures 26–30). Lower fold-changes were not a result of SLE patient cells being maximally stimulated, as they had lower total levels of phospho-signaling proteins following stimulation (Supplemental Figure 27B). TLR7/8- and TLR9-mediated induction of p-p38 and p-ERK1/2 were also lower in White patients versus White healthy controls, which may suggest exhaustion of these pathways from persistent stimulation in patients.

Significant differences in TLR signaling responses between Black and White healthy controls were also found. For instance, Black samples showed increased IFN-α–mediated induction of p-STAT5 as well as increased PMA/ionomycin-, TLR7/8-, and TLR9-mediated induction of cCASP3, p-CREB, p-ERK1/2, p-PLCy2, Syk, and p-STATs (Figure 6C). The TLR9 responses of B cells from healthy Black versus healthy White samples were particularly more prone to exhibit an activated phenotype, with higher expression of CD38, CD27, HLA-DR, CD11c, and CD11b after stimulation, due to a low threshold for activation or lack of appropriate regulation of cell signals. These data suggest that the TLR7/8/9 and IFN-α signaling pathways are enriched in immune cells of healthy Black versus White individuals — a baseline difference that may influence SLE disease manifestations and severity.

### Publicly available databases verify ancestry-dependent immune phenotypes and pathway alterations.

To further validate our scRNA-Seq, IFN signature, and TLR signaling pathway results, we used publicly available data deposited in National Center for Biotechnology Information Gene Expression Omnibus (NCBI GEO; accession number GSE135779) and IMMPORT (SDY40) (31, 32). In GSE135779, PBMCs from pediatric lupus patients and controls were split by ancestry into White and Black categories (Supplemental Table 21). Twenty unique cell clusters were identified and characterized based on gene expression relative to other clusters (Supplemental Figures 31 and 32). Similar to our earlier findings, plasma cells were elevated in Black SLE ACT versus Black SLE INACT and/or controls (Supplemental Figure 32). Furthermore, NK cells, specific subsets of CD8^+^ T cells, cDCs, nonclassical monocytes, and pDCs were all decreased in Black SLE ACT patients compared with Black SLE INACT and/or controls (Supplemental Figure 32). These data further indicate that ancestry-related immune cell profiles of patients with SLE contribute to the disease phenotypes.

To determine whether we could verify the transcriptomic differences seen between the White and Black SLE patient immune cells, we analyzed B cell pathways, IFN modules, and TLR-specific pathways from patient data in the published study GSE135779 (30). Again, we found B cells had greater gene activation for differentiation and migration (Supplemental Figures 33 and 34). IFN modules, as previously determined (29, 30), were examined in monocytes. Verifying our results, White patients had elevated IFN modules in both INACT and ACT disease. In contrast, Black patients had a greater IFN signature for ACT disease compared with INACT disease (Supplemental Figure 35). Black patients and controls also had elevated gene expression in most TLR-specific immune pathways of B cells and monocytes compared with White patients (Supplemental Figure 36, A and B).

To determine more directly whether TLR pathways are altered by ancestry, we examined shared data in IMMPORT SDY40 (32) (Supplemental Figure 37). White (*n* = 7) and Black (*n* = 7) young adult healthy controls were age-matched, and PBMCs stimulated with 7 agonists were assessed for differences in cytokine production. Black controls produced significantly more IFN-α in DCs and pDCs, more IL-6 in DCs, T cells, and NK cells, more IL-12 in NK cells and T cells, and more TNF-α in DCs following TLR7/8 stimulation (Supplemental Figure 36C). No differences were observed following TLR9 stimulation, likely because of the use of a TLR9 agonist that does not target B cells, unlike in our study. Together, these data verify that TLR pathways may be more strongly induced in immune cells from Black individuals.

### TLR stimulation recapitulates SLE immunophenotypes by ancestry.

We next hypothesized that the observed ancestry-enriched differences in TLR7, TLR9, and IFN signaling in healthy controls might contribute to the differences in the immune cell frequencies seen for patients with SLE (Figure 2A). To test this hypothesis, PBMCs were obtained from 10 Black and 10 White healthy controls (matched by age, sex, and BMI) and stimulated in vitro with TLR7/8 agonist, TLR9 agonist, or IFN-α alone or in combination for 7 days. All controls were female, had a mean age of 29.2 years, had no clinical symptoms of autoimmune disease as determined by a connective tissue screening questionnaire (CSQ) (33), were antinuclear antibody (ANA) negative by HEp-2 immunofluorescence and by Bio-Rad Bioplex 2200 ANA, and had no anti–cyclic citrullinated peptide (anti-CCP) or rheumatoid factor–specific (RF-specific) autoantibodies (data not shown).

**Figure 7:**
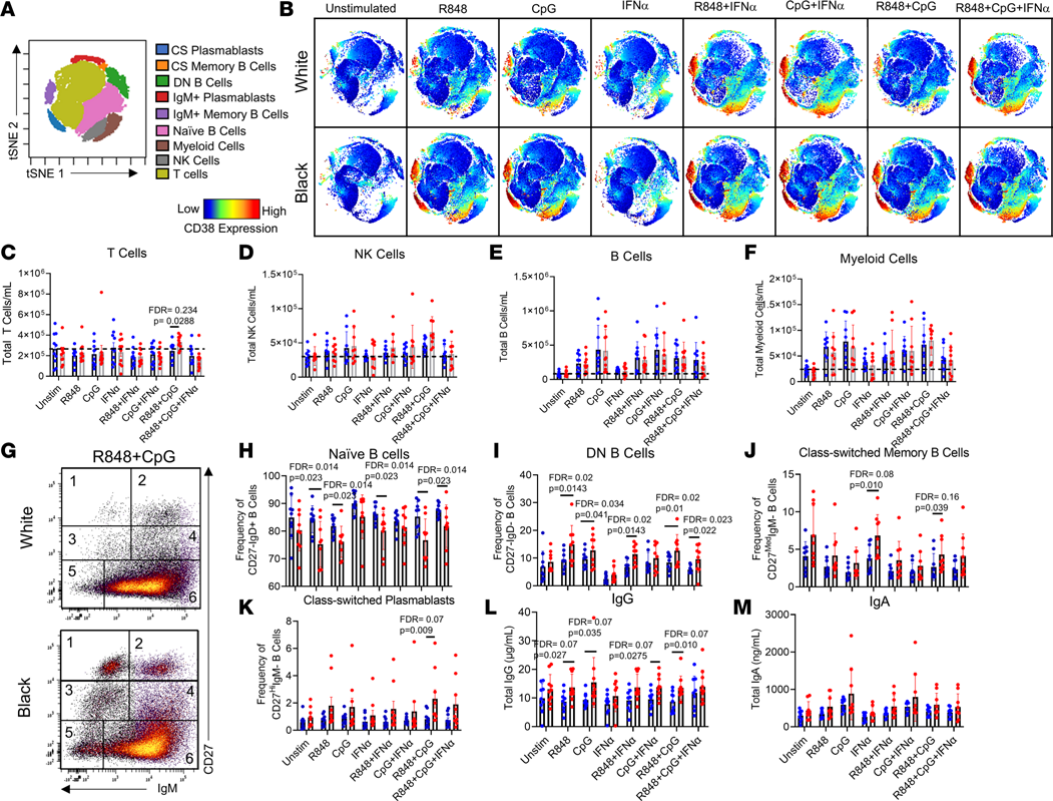
TLR stimulation of healthy Black and White immune cells recapitulates ancestry-associated SLE immunophenotypes. One million PBMCs from 10 White and 10 Black healthy controls, devoid of any autoimmune characteristics (ANA^–^, RF, CCP3 antibody negative, and negative CSQ), were stimulated with R848 (TLR7/8 agonist), CpG (TLR9 agonist), or IFN-α alone or in combination for 7 days and analyzed by flow cytometry. (**A**) tSNE plots identified 9 different immune cell populations across samples. (**B**) Representative tSNE plots of healthy White and Black cells following the 9 different stimulation conditions. CD38 expression is shown from red (high expression) to blue (low expression). Cells were counted in culture following 7-day stimulation, and total cell subset numbers were back-calculated using cell frequencies. The total (mean ± SD) (**C**) T cells, (**D**) NK cells, (**E**) B cells, and (**F**) myeloid cells/mL are shown for the 8 different stimulations by ancestry. (**G**) B cells were further subdivided by biaxial gating on IgM and CD27 to assess 6 different B cell subsets: 1) class-switched plasmablasts, 2) IgM^+^ plasmablasts, 3) class-switched memory B cells, 4) IgM^+^ memory B cells, 5) DN B cells, and 6) naive B cells. The frequency (mean ± SD) of (**H**) naive B cells, (**I**) DN B cells, (**J**) class-switched memory B cells, and (**K**) class-switched plasmablasts is shown for each condition. Supernatants were collected and assessed via ELISA for (**L**) total IgG concentrations and (**M**) IgA concentrations. Statistical significance was determined using a Mann-Whitney *U* test (*P* < 0.05), and all FDR *q* values were used for multiple comparisons.

The immune cell compositions following stimulation of PBMCs with TLR7/8 (R848) and/or TLR9 (CpG) agonists were markedly different for Black versus White healthy controls (Figure 7, A and B). In particular, the combination of TLR7/8 and TLR9 agonists resulted in a trend toward increased T cells (Figure 7C) in Black versus White healthy controls but no differences in total NK cells (Figure 7D), B cells (Figure 7E), and myeloid cells (Figure 7F). B cells were then classified into 6 subsets using CD27 and IgM gating via biaxial plots (Figure 7G and Supplemental Figure 38). Simultaneous treatment with TLR7/8 and TLR9 agonists resulted in fewer naive B cells and increased class-switched plasmablasts in Black versus White healthy controls (Figure 7, H–M). Black samples produced more DN B cells in response to TLR7/8 agonists with or without IFN-α and TLR9 stimulation (Figure 7I) and more class-switched memory B cells and class-switched plasmablasts in response to both TLR7/8 and TLR9 stimulation (Figure 7, J and K). IgG levels were also higher in Black versus White samples, but no differences were observed in IgA or IgM following TLR7/8, TLR9, or IFN-α stimulation (Figure 7, L and M, and Supplemental Figure 39). The response to simultaneous TLR7/8 and TLR9 stimulation in Black samples induced a stronger T cell and class-switched B cell response, whereas White individuals had predominantly more naive B cells. These changes broadly recapitulate the cell population variations we noted in Black and White patients with SLE.

### Elevated pro-inflammatory cytokines in Black patients with SLE and altered responses to TLR-driven cytokine pathways in patients with SLE.

**Figure 8:**
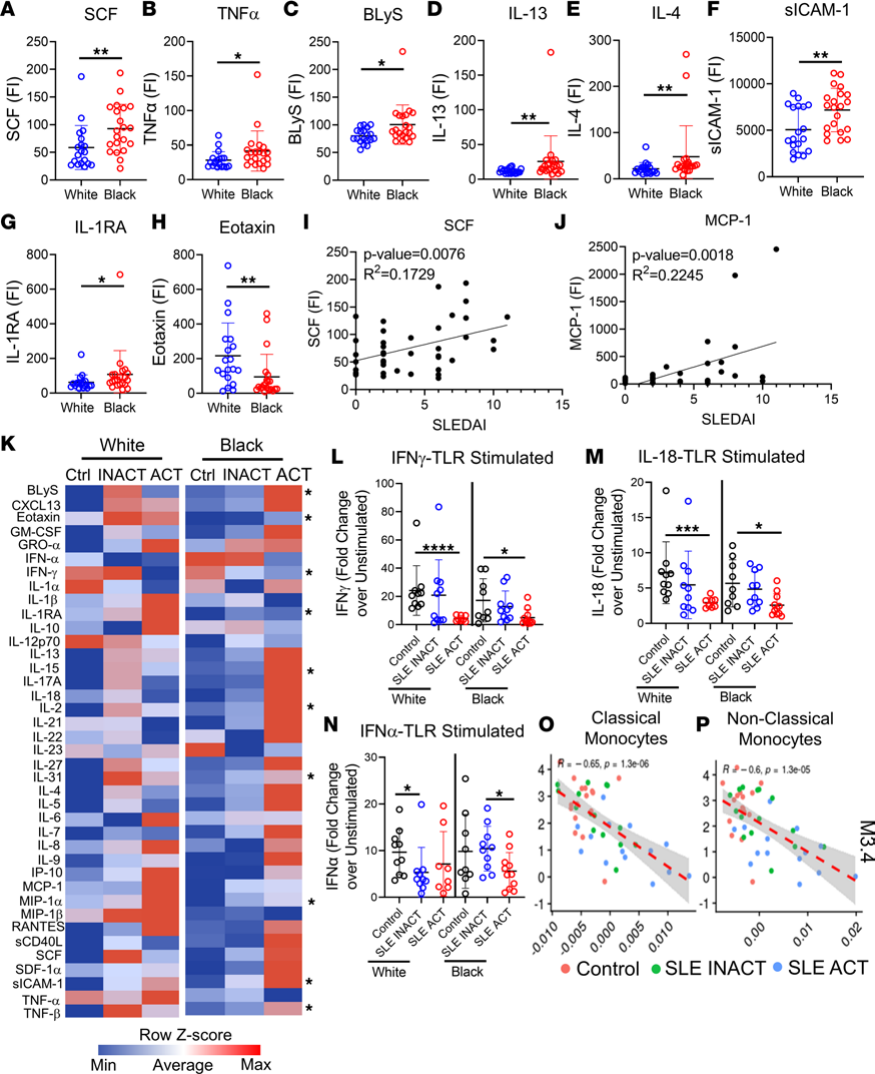
Pro-inflammatory cytokines vary in patients with SLE by ancestry and shape the TLR immune response. Pro-inflammatory soluble mediators were measured by multiplex or ELISA. Significant cytokine differences between Black (*n* = 21) and White (*n* = 19) SLE patients included increased (**A**) SCF, (**B**) TNF-α, (**C**) BLyS, (**D**) IL-13, (**E**) IL-4, (**F**) sICAM-1, and (**G**) IL-1RA in Black patients and increased (**H**) eotaxin in White patients. Linear regression analyses show (**I**) SCF and (**J**) MCP-1 to increase with SLE disease activity (SLEDAI) (*n* = 40). (**K**) A heatmap summary of the MFI supernatant levels following 24-hour whole blood stimulation with TLR4/7/8/9 agonists for each disease group is shown. Soluble mediator levels are displayed on a color scale ranging from blue (protein levels below the mean) to red (protein levels greater than the mean) using a column *z* score. Significant differences between Black and White disease groups are noted. The most significant fold-change differences over unstimulated culture samples were in the IFN pathways, including (**L**) IFN-γ, (**M**) IL-18, and (**N**) IFN-α. TLR-stimulated culture supernatant levels of IFN-γ negatively associated with mean ISG gene expression modules in (**O**) classical and (**P**) nonclassical monocytes by linear regression analysis. Statistical significance was determined using a Mann-Whitney *U* test (*P* < 0.05), and all FDR *q* values were used for multiple comparisons. Mean ± SD is shown. *P < 0.05, **P < 0.01, ***P < 0.001, ****P < 0.0001. FI, fluorescence intensity.

Next, we tested whether increased disease activity and altered immune responses in White and Black SLE patients were associated with alterations in chemokine and soluble mediator profiles. We assessed 39 mediators by multiplex bead-based assays or ELISA (Figure 1B) and found that plasma from Black SLE patients had increased levels of pro-inflammatory cytokines and other inflammatory regulators when compared with plasma from White SLE patients (Figure 8, A–G; Supplemental Figure 40, A–G; and Supplemental Tables 22 and 23). The chemokine eotaxin, which helps recruit eosinophils, was the only cytokine that was significantly increased in White versus Black patients (Figure 8H). SCF (or c-Kit) and MCP-1 levels best correlated with disease activity (Figure 8, I and J). These data suggest that higher levels of pro-inflammatory cytokines could contribute to enhanced disease activity in Black patients with SLE.

To determine whether dysregulated signaling pathways may contribute to the differences in soluble mediators, we stimulated whole blood from controls, SLE INACT, and SLE ACT patients with vehicle, R848/CpG/LPS (TLR7/8/9/4), PHA (T cell activation), or PMA/ionomycin (T cell activation) for 24 hours; collected supernatants; and then measured the levels of 39 soluble mediators (Figure 1, A and B, and Supplemental Figure 41). Cytokine levels were already significantly elevated in unstimulated culture supernatants of patients with SLE compared with controls, most notably in Black patients with SLE ACT (Supplemental Figure 40, H–L). Following TLR stimulation of SLE ACT whole blood, a subset of pro-inflammatory cytokines was increased, including CXCL13, IL-13, IL-6, SCF, and TNF-β, with other cytokines also trending higher (Figure 8K, Supplemental Figure 42, and Supplemental Tables 24 and 25). In contrast, TLR-mediated stimulation of type I and type II IFN cytokine pathways was specifically reduced in patients with SLE versus healthy controls, particularly for patients with SLE ACT (Figure 8, L–N). Diminished type I IFN responsiveness was specific to TLR stimulation, whereas type II IFN reduction was also observed following PHA and PMA/ionomycin stimulation (Supplemental Figures 43–45 and Supplemental Table 26–29). The reduction in TLR-induced IFN-γ levels was associated with higher IFN gene signatures in classical and nonclassical monocytes, suggesting that this may be due to exhaustion of the TLR/IFN cytokine pathway (Figure 8, O and P). The diminished IFN response was not significantly different between White or Black patients; however, White patients with SLE ACT produced higher levels of the regulatory cytokines IL-1RA and IL-10, which may mitigate TLR-induced inflammation (Supplemental Figure 42, B and C). The altered TLR response in SLE patients, with diminished early IFN production and increased pro-inflammatory cytokine levels, indicates external or internal stressors that trigger the TLR pathway may be associated with altered responses to infection or induction of disease flare. These alterations are more pronounced in patients with increased disease activity.

## Discussion

In this study, we identified significant variations in immune cell composition and cellular signaling responses triggered by viral pathways, which differ depending on ancestry and SLE disease status. We suggest that these differences help shape the immune phenotype in SLE, as supported by the stronger response of Black healthy controls to the TLR7/8, TLR9, and IFN-α stimulation pathways that are known to drive SLE pathogenesis. Using multiple profiling platforms, we detected higher TLR gene expression pathways, greater cell activation and phospho-signaling profiles, and amplified in vitro responses in immune cells from Black individuals. Past studies have demonstrated increased pro-inflammatory responses to bacterial pathogens and influenza in Black patients (21, 22), higher rates of inflammatory diseases (34), and greater SLE and COVID-19 disease severity (2, 35, 36). We speculate that enhanced TLR7/8/9 responses may contribute to the greater disease burden, earlier age of onset, and worsened disease symptoms reported for Black patients with SLE.

Ancestry-related differences in cell composition and the TLR7/8/9 and IFN-α responses may be explained by a combination of genetic and environmental factors (22). Benign neutropenia, which helps account for lower frequencies of neutrophils and higher lymphocyte counts in the circulation of Black individuals, has been associated with the rs2814778 (G) variant of the gene encoding atypical ACKR1, the Duffy antigen receptor for cytokines (37). It is possible that higher frequencies of TLR7- and TLR9-expressing cells in the circulation may contribute to greater expansion of these populations in response to stimuli in Black individuals. Higher expression of TLR7 and TLR9 have been reported in Black versus White SLE patient PBMCs (13). Women of Black descent were also found to carry significantly more allelic variants known to upregulate pro-inflammatory cytokines that may influence the strength of TLR7/8/9 responses (38). Other polymorphisms were found to associate with lupus risk and lupus nephritis risk specifically in Blacks, including *ENSA*, *IKBK/Chr8:* *Centromere*, *PCMTD1-ST18*, rs8091180-*NFATC1*, rs1050501-*FCGR2B*, rs6568431-*ATG5/PRDM1*, rs12136063-*SYPL2*, and rs2022013*-NMNAT2* (39, 40). Several of these genes are involved in T cell and B cell receptor activation and thus may contribute to the distinct ancestry-associated phenotypes observed with heightened lupus disease activity. Environmental factors, such as exposure to Epstein-Barr virus (EBV) and cytomegalovirus (CMV), trigger the same TLR pathways and may further prime immune responses to favor increased pro-inflammatory cytokine production while restricting the downregulation of these signals through ubiquitination pathways. Black individuals also have a disproportionally higher prevalence of EBV, CMV, and HSV-1 antibodies, which is associated with socioeconomic variables (41).

One of the clear differences in the immune responses of Black patients with SLE ACT was increased plasmablast, memory B cell, ABC, and activated B cell involvement, which generally supports a stronger B cell phenotype in Black patients with SLE (14–17, 42–44). However, this phenotype is not unique to SLE in the Black population. Healthy Black controls generated more IgG class-switched memory B cells and plasma cells in response to TLR7/8 and TLR9 stimuli. This expansion of IgG^+^ B cells, and the ability to class-switch to IgG, requires at least 3 cell divisions; T cell stimulation; and a proper cytokine milieu that includes IL-13, IL-4, or IL-10 (45). Consistent with this, the plasma of Black patients with SLE exhibited higher concentrations of IL-13 and IL-4 with greater T cell activation and CD38 and CD27 costimulatory marker expression.

We found that H3K18ac and H3K27ac epigenetic modifications in CD8^+^ T cells, both of which are associated with higher activation of transcription, were enhanced in Black patients with SLE ACT. We previously reported that H3K27ac is correlated with cytokine production, namely TNF-α, and treatment with the histone acetyltransferase inhibitor A-485 led to a concentration-dependent decrease in global histone H3K27ac levels that did not affect cell viability (46). Treatment with A-485 also led to a major reduction in the frequency of IL-1β and TNF-α, suggesting this may be a particularly useful therapeutic target for treating Black patients with SLE.

While heightened TLR gene expression pathways were found in patients with SLE, phospho-signaling responses following whole blood TLR7/8 and TLR9 stimulation were reduced. This is consistent with previous reports that correlated diminished responses to transcriptional signatures of cellular senescence, stress, and shortened telomere lengths (47). In the current study, such evidence of signaling exhaustion was enhanced in Black versus White patient samples with heightened disease activity. Hastened telomere shortening contributes to earlier aging and mortality in patients with SLE (48). Therefore, enhanced TLR7/8/9 signaling pathways that drive exhausted responses, stress, and telomere shortening may relate to earlier mortality in Black patients with SLE.

Patients with SLE, specifically those with heightened disease activity and lupus nephritis, are known to be particularly prone to infections, which are a leading cause of morbidity and mortality in SLE (49–51). While part of the high infection risk is likely due to immunosuppressive medications, alterations in the immune response due to SLE may also contribute to increased risk. Following stimulation with TLR agonists, SLE patients with high disease activity produced substantially fewer type I and II IFN and IFN-associated cytokines regardless of ancestry. This decreased IFN response was directly associated with higher IFN gene signature scores in monocytes at baseline. Although IFN levels were reduced compared with controls, other pro-inflammatory cytokine levels remained high, a phenotype consistent with aberrant and severe immune responses in certain viral infections (52, 53). White SLE ACT patients were distinguished from Black SLE ACT patients by higher expression levels of the regulatory cytokines IL-10 and IL-1RA, which may help dampen pro-inflammatory inflammation in the context of a dysregulated immune response. Exhausted IFN pathways from chronic inflammation in patients with SLE may contribute to greater infection risk and severity and lead to the disease flares that are more common in Black patients.

TLR7 and TLR9 pathway activation is also required for effective responses to vaccines and viral infections, such as COVID-19, which disproportionately affects Black individuals and Hispanics in both frequency and severity. Severe COVID-19 infection is characterized by a stronger CD8^+^ T cell response, whereas patients with mild COVID-19 have lower CD8^+^ T cell counts and a more exhausted CD8^+^ T cell phenotype (54). Additionally, patients with severe COVID-19 have an increased and more activated NK cell response (55). The multisystem inflammatory syndrome in children, which is more prevalent among Black and Hispanic children, is defined by a lack of exhausted CD8^+^ T cells that maintain the inflammatory environment (56). Black individuals are also reported to have higher antibody titers to rubella, measles, pertussis, and influenza vaccinations (57–60). These phenotypes are consistent with the heightened response to TLR7/8 and TLR9 pathways we observed in healthy Black control samples and are the same immune subsets altered during heightened disease in Black patients with SLE. While these responses may be helpful in the context of vaccination and promoting an effective memory response, they may predispose individuals of Black descent to more severe disease manifestations during infection and autoimmune disease.

Limitations of the current study include the modest sample size and the cross-sectional study design, which allows capture of the immune profile at only 1 time point. Although our cohort was matched on the Safety of Estrogens in Lupus Erythematosus National Assessment version of the SLEDAI (SELENA-SLEDAI) and clinical criteria (Supplemental Tables 1 and 2), SLE is very heterogenous, and Black and White SLE populations may not be perfectly matched both clinically and physiologically. Further, a large degree of separation in SELENA-SLEDAI was not present between activity groups. Future studies assessing drastically higher disease activity, such as in new-onset lupus nephritis, will be beneficial to examine more dramatic changes in the ancestry-associated phenotypes observed in this study. Nevertheless, the consistency of phenotype differences across multiomic platforms and other publicly available cohorts helps substantiate the ancestry-based results from this study.

These results provide insight into the factors driving immune composition and SLE disease activity in people of different ancestry. Heightened TLR7/8, TLR9, and IFN-α signaling pathways in immune cells from Black individuals support distinct immune phenotypes observed in response to infection, vaccines, and autoimmune disease. Increased CD8^+^ T cell, B cell, monocyte, and DC activity in Black individuals after stimulation of TLR7/8/9 may contribute to increased disease severity and early mortality in Black patients with SLE, and these pathways may be targetable for personalized treatments. Our results stress the importance of ancestry-based assessment in illuminating immune phenotypes that may be key to outcomes and treatment approaches across medicine.

## Methods

### Study population and sample collection.

Blood was collected at OMRF from 18 healthy controls (10 White and 8 Black) and 40 patients with lupus (19 White and 21 Black) over a 3-month period (February–May) following receipt of written consent. Ancestry was self-reported and verified using genetic ancestry informative markers (61). The degree of admixture was assessed by plotting the principal component (PC) values of all individuals with additional individuals from our Oklahoma Rheumatic Disease Cohort that included Asians, American Indians, Blacks, and Whites. Only individuals who clustered within the Black or White PC clusters, and exhibited no admixture, were used for this study. Clinical history and demographic data were recorded at the time of blood collection. Patients with SLE were divided by disease activity into either high (SLEDAI ≥ 4) or low disease activity (SLEDAI <4). No patients had a SLEDAI of 5. Individuals were matched across 6 ancestry/disease groups (White control, White SLE INACT, White SLE ACT, Black control, Black SLE INACT, Black SLE ACT) by sex, age ± 5 years, and medication use. Three patients were on biologics (1 on abatacept and 2 on belimumab); however, these patients were evenly distributed among groups and did not affect significance when dropped from the data set. Black SLE and White SLE patients were also matched by disease activity using SELENA-SLEDAI (62). Patients with SLE fulfilled the American College of Rheumatology and Systemic Lupus International Collaborating Clinics (SLICC) criteria for SLE classification (63). Whole blood was stimulated (see below) and stabilized, and samples were stored in liquid nitrogen and shipped frozen to the Human Immune Monitoring Center (HIMC) at Stanford University for mass cytometry analyses. PBMCs were isolated using Lymphocyte Separation Medium (Mediatech, Inc.) and stored in barcoded vials with freezing media (20% human serum/10% DMSO in RPMI). Plasma samples were collected and stored at –80°C until use.

### Autoantibody screening.

Patients with SLE and healthy controls were assessed for ANA positivity and specificity via indirect immunofluorescence (IIF) and 11 serum autoantibody specificities using the Bioplex 2200 system (Bio-Rad Technologies). IIF testing was performed by the CAP-CLIA certified Clinical Immunology Laboratory using NOVA Lite IIF (Inova Diagnostics, Inc.) following the manufacturer’s recommended protocols and cutoffs (64–66). Briefly, Bioplex 2200 ANA tested autoantibody specificities include dsDNA, chromatin, Ro/SSA, La/SSB, Sm, SmRNP, RNP, centromere B, ribosomal P, Scl-70, and Jo-1. All autoantibodies, except anti-dsDNA, were reported in Ab index (AI) units based on a fluorescence intensity range of 0–8. The manufacturer-specified cutoff was used to determine positivity (≥1 AI) for all auto-specificities, except for anti-dsDNA, where semiquantitative values were reported as IU/mL with a positive ≥ 10 IU/mL. APL Abs were tested using EIA kits (Bio-Rad). APL Ab positivity was defined as at least 1 aPL Ab IgG, IgM, or IgA ≥ 40 units.

### Whole blood stimulations.

Heparinized whole blood was collected and rested for 1 hour at 37°C. Whole blood (200 μL) was added to cluster tubes containing 50 μL of stimuli, either media, LPS (Final Conc: 1 μg/mL), R848 (10 μM), CpG (10 μg/mL), PMA/ionomycin (PMA: 10 ng/mL; ionomycin: 1 μg/mL), or IFN-α (10,000 U/mL) for 4 minutes (TLR stimulations) or 15 minutes (IFN-α, PMA/ionomycin). Cells were immediately stabilized with a proteomic stabilizer (SmartTube, Inc) following the manufacturer’s instructions and stored at –80°C until phospho-CyTOF staining and sample acquisition. Stimuli were purchased from either MilliporeSigma (PHA, PMA, LPS, and ionomycin), PBL Assay Source, or InvivoGen (CpG ODN 2006 and resiquimod [R848]). A second aliquot of whole blood was diluted 1:2 (400 μL total) with complete RPMI (RPMI supplemented with 10% fetal calf serum, penicillin-streptomycin, and glutamine) in cluster tubes. Either 100 μL media (unstimulated), PHA/ionomycin (1 mg/mL each), LPS/R848/CpG (1 mg/mL, 10 μM, and 10 mg/mL), or PMA/ionomycin (10 ng/mL, 1 μg/mL) were added to cluster tubes. Cells were incubated overnight (24 hours), after which cells were pelleted and supernatants collected for soluble mediator assessment.

### Phospho-signaling mass cytometry staining and acquisition.

Phospho-CyTOF was performed at the HIMC at Stanford University. Upon thawing, samples were washed and surface stained for 30 minutes at room temperature (Supplemental Table 4). Cells were washed twice, permeabilized with 100% methanol, and kept at –80°C overnight. The next day, cells were washed and resuspended in an intracellular Ab cocktail for 30 minutes before washing twice. Cells were resuspended in a 100 mL iridium-containing DNA intercalator and incubated for 20 minutes. Cells were washed once with cyFACS buffer and twice with MilliQ water (MilliporeSigma). Cells were diluted to 750 × 10^5^ cells/mL in MilliQ water and acquired on Helios (Fluidigm). Data analysis was performed using viSNE and biaxial gating in Cytobank to determine cell populations (Supplemental Figure 2), and fold-change of the 95th percentile was calculated for all 9 signaling molecules and 8 major cell populations.

### Epigenetic landscape profiling using mass cytometry (EpiTOF).

As part of the Autoimmunity Center of Excellence (ACE) Network Collaborative Project, EpiTOF was performed as previously described (28) on 53 of the 58 individuals in this study (10 White control, 10 SLE INACT, 8 SLE ACT, 8 Black control, 9 Black SLE INACT, 8 SLE ACT). Black and White samples were stained with an EpiTOF Ab panel containing 15 surface markers, 40 chromatin modification markers, and 2 histone controls (Supplemental Figure 9). Briefly, PBMCs were thawed and incubated in RPMI 1640 medium (Thermo Fisher Scientific) with 10% FBS (ATCC) for 1 hour prior to processing. Cisplatin (10 μM) (ENZO Life Sciences) was added for viability staining for 5 minutes before quenching with CyTOF buffer. Cells were washed and stained with 15 immunophenotypic markers. Following extracellular marker staining, cells were washed 3 times and fixed in 1.6% paraformaldehyde (PFA) (Electron Microscopy Sciences) for 15 minutes at room temperature (RT). Cells were permeabilized with 1 mL ice-cold methanol (Thermo Fisher Scientific) for 20 minutes at 4°C. CyTOF buffer was added to stop permeabilization, followed by 2 PBS washes. Mass-tag sample barcoding was performed following the manufacturer’s protocol (Fluidigm). Individual samples were then combined and stained with 42 intracellular Abs in CyTOF buffer containing Fc receptor blocker (BioLegend) overnight at 4°C. The following day, cells were washed twice and stained with 191/193Ir DNA intercalator (Fluidigm) in PBS with 1.6% PFA for 30 minutes at RT. Cells were washed, filtered, resuspended with calibration beads, and analyzed on the Helios system in the Stanford Shared FACS Facility. A total of 200,000 cells were collected for each participant in the study. Individuals were split into 2 biological replicates with matched demographics distributed evenly between Black and White individuals.

### In vitro TLR7, TLR9, and IFN-α stimulations with healthy controls.

PBMCs from 20 healthy controls were recruited through health fairs, as previously described (23). A total of 10 White and 10 Black healthy individuals were selected for in vitro stimulation studies. To ensure no individuals had any autoimmune disease manifestations, only controls who were ANA negative by both IIF and Bioplex, who were negative for CCP and RF Abs, and who had scored 0 on the CSQ in all autoimmune disease categories were selected. Individuals were all women and were matched across ancestries by age and BMI. Cells were plated at 1 × 10^6^ cells/mL (900 μL) in a 24-well plate, where 100 μL of stimuli was added in the following stimulation conditions: media (unstimulated), R848, CpG, IFN-α, R848+IFN-α, CpG+IFN-α, R848+CpG, R848+CpG+IFN-α. R848 and CpG were both used at a final concentration of 300 ng/mL, while IFN-α was used at 1,000 U/mL. Cells were incubated with stimuli at 37°C and 5% CO_2_ for 7 days. Plates were spun down, and supernatants were collected and stored at –80°C until use for Ab ELISAs. Cells were collected for B cell and other cell subset assessment using an 8-marker Ab panel for flow cytometry. Cells were washed, incubated in Fc block (BD Biosciences), and stained with CD3 (UCHT1), CD19 (HIB-19), CD27 (L128), CD38 (HIT2), IgD (I-A62), CD11c (B-Ly6), IgM (G20-127), and CD24 (ML5) from BD Biosciences at the manufacturer’s recommended concentrations. Cells were washed and immediately run on an LSRII (BD Biosciences). Cells were gated as described in Supplemental Figure 38.

### Total Ab ELISAs.

IgM, IgA, and total IgG ELISAs (Thermo Fisher Scientific) were run on culture supernatants following a 7-day PBMC incubation for unstimulated and stimulated samples. Samples were thawed after storage at –80°C and diluted. The assays were performed according to the manufacturer’s instructions. A dilution of 1:50 for IgM, 1:40 for IgG, and 1:20 for IgA was used to calculate mg or ng/mL.

### Soluble mediator measurements.

Plasma levels of BLyS, CXCL13, eotaxin, GM-CSF, GRO-α, IFN-α, IFN-γ, IL-1α, IL-1β, IL-10, IL-12p70, IL-13, IL-15, IL-17A, IL-18, IL-2, IL-21, IL-22, IL-23, IL-27, IL-31, IL-4, IL-5, IL-6, IL-7, IL-8, IL-9, IP-10, MCP-1, MIP-1α, MIP-1β, RANTES, sCD40L, SCF, SDF-1α, sICAM-1, TNF-β, TNF-α, and IL-1RA were assessed by xMAP multiplex assay (eBioscience, Thermo Fisher Scientific). Data were obtained using a Bio-Rad Bioplex 200 system. Further, whole blood culture supernatants were also assessed using xMAP multiplex assays.

### Statistics.

tSNE analyses were performed using Cytobank Premium software as a service on the Amazon Web Services cloud (67). FCS files were uploaded to Cytobank and gated off live, intact, singlet cells. Concatenated files of 430,000 cells were used for representative tSNE images and cell subset profiling. Frequencies of cell subsets were exported from tSNE for cell number calculations and analysis. Traditional bivariant gating was performed in Cytobank. Cytokine data were non-normally distributed; therefore, continuous data were analyzed using the Kruskal-Wallis test in TIBCO Spotfire (version 11.4.0 LTS) with Wilcoxon-Mann-Whitney 2-tailed test for 2-group comparisons. The *q* values were calculated using Prism (version 9.2.0) for Windows (GraphPad Software) with 2-stage step-up method of Benjamini, Krieger, and Yekutieli to correct for multiple comparisons and estimate the false discovery rate to control for the expected proportion of incorrectly rejected null hypotheses. All dot plots were generated using GraphPad Prism or R package, and heatmaps were generated using TIBCO Spotfire.

### Study approval.

All experiments using human samples were conducted in accordance with the Declaration of Helsinki and approved by the Institutional Review Board at the OMRF. Inclusion in the study was documented through written informed consent.

### Code availability.

All code used in scRNA-Seq and CITE-Seq analysis can be found in GitHub at https://github.com/KevinTThomas/sle_activity_scrnaseq (commit ID 58eaf62). The raw files for CyTOF, EpiTOF, and cytokine-multiplex bead array assays are uploaded to IMMPORT and will be available upon acceptance under the study title “Molecular Heterogeneity of SLE Disease Activity” and study ID SDY1879. IMMPORT link to access is provided here: https://immport.niaid.nih.gov/research/study/studysearchmain#!/studysearch/viewStudyDetails/subjectsSDY1879 All other data are available within the manuscript and its supplement.

### Data availability.

All RNA-Seq and CITE-Seq data that support the findings of this study have been deposited in GEO, accession number GSE189050. Reviewers can access scRNA-Seq data on GEO through token uvoxsmkchjadzkn. All other data (CyTOF, EpiTOF, and cytokine-multiplex bead array assays) are uploaded to IMMPORT and available under the study title “Molecular Heterogeneity of SLE Disease Activity” and study ID SDY1879. All supporting data values associated with the main manuscript and supplemental material, including values for all data points shown in graphs and values behind any reported means, are reported in the additional Supporting Data Values document. Further information can be found in Supplemental Methods.

## Author contributions

JMG, JAJ, SSW, and KT conceptualized the study. JMG, JAJ, SSW, KT, MS, PJU, and PK were responsible for developing the methodology, and SSW, KT, MS, CAW, SM, AB, MD, MD, SEC, AK, PC, CW, JTM, EC, CA, HTM, PK, PJU, JAJ, and JMG were responsible for the experimental investigation. SSW, KT, MS, JMK, LK, AG, DD, CJG, WD, MHF, JAJ, and JMG were responsible for visualization. Funding was acquired by JMG, JAJ, PJU, and SSW. JMG and JAJ were responsible for project administration, and JMG, JAJ, SSW, PJU, and PK were responsible for project supervision. SSW, KT, MS, CAW, SM, AB, MD, MD, SEC, AK, PC, LK, AG, DD, CJG, WD, CW, MHF, JTM, EC, CA, HTM, PK, PJU, JAJ, and JMG were responsible for writing and editing. SSW was listed first because SSW was responsible for all experimental designs and data interpretation and drafted and revised the manuscript.

## Supplementary Material

Supplemental data

Supporting data values

## Version 1 08/22/2023

Electronic publication
