# Supplementary material for: Ancestry-based differences in the immune phenotype are associated with lupus activity
Source: JCI Insight. 2023 Aug 22;8(16):e169584. doi: 10.1172/jci.insight.169584 (PMC10543734; doi:10.1172/jci.insight.169584)
Supplement: Supplemental data [file jciinsight-8-169584-s225.pdf]

SUPPLEMENTAL INFORMATION

Ancestry-based differences in the immune phenotype are associated with lupus activity

Authors: Samantha Slight-Webb[†1], Kevin Thomas[†1,2], Miles Smith[1], Susan Macwana[1], Aleksandra Bylinska[1,2], Michele Donato[3,4], Mai Dvorak[3,4], Sarah Chang[3], Alex Kuo[5], Peggie Cheung[5], Laurynas Kalesinskas[3,4], Ananthakrishnan Ganesan[3,4], Denis Dermadi[3,4], Carla Guthridge[1], Wade Dejager[1], Christian Wright[1], Mariko Foecke[5], Joan T. Merrill[1], Eliza Chakravarty[1], Cristina Arriens[1,2], Holden T. Maecker[6], Purvesh Khatri[3,4], Paul J. Utz[5], Judith A. James[1,2‡], Joel M. Guthridge[1,2‡*]

Affiliations:

[1]Department of Arthritis and Clinical Immunology, Oklahoma Medical Research Foundation; Oklahoma City, OK, 73104, USA.

[2]Departments of Medicine and Pathology, University of Oklahoma Health Sciences Center; Oklahoma City, OK, 73104, USA.

[3]Institute for Immunity, Transplantation and Infection, Stanford University School of Medicine; Stanford, CA, 94305, USA.

[4]Center for Biomedical Informatics Research, Department of Medicine, Stanford University School of Medicine; Stanford, CA, 94305, USA.

[5]Division of Immunology and Rheumatology, Stanford University School of Medicine; Stanford, CA, 94305, USA.

[6]Institute for Immunity, Transplantation and Infection, Stanford University School of Medicine; Stanford, CA, 94305, USA.

†These authors contributed equally to the work.

‡ These authors jointly supervised this work.

Competing interests: OMRF has licensed intellectual property of JAJ to Progentec Biosciences. Otherwise, the authors declare no competing interests.

*Corresponding Author:
Joel M Guthridge, PhD
Arthritis and Clinical Immunology
Oklahoma Medical Research Foundation
825 N.E. 13th Street
Oklahoma City, OK 73104
Phone: (405) 271-4987
Fax: (405) 271-7063
Email: Joel-Guthridge@omrf.org

SUPPLEMENTAL METHODS

Single Cell RNA sequencing (scRNA-seq). PBMCs were thawed at 37ºC and resuspended in complete RPMI supplemented with 5 U/mL benzonase. Complete RPMI consists of RPMI media 10% FBS, 100 U/mL Penicillin, 100 U/mL Streptomycin, and 2 mM glutamine. PBMCs were washed once in this media and resuspended in complete RPMI supplemented with benzonase and 3 µM actinomycin D. PBMCs were depleted of T cells and any remaining erythrocytes using CD2 and CD235a MicroBeads (Miltenyi Biotec). Depletion was carried out according to protocol by Miltenyi Biotec with MACS buffer supplemented with 3 µM actinomycin D.

CITE-seq and Cell Hashing

Approximately two million post-depletion PBMCs from each sample were stained with a panel of oligo-tagged antibodies following CITE-seq and Cell Hashing as detailed by the New York Genome Center (NYGC); separately, one million NIH/3T3 cells (ATCC CRL-1658) were stained with the same antibody panel as a negative control. Staining buffer was supplemented with 3 µM actinomycin D, and the CITE-seq antibody panel consisted of TotalSeq-A antibodies (BioLegend) or were conjugated in house using the Thunder-Link® PLUS oligo antibody conjugation kit (Novus Biologicals). A full list of antibodies can be found in Figure 1 panel C. Cells were stained with 0.1 µg of each CITE-seq antibody in a total of 100 ul staining buffer. Cell hashing antibodies were also obtained from BioLegend. Cell numbers and viability were obtained on a Nexcelom Cellometer Auto 2000 using acridine orange/propidium iodine staining. Cells from 4-5 samples were combined with 3% NIH 3T3 cells in batches to a target total of 16,000 – 20,000 cells immediately before encapsulation.

Encapsulation, Library generation, and Sequencing

Cells were captured and encapsulated using the Chromium Single Cell 3' Gene Expression system, v2 (10X Genomics). Libraries for sequencing were prepared according to the manufacturer's protocol (revision F); separately, CITE-seq and hashing libraries were prepared according to the CITE-seq and cell hashing protocol (version 2019-02-13). Gene expression, CITE-seq, and hashing libraries were pooled at an 85/10/5 mass ratio. Each library set was sequenced in a NextSeq HO PE75 lane according to recommendations by 10X Genomics and the NYGC to target approximately 50,000 reads per cell. Additional sequencing for runs 3a-5b was performed on one lane of a NovaSeq S4 PE150. All sequencing was performed by the OMRF Clinical Genomics Core.

Preprocessing

Raw sequencing data was processed using the Cell Ranger v5.0.0 analysis pipeline on the OMRF High Performance Compute cluster. Sequences were aligned to the chimeric mm10 GRCh38 reference available from 10x Genomics (2020-A). Separate feature-cell matrices for gene expression and antibody feature libraries were generated for each sample pool. These matrices were further processed in R (4.1.1) using a custom pipeline running on the Google Cloud Engine platform. Ambient RNA was identified and removed using SoupX (1.5.2) (1). This was done separately for each sample pool using the set of hemoglobin genes to estimate contamination before assembling all batches into a single object with Seurat (4.0.4) (2). Mouse cells were separated from human cells in each batch by visual gating on biaxial plots of counts aligned to human and mouse genomes (Figure S10A). Assignment of hashtag IDs for sample deconvolution

was done by applying two demultiplexing algorithms, HTODemux (Seurat) and MULTISeqDemux (DeMULTIplex [1.0.2] (3)), to the human cells in each batch and choosing the assignment based on which method gave the highest proportion of singlets (Figure S10B). After selection of identifiable singlets, quality control cutoffs for number of genes per cell and percent mitochondrial counts were set across all batches at 40 and 25%, respectively (Figure S10C).

Analysis

After preprocessing and quality control steps, the cells were scored for differences in the immunoglobulin heavy chain and light chain constant genes. These differences were regressed out along with mitochondrial content and hemoglobin content during normalization and scaling by SCTransform (Seurat). This was done to prevent B cell subsets from separating solely on the large differences between highly expressed immunoglobulin genes. CITEseq data for epitope expression was independently normalized with the dsb (denoised and scaled by background) package (0.2.0) (4), using the discarded mouse cells for background levels of all antibody barcodes. Principal component analysis (PCA) was performed on normalized gene expression and epitope matrices to generate dimensional reductions, and harmony (0.1.0) (5) was performed on both of these PCA reductions to eliminate batch effects. Batch-corrected PCAs of gene expression and epitope multimodal data were integrated using Seurat's weighted nearest neighbor (WNN) analysis (2), with the number of PCs of each modality selected visually by the elbow method. Uniform manifold approximation and projection (UMAP) and clustering by the Leiden algorithm were performed on this weighted nearest neighbor network to generate an initial mapping of the dataset.

Resolution was selected based on the best separation of canonical cell type markers. Clusters were annotated by hand, with top gene and surface epitope markers generated by Wilcoxon rank sum tests. The two major populations of interest, B cells and myeloid cells, were isolated for further manual adjudication and quality control. These populations were re-analyzed independently of other cells, and clusters with canonical gene/epitope expression indicating doublets with low counts or large amounts of ambient RNA contamination were removed. Additionally, clusters that expressed high levels of hemoglobin transcripts or megakaryocytic genes were also removed from the final analysis. After manual quality control, a total of 88,053 cells were analyzed. Clusters were manually annotated by expression of canonical genes and epitopes, clusters smaller than 10 cells were merged into their nearest neighbors, and clusters containing multiple identifiable subpopulations were subclustered.

Gene Expression Analysis

Differential expression of genes and epitopes between clusters (marker identification) was performed using Wilcoxon rank sum tests of the normalized expression values of genes and antibody tags. These identified markers by comparing each cluster to all other cells in the dataset. Benjamini-Hochberg correction for multiple comparisons was performed, with a cutoff threshold of 0.1.

Expression of interferon gene modules at the single-cell level was calculated using Seurat’s AddModuleScore function. These gene modules were previously identified and annotated as interferon response signatures in previous work (6, 7).

Within each cluster, differential expression of genes between study groups was carried out by Model-based Analysis of Single-cell Transcriptomics (MAST) (8). This package fits two-part linear models specifically adapted for single-cell gene expression data. Comparisons were done to contrast two study groups at a time, with a total of 12 comparisons total: all SLE INACT vs all Control, all SLE ACT vs all Control, all SLE ACT vs all SLE INACT, Black Control vs White Control, Black SLE INACT vs White SLE ACT, Black SLE ACT vs White SLE ACT, Black SLE INACT vs Black Control, Black SLE ACT vs Black Control, Black SLE ACT vs Black SLE INACT, White SLE INACT vs White Control, White SLE ACT vs White Control, White SLE ACT vs White SLE INACT. Each comparison was filtered for genes expressed in at least 5 percent of cells (a minimum of 10 cells) in one of the compared groups. For the models, fixed effect terms were included for the per-cell gene detection rate, batch, and race (as a blocking variable, for applicable comparisons). A random effect term was included for the subject identifier.

Ingenuity® Pathway Analysis

Results from the differential expression testing of each comparison by MAST were used as input to individual core analyses in Qiagen's Ingenuity® Pathway Analysis (IPA). Genes from each comparison were filtered on a p-value less than 0.05 to provide the broadest possible analysis of differentially expressed genes. Depending on the cluster and comparison, these analyses included between 12 and 1360 molecules, with a median of 119 molecules. Upstream Regulator and Function Annotation results were displayed as networks connecting DEGs to the terms of interest. Results from comparison analyses are shown as heatmaps or tables of activation z-scores.

Second phenotype cohort using a PBMC CYTOF dataset

This experiment was performed in accordance with the Helsinki Declaration and approved by the Institutional Review Board of the Oklahoma Medical Research Foundation. ANA-healthy controls and SLE patients were divided and analyzed by race and disease activity, which was self-reported and verified using genetic ancestry informative markers (AIMs) (9). SLE patients were divided by disease activity into either high (SLEDAI ≥4) or low disease activity (SLEDAI <4). Individuals were matched across 6 ancestry/disease groups (White Control, White SLE INACT, White SLE ACT, Black Control, Black SLE INACT, Black SLE ACT) by sex, age ± 5 years, and medication use. Black SLE and White SLE patients were also matched by disease activity using SELENA-SLEDAI (10). SLE patients fulfilled the American College of Rheumatology (ACR) and SLICC criteria for SLE classification (11). Peripheral blood mononuclear cells (PBMCs) were isolated using Lymphocyte Separation Medium (Mediatech, Inc. Manassas, VA) and stored in freezing media (20% human serum and 10% DMSO in RPMI) in liquid nitrogen until use.

Assays were performed in the Human Immune Monitoring Center at Stanford University. Antibody clones, staining protocols, and gating strategies are previously described(12). Briefly, PBMCs were thawed, washed, and resuspended in CyFACS buffer (PBS supplemented with 2% BSA, 2 mM EDTA, and 0.1% sodium azide), and viable cells were counted by Vicell. Viable cells (1.5 million cells/well) were stained with antibody-polymer conjugate cocktail (Supplemental Table 9). All antibodies were unconjugated and carrier free, and conjugated using polymer and metal isotopes from Fluidigm. Cells were washed and resuspensed in CyFACS buffer. The cells were resuspended in 2 ug/mL Live-Dead (DOTA-maleimide [Macrocyclics] containing natural-

abundance indium). The cells were washed and resuspended in 2% paraformaldehyde in PBS and placed at 4°C overnight. The next day, cells were washed and placed in eBiosciences/ThermoFischer permeabilization buffer (1x in PBS) and incubated on ice. Cells were then washed twice in PBS and acquired on CyTOF (Fluidigm, San Francisco, CA). Data analysis was performed using Cytobank by gating on intact cells based on the iridium isotopes from the intercalator, then on singlets by Ir191 vs cell length, then on live cells (Indium-LiveDead minus population), followed by cell subset-specific gating as shown by tSNE and manually, as previously published(12) and described here in Supplemental Figure 2. Cells frequencies were back-calculated as if from whole blood using complete blood count (CBC) frequency information using neutrophils, lymphocytes, and monocyte information. Data was combined with the 58 subjects in primary cohort.

Confirmatory analysis in a second single cell dataset

An analysis of the single cell data from GSE135779(13) was carried out to confirm our findings. Analysis of PBMC immunophenotypes was performed on the combined pediatric and adult SLE dataset with the 21 clusters reported in the original manuscript. Individuals were restricted to reported non-Hispanic Black or White ancestry. A reported SLEDAI value was also required for lupus patients, who were classified as either inactive (SLEDAI < 4) or active (SLEDAI ≥ 4) SLE. This resulted in a final dataset of ~174,000 total PBMCs from 29 individuals (6 White CTRL, 3 White SLE INACT, 2 White SLE ACT, 6 Black CTRL, 4 Black SLE INACT, 8 Black SLE ACT). Immunophenotypes were calculated as percentages of each broad cell type or cluster of total PBMCs. For analysis of gene expression, scRNA-seq count data was obtained from the Gene Expression Omnibus and

processed using the python (version 3.9)-based scanpy(14) (version 1.8.1) generally as described in the methods section of Nehar-Belaid et al(13). Once processed, B cell and monocyte clusters were isolated for independent analysis in R as described above. Cells were filtered for only the individuals matching our ancestry and disease activity criteria. Downstream analysis was carried out by the same methods used on our own dataset. Interferon module scoring was done with Seurat's AddModuleScores function. Within each identified cluster, differential expression of genes between study groups was carried out by MAST. Differentially expressed genes in B cell and monocyte clusters were fed into IPA for analysis of Upstream Regulators and Function Annotations.

SDY40 Intracellular Cytokine Staining. To assess a second cohort for changing in TLR signaling, seven Black controls and 7 White controls were age and sex matched. As described previously (15). Briefly, all subjects from 21-30 years of age were enrolled at Yale Health Services. Immunocompromised subjects were excluded and anyone with a self-reported infection within 2 weeks of enrollment. Human PBMCs were isolated using Ficoll-Hisopaque (Sigma-Aldrich, St. Louis, MO) gradient centrifugation. PBMCs were suspended in RPMI 1640 medium plus 10% FBS and adjusted to a concentration of 2×10^6 cells/ml. The following ligands in medium were added to PBMCs: TLR1/2 ligand (Pam3CSK4, 10 μg/ml), TLR2/6 ligand (lipoteichoic acid [LTA], 2 μg/ml), TLR3 ligand (polyinosinic:polycytidylic acid [poly I:C], 10 μg/ml), TLR5 ligand (flagellin, 5 μg/ml), TLR7/8 ligand (R848 0.5 μg/ml), and TLR9 ligand (CpG-ODN2216, 3 μg/ml). RPMI 1640 medium plus FBS alone was used in control cultures. All ligands were purchased from Invivogen (San Diego, CA). Cells were incubated for 6 h, and brefeldin A (GolgiStop, BD Biosciences) was added for the last 4 h of incubation to retain

intracellular cytokines. Cell suspensions were labeled with primary antibodies (Abs) for surface staining as follows: lineage mixture (CD3 [Clone SK7], CD14 [Clone MphiP9], CD16 [3G8], and CD19 [SJ25C1]-APC-Cy7, HLA-DR–PE-Cy7, CD11c [B-ly6]-APC, and CD123 [9F5]–PE-Cy5). For analysis for CD80/86 molecules, antibodies against CD80 [2D10.4]-FITC and CD86 [IT2.2]-PE were used. All Abs were purchased from BD Biosciences, except for anti–CD11c and anti-CD86 antibodies, which were from BD Biosciences PharMingen.

On the day of analysis, cells were thawed, permeabilized in BD Perm/Wash buffer (BD Pharmingen, San Diego, CA), pelleted at 500 × *g* for 10 min at 4°C, and resuspended in BD Perm/Wash buffer containing Abs for intracellular staining of cytokines or intracellular TLRs as follows: IL-6–PE (Clone MQ2-6A3), TNF-α Alexa 700 (Clone MAb11), IL-12 (p40)-Pacific Blue (Clone C8.6), IFN-α–FITC (Clone 225.2C), with appropriate isotype controls. A biotinylated isotype control plus streptavidin-FITC was used for all experiments employing biotinylated Abs. IL-6, IL-12, and TNF-α Abs were purchased from BD Pharmingen. Abs to IFN-α were purchased from Chromaprobe (Maryland Heights, MO). Approximately 0.5–1 million total events were collected per sample using an LSR II instrument (BD Biosciences), with analysis using Cytobank.

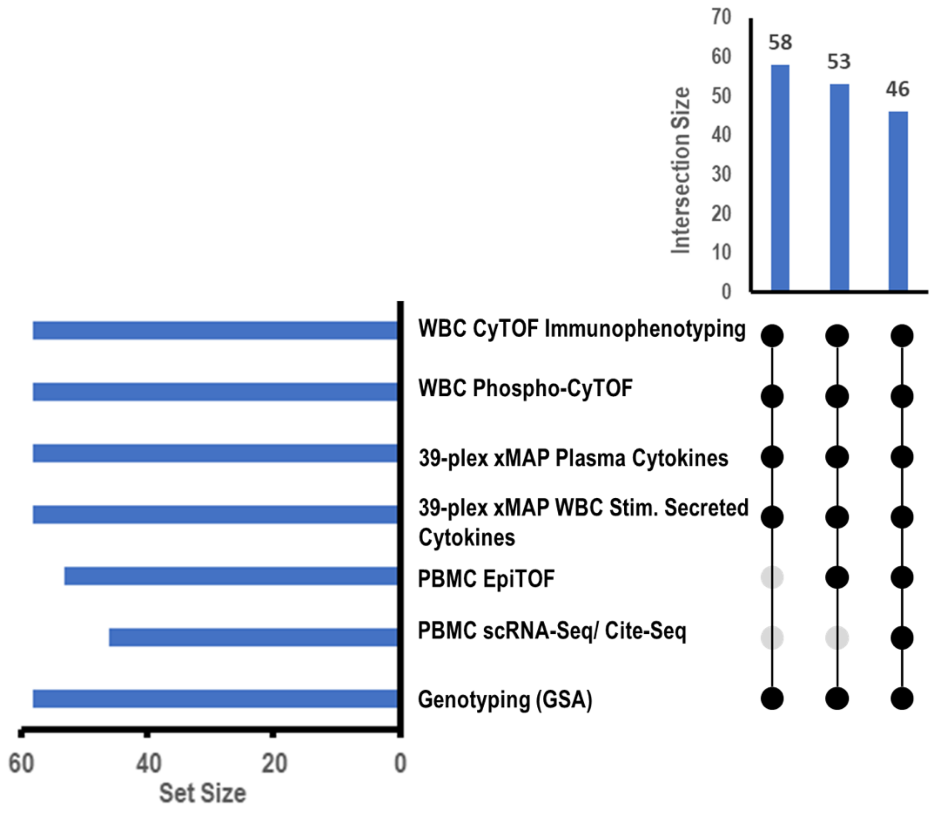

Supplemental Figure 1. Systems overview of overlapping samples. A total of 58 subjects including White and Black controls, SLE INACT, and SLE ACT patients were used as the primary study subset for this study. The majority of systems analyses included all 58 subjects. The set size is indicated on the left for each data platform and the intersection size of subjects across datasets is indicated on the top. Additional public datasets were used for a replication set of scRNA-sequencing (GSE135779) and intracellular signaling set on IMMPORT (SDY40). Further, a second CyTOF dataset was combined with the immunophenotyping data of this 58-sample set as described in the text.

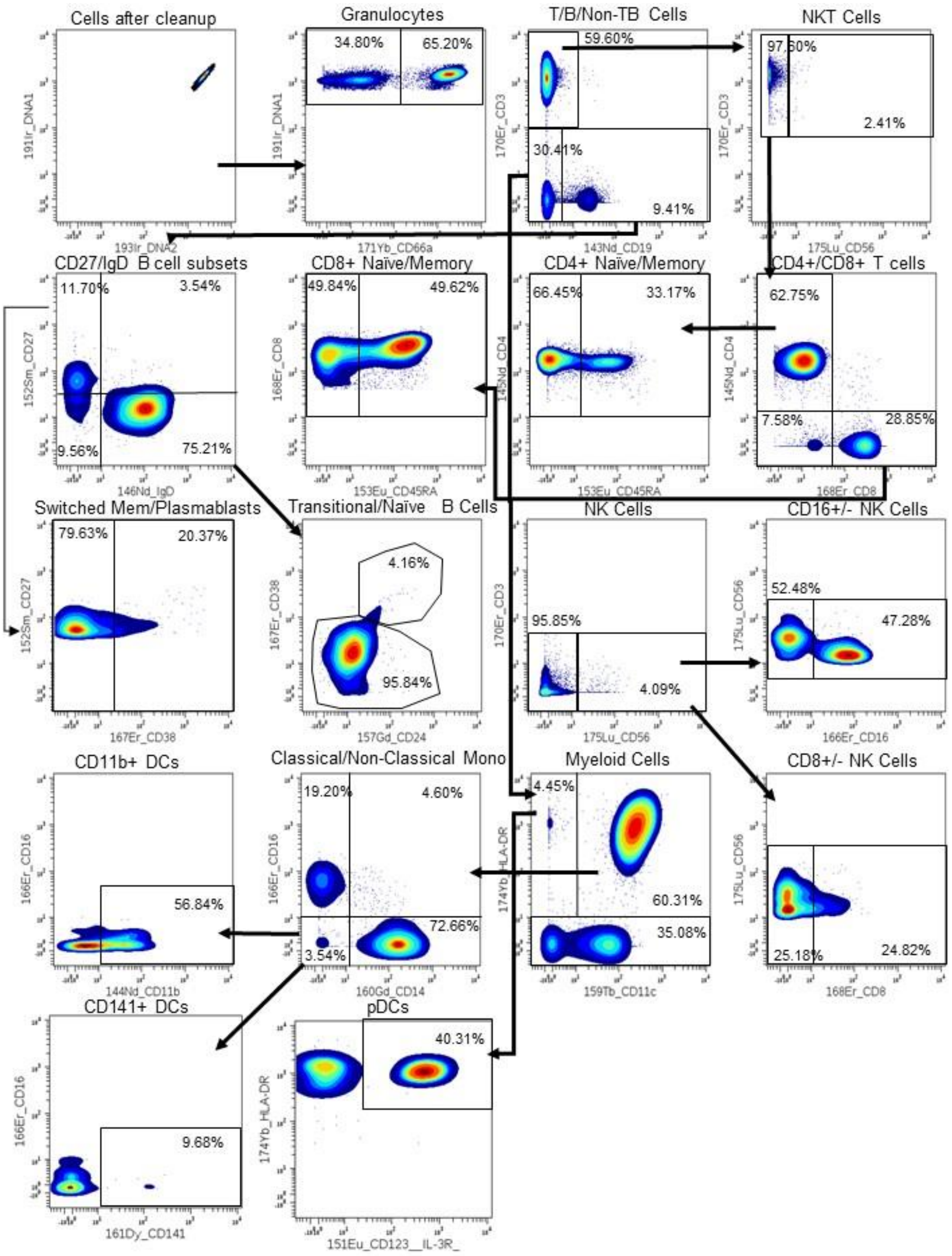

Supplemental Figure 2. Representative gating hierarchies for mass cytometry phenotyping analysis. Bivariate gating hierarchy was used to confirm and access tSNE results of whole blood phenotyping from 58 subjects. Cells went through an initial cleanup strategy following the manufacturer recommended cleanup gates to remove aggregates, debris, beads, and doublets as found in the Fluidigm Maxpar manual. In addition, unsupervised clustering and cell identification were done via tSNE as shown in Supplemental Figure 3.

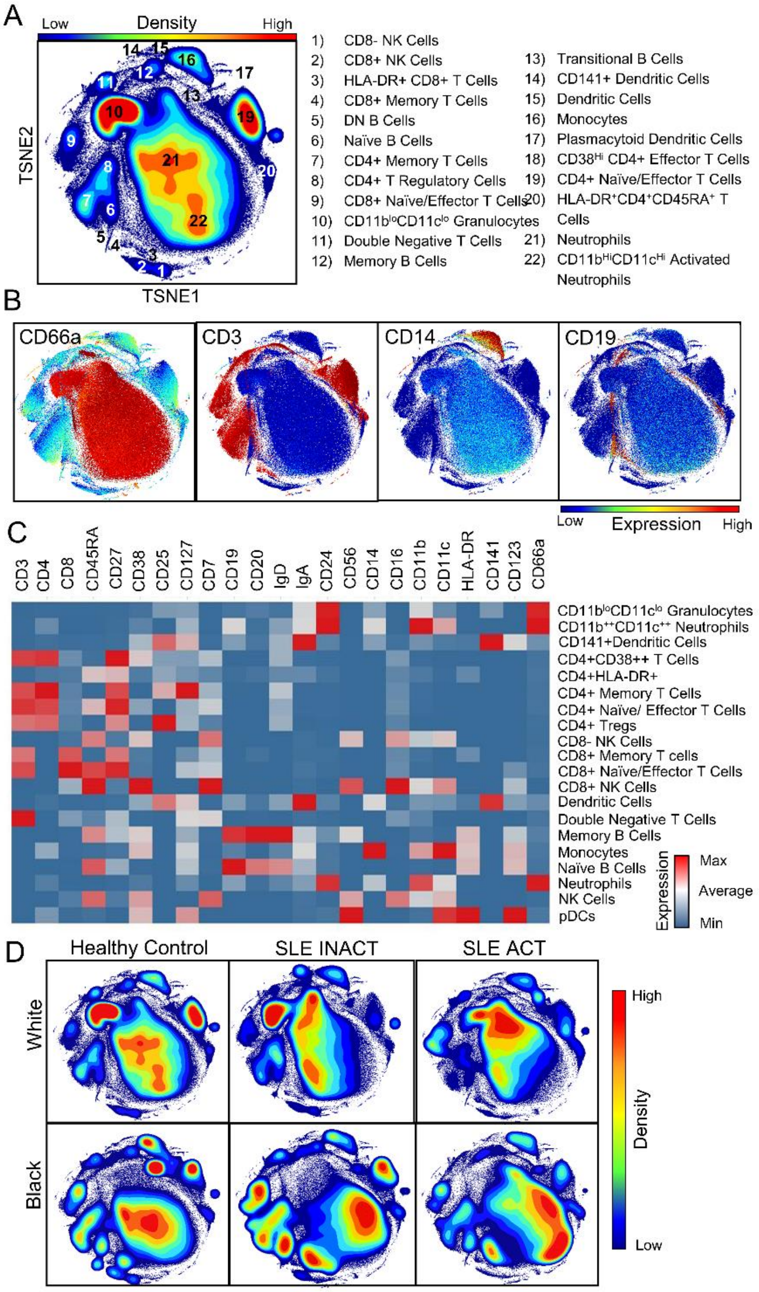

Supplemental Figure 3. Whole blood tSNE plots in White and Black patients. Mass cytometry data was visualized using the Cytobank platform. (A) Using dimensionality reduced t-SNE plots from PBMC data (1.3 million cells) derived from 58 samples. tSNE analyses identified 22 distinct cell populations. Cell surface expression of major lineage markers was used to name cell subsets via (B) tSNE marker expression plots with CD66a, CD3, CD14, and CD19 shown and (C) a heatmap summary of the average expression values of all 23 surface markers assessed. Marker values are displayed on a color scale ranging from blue (levels below the mean) through White (levels equal to the mean) to red (levels greater than the mean) using a column Z-score. (D) Density maps depicting White and Black healthy controls, SLE INACT, and SLE ACT patients whole blood t-SNE plots were created using all 23 surface markers. All plots were derived from cumulative data from 8-11 individuals per group.

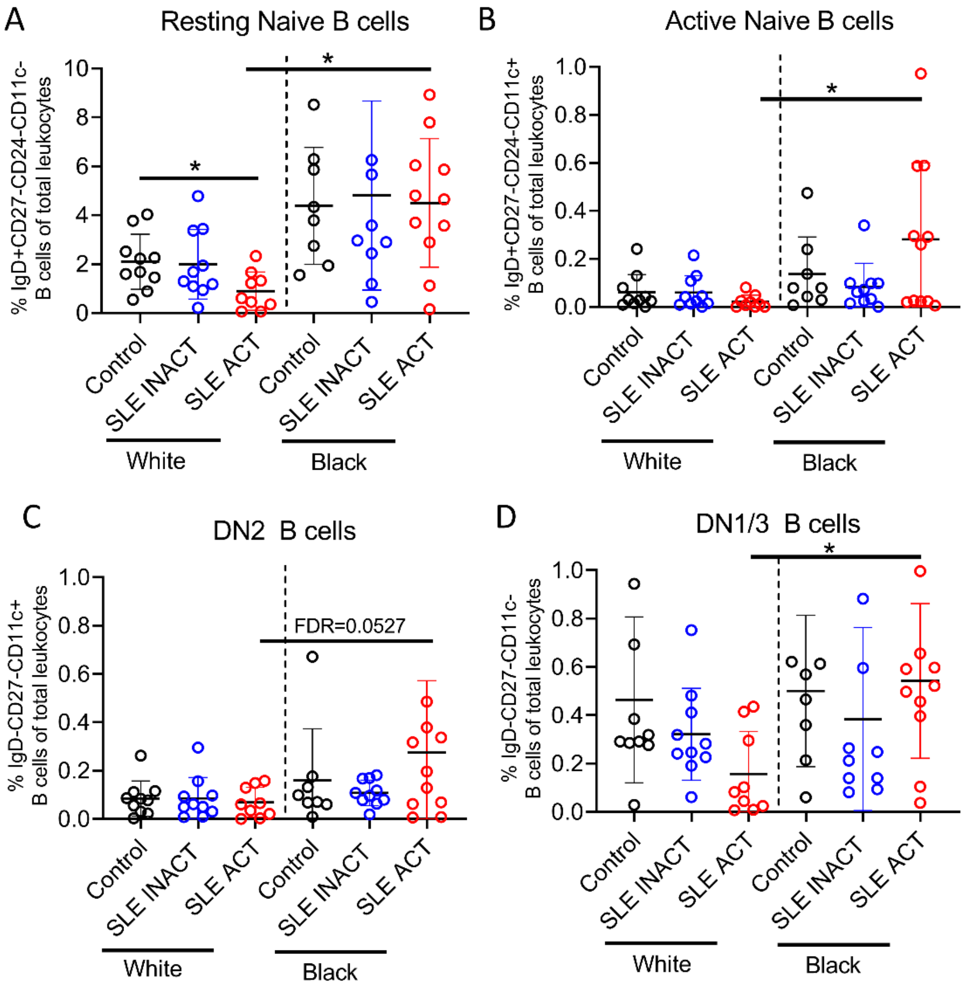

Supplemental Figure 4. Naïve and DN B cell subset frequencies in Black and White SLE ACT patients. All cell subsets were manually gated and back calculated for total frequency of leukocytes. B cell frequencies on White and Black control, SLE INACT, and SLE ACT patient are shown for (A) IgD+CD27-CD24-CD11c- resting naïve B cells, (B) IgD+CD27-CD24-CD11c+ active naïve B cells, (C) IgD-CD27-CD11c+ DN2 B cells, and (D) IgD-CD27-CD11c- DN1/3 B cells. Statistical significance was determined using a Mann-Whitney U test and FDR *p<0.05, Mean± SD shown. (n=58)

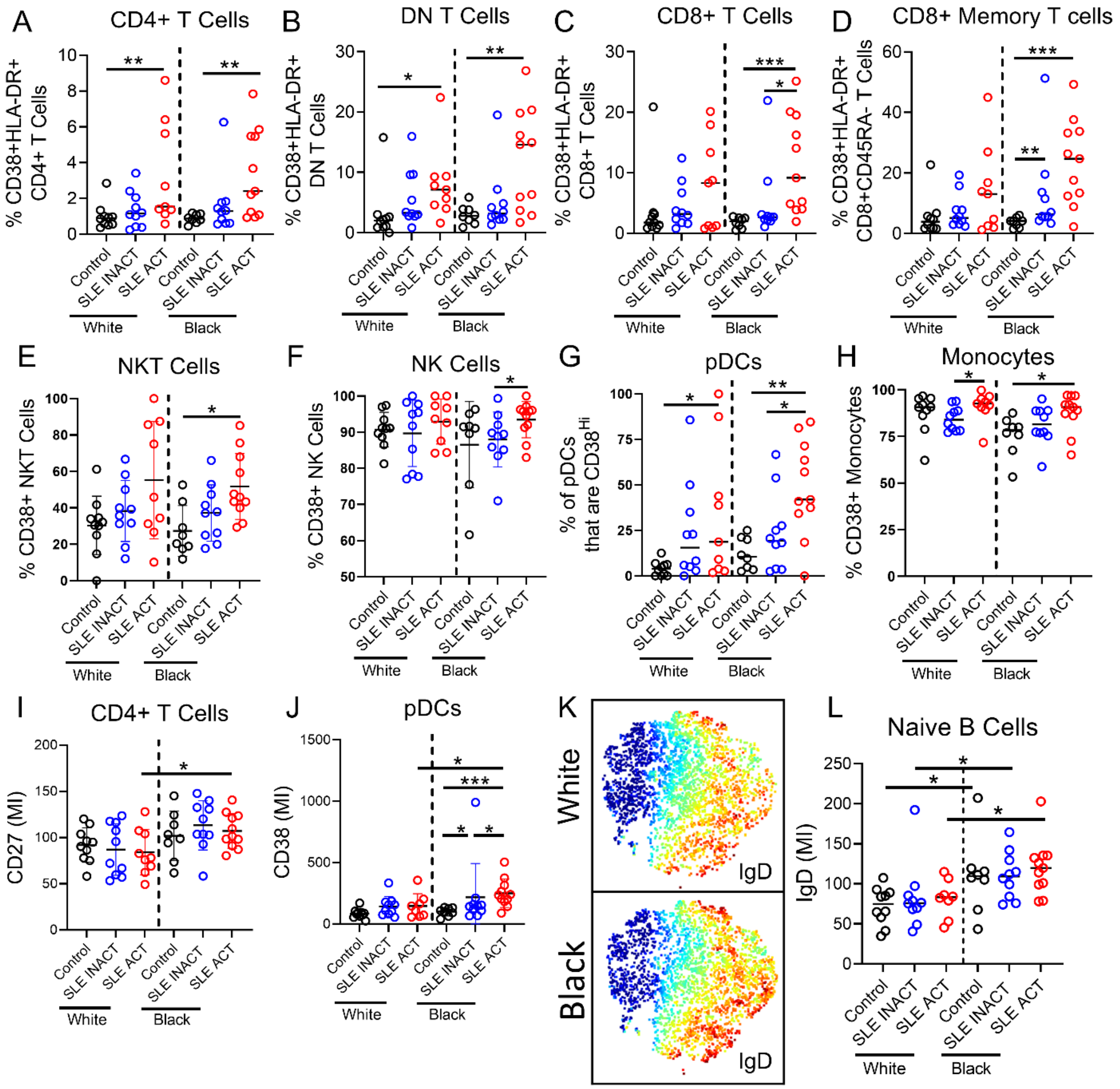

Supplemental Figure 5. Cell activation in Black and White SLE ACT patients. All cell subsets were manually gated and assessed for frequencies and differences in metal intensity (MI) of activation and regulatory surface markers (A-D) CD38+HLA-DR+ frequencies on White and Black control, SLE INACT, and SLE ACT patient (A) CD4+ T cells, (B) DN T cells, (C) CD8+ T cells, and (D) CD8+ memory T cells. CD38 frequencies on (E) NKT cells, (F) NK cells, (G) pDCs, and (H) monocytes, (I) CD27 metal intensity on CD4+ T cells, and (J) CD38 metal intensity on pDCs is shown by disease. t-SNE plots were used to illustrate specific expression differences among B cells showing (K) IgD expression and (L) IgD metal intensity in naïve B cells. tSNE plots use red to indicate elevated expression and blue to indicate low expression. *p<0.05, **p<0.01, ***p<0.001, by Kruskal-Wallis test with two-tailed Mann-Whitney for multiple comparisons. Mean± SD shown. (n=58)

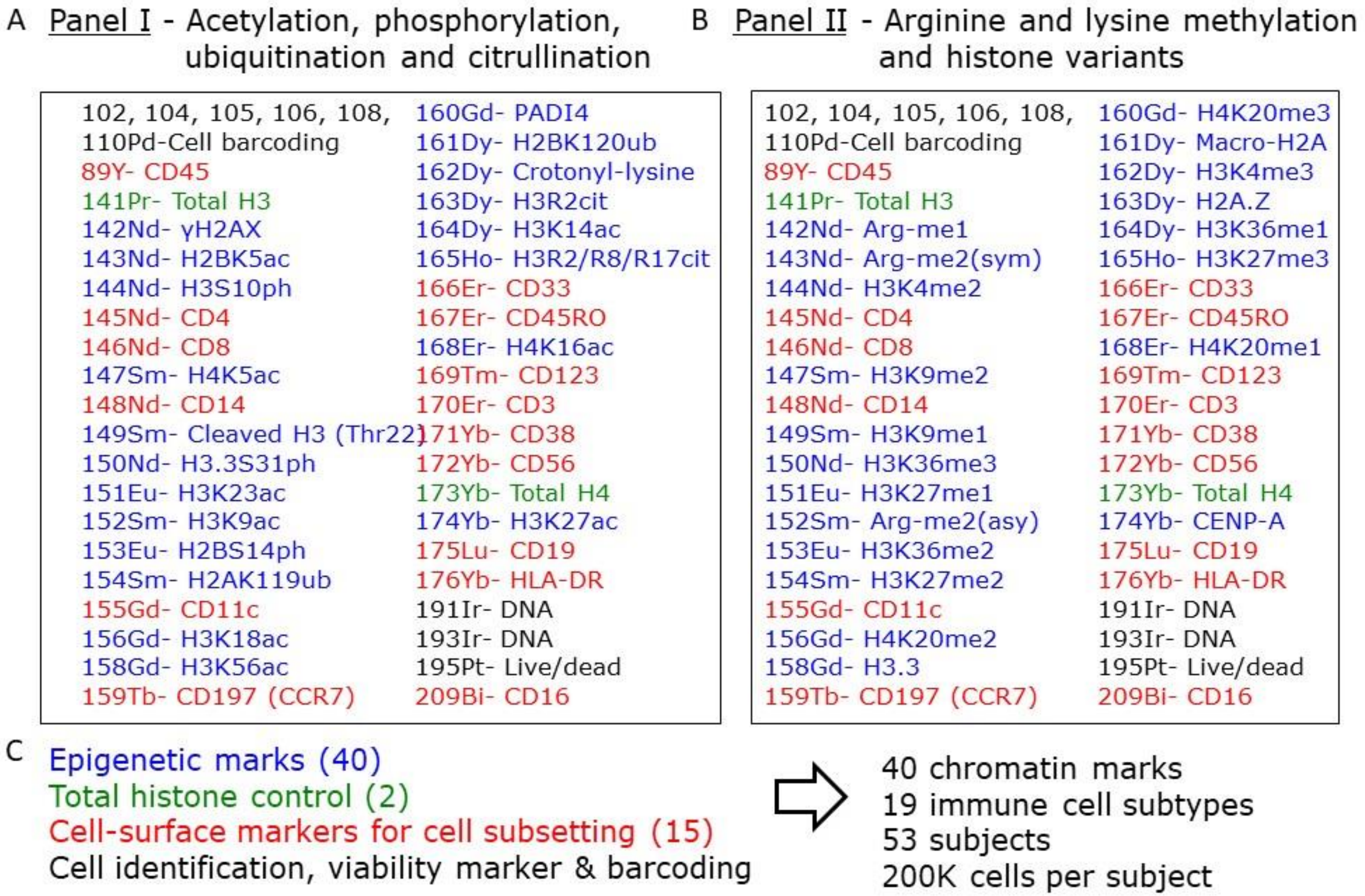

Supplemental Figure 6. EpiTOF antibody panel. Two panels (A) and (B) were used to assess 40 different chromatin marks to observed differences in acetylation, phosphorylation, ubiquitination, citrullination, arginine and lysine methylation, and histone variants across White and Black controls, SLE INACT, and SLE ACT patients. (C) This panel allowed us to assess 40 chromatin marks and 19 immune cell subsets using 200,000 cells/subject.

A

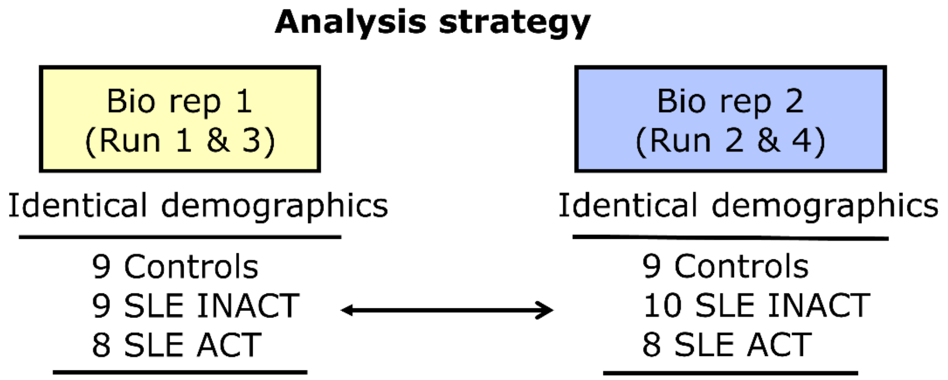

B

Run 1

Run	Study Group	Ancestry	SLEDAI	Cell Viability (%)
1	Control	White	NA	93
1	Control	White	NA	92
1	Control	White	NA	84
1	Control	White	NA	81
1	Control	White	NA	85
1	SLE INACT	White	0	95
1	SLE INACT	White	2	92
1	SLE INACT	White	2	87
1	SLE INACT	White	0	78
1	SLE INACT	White	2	90
1	SLE ACT	White	6	87
1	SLE ACT	White	6	74
1	SLE ACT	White	6	85
1	SLE ACT	White	10	74

Run 2

Run	Study Group	Ancestry	SLEDAI	Cell Viability (%)
2	Control	White	NA	84
2	Control	White	NA	89
2	Control	White	NA	86
2	Control	White	NA	92
2	Control	White	NA	94
2	SLE INACT	White	0	85
2	SLE INACT	White	2	85
2	SLE INACT	White	0	84
2	SLE INACT	White	3	86
2	SLE INACT	White	2	89
2	SLE ACT	White	6	85
2	SLE ACT	White	4	83
2	SLE ACT	White	6	86
2	SLE ACT	White	6	89

Run 3

Run	Study Group	Ancestry	SLEDAI	Cell Viability (%)
3	Control	Black	NA	85
3	Control	Black	NA	91
3	Control	Black	NA	89
3	Control	Black	NA	84
3	SLE INACT	Black	2	84
3	SLE INACT	Black	2	87
3	SLE INACT	Black	2	89
3	SLE INACT	Black	0	92
3	SLE ACT	Black	8	93
3	SLE ACT	Black	8	92
3	SLE ACT	Black	8	90
3	SLE ACT	Black	6	77

Run 4

Run	Study Group	Ancestry	SLEDAI	Cell Viability (%)
4	Control	Black	NA	95
4	Control	Black	NA	87
4	Control	Black	NA	93
4	Control	Black	NA	62
4	SLE INACT	Black	0	94
4	SLE INACT	Black	2	78
4	SLE INACT	Black	2	90
4	SLE INACT	Black	0	90
4	SLE INACT	Black	2	89
4	SLE ACT	Black	8	90
4	SLE ACT	Black	7	85
4	SLE ACT	Black	4	88
4	SLE ACT	Black	11	56

Supplemental Figure 7. Sample bioreplicates used for EpiTOF analyses. 53 matched subjects were divided into two biological replicates with identical demographics that were evenly distributed between Black (Black) and White (White) Americans.

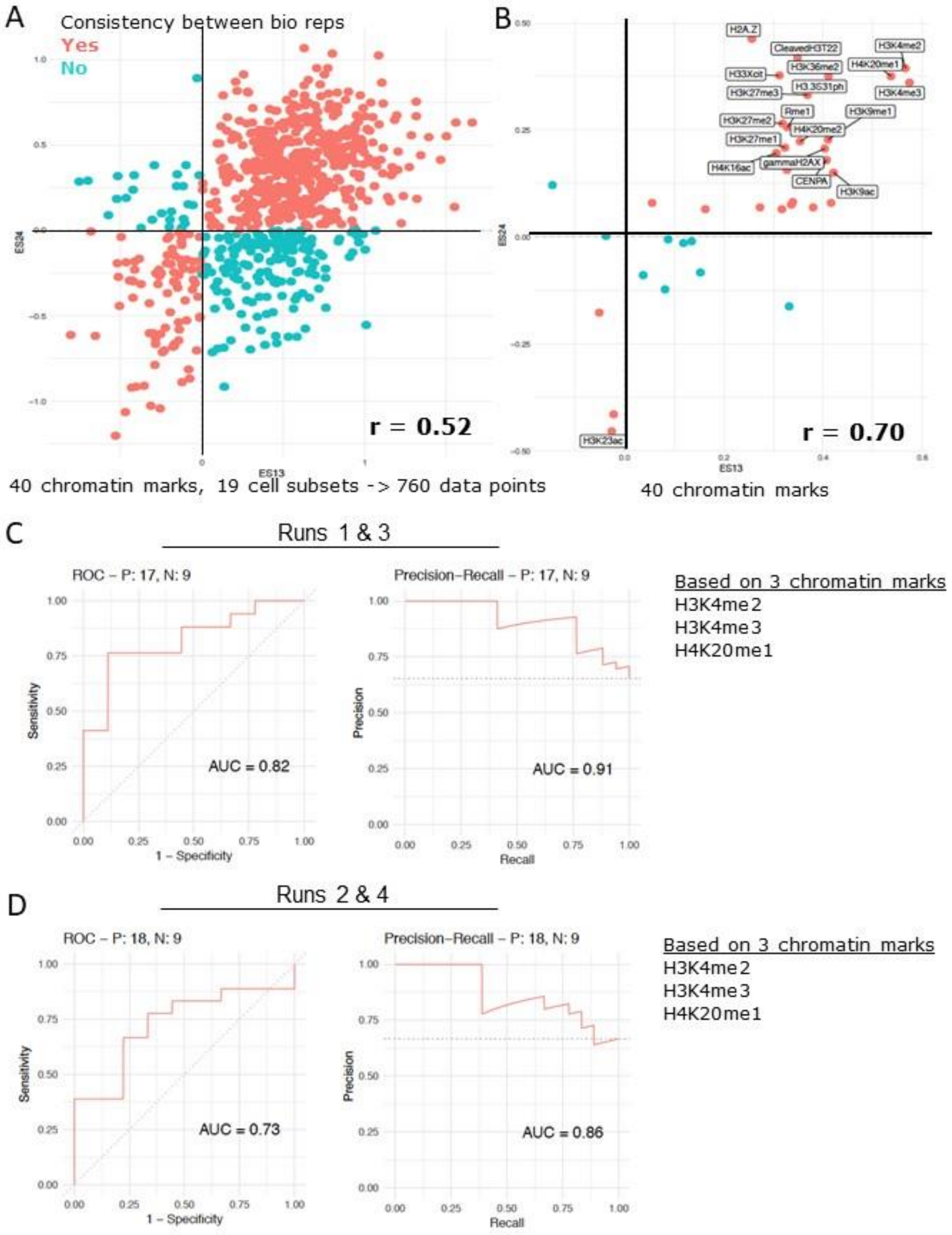

Supplemental Figure 8. Chromatin marks significantly differentiate SLE patients from controls. PBMCs were used to assess global differences in 40 different chromatin modifications in 19 immune cell subsets. (A) Consistency between bioreplicates in differentially expressed chromatin marks between SLE and controls. (B) Effect size comparison where each dot represents the average of a chromatin mark across all immune cell subtypes. Correlation coefficient is shown. (C) ROC (left) and precision recall (right) curves depict the segregation of controls from SLE patients using the variance in 3 chromatin marks. Curves summarizing the results from biological replicates in (C) runs 1 and 3 and (D) runs 2 and 4, with AUC percentages from independent replicates separately listed. Classification specificity (x-axis) and sensitivity (y-axis) are shown for ROC curve (left). Precision (y-axis) and recall (x-axis) rates are shown for precision recall curve (right).

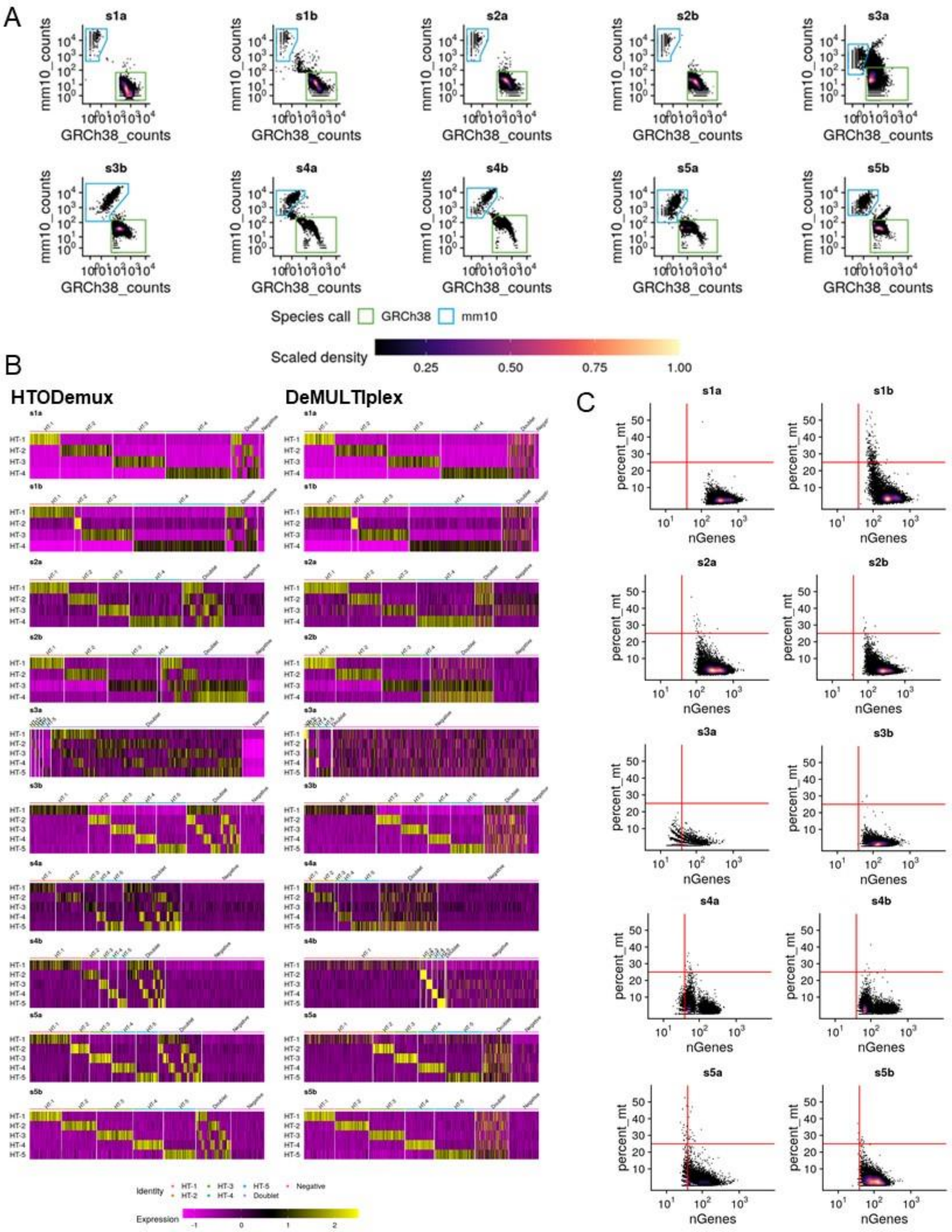

Supplemental Figure 9. Preprocessing and QC of single cell RNA-sequencing data. After analysis via the cell ranger pipeline, feature count matrices for each batch were fed into a custom R pipeline for preprocessing and quality control. (A) Biaxial plots of total counts aligned to the mouse (mm10_counts) and human (GRCh38_counts) genomes calculated for each barcode in each batch. The scaled density of barcodes on these plots is represented by a pseudocolor gradient. Human and mouse cells were separated by the manually drawn gates in green and blue, respectively. (B) Heatmaps of scaled counts of hashtag oligos of human cells in each batch for identities called by two different algorithms: HTODemux and DeMULTIplex. Barcodes called for each hashtag identity, doublets, and negatives are grouped together. (C) Biaxial plots of number of genes (nGenes) and percent mitochondrial counts (percent_mt) for singlet barcodes in each batch. Scaled density of barcodes is represented by pseudocolor gradient as in (A). Global cutoffs are indicated as red lines at nGenes = 40 and percent_mt = 25.

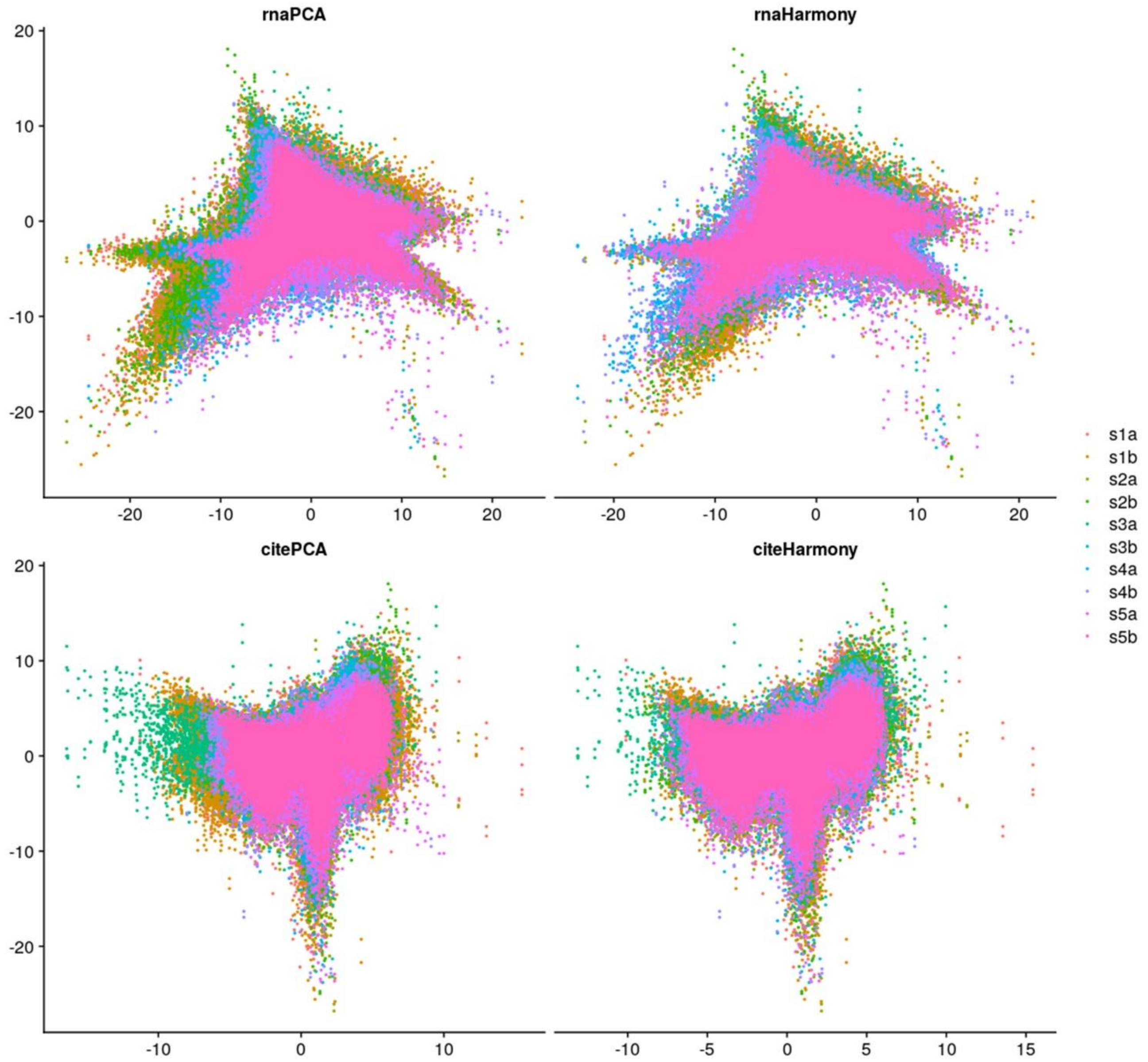

Supplemental Figure 10. Harmony corrects for batch effects in scRNA-sequencing on the principal component reductions of each modality. Plots of the first two components of principal component analysis with (right) and without (left) correction by Harmony. This was applied to both feature barcode matrices of (A) gene expression (rnaPCA and rnaHarmony) and (B) protein expression (citePCA and citeHarmony).

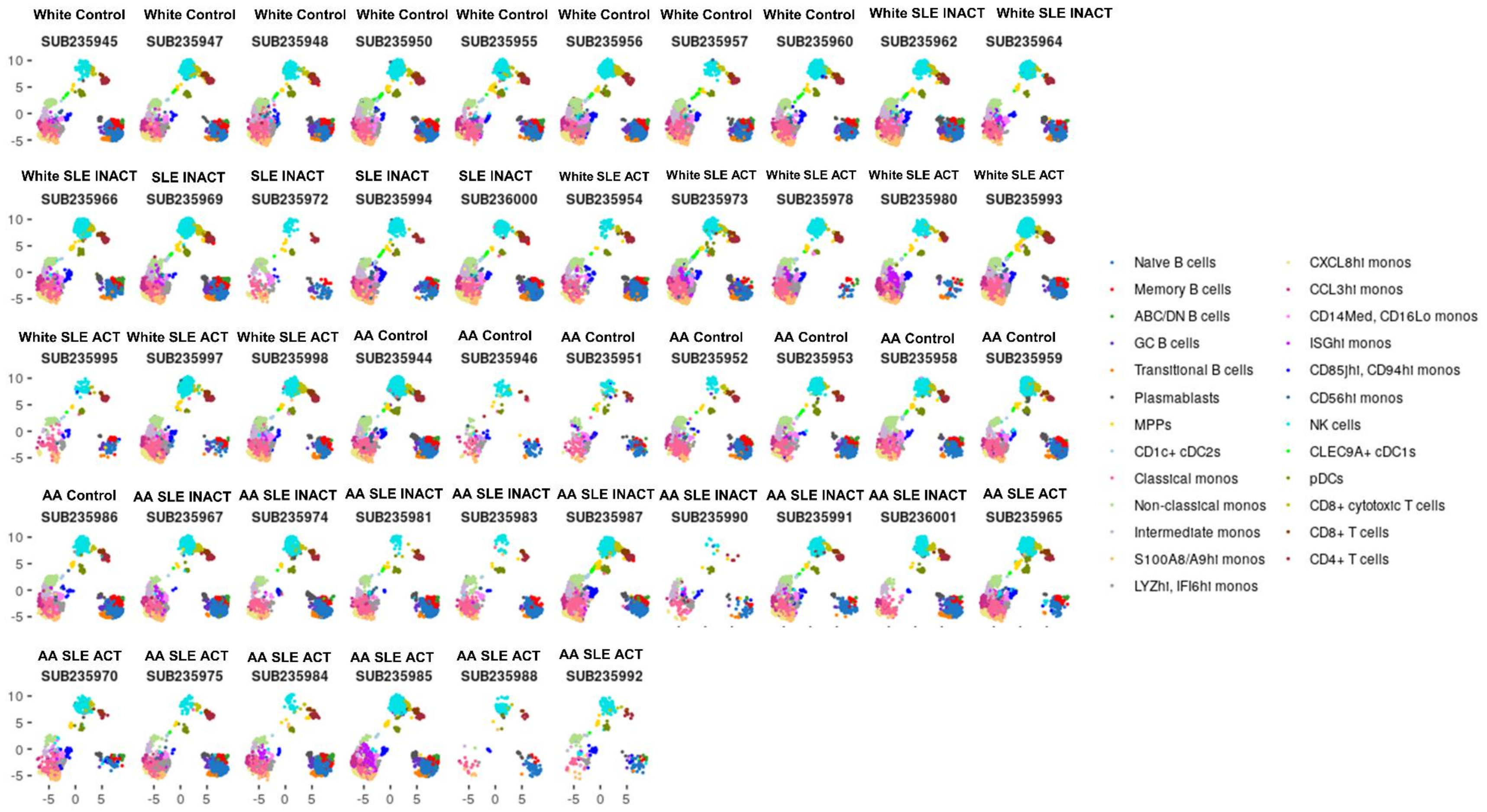

Supplemental Figure 11. Cell subsets identified in multimodal analysis are represented across all patients. UMAP plots of single cells from all 48 patients included in the analysis. Colors represent adjudicated cell types.

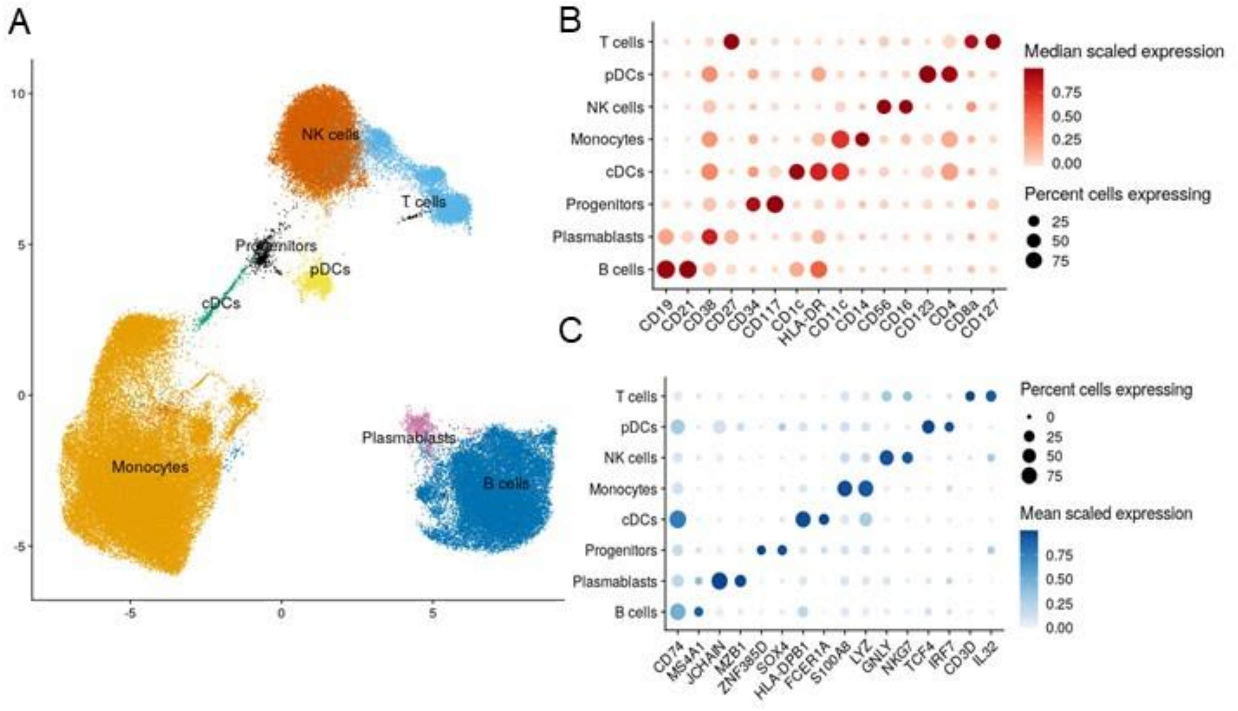

Supplemental Figure 12. Analysis of total single cell dataset identifies major canonical cell types. After manual QC, the final multimodal dataset of 88,053 cells was analyzed. (A) Two-dimensional uniform manifold approximation and projection (UMAP) based on a weighted nearest neighbors analysis of gene and protein expression data. Broad cell types are labeled and colored on this representation. (B) Dot plot of scaled expression of top protein markers used to manually annotate cells. For protein expression, the color gradient of the dot represents the median expression of the indicated protein, while the size of the dot indicates the percent of cells in each cluster expressing the protein above a baseline threshold. (C) Dot plot of scaled expression of top gene markers used to manually annotate cells. For gene expression, the dot gradient of the dot indicates the mean expression of a gene, while the size of the dot indicates the percent of cells in each cluster detectably expressing the gene.

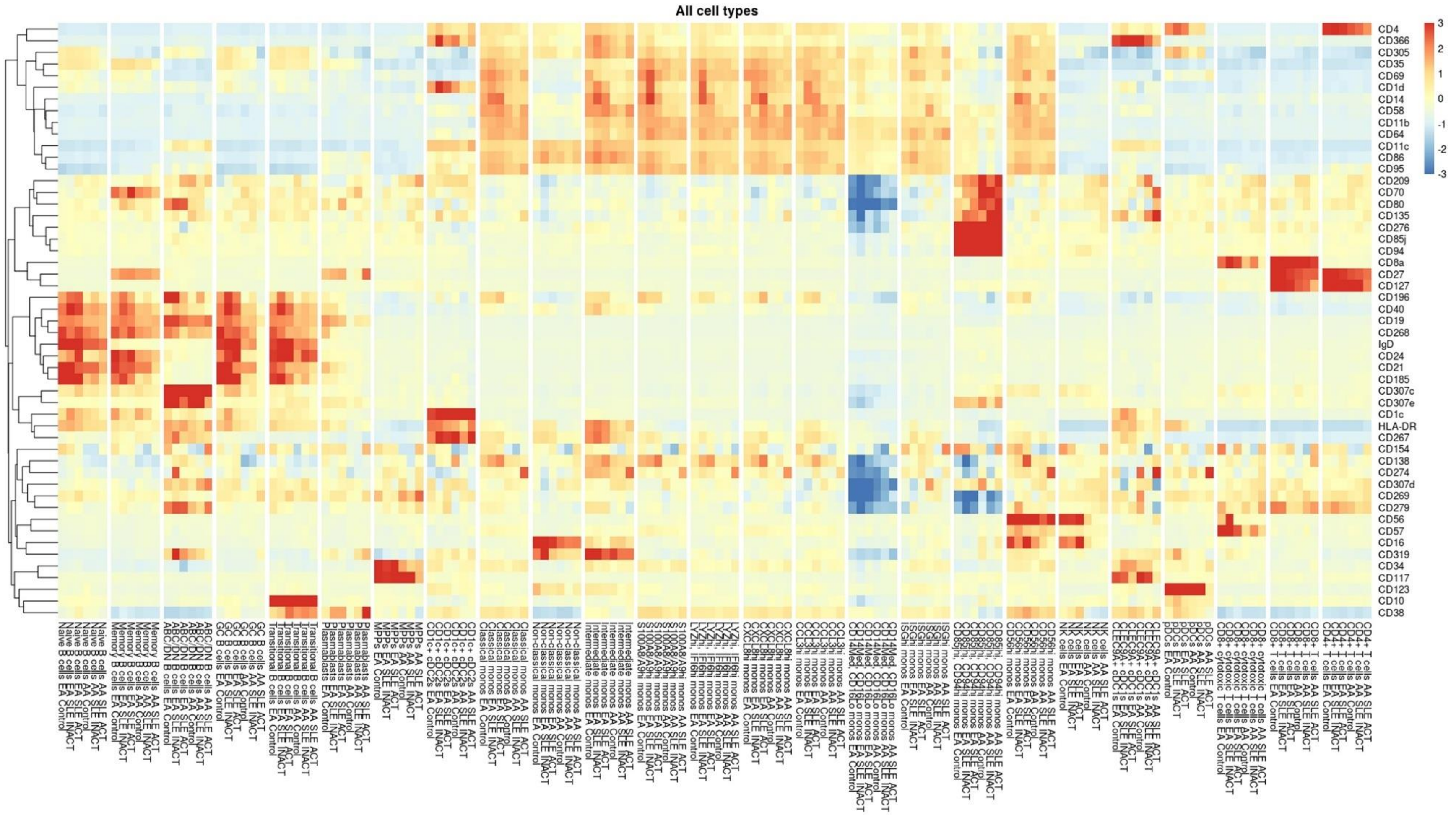

Supplemental Figure 13. Global landscape of protein expression by CITEseq shows epitope variation by ancestry and disease status. Heatmap of scaled median expression (z-scores) of all proteins assayed by the CITEseq panel. Each identified cell subset is divided into six study groups by ancestry and disease status.

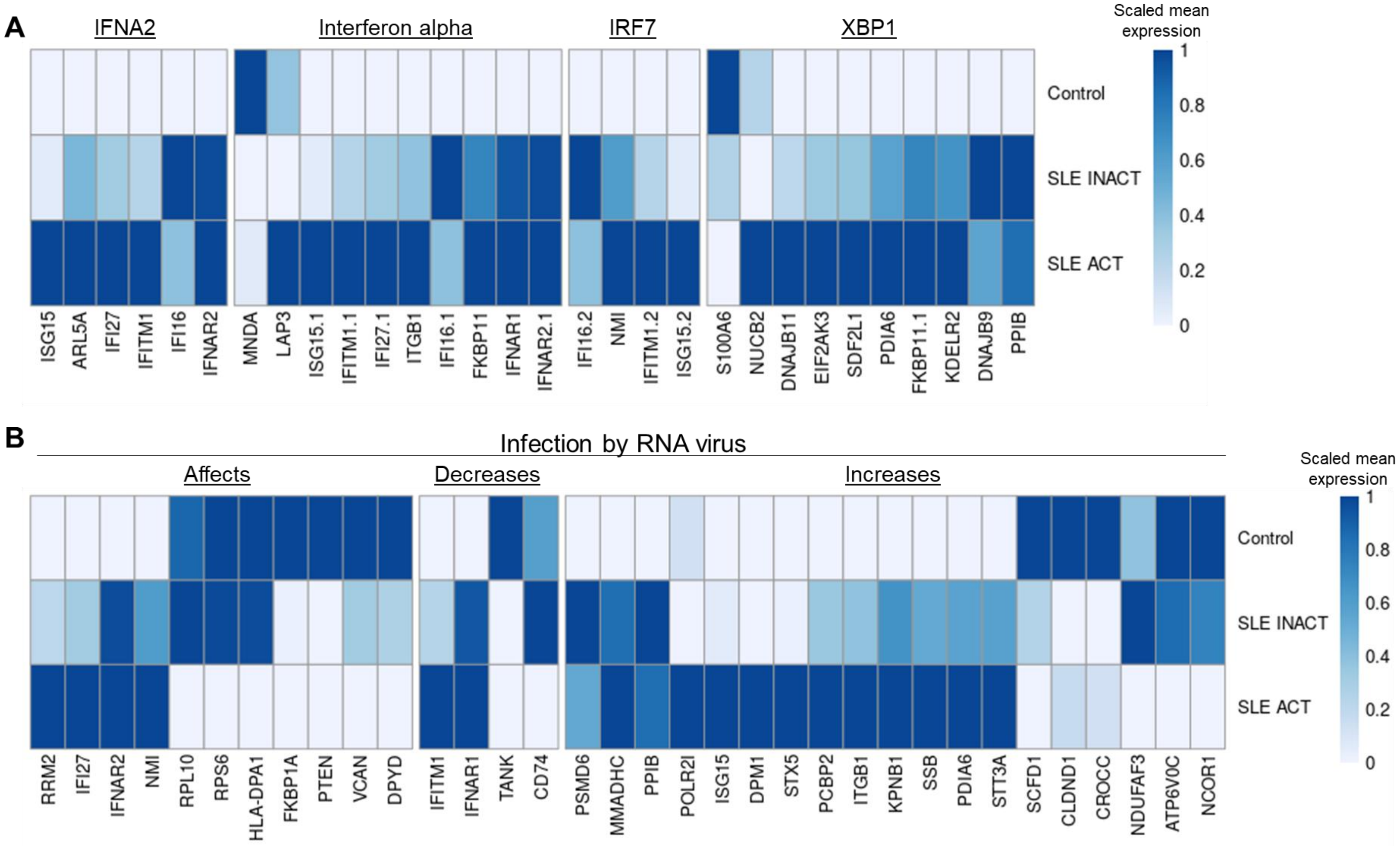

Supplemental Figure 14. Ingenuity Pathway Analysis of plasmablast DEGs predicts activation of interferon-associated upstream regulators and RNA viral infection pathways in SLE ACT patients. Differentially expressed genes in plasma cells comparing SLE ACT patients against controls were input to Ingenuity Pathway Analysis (IPA). IPA predicted differences in (A) upstream activation of type I IFN-associated factors IFNA2, XBP1, and IRF7, as well as (B) the RNA viral infection pathway from biological function analysis. Genes in RNA infection pathway are divided by pathway contribution based on IPA literature findings.

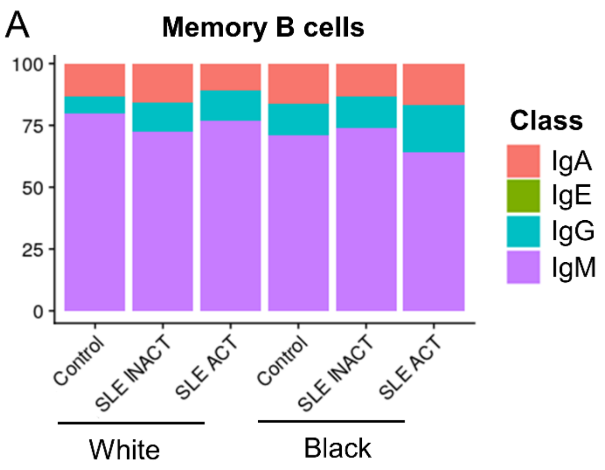

Supplemental Figure 15. Single cell transcriptomics reveal greater frequency of IgG in memory B cells of Black SLE patients. Percentage of memory B cells with highest gene expression of class-switched IgA, IgE, IgG, or IgM.

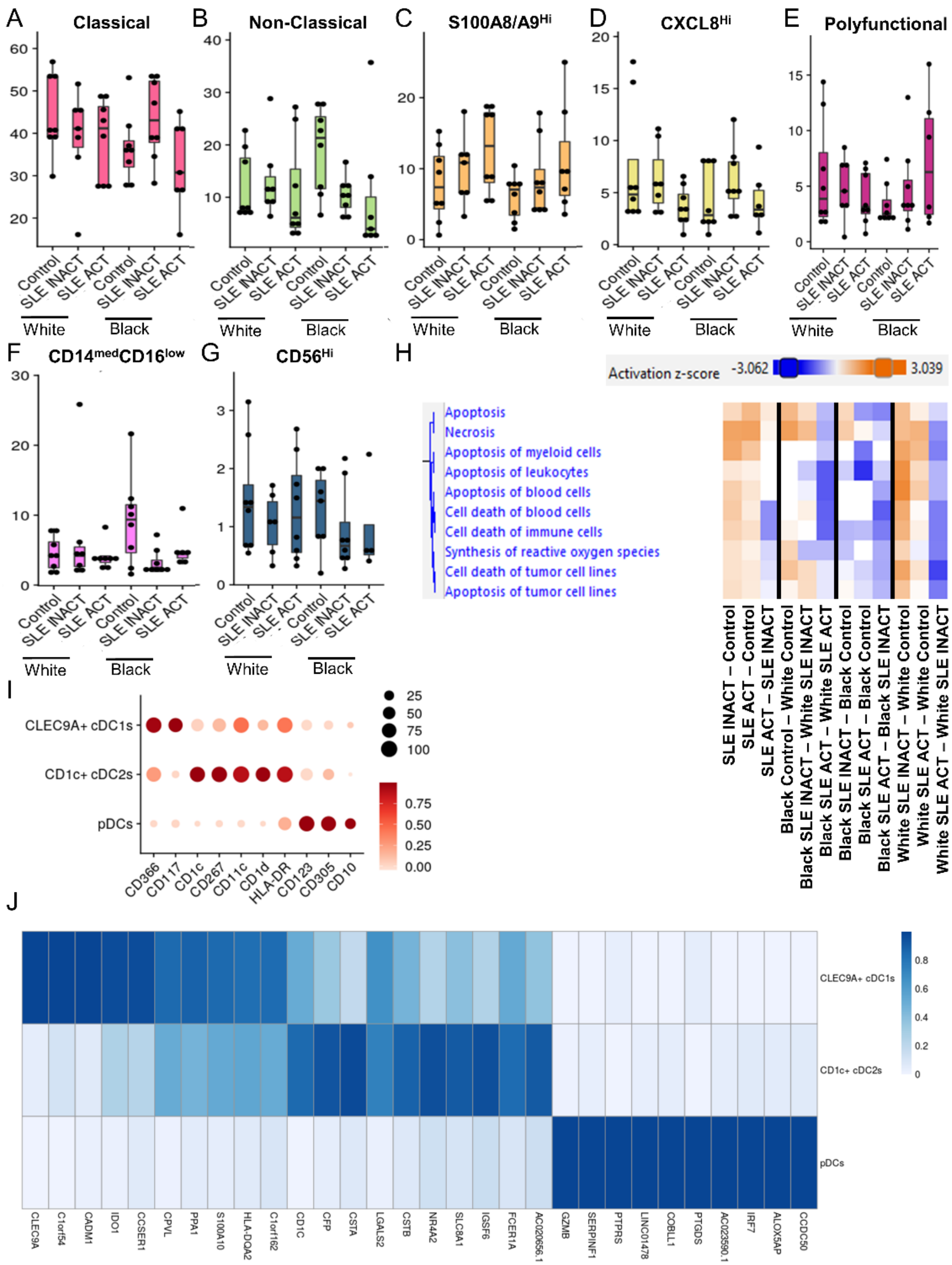

Supplemental Figure 16. Single cell multi-omic analysis of myeloid cells identifies additional monocyte and dendritic cell subsets. Violin plots comparing the percentage of total monocytes across disease groups for (A) classical, (B) non-classical, (C) S100A8/9^{Hi}, (D) CXCL8^{Hi}, (E) CCL3^{Hi}, (F) CD14^{Med}CD16^{Low}, and (G) CD56^{Hi} monocytes. (H) Heatmap of z-scores indicating predicted activation or inactivation of apoptosis functional pathways by Ingenuity Pathway Analysis of intermediate monocytes for all disease group comparisons. (I) Dot plot of scaled expression of top protein markers used to annotate subsets of dendritic cells. (J) Heatmap of scaled expression of top gene markers used to annotate subsets of dendritic cells.

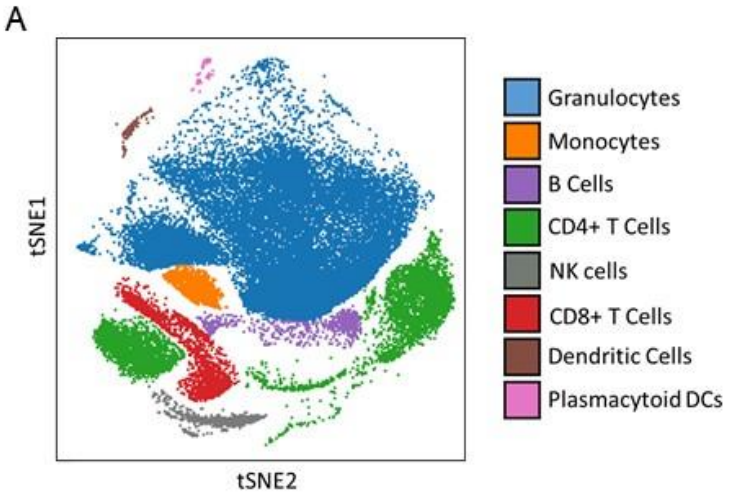

Supplemental Figure 17. Phospho-CyTOF cell subset identification by tSNE. Eight different immune cell populations were identified using the 23 surface markers in the phospho-CyTOF panel. Cell subsets are colored for reference to give spatial orientation for Supplemental Figures 21-28.

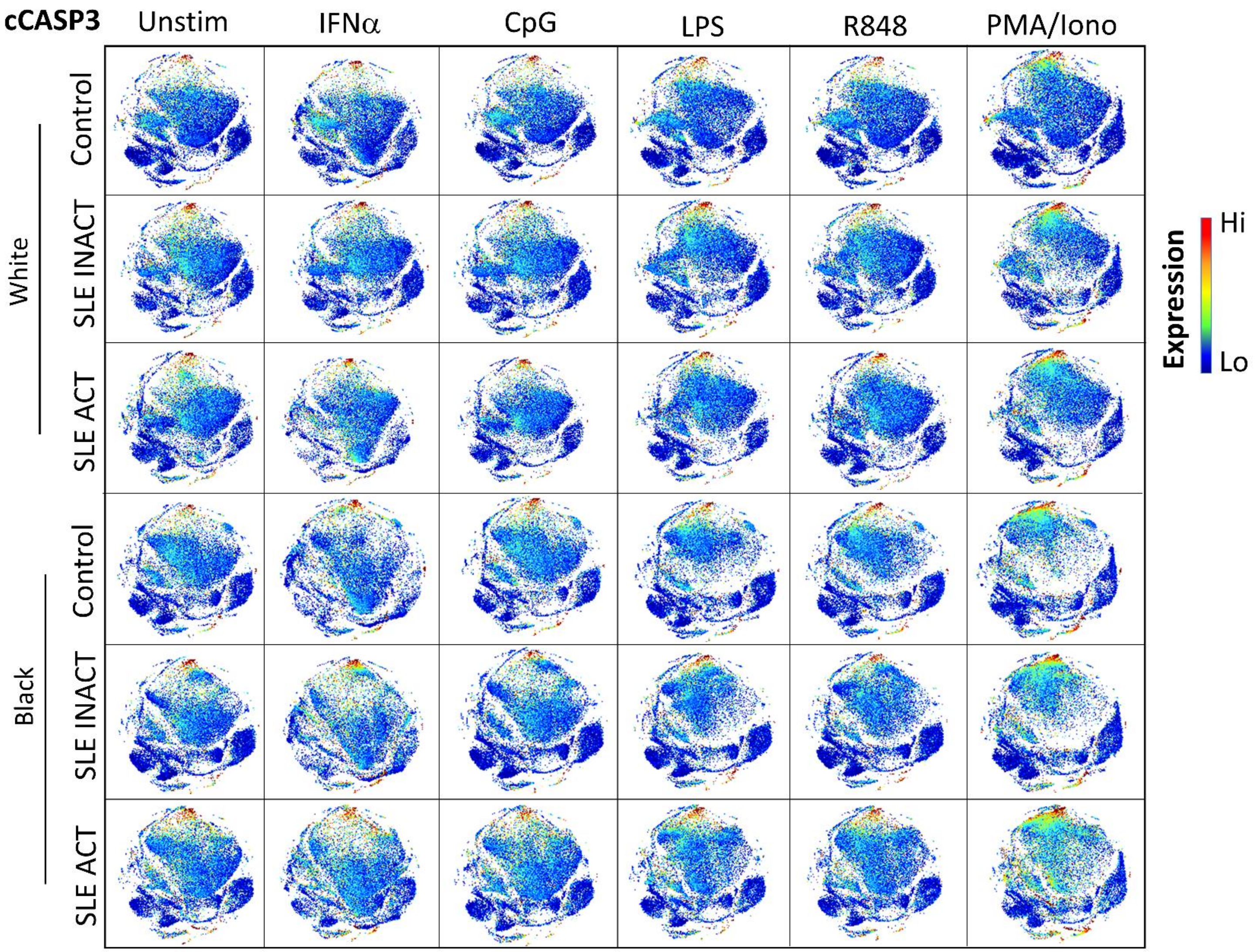

Supplemental Figure 18. cCASP3 expression in cells by disease type. Concatenated files (430,000 cells/each) were used for each ancestry and disease group to visualize expression and changes in phospho-signaling markers with stimulation. High expression is indicated in red, medium in yellow, and low expression in blue.

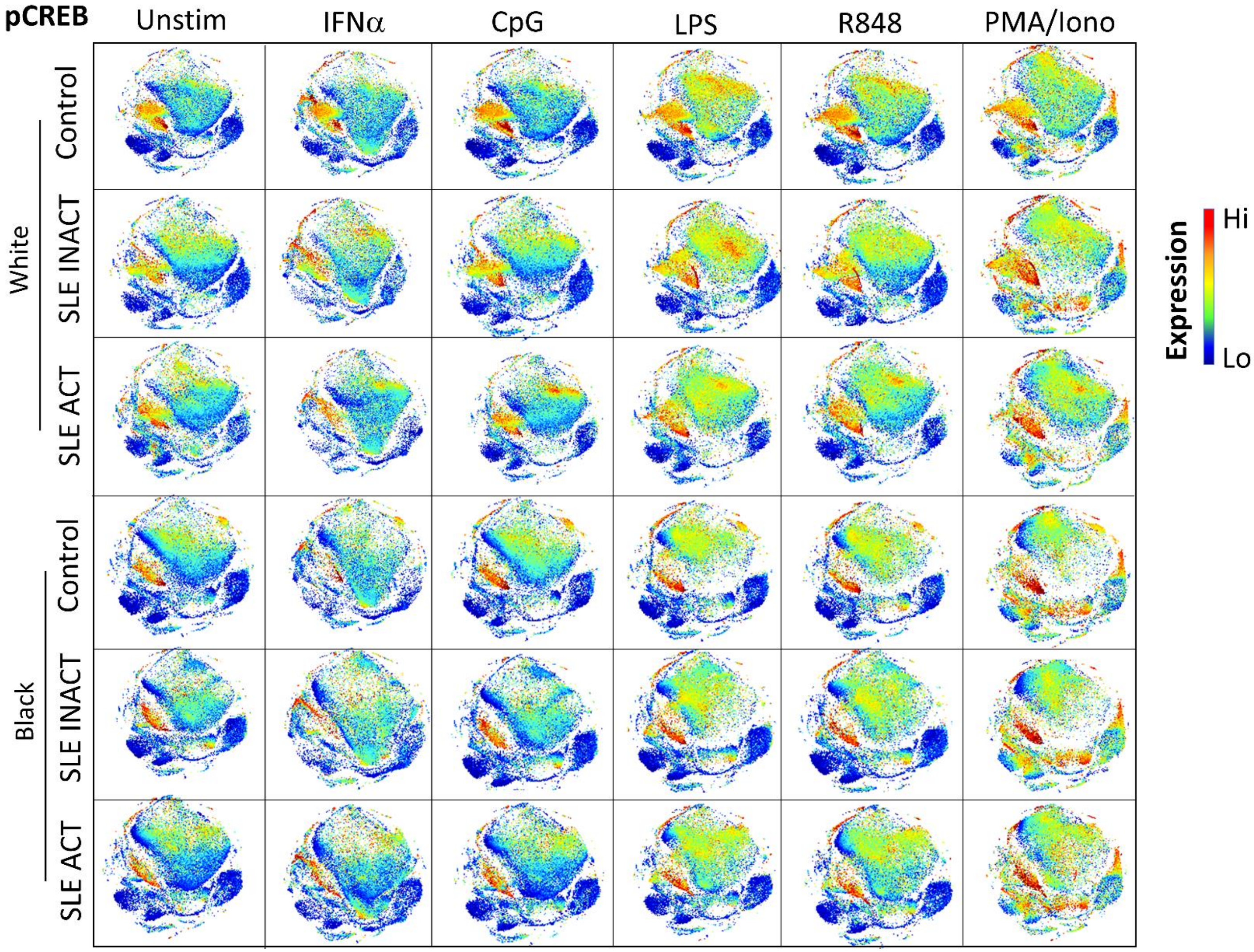

Supplemental Figure 19. pCREB expression in cells by disease type. Concatenated files (430,000 cells/each) were used for each ancestry and disease group to visualize expression and changes in phospho-signaling markers with stimulation. High expression is indicated in red, medium in yellow, and low expression in blue.

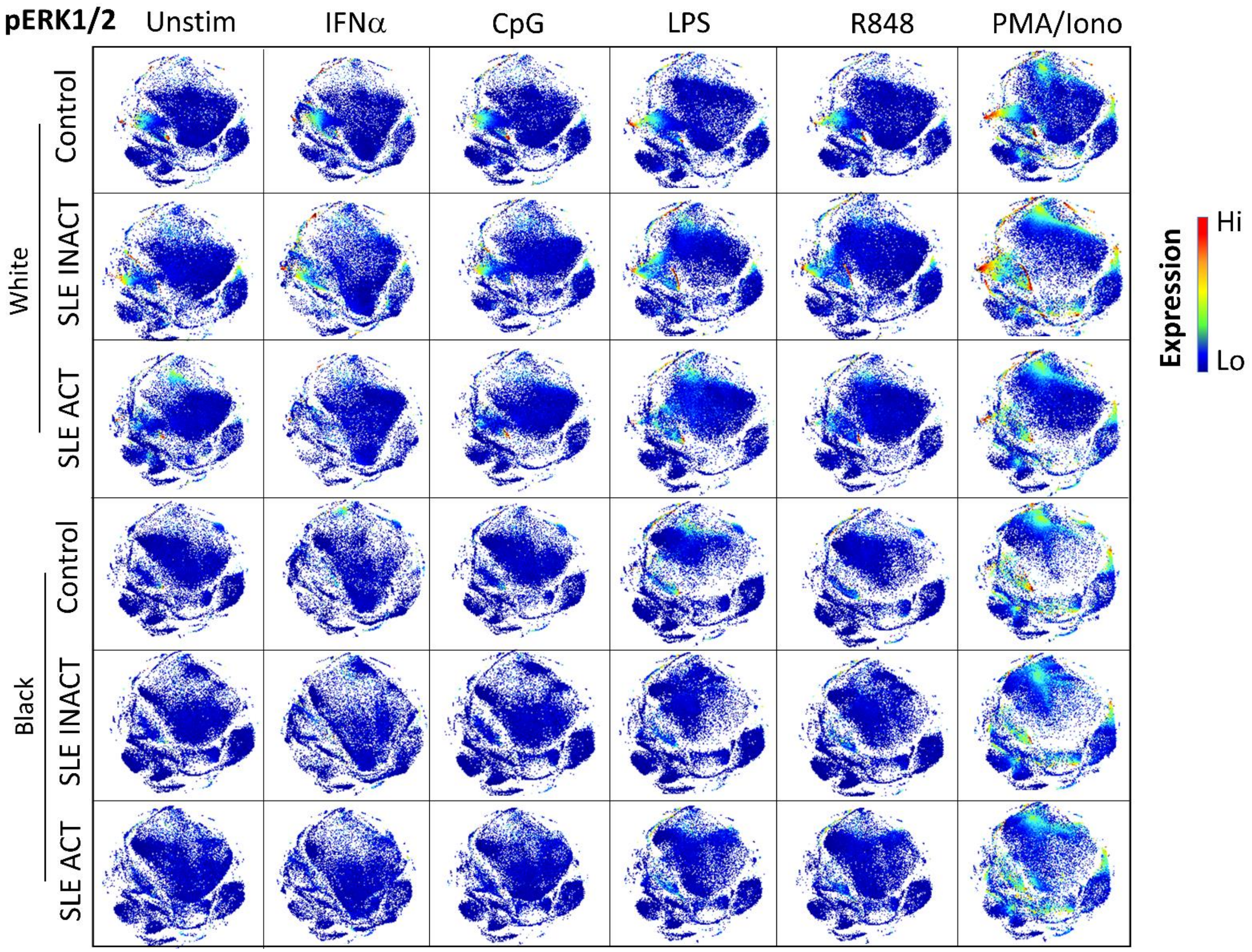

Supplemental Figure 20. pERK1/2 expression in cells by disease type. Concatenated files (430,000 cells/each) were used for each ancestry and disease group to visualize expression and changes in phospho-signaling markers with stimulation. High expression is indicated in red, medium in yellow, and low expression in blue.

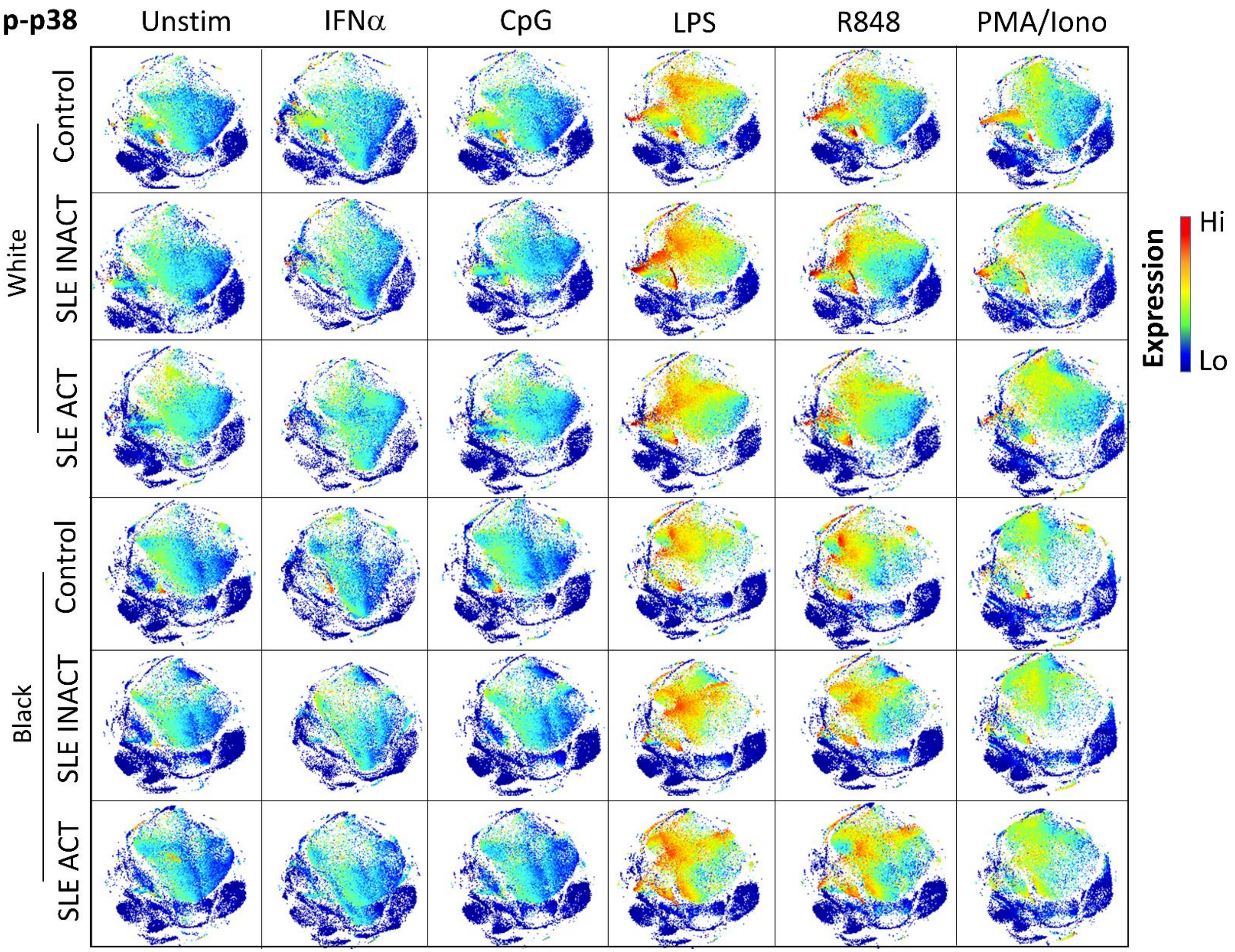

Supplemental Figure 21. P-p38 expression in cells by disease type. Concatenated files (430,000 cells/each) were used for each ancestry and disease group to visualize expression and changes in phospho-signaling markers with stimulation. High expression is indicated in red, medium in yellow, and low expression in blue.

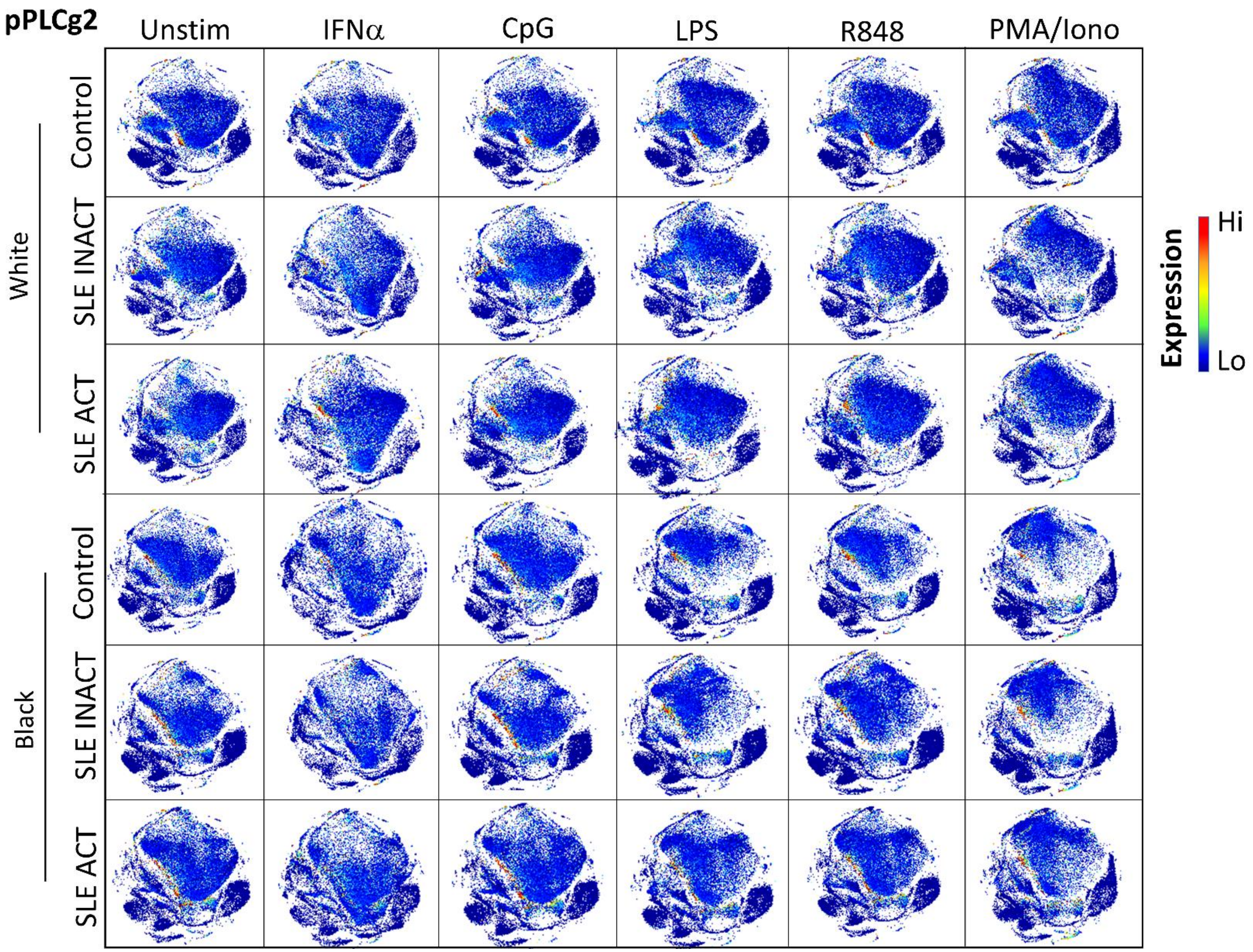

Supplemental Figure 22. pPLCγ2 expression in cells by disease type. Concatenated files (430,000 cells/each) were used for each ancestry and disease group to visualize expression and changes in phospho-signaling markers with stimulation. High expression is indicated in red, medium in yellow, and low expression in blue.

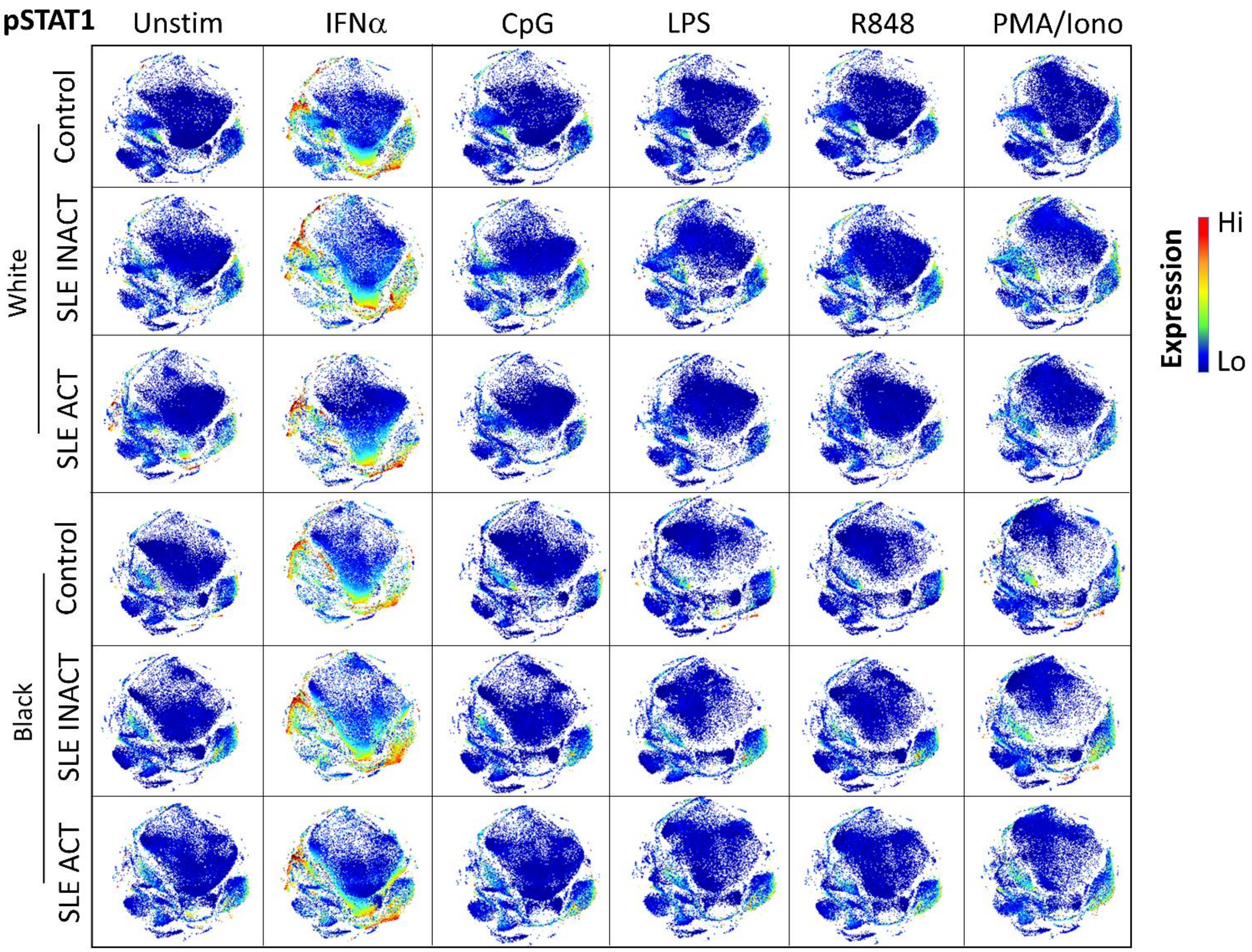

Supplemental Figure 23. pSTAT1 expression in cells by disease type. Concatenated files (430,000 cells/each) were used for each ancestry and disease group to visualize expression and changes in phospho-signaling markers with stimulation. High expression is indicated in red, medium in yellow, and low expression in blue.

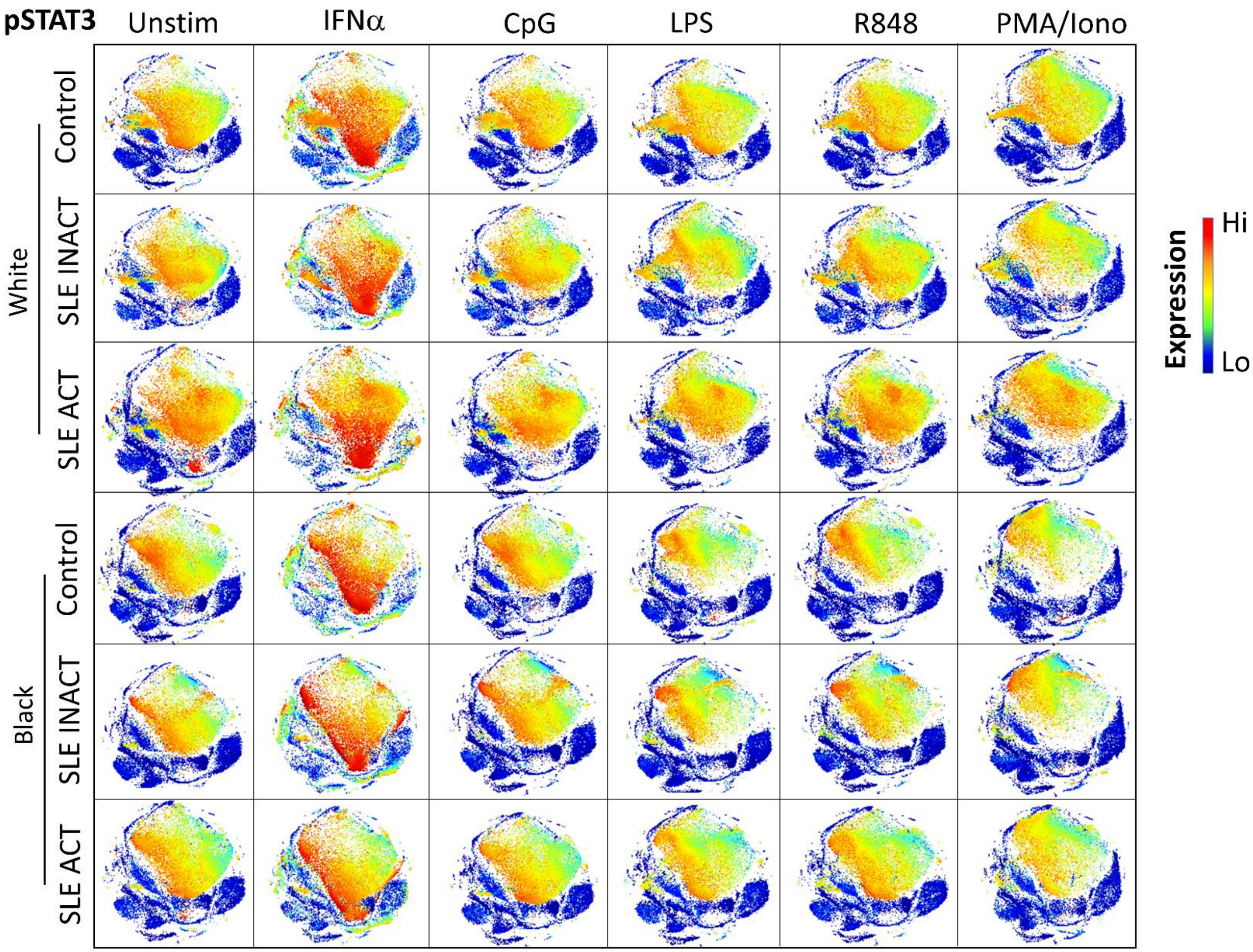

Supplemental Figure 24. pSTAT3 expression in cells by disease type. Concatenated files (430,000 cells/each) were used for each ancestry and disease group to visualize expression and changes in phospho-signaling markers with stimulation. High expression is indicated in red, medium in yellow, and low expression in blue.

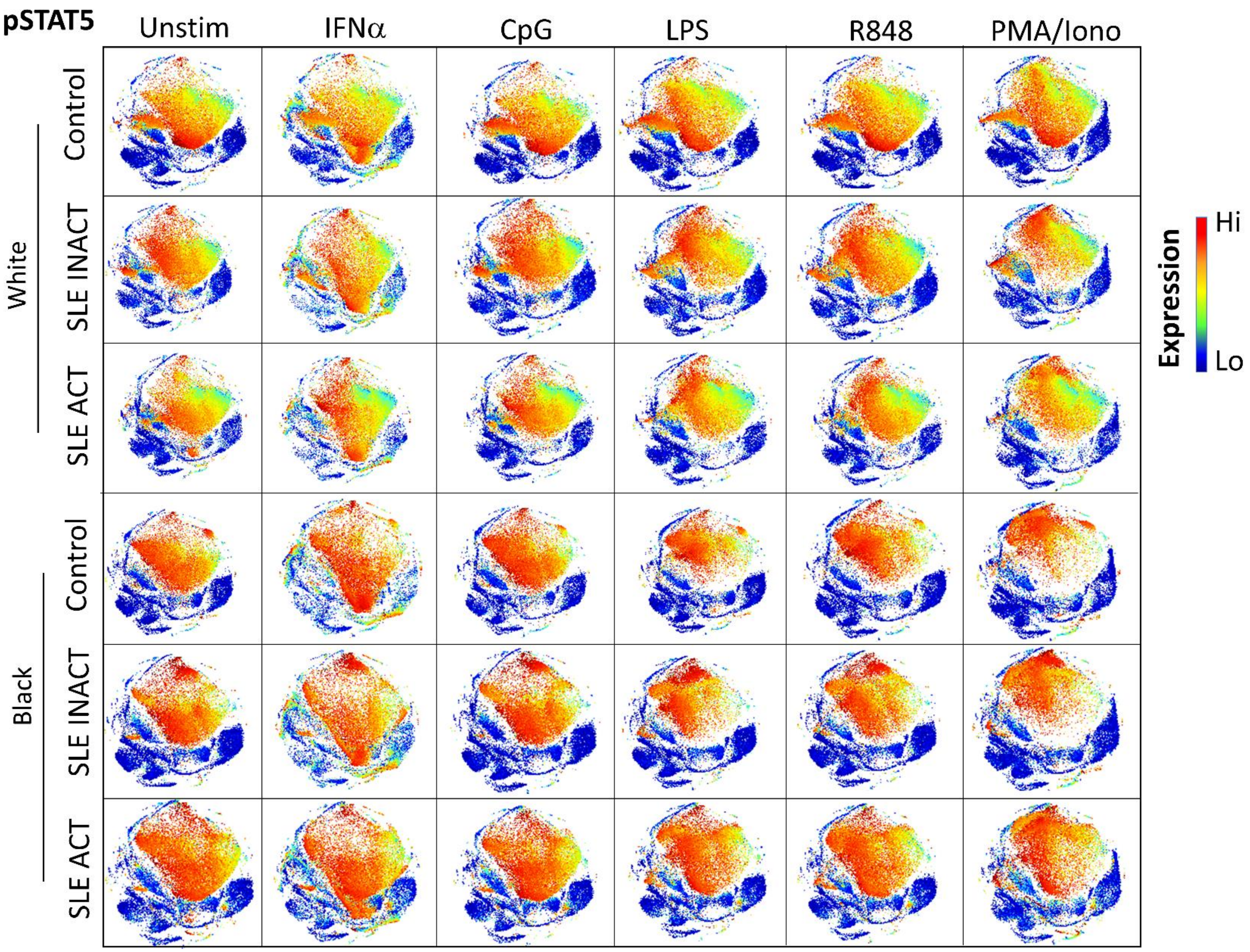

Supplemental Figure 25. pSTAT5 expression in cells by disease type. Concatenated files (430,000 cells/each) were used for each ancestry and disease group to visualize expression and changes in phospho-signaling markers with stimulation. High expression is indicated in red, medium in yellow, and low expression in blue.

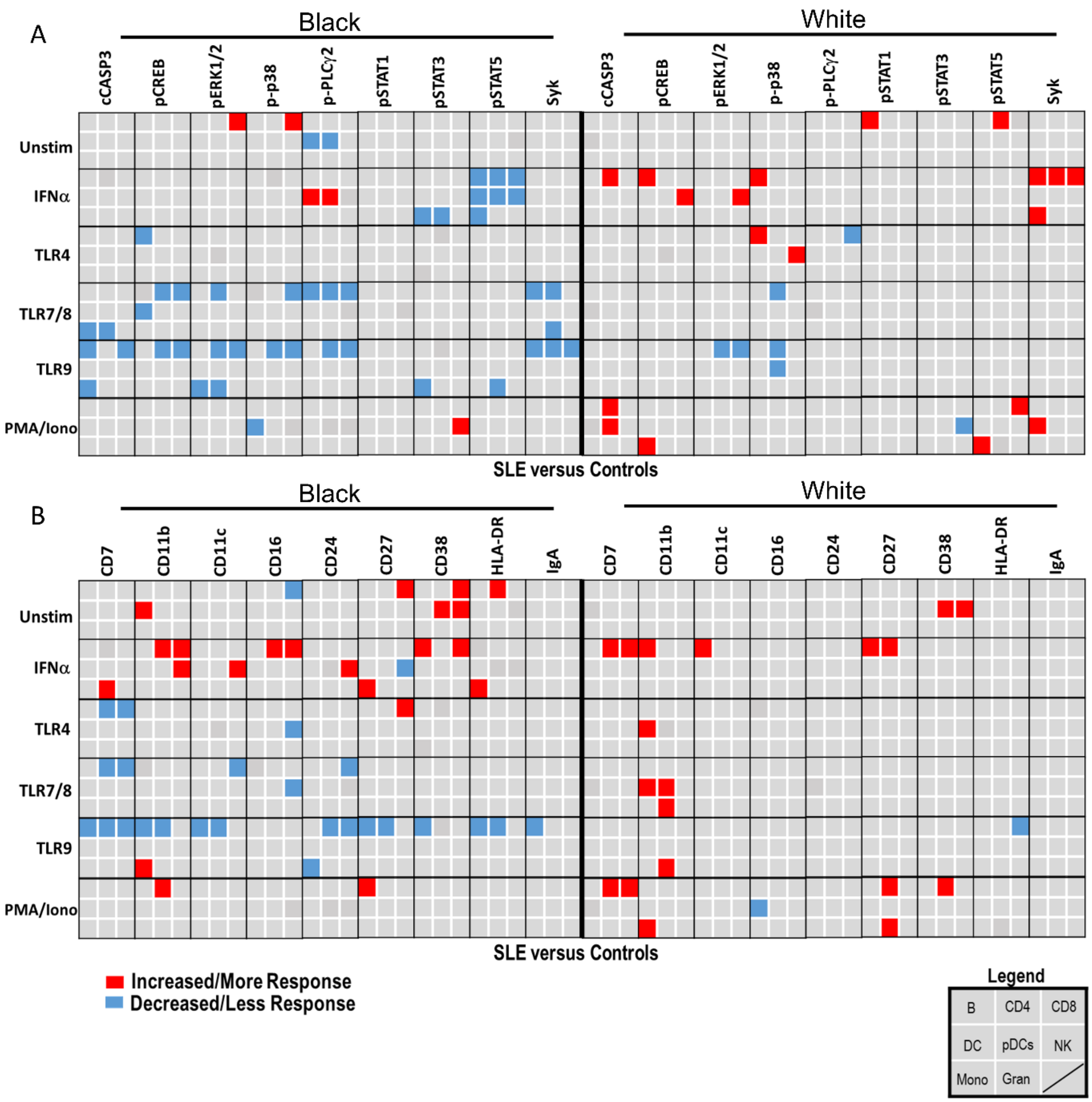

Supplemental Figure 26. Diminished TLR7/8 and TLR9 phospho-signaling responses in SLE patients. Peripheral whole blood from either Black or White healthy controls and SLE patients were stimulated for 4 or 15 minutes with either IFNα, PMA and ionomycin, or TLR4, TLR7/8, or TLR9 agonists. The median 95th percentile was used to calculate the fold change of (A) phopho-signaling markers and (B) activation markers in SLE patients over controls in 8 cell populations (See legend: B cells, CD4+ T cells, CD8+ T cells, dendritic cells [DCs], plasmacytoid DCs [pDCs], natural killer [NK] cells, monocytes [mono] and granulocytes [gran]). Significant increases or decreases in phosphorylation or activation markers following stimulation in a particular signaling molecule are noted by a blue box (decrease) or a red box (increase). The location of the box coincides with significant differences found in a specific cell population (refer to legend). $p<0.05$

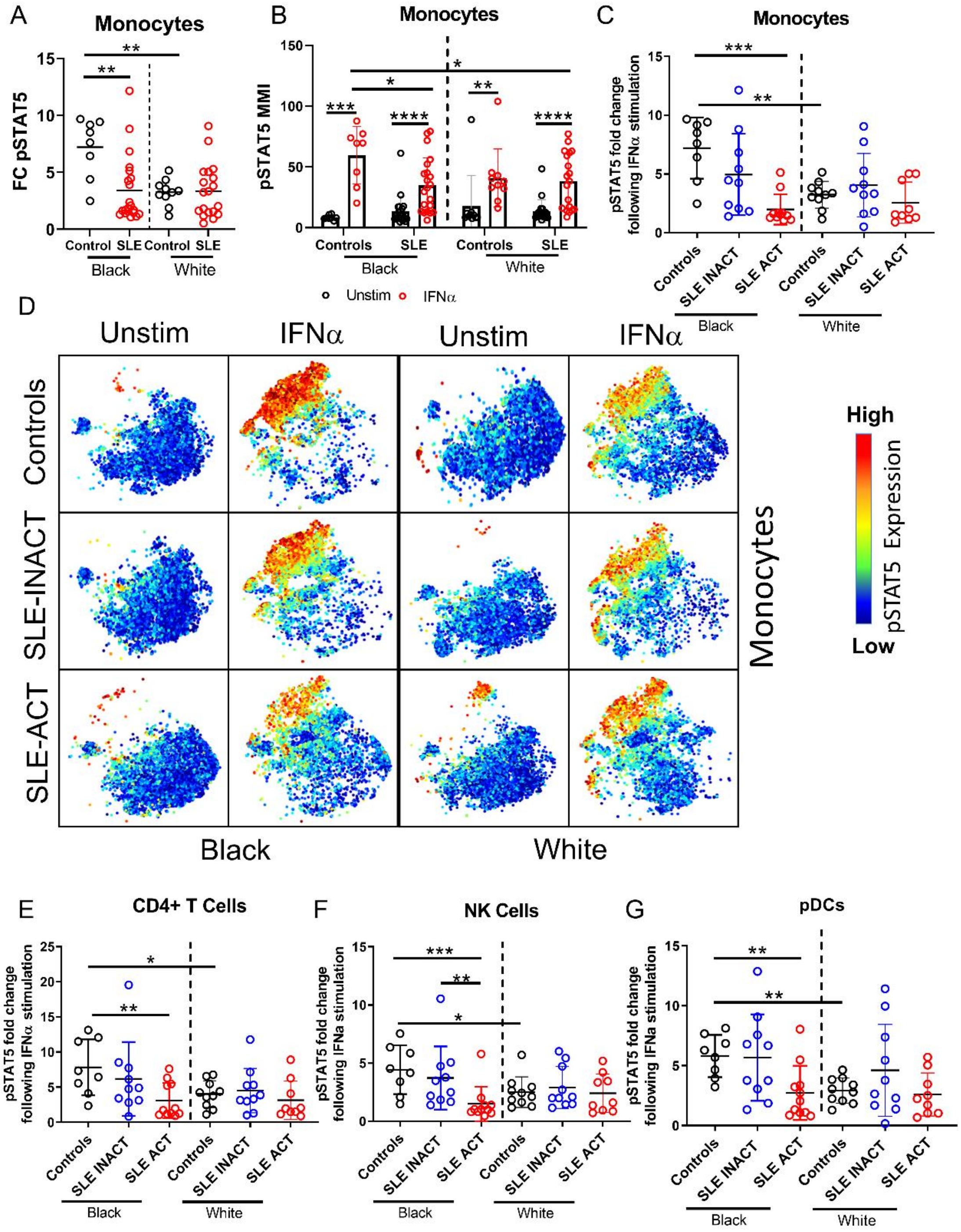

Supplemental Figure 27. IFNα-induced pSTAT5 responses are diminished in Black SLE ACT patients. (A) Fold change pSTAT5 levels in White and Black controls and SLE patients. (B) The total mean metal intensity of pSTAT5 (without fold change) with and without IFNα stimulation in White and Black controls and SLE patients. (C) Fold change in pSTAT5 following IFNα stimulation in SLE ACT and SLE INACT disease activity. (D) tSNE plots of monocytes showing pSTAT5 expression. The fold change of pSTAT5 following IFNα stimulation over unstimulated in (E) CD4+ T cells, (F) NK cells, and (G) pDCs. *p<0.05, **p<0.01, ***p<0.001, by Kruskal-Wallis test with two-tailed Mann-Whitney for multiple comparisons. Mean± SD shown.

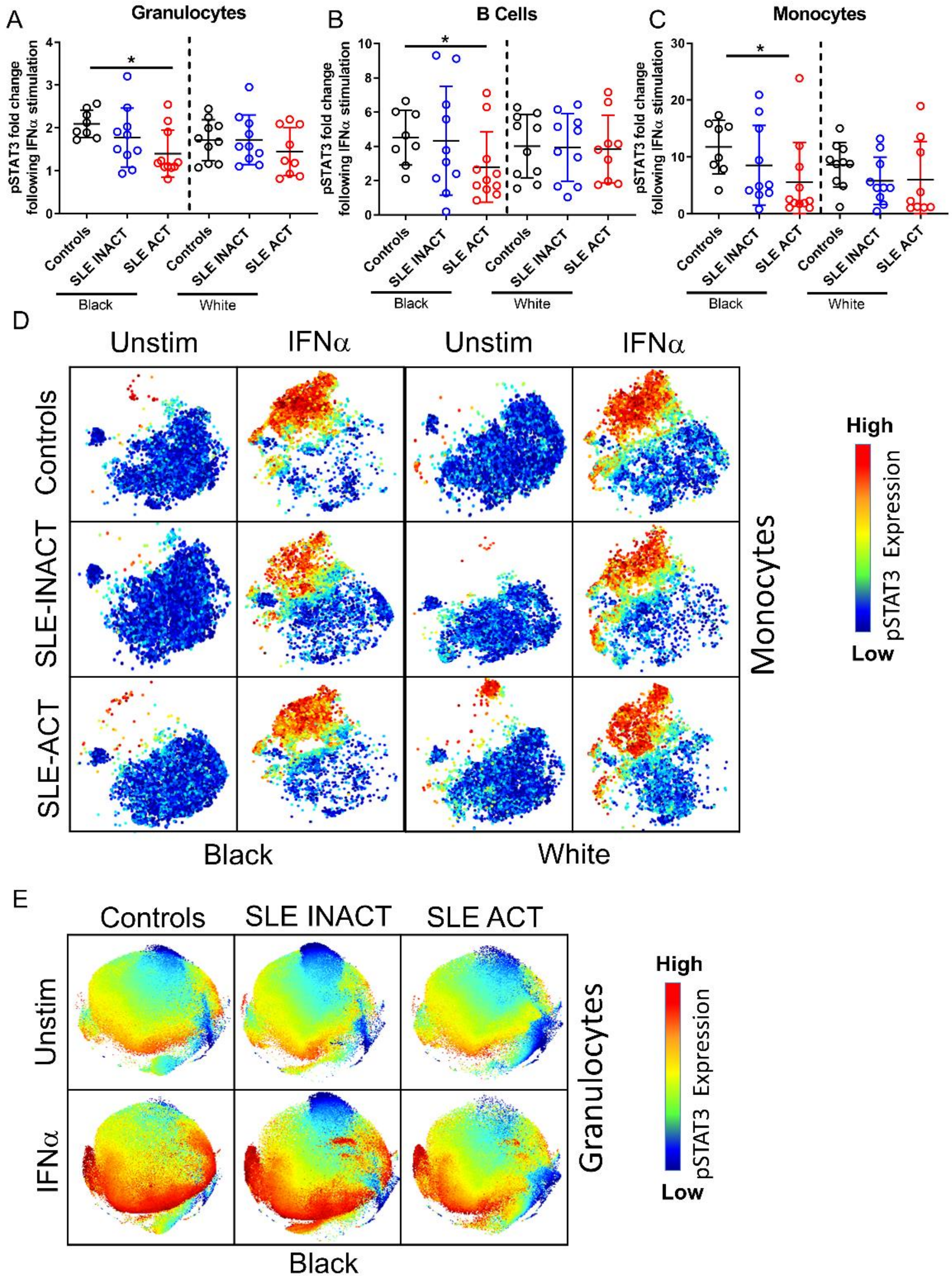

Supplemental Figure 28. IFNα–induced pSTAT3 responses are diminished in Black SLE ACT patients. The fold change of pSTAT3 following IFNα stimulation over unstimulated in (A) granulocytes, (B) B cells, and (C) monocytes. (D) Concatenated tSNE plots of monocytes showing pSTAT3 expression in unstimulated and IFNα-stimulated ancestry-disease groups. (E) Concatenated tSNE plots of granulocytes showing pSTAT3 expression in unstimulated and IFNα-stimulated Black disease groups. Red indicates high pSTAT3 expression, yellow medium expression, and blue low expression. *$p<0.05$, by Kruskal-Wallis test with two-tailed Mann-Whitney for multiple comparisons. Mean± SD shown.

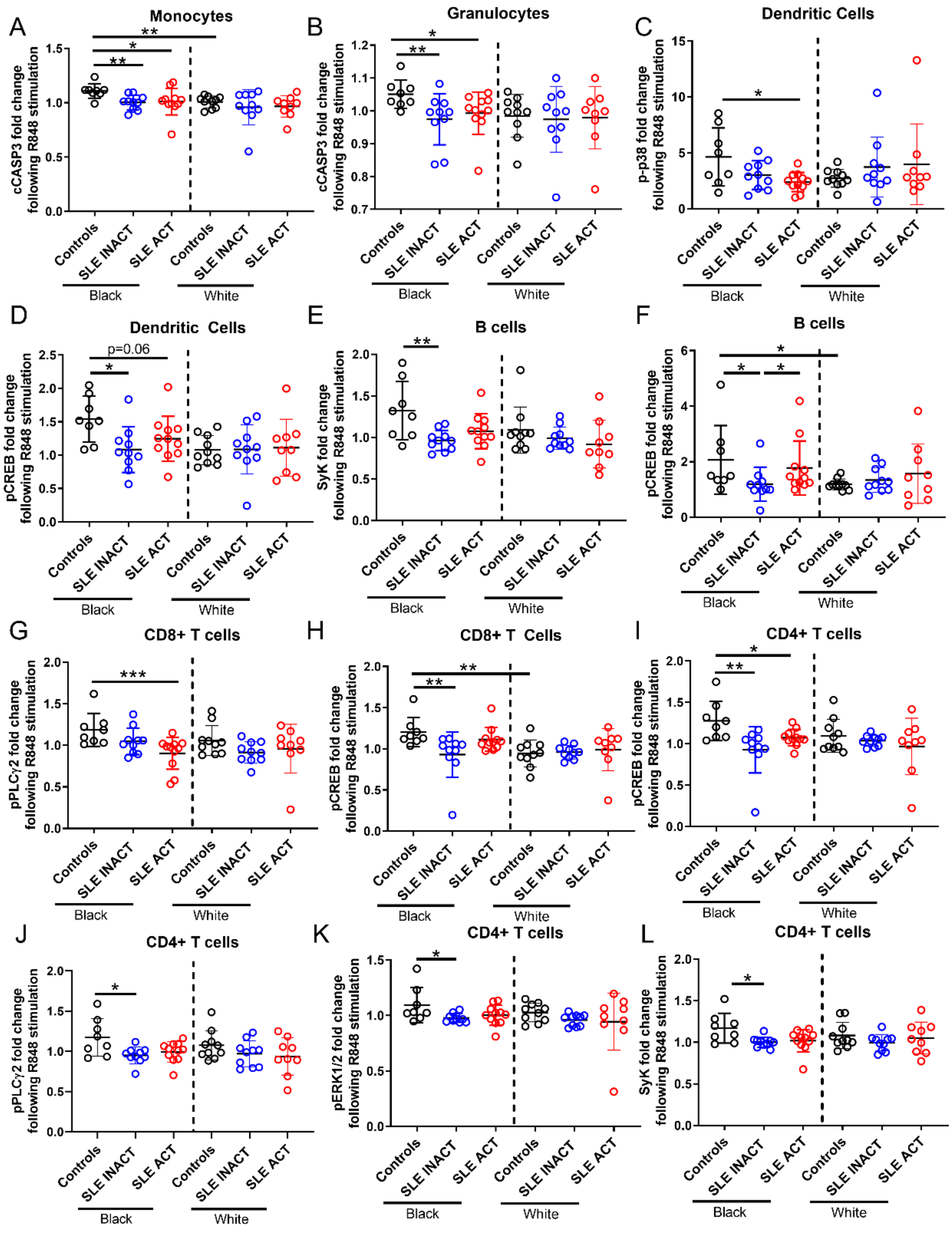

Supplemental Figure 29. Black SLE ACT patients exhibit diminished R848 (TLR7/8) phospho-signaling responses. Fold change levels were calculated using the 95th percentile MMI (mean metal intensity) of R848 stimulated over unstimulated levels for specific cell subsets. Significant differences were observed in (A) cCASP3-Monocytes, (B) cCASP3-Granulocytes, (C) p-p38- DCs, (D) pCREB-DCs, (E) Syk-B cells, (F) pCREB-B cells, (G) pPLCγ2-CD8+ T Cells, (H) pCREB-CD8+T Cells, (I) pCREB-CD4+ T Cells, (J) pPLCγ2-CD4+ T Cells, (K) pERK1/2-CD4+ T Cells, and (L) Syk-CD4+ T Cells. *p<0.05, by Kruskal-Wallis test with two-tailed Mann-Whitney for multiple comparisons. Mean± SD shown

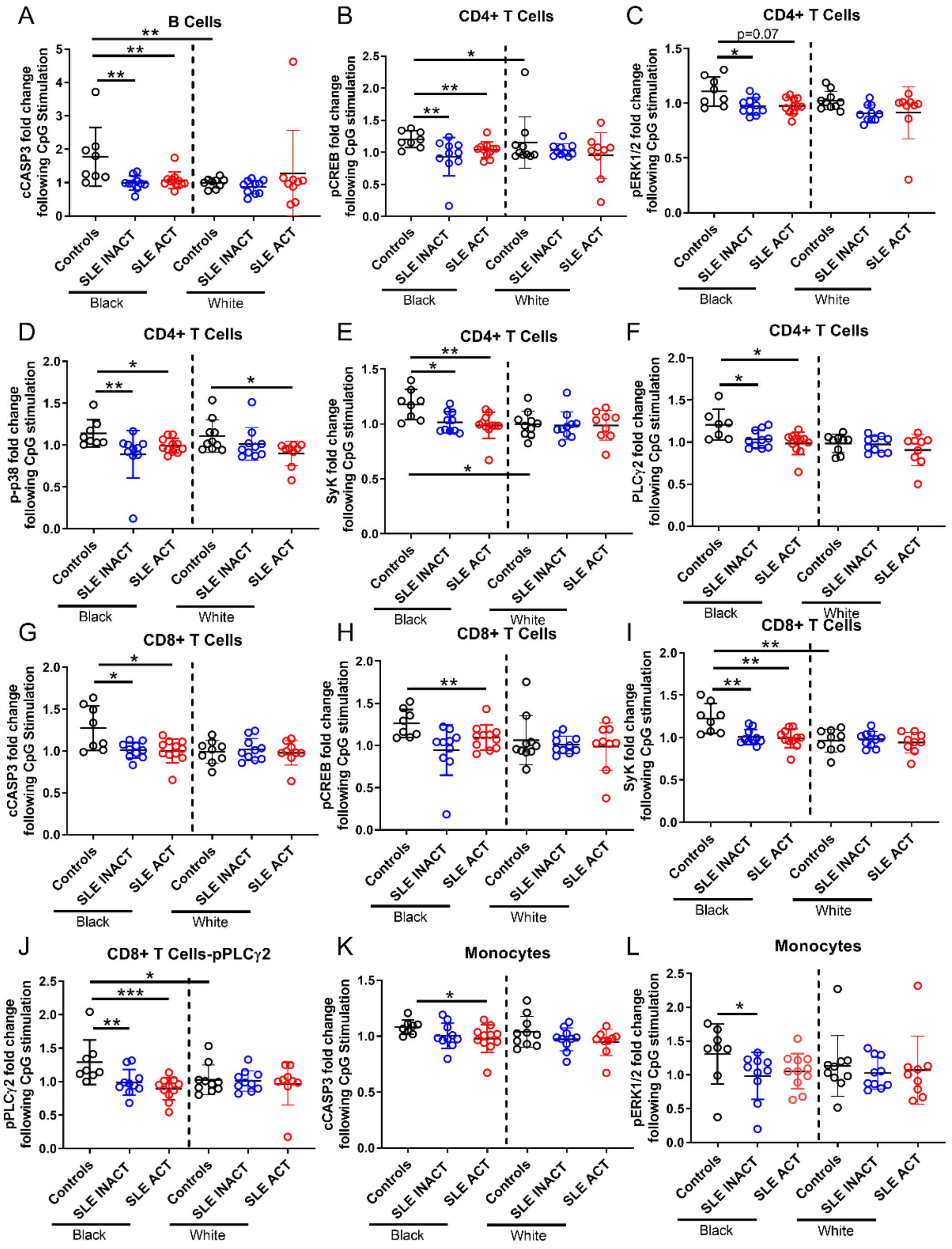

Supplemental Figure 30. Black SLE ACT patients exhibit diminished CpG (TLR9) phospho-signaling responses. Fold change levels were calculated using the 95th percentile MMI (mean metal intensity) of CpG stimulated over unstimulated levels for specific cell subsets. Significant differences were observed in (A) cCASP3-B cells, (B) pCREB-CD4+ T Cells, (C) pERK1/2-CD4+ T Cells, (D) p-p38-CD4+ T Cells, (E) Syk-CD4+ T Cells, (F) pPLCγ2-CD4+ T Cells, (G) cCASP3-CD8+ T Cells, (H) pCREB-CD8+T Cells, (I) Syk-CD8+ T Cells, (J) pPLCγ2-CD8+ T Cells, (K) cCASP3-Monocytes, and (L) pERK1/2-Monocytes. *$p<0.05$, by Kruskal-Wallis test with two-tailed Mann-Whitney for multiple comparisons. Mean± SD show.

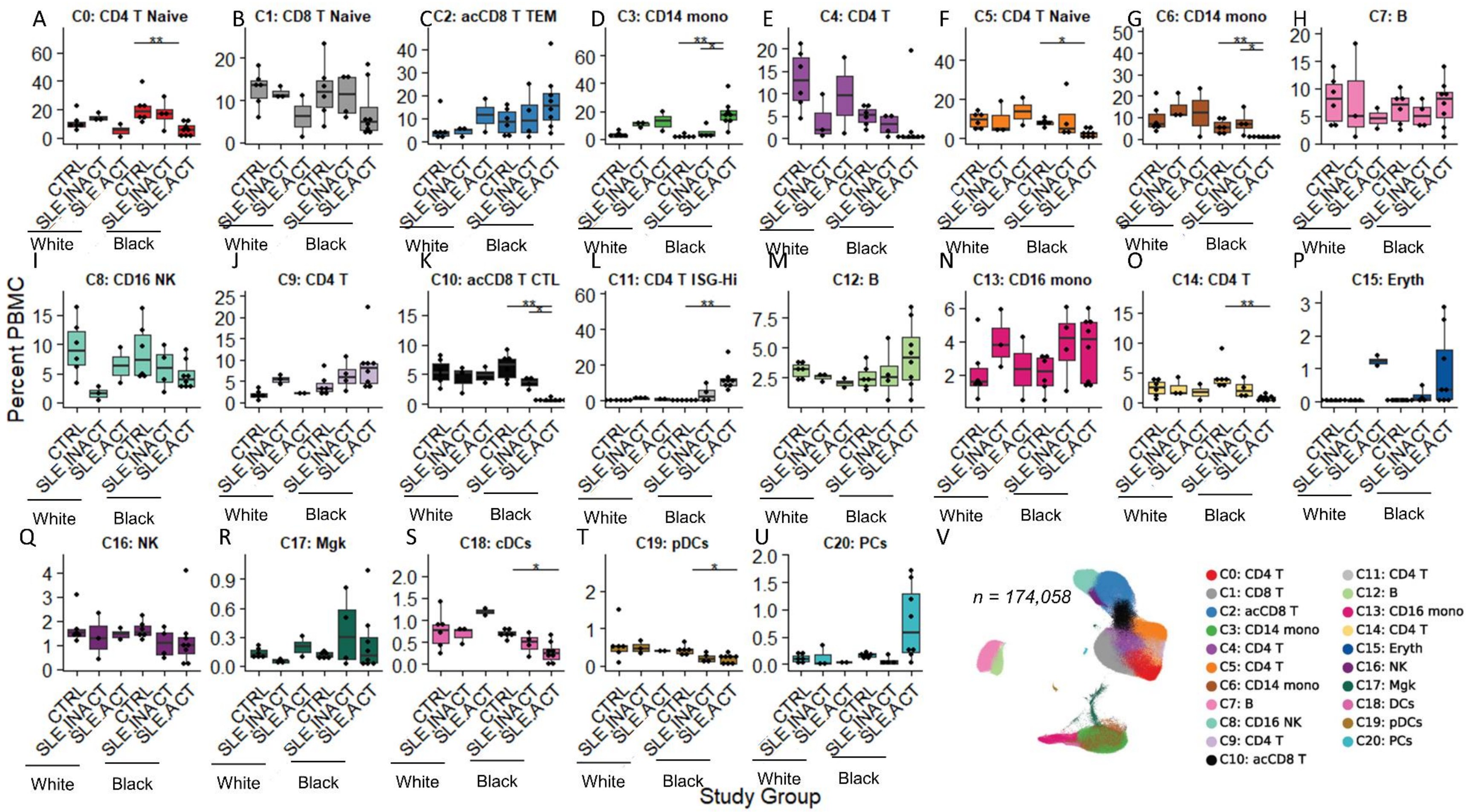

Supplemental Figure 31. GSE135779 clustering and all cell subset frequencies. PBMCs from 29 controls and patients with SLE were assessed by droplet-based scRNA-sequencing using a 10x genomics platform. Frequencies for **(A)** naïve CCR7+ CD4+ T cells **(B)** naïve CD8+ T Cells, **(C),** acCD8+ GZMA+ GZMH+ TEM Cells, **(D)** CD14 monocytes, **(E)** Naïve CD4+ T cells- RPS3A+ RPLP2+ **(F)** CD4+ T-LTB+, IL-32+, CD127+ Cells **(G)** CD14 monocytes **(H)** B cells **(I)** CD16+ NK Cells **(J)** CD4+ T Cells- ITGB1+ ITM2A+ **(K)** acCD8+ GZMK+ T Cells, **(L)** ISG-Hi CD4+ T cells, **(M)** B Cells, **(N)** CD16+ monocytes, **(O)** CD4+ T cells- C1orf56+, CDC42SE1+, CD16or54+, **(P)** erythrocytes, **(Q)** NK Cells, **(R)** megakaryocytes, **(S)** cDCs, **(T)** pDCs, and **(U)** plasma cells. **(V)** UMAP plot representing the 21 clusters of cells are shown. The putative identity of each cluster was determined using gene expression and protein expression from CITE-seq. UMAP representation of confirmatory dataset as presented in its original publication. *p<0.05, **p<0.01, ***p<0.001, by pairwise Wilcoxon rank sum tests between disease groups with Benjamini-Hochberg correction for multiple comparisons.

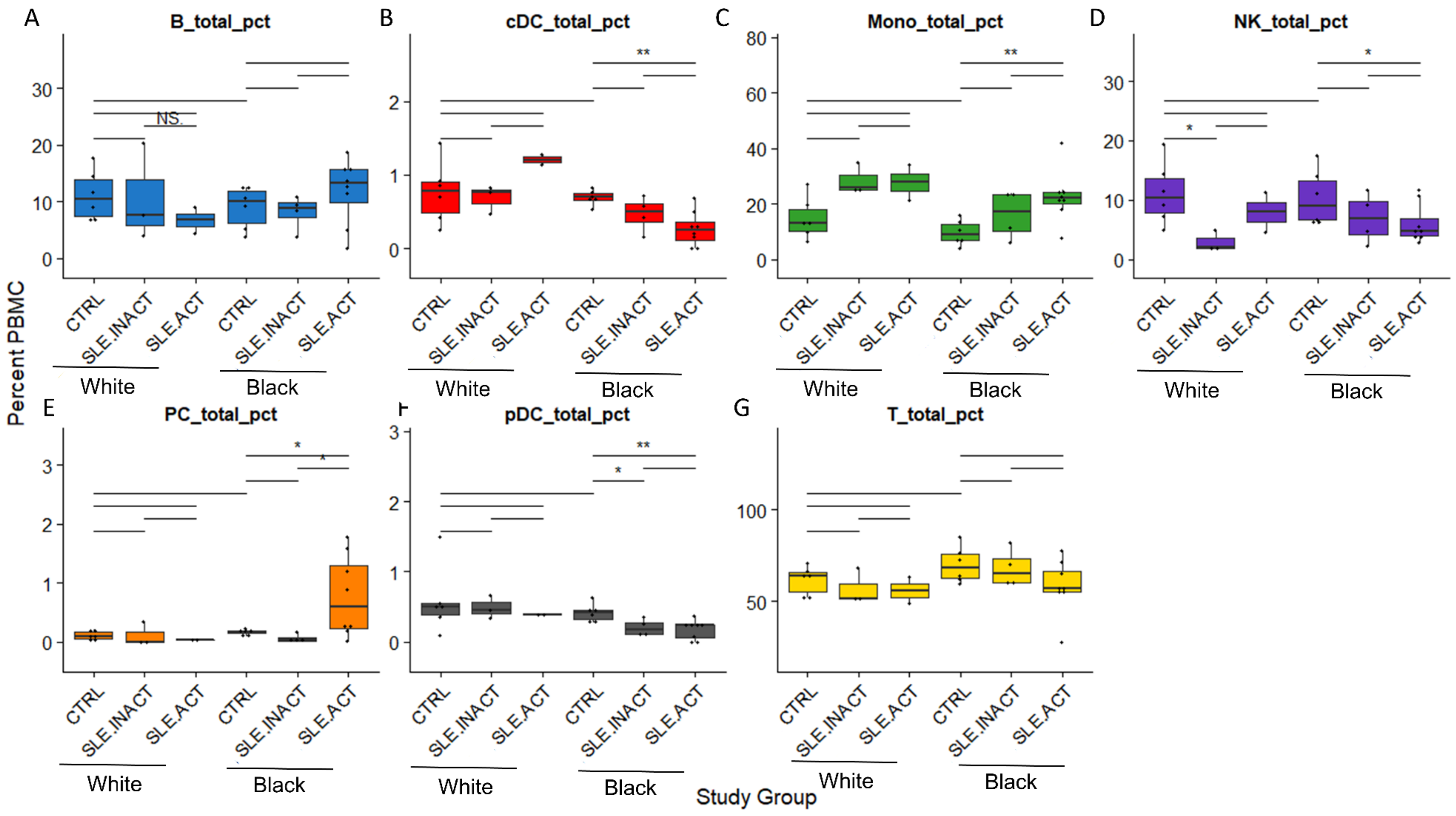

Supplemental Figure 32. GSE135779 clustering and main cell subset frequencies. PBMCs from 29 controls and patients with SLE were assessed by droplet-based scRNA-sequencing using a 10x genomics platform. Frequencies for total **(A)** B cells **(B)** cDCs, **(C),** monocytes, **(D)** NK cells, **(E)** plasma Cells **(F)** pDCs and, **(G)** total T cells. *p<0.05, and **p<0.01., by pairwise Wilcoxon rank sum tests between disease groups with Benjamini-Hochberg correction for multiple comparisons.

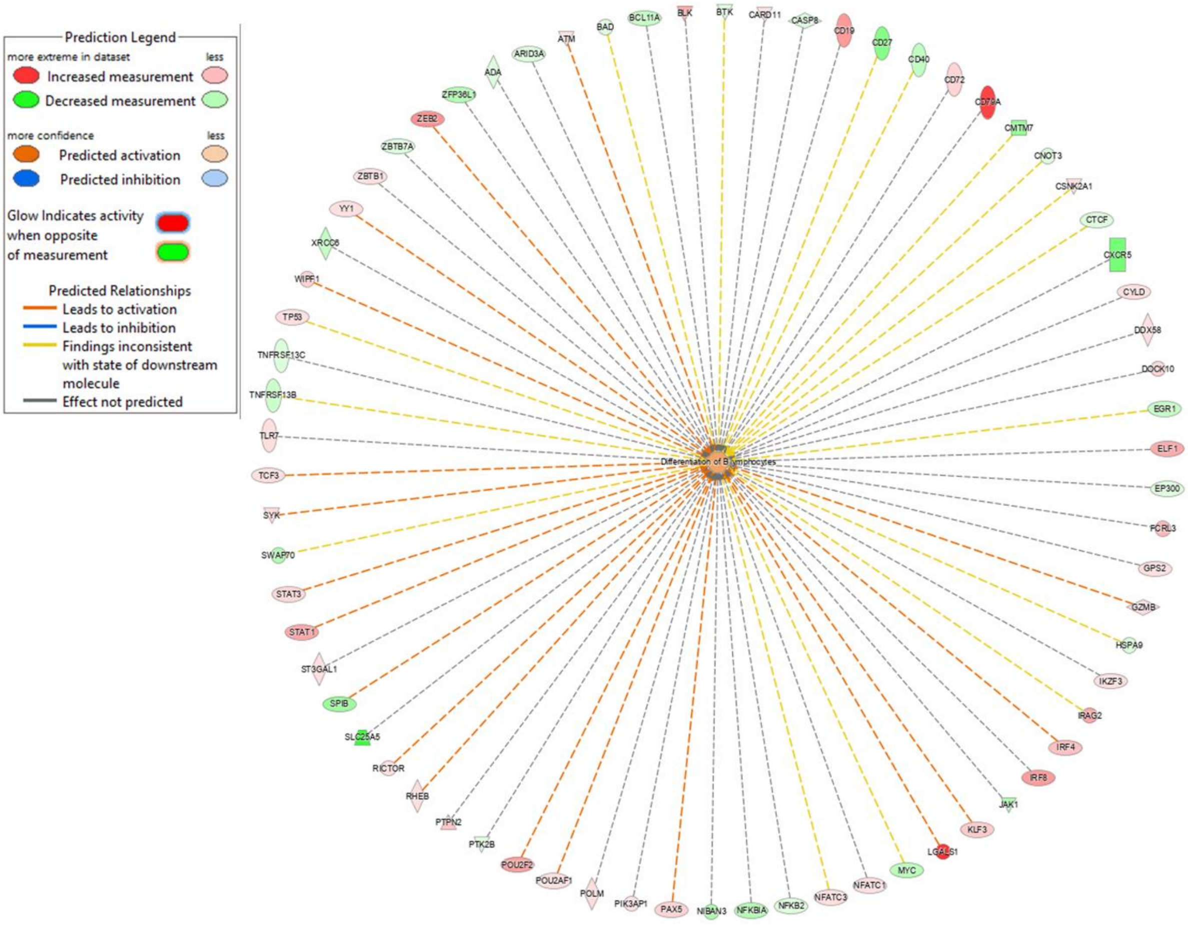

Supplemental Figure 33. GSE135779 pathway analysis reveals increased B cell differentiation pathways in active lupus patients. Ingenuity pathway analyses of differentially expressed genes identified differences in differentiation of B cells between SLE ACT disease activity patients and controls, specifically in memory B cells. Red indicates increased measurement of that gene expression pathway and green indicated decreased measurement.

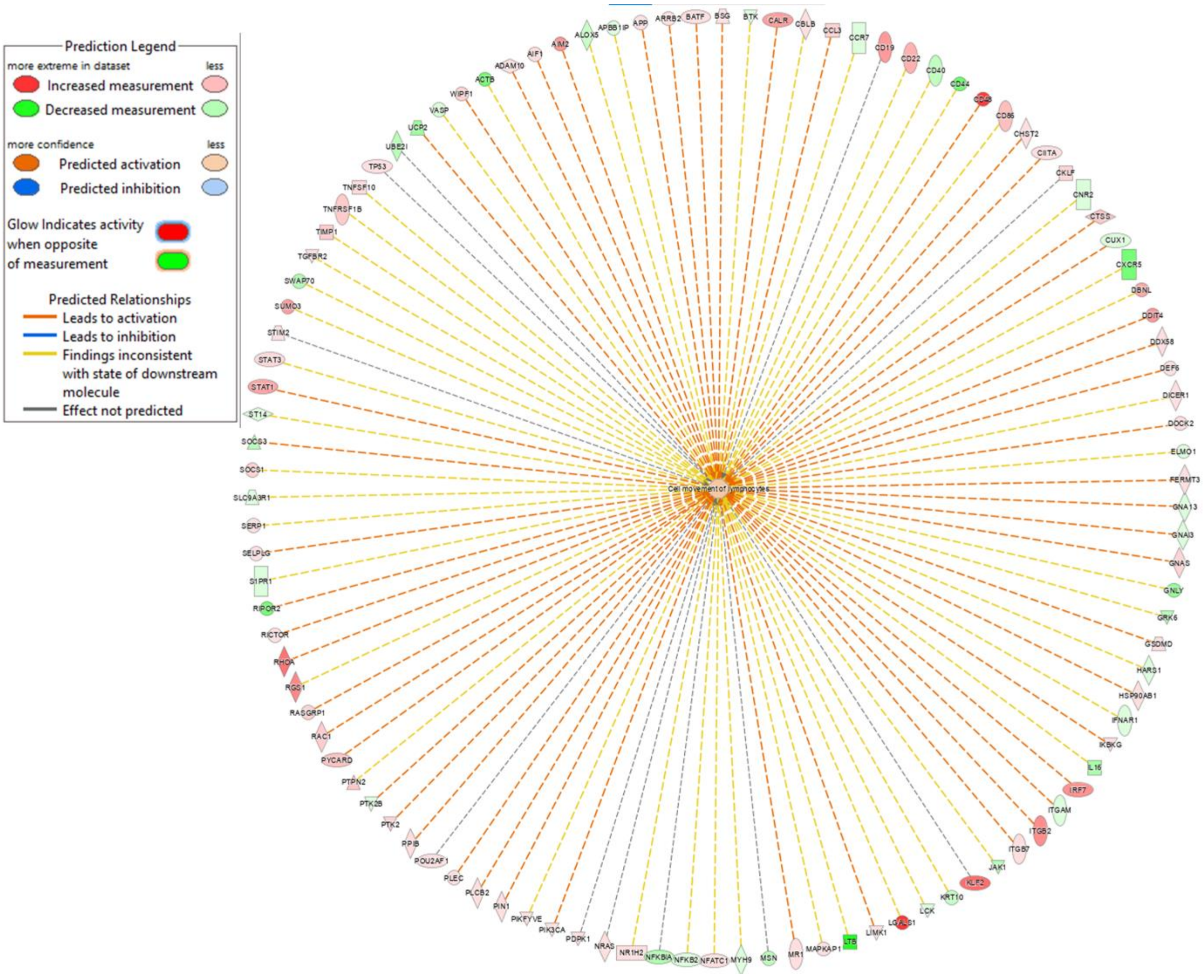

Supplemental Figure 34. GSE135779 pathway analysis reveals increased gene expression pathways associated with cell movement. Ingenuity pathway analyses of differentially expressed genes identified differences in migration/cell movement of B cells between SLE ACT disease activity patients and controls, specifically in memory B cells. Red indicates increased measurement of that gene expression pathway and green indicated decreased measurement.

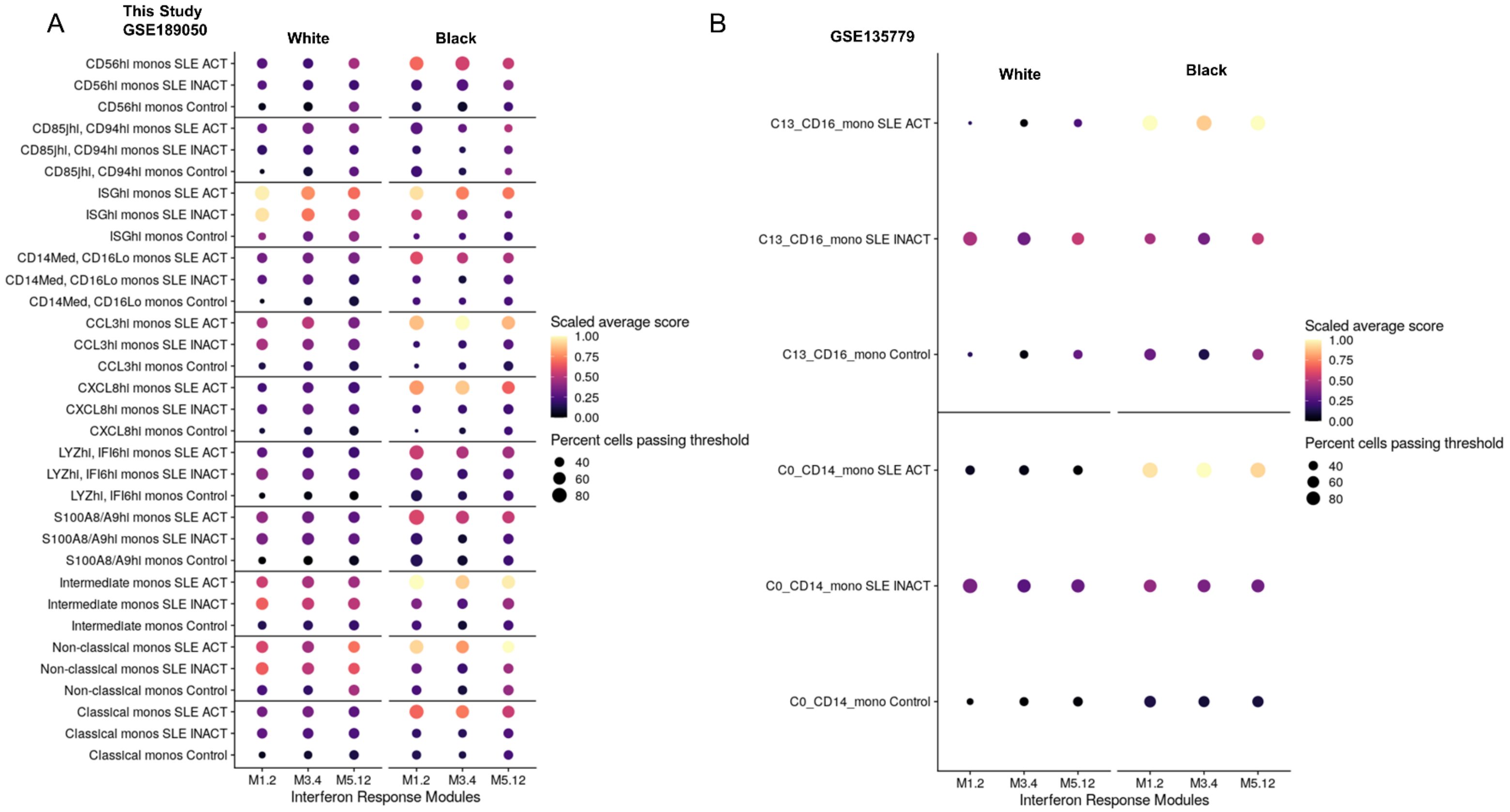

Supplemental Figure 35. IFN response modules increase with disease activity in Black patients in both GSE135779 and this study (GSE189050). Gene expression for specific IFN response modules are shown using dot plots for all monocyte subsets found by scRNA-sequencing in each study. A side by side comparison of **(A)** this study versus **(B)** GSE135779 shows similar signatures between the two studies with different patterns between White and Black patients.

A

B cell clusters

	C7: B			C12: B		
	CTRL	SLE INACT	SLE ACT	CTRL	SLE INACT	SLE ACT
LPS	6.406	-0.222	8.191	5.329	1.974	9.651
poly I:C	4.212	1.985	7.305	1.552	2.836	5.989
TLR7	N/A	N/A	4.502	N/A	1.441	N/A
R848	1.143	1.564	2.426	1.682	1.353	2.96
TLR9	N/A	1.525	4.626	N/A	2.082	3.433
CpG 2006	N/A	1.587	4.504	N/A	1.206	3.195
CpG 2216	N/A	N/A	N/A	N/A	0.915	N/A

Activation z-score

3, 2, 1, 0, -1, -2, -3

More activity in Black cells vs White cells

Less activity in Black cells vs White cells

B

Monocyte Clusters

	CD14 Mono			CD16 Mono		
	CTRL	SLE INACT	SLE ACT	CTRL	SLE INACT	SLE ACT
LPS	2.442	6.323	9.113	2.977	5.421	7.639
poly I:C	3.969	5.774	8.544	2.588	4.392	7.241
TLR7	2.683	3.615	4.254	2.454	3.325	4.176
R848	1.78	2.77	1.67	2.119	1.329	3.33
TLR9	N/A	3.398	4.453	1.21	2.693	3.951
CpG 2006	2.585	3.877	4.145	1.339	2.48	5.263
CpG 2216	N/A	1.953	2.327	1.987	2.173	1.508

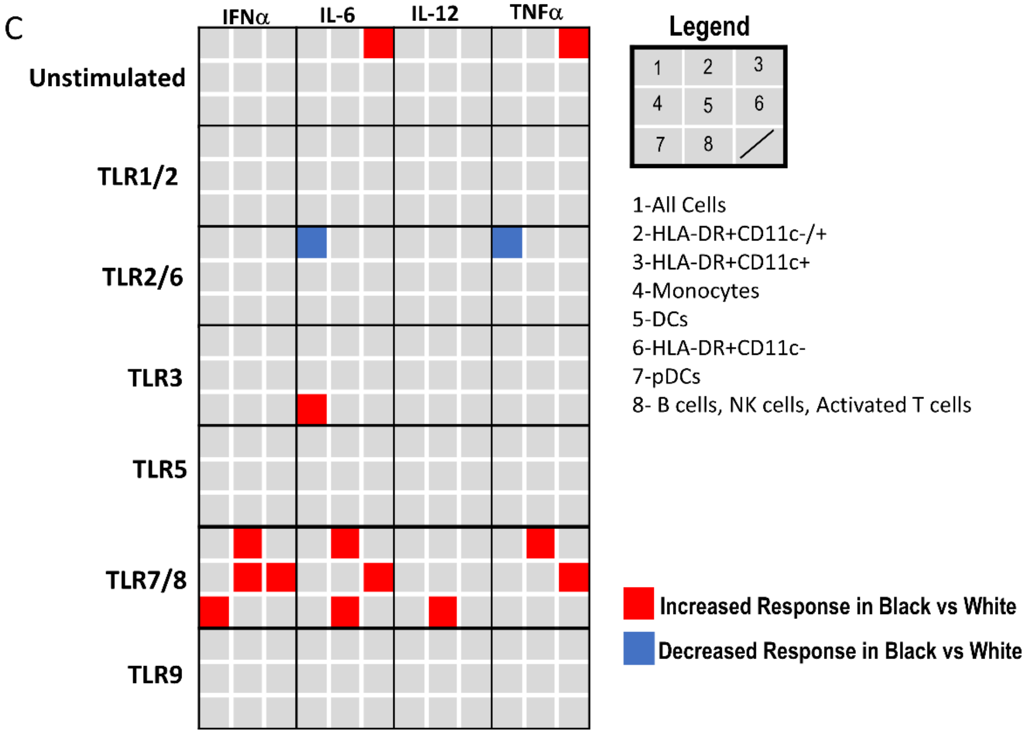

Supplemental Figure 36. TLR signaling pathways are elevated in Black SLE patients and TLR7/8 pathways are also elevated in Black controls. Differentially expressed genes between Black and White samples identified by scRNA-seq in GSE135779 of **(A)** B cells and **(B)** monocyte cell clusters were assessed by Ingenuity Pathway Analysis to determine differences in the activity of TLR pathways. Red indicates increased TLR pathway activity in Black versus White, White indicates no change, and blue indicates decreased TLR activity. **(C)** PBMCs from 14 subjects in SDY40 were stimulated for 18 hours, with brefeldin A added at the end of stimulation, for assessment of intracellular cytokines. Cells were assessed for IFNα, IL-6, IL-12, and TNFα by flow cytometry. Significantly increased (red) or decreased (blue) production of cytokines are reported by cell type based on the location within the grey box. $p<0.05$

SDY40-IMMPORT study

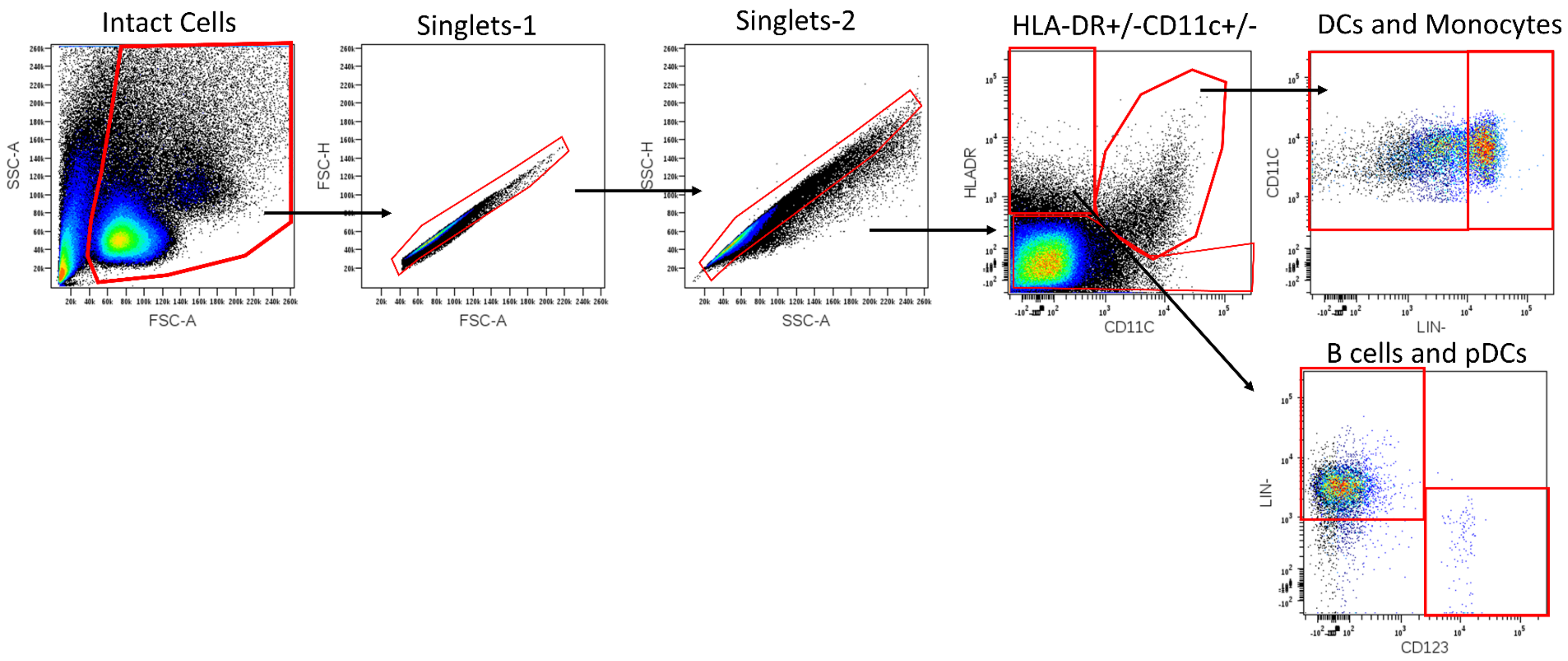

Supplemental Figure 37. Gating strategy for SDY40. Cells were first gated on intact, singlet cells, following by cell type-specific subgating using the markers available in the panel.

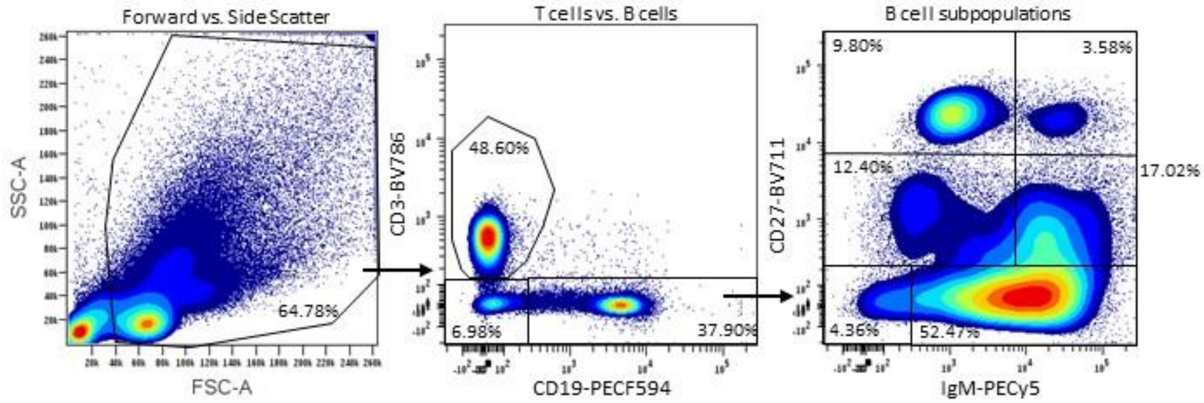

Supplemental Figure 38. Representative gating hierarchies for B cell subpopulations for in vitro TLR stimulation experiment. Confirmation gating hierarchy used to access the B cell subpopulations from 10 healthy control Black and 10 healthy control White PBMCs stimulated with TLR9, TLR7/8, IFNα, or left unstimulated for 7 days. In addition, all major cell populations and B cell subsets were assessed using tSNE as depicted in Figure 7.

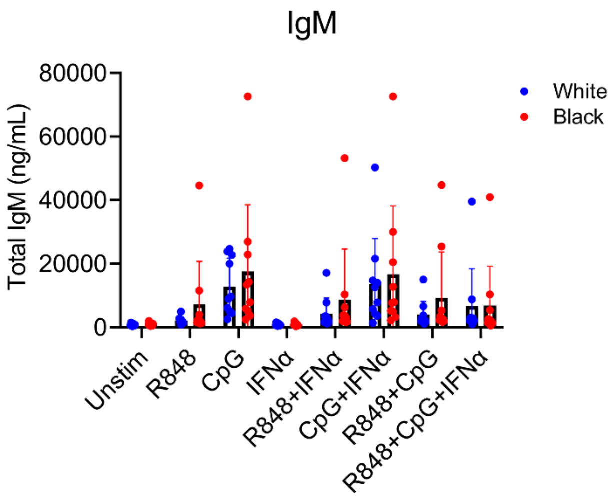

Supplemental Figure 39. IgM culture supernatant levels after 7 days stimulation with TLR agonists and/or IFNα. PBMCS from 10 healthy White controls and 10 healthy Black controls without autoimmune disease characteristics were plated at 1 million cells/well and stimulated alone or in combination with TLR7/8 (R848), TLR9 (CpG), or IFNα for 7 days. Culture supernatants were collected and assayed for IgM antibody levels using ELISA. No significant differences were observed between White and Black subjects.

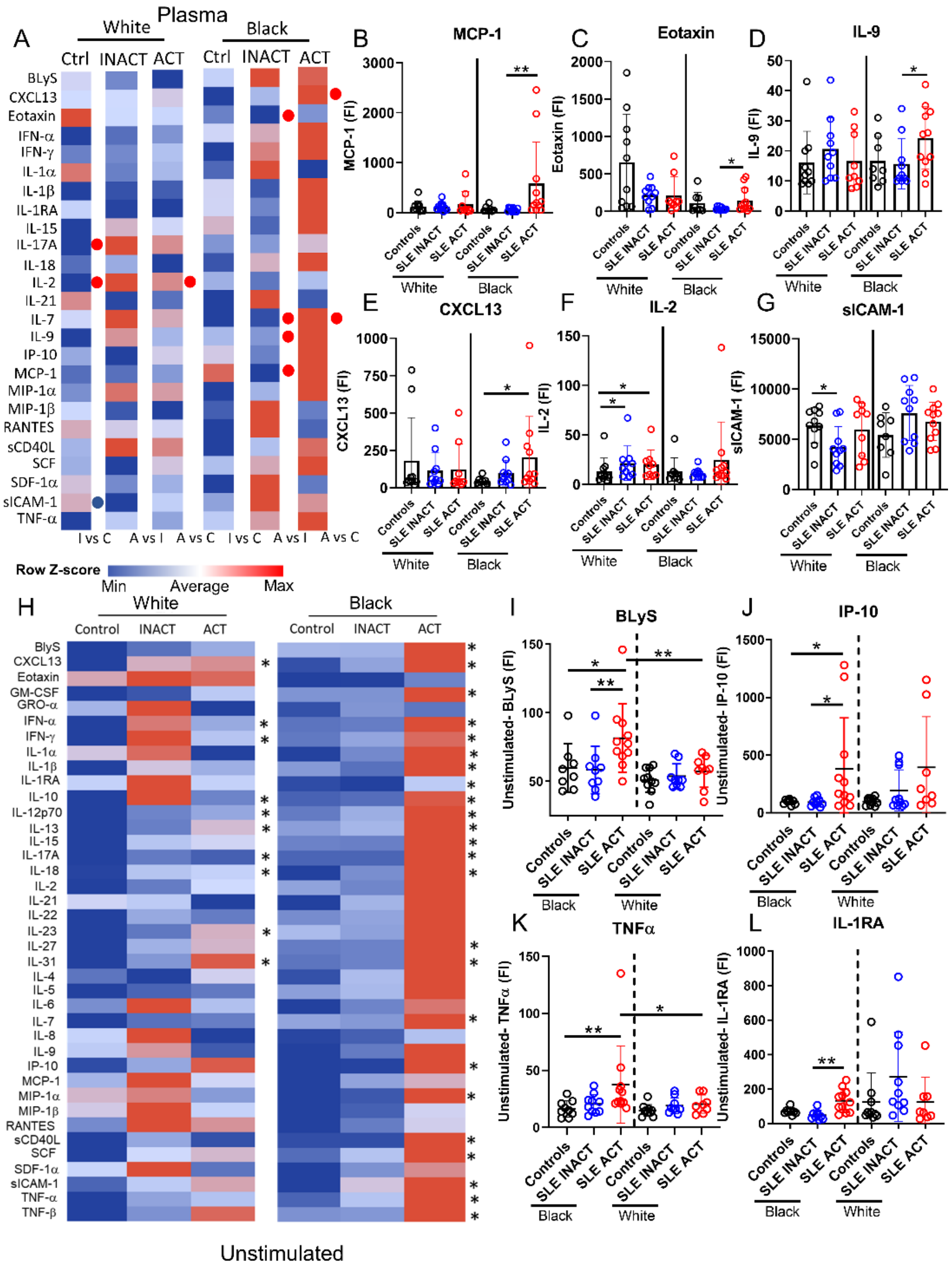

Supplemental Figure 40. Plasma and unstimulated cytokine levels are elevated in Black SLE ACT patients. Plasma and whole blood culture supernatant cytokine levels were assessed using multiplex assay and ELISA (BLyS) to measure 39 different soluble mediators. (A) A heatmap summary of cytokine levels by ancestry disease groups is shown for plasma cytokines. Significant differences (p<0.05) between disease groups are noted with a red dot (significantly increased) or blue dot (significant decrease). Dot plots of significant differences in plasma levels of (B) MCP-1, (C) eotaxin, (D) IL-9, (E) CXCL13, (F) IL-2, (G) sICAM-1 are shown. (H) A heatmap summary of unstimulated culture supernatants reflect cytokine levels after 24 hours in culture. Significant differences (p<0.05) among White or Black controls, SLE INACT, and SLE ACT patients are noted with an asterisk. Notably, Black SLE ACT patients had significantly higher levels (I) BLyS, (J) IP-10, (K) TNFα, and (L) IL-1RA following 24 hour incubation. *p<0.05, **p<0.01, by Kruskal-Wallis test with two-tailed Mann-Whitney for multiple comparisons. Mean± SD shown.

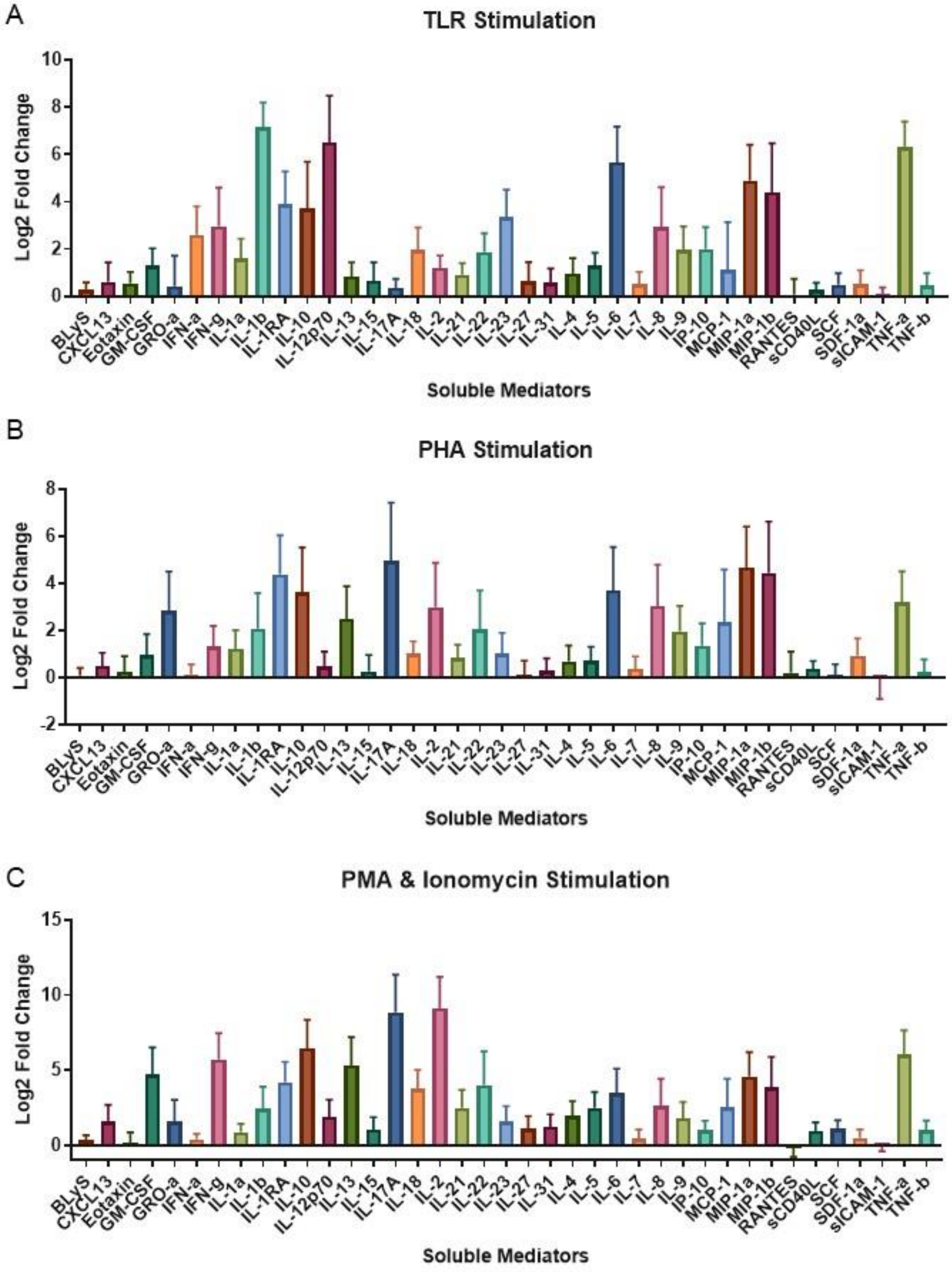

Supplemental Figure 41. Overall cytokine production after different stimulation conditions. To illustrate the cytokine pathways being driven in whole blood by (A) TLR (TLR4/7/8/9 agonists), (B) PHA, and (C) PMA/ionomycin stimulations, the log2 fold change following a 24-hour incubation for all 58 subjects is shown.

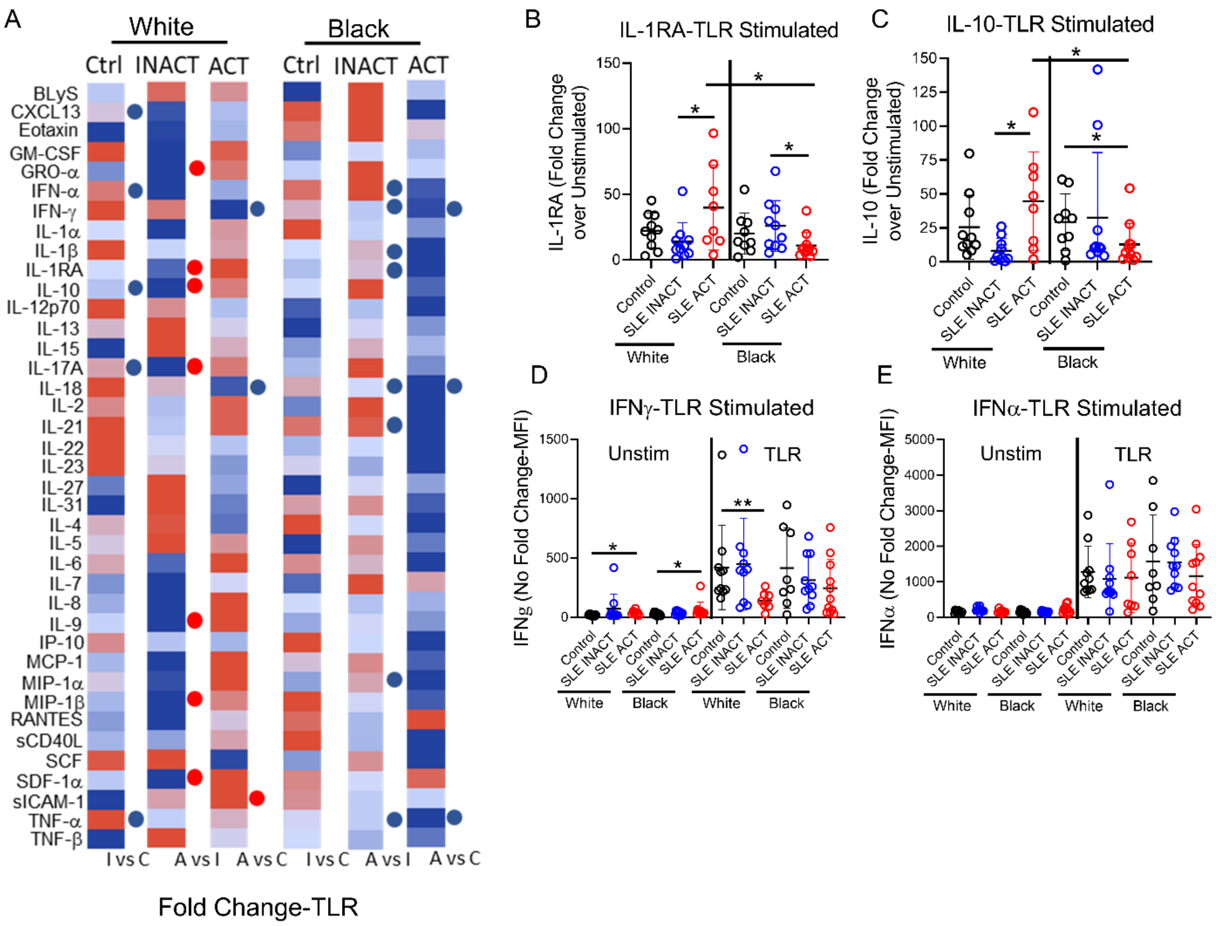

Supplemental Figure 42. Fold change in cytokine levels following TLR stimulation. Whole blood was stimulated for 24 hours with TLR4/7/8/9 agonists. Soluble mediators were measured in culture supernatants using multiplex and ELISA assays. (A) A heatmap summary of the fold change in cytokine levels by ancestry and disease group is shown. Significant differences ($p<0.05$) between disease groups are noted with a red dot (significantly increased) or blue dot (significant decrease). White SLE ACT patients were distinguished by significantly greater fold change differences in (B) IL-1RA and (C) IL-10 following TLR stimulation. Raw cytokine levels of (D) IFNγ and (E) IFNα in unstimulated and TLR stimulated culture supernatants show increased expression following stimulation but an inability of SLE ACT patients to make as much type I or II IFN cytokine as healthy controls. *$p<0.05$, **$p<0.01$, by Kruskal-Wallis test with two-tailed Mann-Whitney for multiple comparisons. Mean± SD shown.

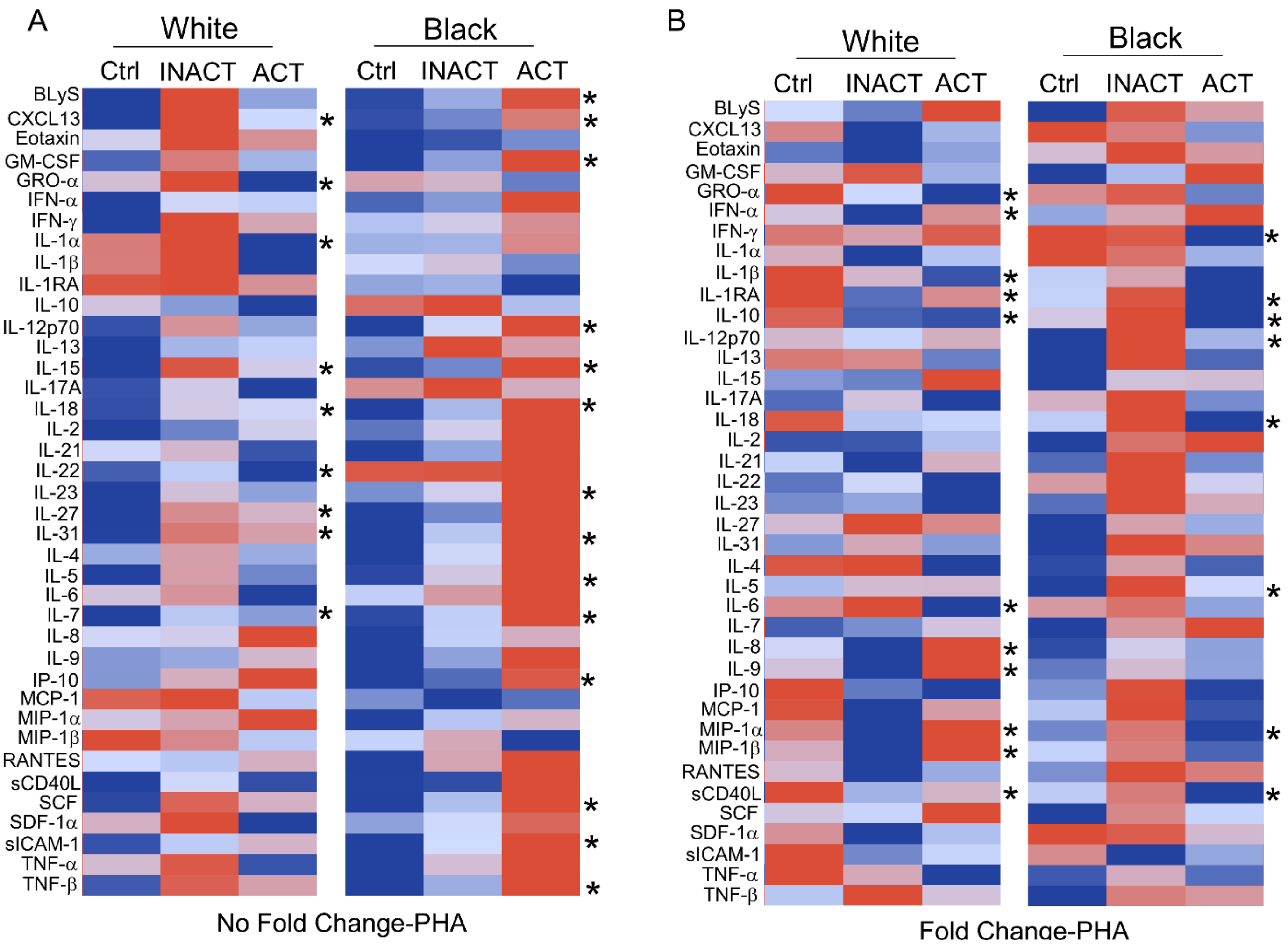

Supplemental Figure 43. Raw level and fold change cytokine levels following PHA stimulation. Whole blood was stimulated for 24 hours with PHA. Soluble mediators were measured in culture supernatants using multiplex and ELISA assays. Heat map summaries of (A) the total cytokine levels and (B) fold change in cytokine levels by ancestry and disease group are shown. Significant differences (p<0.05) between White and Black controls and disease groups are noted with an asterisk. Stastical significance assessed using Kruskal-Wallis test.

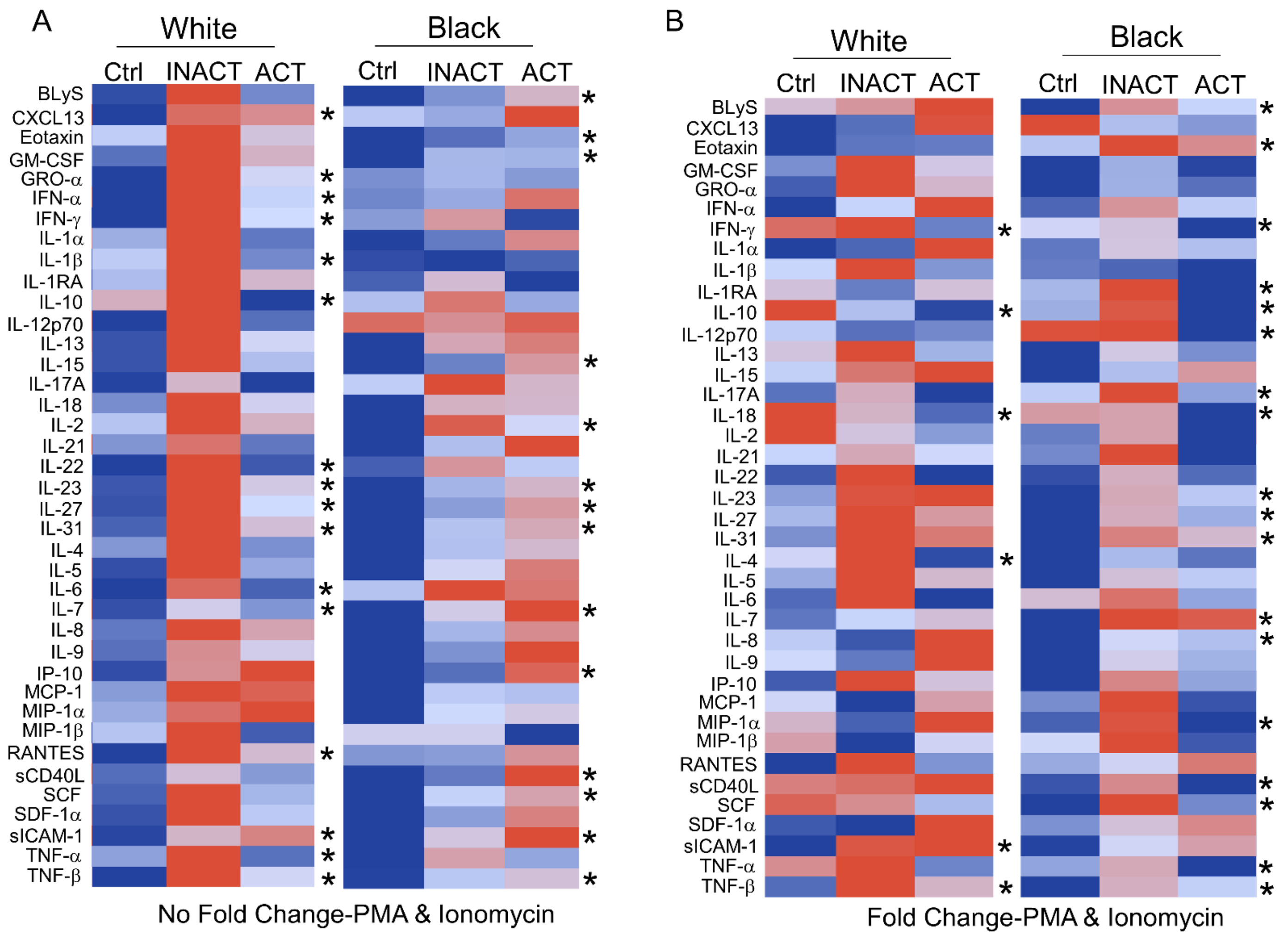

Supplemental Figure 44. Raw level and fold change cytokine levels following PMA and ionomycin stimulation. Whole blood was stimulated for 24 hours with PMA/ionomycin. Soluble mediators were measured in culture supernatants using multiplex and ELISA assays. (A) Heat map summaries of (A) the total cytokine levels and (B) fold change in cytokine levels by ancestry and disease group. Significant differences ($p<0.05$) between White and Black controls and disease groups are noted with asterisk. Statistical significance assessed using Kruskal-Wallis test.

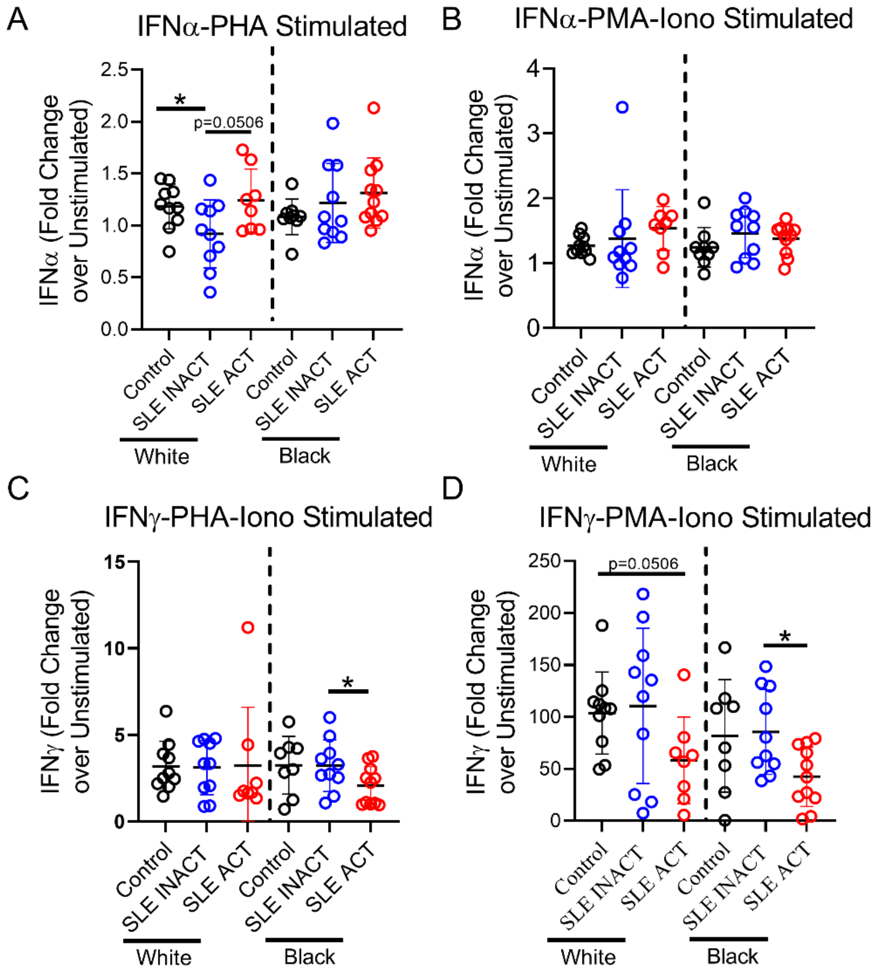

Supplemental Figure 45. Fold change in type I and II IFN levels following PHA or PMA/ionomycin stimulation. Whole blood was stimulated for 24 hours with PHA or PMA/ionomycin. Soluble mediators were measured in culture supernatants using multiplex and ELISA assays and fold change values were calculated over unstimulated culture supernatants for IFNα following (A) PHA and (B) PMA/ionomycin and for IFNγ following (C) PHA and (D) PMA-ionomycin. *p<0.05, by Kruskal-Wallis test with two-tailed Mann-Whitney for multiple comparisons. Mean± SD shown.

Supplemental Table 1. Age, Medication Use, and Disease Activity of Study Participants

Race (n)	White (n=29)				Black (n=29)			
Groups (n)	Control (n=10)	SLE INACT (n=10)	SLE ACT (n=9)	p-value	Control (n=8)	SLE INACT (n=10)	SLE ACT (n=11)	p-value
**Age (Mean±SD)**	45.9 (13.5)	45.7 (15.2)	47.4 (13.7)	0.9	45.4 (11.3)	40.8 (15.2)	36.0 (12.4)	0.2
**Medication**								
Anti-malarials	-	6 (60%)	6 (67%)	-	-	7 (70%)	7 (64%)	-
Steroids	-	3 (30%)	5 (56%)	-	-	4 (40%)	2 (18%)	-
Immunosuppressants	-	5 (50%)	4 (44%)	-	-	5 (50%)	4 (36%)	-
Biologics	-	1 (10%)	2 (22%)	-	-	0 (0%)	0 (0%)	-
**Mean SLEDAI (Range)**	-	1.2 (0-3)	6.3 (4-10)	-	-	1.3 (0-2)	7.4 (4-11)	-

Supplemental Table 2. SLE patient active SLEDAI criteria at time of study

	White		Black		p-value
**Clinical Criteria**	**SLE-INACT (n=10)**	**SLE-ACT (n=9)**	**SLE-INACT (n=10)**	**SLE-ACT (n=11)**	**Black vs White**
**Arthritis**	None	6 (67%)	None	5 (50%)	0.727
**Proteinuria**	None	1 (11%)	None	3 (30%)	0.607
**Pyuria**	None	None	None	3 (30%)	0.233
**Rash**	None	3 (33%)	1 (10%)	3 (30%)	>0.999
**Alopecia**	None	2 (22%)	2 (20%)	3 (30%)	0.413
**Mucusol Ulcers**	None	1 (11%)	1 (10%)	1 (10%)	>0.999
**Low Complement**	4 (40%)	5 (56%)	1 (10%)	7 (70%)	0.75
**Increased DNA binding**	2 (20%)	3 (33%)	2 (20%)	6 (60%)	0.495
**Leukopenia**	1 (10%)	None	None	3 (30%)	0.607
**Lymphopenia**	1 (10%)	3 (33%)	1 (10%)	5 (50%)	0.721

Supplemental Table 3. Autoantibody specificity by SLE disease activity.

	White			Black			p-value	p-value	p-value
**Antibody Specificity**	**SLE-INACT (n=10)**	**SLE-ACT (n=9)**	**p-value**	**SLE-INACT (n=10)**	**SLE-ACT (n=11)**	**p-value**	**White SLE-INACT vs Black SLE-INACT**	**White SLE-ACT vs Black SLE-ACT**	**White SLE vs Black SLE**
dsDNA	6 (60%)	6 (67%)	>0.999	3 (30%)	8 (64%)	0.086	0.370	>0.999	0.538
Chromatin	3 (30%)	4 (44%)	0.650	2 (20%)	7 (64%)	0.085	>0.999	0.653	0.755
RNP	2 (20%)	3 (33%)	0.629	4 (40%)	7 (64%)	0.395	0.629	0.370	0.117
Sm	1 (10%)	3 (33%)	0.303	1 (10%)	5 (45%)	0.149	>0.999	0.670	0.721
SmRNP	2 (20%)	4 (44%)	0.350	6 (60%)	7 (64%)	>0.999	0.170	0.653	0.067
Ro	5 (50%)	5 (55%)	>0.999	3 (30%)	2 (18%)	0.635	0.650	0.160	0.102
La	0 (0%)	3 (33%)	0.087	1 (10%)	0 (0%)	0.476	>0.999	0.074	0.331
Ribo P	1 (10%)	2 (22%)	0.582	0 (0%)	3 (27%)	0.214	>0.999	>0.999	>0.999
Centromere B	0	0	-	0	0	-	-	-	-
Scl-70	0	0	-	0	0	-	-	-	-
Jo-1	0	0	-	0	0	-	-	-	-

Supplemental Table 4. Antibodies and elemental isotope tags.

Antigen Target (Human)	Clone	Supplier	Elemental Isotope
cCasp3	D3E9	Fluidigm	142Nd
CD11b	ICRF44	Fluidigm	144Nd
CD11c	Bu15	Fluidigm	159Tb
CD123 (IL-3R)	6H6	Fluidigm	151Eu
CD127 (IL-7Ra)	A019D5	Fluidigm	176Yb
CD14	M5E2	Fluidigm	160Gd
CD141 (BDCA-3)	AD514H12	Miltenyi	161Dy
CD16	B73.1	Biolegend	166Er
CD19	HIB19	Biolegend	143Nd
CD20	2H7	Fluidigm	147Sm
CD24	ML5	BD	157Gd
CD25	M-A251, BD	BD	164Dy
CD27	L128	BD	152Sm
CD3	UCHT1	Fluidigm	170Er
CD38	HIT2	Fluidigm	167Er
CD4	RPA-T4	Fluidigm	145Nd
CD45	HI30	Fluidigm	154Sm
CD45RA	H100	Fluidigm	153Eu
CD56	HCD56	Biolegend	175Lu
CD66a	CD66a-B1.1	Fluidigm	171Yb
CD7	CD7-687	Biolegend	149Sm
CD8a	SK1	Fluidigm	168Er
HLA-DR	L243	Fluidigm	174Yb
IgA	Polyclonal	Fluidigm	148Nd
IgD	IA6-2	Fluidigm	146Nd
pCREB [S133]	87G3	Fluidigm	165Ho
pERK1/2	20A, BD	BD	162Dy
p-p38 [T180/Y182]	D3F9	Fluidigm	156Gd
pPLCg2	K86-68937	BD	173Yb
pSTAT1	4a	BD	169Tm
pStat3 [Y705]	4	Fluidigm	158Gd
pSTAT5	47/Stat5(pY694)	BD	172Yb
Syk	4D10.2	Biolegend	150Nd

Table 5. Antibodies and elemental isotope tags.

Antigen Target (Human)	Clone Number	Supplier	Elemental Isotope
CCR6	IA9	BD	Gd155
CCR7	150503	R&D Systems	Gd160
CD11b	ICRF44	BioLegend	Eu153
CD11c	Bu15	BioLegend	Nd148
CD123	9F5	BD	Yb173
CD127	A019D5	BioLegend	Ho165
CD14	M5E2	BioLegend	Sm154
CD16	3G8	BioLegend	Sm149
CD161	DX12	BD	Er170
CD19	SJ25C1	Southern Biotech	Nd142
CD20	2H7	BioLegend	Dy164
CD24	ML5	BioLegend	Er168
CD25	M-A251	BD	Yb176
CD27	L128	BD	Sm152
CD28	L293	BD	Er167
CD3	UCHT1	BD	Nd150
CD33	P67.8	BD	Er166
CD38	HB-7	BD	Eu151
CD4	SK3	BioLegend	Nd143
CD45RA	HI100	BioLegend	Dy162
CD56	NCAM16.2	BD	Yb174
CD57	HCD57	BioLegend	In113
CD8	SK1	BioLegend	Nd144
CD85j	292319	R&D Systems	Sm147
CD86	IT2.2	BioLegend	Gd157
CD94	HP-3D9	BD	Gd156
CXCR3	G025H7	BioLegend	Tb159
CXCR5	RF8B2	BD	Gd158
HLADR	G46-6	BD	Lu175
ICOS	DX29	BD	Tm169
IgD	IA6-2	BioLegend	Nd146
PD-1	EH12.1	BD	Yb172
TCRγδ	B1	BioLegend	Yb171

Supplemental Table 6. Demographics of Study Participants by Disease Group

Race (n)	White (n=38)				Black (n=27)			
Groups (n)	ANA- (n=18)	SLE INACT (n=13)	SLE ACT (n=7)	p-value	ANA- (n=14)	SLE INACT (n=6)	SLE (n=7)	p-value
Age (Mean±SD)	40.5 (14.6)	49.0 (10.3)	43.0 (12.2)	0.7	41.5 (10.5)	44.5 (10.0)	47.0 (12.2)	0.7
Sex (Female)	100%	100%	100%	-	93%	100%	100%	-

Supplemental Table 7. Autoantibody specificities and ACR criteria of SLE patients

White (n=19)		Black (n=21)	
**Autoantibodies**	**%**	**Autoantibodies**	**%**
dsDNA	5.0%	dsDNA	15.4%
Chromatin	5.0%	Chromatin	15.3%
Ro	25.0%	Ro	15.4%
La	0.0%	La	7.7%
Sm	5.0%	Sm	23.1%
RNP	5.0%	RNP	30.8%
sm/RNP	5.0%	sm/RNP	30.8%
RiboP	0.0%	RiboP	0.0%
Centromere B	10.0%	Centromere B	0.0%
Scl-70	0.0%	Scl-70	0.0%
Jo-1	0.0%	Jo-1	0.0%
**ACR Classification Criteria**		**ACR Classification Criteria**	
Arthritis	90.0%	Arthritis	92.3%
Oral Ulcers	90.0%	Oral Ulcers	69.2%
Malar Rash	70.0%	Malar Rash	61.5%
Photosensitivity	80.0%	Photosensitivity	38.4%
Immunologic Criteria	25.0%	Immunologic Criteria	53.8%
Serositis	40.0%	Serositis	46.2%
Leukopenia	15.0%	Leukopenia	23.1%
Lymphopenia	5.0%	Lymphopenia	53.8%
Renal Disease	5.0%	Renal Disease	61.5%
CNS Disease	20.0%	CNS Disease	15.4%
Discoid Rash	5.0%	Discoid Rash	30.8%
Hemolytic Anemia	5.0%	Hemolytic Anemia	15.4%
Thrombocytopenia	0.0%	Thrombocytopenia	7.7%
**Mean SLEDAI (range)**	4.3 (0-10)	**Mean SLEDAI (range)**	4.1 (0-12)
**Median SLEDAI**	4	**Median SLEDAI**	4

Supplemental Table 8. Cell subset frequencies as determined by bivariate gating (n=91).

		White Median Frequency (IQR)					Black Median Frequency (IQR)				
**Cell Subset**	**Cell Surface Designation**	**Control**	**SLE INACT**	**SLE ACT**	**KW p-value**	**FDR q-value**	**Control**	**SLE INACT**	**SLE ACT**	**KW p-value**	**FDR q-value**
Granulocytes	CD66a+CD14-CD3-CD19-	66.17 (13.86)	74.00 (6.91)	70.90 (21.90)	0.258	0.476	56.38 (19.18)	57.92 (9.62)	63.79 (18.12)	0.622	0.712
B Cells	CD66a-CD3-CD19+	2.10 (1.19)	2.06 (2.23)	1.41 (0.91)	0.073	0.267	4.83 (4.13)	3.34 (3.76)	5.07 (4.58)	0.555	0.670
Non-switched Memory B Cells	CD66a-CD3-CD19+IgD+CD27+	0.10 (0.22)	0.12 (0.20)	0.12 (0.13)	0.755	0.793	0.18 (0.28)	0.14 (0.14)	0.23 (0.43)	0.550	0.670
Switched Memory B Cells	CD66a-CD3-CD19+IgD-CD27+CD38-	0.10 (0.05)	0.06 (0.09)	0.06 (0.13)	0.454	0.577	0.17 (0.12)	0.13 (0.13)	0.11 (0.23)	0.538	0.670
Plasmablasts	CD66a-CD3-CD19+IgD-CD27+CD38+	0.16 (0.10)	0.11 (0.10)	0.08 (0.10)	0.315	0.476	0.27 (0.27)	0.20 (0.19)	0.21 (0.58)	0.508	0.670
IgD-CD27- DN B cells	CD66a-CD3-CD19+IgD-CD27-	0.40 (0.40)	0.26 (0.19)	0.24 (0.15)	0.023	0.184	0.62 (0.35)	0.32 (0.36)	0.69 (0.77)	0.292	0.670
DN1/DN3 B cells	CD66a-CD3-CD19+IgD-CD27-CD11c-	0.51 (0.29)	0.22 (0.32)	0.52 (0.20)	0.078	0.342	0.31 (0.33)	0.26 (0.16)	0.082 (0.27)	0.559	0.922
DN2 B cells	CD66a-CD3-CD19+IgD-CD27-CD11c+	0.08 (0.08)	0.10 (0.07)	0.20 (0.29)	0.916	1	0.08 (0.06)	0.06 (0.06)	0.06 (0.10)	0.456	0.922
Naïve B Cells	CD66a-CD3-CD19+IgD+CD27-CD24±CD38±	1.98 (0.93)	1.19 (1.13)	0.86 (0.88)	0.011	0.181	4.17 (3.72)	2.29 (3.22)	3.67 (3.80)	0.402	0.670
Resting Naïve B cells	CD66a-CD3-CD19+IgD+CD27-CD24±CD38±CD11c-	1.95 (0.96)	1.48 (1.90)	0.60 (0.96)	0.053	0.342	4.06 (3.42)	4.65 (2.72)	3.27 (3.55)	0.979	1
Active Naïve B cells	CD66a-CD3-CD19+IgD+CD27-CD24±CD38±CD11c+	0.03 (0.05)	0.05 (0.01)	0.01 (0.02)	0.230	0.612	0.08 (0.12)	0.26 (0.42)	0.06 (0.07)	0.628	0.922
Transitional B Cells	CD66a-CD3-CD19+IgD+CD27-CD24^{hi}CD38^{hi}	0.08 (0.09)	0.03 (0.09)	0.02 (0.05)	0.303	0.476	0.30 (0.21)	0.09 (0.63)	0.09 (0.47)	0.649	0.712
Memory B cells/Plasmablasts	CD66a-CD3-CD19+IgD-CD27+	0.27 (0.10)	0.16 (0.19)	0.13 (0.22)	0.336	0.477	0.47 (0.43)	0.36 (0.25)	0.41 (0.77)	0.440	0.670

T Cells	CD66a-CD3+CD56-	21.84 (7.41)	14.27 (5.55)	16.31 (15.64)	0.176	0.472	26.77 (16.20)	29.81 (14.41)	20.53 (14.34)	0.459	0.670
CD4+ T Cells	CD66a-CD3+CD56-CD4+CD8-	14.84 (7.37)	9.82 (5.72)	11.16 (8.38)	0.102	0.308	15.99 (10.03)	17.34 (11.09)	12.35 (10.03)	0.363	0.670
CD8+ T Cells	CD66a-CD3+CD56-CD4-CD8+	5.01 (3.17)	3.63 (1.91)	3.69 (4.53)	0.507	0.612	9.21 (4.39)	8.34 (5.60)	3.91 (3.87)	0.038	0.202
CD4+ Naïve/Effector T Cells	CD66a-CD3+CD56-CD4+CD45RA+	6.50 (2.98)	2.90 (3.22)	3.61 (4.13)	0.052	0.251	5.72 (4.06)	8.13 (6.97)	3.34 (4.39)	0.162	0.435
CD8+ Naïve/Effector T Cells	CD66a-CD3+CD56-CD8+CD45RA+	2.68 (1.12)	1.89 (1.37)	1.90 (2.89)	0.283	0.476	5.19 (3.50)	5.08 (4.49)	2.64 (3.57)	0.044	0.202
CD4+ Memory T Cells	CD66a-CD3+CD56-CD4+CD45RA-	7.24 (2.36)	5.69 (4.93)	6.38 (6.12)	0.367	0.492	8.78 (6.35)	9.53 (6.25)	8.41 (5.18)	0.840	0.882
CD8+ Memory T Cells	CD66a-CD3+CD56-CD8+CD45RA-	1.88 (1.63)	1.56 (0.93)	1.00 (2.24)	0.617	0.677	2.04 (1.30)	1.95 (1.54)	1.38 (1.25)	0.378	0.670
NK Cells	CD66a-CD3-CD19-CD56+	0.45 (0.48)	0.93 (0.81)	0.72 (0.51)	0.246	0.476	0.59 (0.52)	0.60 (1.28)	0.28 (0.25)	0.050	0.202
Monocytes	HLA-DR+CD11c+CD14+CD16±CD3-CD19-CD56-	5.07 (2.11)	3.81 (2.74)	3.73 (2.39)	0.217	0.476	6.47 (2.27)	3.96 (2.07)	3.38 (3.45)	0.028	0.202
Classical Monocytes	HLA-DR+CD11c+CD14+CD16-CD3-CD19-CD56-	4.30 (1.71)	3.14 (2.16)	2.99 (2.29)	0.238	0.476	3.97 (1.45)	3.28 (1.24)	2.88 (2.37)	0.071	0.245
Intermediate Monocytes	HLA-DR+CD11c+CD14^{lo}CD16+CD3-CD19-CD56-	0.08 (0.09)	0.03 (0.14)	0.03 (0.15)	0.603	0.677	0.18 (0.13)	0.07 (0.15)	0.06 (0.18)	0.148	0.435
Non-classical Monocytes	HLA-DR+CD11c+CD14+CD16+CD3-CD19-CD56-	0.50 (0.49)	0.35 (0.39)	0.14 (0.15)	0.048	0.251	1.05 (0.79)	0.46 (0.62)	0.10 (0.36)	0.003	0.084
Basophils	CD66a+HLA-DR-CD11c-CD123+	0.56 (0.38)	0.31 (0.61)	0.15 (0.33)	0.015	0.181	0.64 (0.29)	0.32 (0.56)	0.16 (0.31)	0.017	0.202

DCs	HLA-DR+CD11c+CD14-CD16-CD3-CD19-CD56-	0.22 (0.15)	0.50 (0.74)	0.50 (0.45)	0.078	0.267	0.22 (0.17)	0.33 (0.30)	0.33 (0.45)	0.434	0.670
pDCs	HLA-DR+CD11c-CD123+CD3-CD19-CD56-	0.12 (0.09)	0.09 (0.10)	0.03 (0.10)	0.450	0.915	0.08 (0.086)	0.08 (0.14)	0.06 (0.05)	0.529	0.8549

Supplemental Table 9. Statistical significance of cell frequency comparisons by bivariate gating (FDR)

Cell Subset	White			Black					
	Ctrl vs SLE INACT	Ctrl vs SLE ACT	SLE INACT vs SLE ACT	Ctrl vs SLE INACT	Ctrl vs SLE ACT	SLE INACT vs SLE ACT	White Ctrl vs Black Ctrl	White SLE INACT vs Black SLE INACT	White SLE ACT vs Black SLE ACT
Neutrophils	ns	ns	ns	ns	ns	ns	ns	**<0.01**	ns
B Cells	ns	ns	ns	ns	ns	ns	ns	ns	**<0.05**
Non-switched Memory B Cells	ns	ns	ns	ns	ns	ns	ns	ns	ns
Switched Memory B Cells	ns	ns	ns	ns	ns	ns	ns	ns	ns
Plasmablasts	ns	ns	ns	ns	ns	ns	ns	ns	ns
IgD-CD27- DN B cells	ns	ns	ns	ns	ns	ns	ns	ns	**<0.05**
DN1/DN3 B cells	ns	ns	ns	ns	ns	ns	ns	ns	**<0.05**
DN2 B cells	ns	ns	ns	ns	ns	ns	ns	ns	ns
Naïve B Cells	ns	**<0.05**	ns	ns	ns	ns	ns	ns	**<0.05**
Resting Naïve B cells	ns	**<0.05**	ns	ns	ns	ns	ns	ns	**<0.05**
Active Naïve B cells	ns	ns	ns	ns	ns	ns	ns	ns	**<0.05**
Transitional B Cells	ns	ns	ns	ns	ns	ns	ns	ns	ns
Memory Cells and Plasmablasts	ns	ns	ns	ns	ns	ns	ns	ns	**<0.05**
T Cells	ns	ns	ns	ns	ns	ns	ns	**<0.01**	ns
CD4+ T Cells	ns	ns	ns	ns	ns	ns	ns	ns	ns
CD8+ T Cells	ns	ns	ns	ns	ns	ns	ns	**<0.01**	ns
CD4+ Naïve/Effector T Cells	ns	ns	ns	ns	ns	ns	ns	ns	ns
CD4+ Memory T Cells	ns	ns	ns	ns	ns	ns	ns	ns	ns
CD8+ Naïve/Effector T Cells	ns	ns	ns	ns	ns	ns	ns	**<0.01**	ns
CD8+ Memory T Cells	ns	ns	ns	ns	ns	ns	ns	ns	ns
NK Cells	ns	ns	ns	ns	ns	ns	ns	ns	ns
Monocytes	ns	ns	ns	ns	ns	ns	ns	ns	ns

Classical Monocytes	ns	ns	ns	ns	ns	ns	ns	ns	ns
Intermediate Monocytes	ns	ns	ns	ns	ns	ns	ns	ns	ns
Non-classical Monocytes	ns	ns	ns	ns	**<0.05**	ns	ns	ns	ns
Basophils	ns	**<0.05**	ns	ns	**<0.05**	ns	ns	ns	ns
DCs	ns	ns	ns	ns	ns	ns	ns	ns	ns
pDCs	ns	ns	ns	ns	ns	ns	ns	ns	ns

Supplemental Table 10. Cell subset activation marker frequency differences.

	Median Frequency (Interquartile Range)					Median Frequency (Interquartile Range)				
	**White Controls**	**White SLE INACT**	**White SLE ACT**	**KW p-value**	**FDR q-value**	**Black Controls**	**Black SLE INACT**	**Black SLE ACT**	**KW p-value**	**FDR q-value**
**Granulocytes**										
**CD38+**	14.9 (10.3-23)	16.9 (11.4-32.8)	22.3 (11.4-40)	0.8030	0.9732	17.6 (8.98-32.2)	26.6 (17.2-41.3)	32.8 (25.3-57.9)	0.1100	0.4051
**CD11c+**	95.4 (81.1-98.6)	88.1 (73.2-94.9)	94 (90-95.9)	0.5150	0.8555	98.9 (98.1-99.1)	97.9 (96.7-99)	97.2 (94.2-97.9)	0.2090	0.5496
**B Cells**										
**CD38+**	42.4 (39-58.3)	57.5 (40.4-67.8)	84.1 (40.2-87.6)	0.3460	0.8044	63.4 (52-67)	60.9 (47.5-72.3)	54 (47.9-59.9)	0.6670	0.9125
**CD11c+**	7.07 (4.27-13.3)	5.64 (4.05-15.3)	8.27 (5.92-16.1)	0.3920	0.8281	6.62 (4.45-9.34)	7.42 (2.97-9.21)	13.1 (3.62-24.5)	0.4900	0.8862
**CD11b+**	42 (32.3-65.4)	35.2 (28.5-57.4)	59.5 (34.4-73.7)	0.6640	0.9563	32.1 (14-46.1)	31.7 (17.3-62.7)	35.6 (25.3-75.5)	0.5370	0.9125
**CD27+**	56.7 (43.7-73.4)	67.7 (47-79.3)	65.3 (41.9-90.2)	0.9460	0.9809	56.3 (52.6-80.7)	73.1 (48.3-84.3)	78.4 (55.7-89.1)	0.5850	0.9125
**Non-switched Memory B Cells**										
**CD38+**	28.2 (16.8-49.5)	49.1 (35.6-74.7)	44.4 (33.3-88.4)	0.2550	0.7802	44.3 (37.5-59.8)	54.4 (50.2-82.6)	73.4 (51.5-85.3)	0.1580	0.4724
**CD11c+**	11 (2.93-14.3)	17 (5.67-26.3)	6.67 (2.15-11.1)	0.1340	0.7444	11.1 (7.92-13.2)	14.8 (8-18.1)	17.6 (4.17-26)	0.8160	0.9321
**CD11b+**	43.2 (28.1-70)	52.5 (47.8-85.9)	64.6 (36.4-77.8)	0.4290	0.8555	46.1 (23.4-60.3)	45.2 (34.6-65.4)	55.8 (40-84.1)	0.5540	0.9125
**Memory B Cells/Plasmablasts**										
**CD11c+**	14.1 (6.58-18.6)	21.6 (7.01-31.3)	6.9 (4-21.6)	0.3550	0.8044	22.8 (18.3-26.4)	23.9 (15.7-28.3)	29.4 (16-32.3)	0.6630	0.9125
**CD11b+**	57.4 (47-81.8)	56.8 (46.3-85.8)	47.5 (36-73.3)	0.7780	0.9732	50.8 (36.4-66.2)	53.2 (36.5-82.1)	56.9 (38.1-83.8)	0.7840	0.9321
**DN B Cells**										
**CD38+**	36.5 (32.9-41)	42 (32.2-58.6)	73.3 (39.1-87.5)	0.0878	0.7259	41 (29.6-49.1)	45.8 (32.9-49.5)	44.7 (26.6-48.6)	0.8660	0.9354

**CD11c+**	13.7 (9.24-22)	25.4 (17-28.9)	27.6 (13.1-74.4)	0.1870	0.7802	15.1 (12.2-19.1)	25 (17-35.9)	16.5 (11-43.8)	0.2350	0.5711
**CD11b+**	50.7 (41-53.1)	45.1 (28.9-69.2)	73.1 (52.6-77)	0.2900	0.7802	40.2 (21.7-62.7)	40.2 (26.3-67.1)	41.4 (22-79.8)	0.8620	0.9354
**Naïve B Cells**										
**CD38+**	54.9 (42.8-61.5)	62 (45.1-78.8)	71.4 (42.9-86.2)	0.4790	0.8555	70 (58-76.5)	66.4 (61.3-78.5)	53.5 (46-62.9)	0.2300	0.5711
**CD11c+**	1.82 (0.545-2.96)	2.45 (1.24-4)	2 (0-3.15)	0.7350	0.9732	1.62 (0.673-2.75)	0.858 (0.545-3.04)	3.64 (0.46-12.3)	0.4790	0.8862
**CD11b+**	37.5 (19-65.7)	26.6 (20.7-54.7)	42.9 (28.6-69)	0.7720	0.9732	24.7 (10.9-37.1)	24.2 (13.7-55.8)	24 (18.3-72.4)	0.5870	0.9125
**Transitional B Cells**										
**CD11c+**	0 (0-3.57)	0.304 (0-4.99)	0 (0-2.14)	0.8690	0.9732	1.85 (0.169-2.43)	0.139 (0-1.2)	0.772 (0-1.13)	0.3990	0.7976
**CD11b+**	39.5 (23.1-45.4)	47.5 (27.4-79)	23.5 (0-60)	0.3100	0.7802	23.3 (16.4-44.7)	23.2 (19.4-42.9)	42.9 (26.7-70.4)	0.2850	0.6519
**T Cells**										
**CD38+**	34.2 (30.2-38.2)	25.7 (20.6-30.4)	25.7 (22.8-42.7)	0.2230	0.7802	30.3 (26.5-36.9)	31 (23.5-47.1)	39 (27.5-50)	0.5250	0.9125
**HLA-DR+**	3.66 (2.98-4.31)	5.25 (4.07-8.14)	13.5 (4.52-16.1)	0.1440	0.7466	3.93 (3.49-4.86)	4.72 (3.58-7.99)	10.3 (5.44-17)	0.0502	0.2415
**CD11b+**	42.6 (30.4-49.7)	40.9 (32.1-62.6)	49.1 (35.7-59.1)	0.8900	0.9732	28 (21.1-42.3)	29.4 (22.1-45.2)	40.9 (21.2-61.6)	0.7740	0.9321
**CD27+**	91.1 (84.3-93.2)	88 (77.2-91.9)	83.4 (83.2-90.2)	0.5580	0.8722	90.7 (84.2-95.1)	89.9 (87-93.8)	89.2 (85.5-94.3)	0.9560	0.9783
**CD38+HLA-DR+**	0.974 (0.869-1.42)	2.05 (1.16-2.76)	4.19 (1.35-9.03)	0.0721	0.7259	1.42 (1.04-1.61)	2.02 (1.25-2.31)	7.25 (2.2-12.3)	**0.0031**	**0.0482**
**NKT Cells**										
**CD38+**	31.3 (26.3-34.5)	37.5 (31.3-47.5)	45.9 (31.3-85.7)	0.2080	0.7802	22.6 (18.6-32.7)	37.5 (25.3-45.9)	45.9 (41.7-63.6)	**0.0122**	0.121
**HLA-DR+**	12.8 (7.32-23.5)	16.4 (8.25-24.1)	32.8 (7.81-48.9)	0.2530	0.7802	6.82 (4.95-16.8)	13.5 (9.37-33.7)	37.5 (15.5-42.2)	**0.0291**	0.1886
**CD11c+**	7.19 (4.37-13.5)	8.02 (4.33-20.5)	22.4 (16.4-28.6)	0.0638	0.7259	8.97 (3.95-21.7)	6.72 (4.3-17)	12 (4.05-18.9)	0.8290	0.9321

**CD11b+**	69.8 (47.5-75.5)	69 (59.5-90.9)	66.7 (39.3-77.6)	0.7520	0.9732	74.3 (59.4-82)	53 (46.7-76.7)	62.9 (44.8-85.4)	0.4000	0.7976
**CD27+**	65.2 (51.7-75.3)	61.7 (34.4-67.6)	62.5 (53.4-66.7)	0.8670	0.9732	70.8 (34.2-82.7)	67 (54.4-85.1)	76 (63.7-84.1)	0.8290	0.9321
**CD4+ T Cells**										
**CD38+**	41 (36.9-44.3)	25.6 (23-31)	28.8 (26-41.4)	**0.0296**	0.7259	34.4 (30.3-38.1)	31.6 (29.6-48)	32.3 (30.5-41)	0.9890	0.9989
**HLA-DR+**	2.98 (2.57-4.16)	4.03 (3.18-5.15)	5.75 (4.38-11.4)	0.0954	0.7259	3.07 (2.66-3.71)	3.8 (2.99-5.57)	7.45 (4.18-8.44)	**0.0405**	0.225
**CD11b+**	38.5 (27.9-45.3)	36.9 (28.8-61.3)	51.5 (27.1-60.6)	0.8620	0.9732	31.6 (17.4-43.4)	29.8 (17.4-46.2)	42.3 (19.3-65.3)	0.6870	0.9125
**CD27+**	94.2 (90.1-95.3)	90.1 (87.3-92.3)	90.1 (88.8-92.1)	0.1280	0.7444	92.3 (89.2-95.1)	92.9 (89.6-94.8)	93.8 (92.5-94.7)	0.6300	0.9125
**CD38+HLA-DR+**	0.904 (0.57-1)	1.15 (0.566-1.92)	1.54 (1.38-5.63)	**0.0275**	0.7259	0.878 (0.762-1.1)	1.29 (0.676-1.7)	2.41 (1.17-5.47)	**0.0113**	0.121
**CD8+ T Cells**										
**CD38+**	17.3 (13.4-22)	22.5 (21.9-26.3)	21.3 (13.2-44.6)	0.4490	0.8555	25.2 (21.1-35.3)	25.2 (16.6-51.9)	45.7 (25.7-56.7)	0.1740	0.4833
**HLA-DR+**	4.73 (3.99-7.5)	8.68 (4.41-16.3)	17.1 (3.81-24)	0.5720	0.8722	5.17 (3.93-6.93)	5.74 (3.95-10.4)	13.8 (7.47-23.8)	**0.0335**	0.2004
**CD11b+**	47.7 (33.5-57.9)	41.8 (31.8-65.9)	52.1 (42.6-55.6)	0.9260	0.9732	24.3 (19.1-40.1)	30.3 (26.6-40.6)	39.2 (33-56.7)	0.2670	0.6292
**CD27+**	81.7 (71.2-90.6)	83.6 (64.5-95.4)	77.4 (69.2-87.3)	0.9200	0.9732	91.3 (83.9-95.7)	86.2 (77.3-95.3)	84 (72.9-93.1)	0.6360	0.9125
**CD38+HLA-DR+**	1.77 (1.15-3.02)	3.19 (1.89-5.94)	8.31 (1.25-13.3)	0.5650	0.8722	2 (1.14-2.52)	2.67 (2.24-3.02)	9.16 (4.44-17.9)	**0.0013**	**0.0369**
**DN T Cells**										
**CD38+**	12.7 (9.22-17.7)	20.4 (12.5-34.8)	26.8 (19.7-34.6)	0.1120	0.7259	15.7 (11.4-17.1)	23.4 (19.9-27)	41.3 (29.3-54.3)	**0.0017**	**0.0369**
**HLA-DR+**	5.04 (3.39-7.94)	7.05 (5.24-17.3)	10.7 (8.51-22.7)	0.2100	0.7802	7.04 (5.01-8.47)	5.24 (3.61-8.99)	17.1 (7.45-22.6)	0.1060	0.4051
**CD11b+**	71.1 (62.2-76.7)	59.4 (45.4-61.2)	62 (43.4-75.8)	0.3430	0.8044	42.8 (40.4-65.7)	41.6 (32-59)	40.7 (26.5-66)	0.6950	0.9125

**CD27+**	76.1 (65.5-80.7)	79.3 (67.4-82.8)	73.2 (69-79.8)	0.8050	0.9732	89.1 (59.5-91.9)	86 (75-95.5)	82.8 (78.8-94.1)	0.6860	0.9125
**CD38+HLA-DR+**	2.03 (0.996-2.6)	3.29 (2.84-8.56)	7.14 (4.61-9.28)	**0.0116**	0.7259	2.79 (1.96-3.83)	3.19 (2.35-4.68)	14.6 (4.82-18)	**0.0192**	0.1357
**CD4+ Naïve/Effector T Cells**										
**CD38+**	71.4 (57.3-73)	55.6 (50.7-58.7)	52.9 (49.5-71.7)	0.3620	0.8044	66.6 (60.8-76.3)	63.3 (51.6-75)	63.1 (51.7-69.8)	0.4680	0.8862
**HLA-DR+**	0.387 (0.205-1.04)	1.02 (0.278-1.97)	0.956 (0.633-1.32)	0.5420	0.8722	0.533 (0.319-0.806)	0.541 (0.317-0.831)	1.44 (0.714-1.55)	**0.0140**	0.121
**CD11b+**	32.8 (23-44)	33.1 (25.1-60.7)	46.7 (20.8-56.7)	0.8930	0.9732	24.1 (14.7-46.3)	26.9 (12.9-40.7)	33.3 (14.5-60.2)	0.7410	0.9295
**CD27+**	99.7 (96.8-99.9)	99.1 (88.8-99.6)	97.6 (93.9-99.4)	0.2910	0.7802	98.6 (93.6-99.8)	99.2 (98.4-99.5)	99.2 (98.3-99.8)	0.7240	0.923
**CD38+HLA-DR+**	0.244 (0.105-0.593)	0.438 (0.0944-0.841)	0.692 (0.579-1.01)	0.5030	0.8555	0.185 (0.104-0.392)	0.259 (0.111-0.643)	0.424 (0.34-1.09)	0.2120	0.5496
**CD4+ Memory T Cells**										
**CD38+**	16.9 (13.2-21.9)	11.7 (9.27-17.1)	16.2 (14.1-35.4)	0.2120	0.7802	14.5 (12.1-16.4)	14.5 (9.19-22.5)	24.1 (16.2-29.9)	0.0524	0.2415
**HLA-DR+**	5.99 (5.21-6.83)	4.3 (4.11-6.98)	12.4 (6.43-17.3)	0.0757	0.7259	4.61 (4.08-5.53)	5.37 (4.57-7.89)	9.07 (5.81-10.6)	0.0601	0.2597
**CD11b+**	43.8 (31.3-46.7)	36.7 (30.2-61.8)	54.6 (29-61.4)	0.8940	0.9732	32.9 (20.3-46)	33 (20.9-49.7)	45.3 (21.9-66.9)	0.7620	0.9321
**CD27+**	89.9 (85.3-91.1)	87.3 (84.4-90.6)	85.7 (83.8-89.1)	0.5090	0.8555	89.7 (85.9-91.8)	86.8 (84.1-92.4)	91.4 (90.1-92.4)	0.3280	0.7086
**CD38+HLA-DR+**	1.34 (1.02-1.8)	1.21 (0.651-2.49)	3.37 (2.07-6.69)	**0.0496**	0.7259	1.24 (1.16-1.33)	1.52 (1.09-2.47)	3.24 (1.62-6.75)	**0.0128**	0.121
**CD8+ Naïve/Effector T Cells**										
**CD38+**	23.8 (16.5-29.6)	27.8 (23.2-30)	32.9 (13.9-41.9)	0.8270	0.9732	30.1 (27.3-45.1)	27.7 (16.1-57.2)	45.6 (27.1-56.3)	0.4270	0.8302
**HLA-DR+**	2.53 (1.83-4.01)	3.62 (1.73-12.5)	6.67 (2.86-14.4)	0.5090	0.8555	2.51 (1.75-4.18)	4.19 (1.64-7.07)	5.86 (4.69-11.9)	0.1370	0.4262

**CD11b+**	51.4 (31.7-55.2)	40.8 (29.5-70.7)	56.2 (41.9-60.6)	0.8500	0.9732	22.5 (12.5-35.6)	32.4 (27.4-38.8)	39.5 (34.8-55.1)	0.1640	0.4724
**CD27+**	78.6 (66-92.9)	83.7 (58.9-96.9)	79.7 (60-85.9)	0.9960	1	94.5 (85.9-96.5)	85.9 (78-96.9)	83.6 (70-95.4)	0.6300	0.9125
**CD38+HLA-DR+**	1.43 (0.6-1.68)	0.458 (0.351-1.37)	0.56 (0.501-2.56)	0.2550	0.7802	1.8 (1.32-8.07)	0.661 (0.457-1.46)	1.67 (0.898-9.1)	0.1200	0.4051
**CD8+ Memory T Cells**										
**CD38+**	12.9 (5.89-16.8)	16.9 (10.3-22.3)	26.2 (10.2-48.5)	0.2080	0.7802	10.5 (7.68-13.5)	19 (13.4-29.3)	46.7 (22.9-57.7)	**0.0019**	**0.0369**
**HLA-DR+**	8.45 (7.47-10.7)	12.8 (7.87-15.4)	23.8 (6.35-32.1)	0.5080	0.8555	11.4 (8.43-18.6)	14.5 (12.1-23.8)	32 (21.8-45.7)	**0.0171**	0.133
**CD11b+**	40.8 (36.2-50.9)	38.6 (30.4-57.4)	42.9 (39.3-54)	0.9110	0.9732	35.2 (26.4-48)	36.1 (25.6-48.4)	39.1 (27.9-62.4)	0.8390	0.9321
**CD27+**	85.2 (78.2-88.7)	89 (82.4-92.8)	87.8 (65.6-89.5)	0.5170	0.8555	89 (79.6-95.2)	87.8 (75.6-92.5)	86.2 (85-90.1)	0.9400	0.9783
**CD38+HLA-DR+**	3.86 (1.83-5.56)	5.2 (3.42-7.84)	13.1 (2.58-16.8)	0.3000	0.7802	4.17 (2.6-4.47)	6.37 (5.6-13.1)	24.7 (12.9-33.5)	**0.0013**	**0.0369**
**NK cells**										
**CD38+**	91.1 (88.9-92.9)	93.4 (80.1-97.2)	93.7 (87.6-97.3)	0.6420	0.9494	91.3 (86.1-92.5)	89.2 (85.3-91.4)	95.8 (91.7-96.2)	0.0897	0.3672
**HLA-DR+**	48.2 (31.6-69.1)	45.4 (37.8-58.2)	50 (47.2-59.7)	0.8740	0.9732	37.8 (25.9-59.1)	39.3 (30.3-63.1)	53.1 (37.5-75.4)	0.3150	0.6999
**CD11c+**	91.8 (85.5-92.8)	91.1 (84.9-96)	84.4 (83.1-89.5)	0.2840	0.7802	90.4 (87.3-95.6)	89.8 (86.3-92.9)	87.5 (79.5-91.9)	0.6620	0.9125
**CD11b+**	94.2 (90.4-97.6)	96 (91.6-97.7)	95.8 (91-99.5)	0.9010	0.9732	95.8 (91.3-96.8)	95.8 (90.5-97.6)	90.1 (81.3-96.4)	0.5420	0.9125
**CD27+**	15.9 (14.2-21.1)	12.8 (9.6-17.9)	14.4 (13-19.1)	0.3940	0.8281	16.6 (14.4-20.4)	18.9 (15-25.4)	18.8 (10.7-25.5)	0.7040	0.9125
**Monocytes**										
**CD38+**	90.7 (86.9-94.5)	84 (78.4-88.6)	92.7 (90.6-95.1)	0.0836	0.7259	78.4 (73.8-80.8)	81.4 (77.1-88.7)	89.1 (79.3-94.4)	0.1250	0.4051
**Classical Monocytes**										
**CD38+**	99 (98.3-99.3)	97.2 (93.6-99.2)	98.2 (97.3-98.7)	0.4150	0.8493	97.7 (96.3-98.9)	97.9 (96.7-98.6)	98 (96.2-99.1)	0.9480	0.9783

					**Intermediate Monocytes**					
**CD38+**	56.3 (51.1-77.5)	53.6 (47.1-78.9)	57.1 (47.1-75)	0.9710	0.9936	45.1 (22.7-46.9)	27 (21.5-48.1)	31.4 (22.5-43.4)	0.8080	0.9321
					**Non-Classical Monocytes**					
**CD38+**	7.78 (5.73-14.1)	8.88 (5.91-25.8)	20.8 (7.79-40.9)	0.3110	0.7802	4.29 (3.86-5.19)	6.13 (3.68-12.3)	11 (7.46-18.2)	0.1250	0.4051
					**Basophils**					
**CD11c+**	64.8 (48.7-77.4)	59.7 (48.5-67.9)	56.4 (46.9-70.6)	0.7740	0.9732	59 (47-68)	60.2 (48.7-72.6)	28.1 (19.8-73.4)	0.3540	0.7441
					**DCs**					
**CD38+**	89.4 (82.1-90.5)	81.1 (62.6-91.4)	92.6 (89.7-96)	0.1060	0.7259	82.2 (78.3-91.9)	83.6 (78.8-91.5)	83.1 (62.7-89.4)	0.6400	0.9125
					**pDCs**					
**CD38+**	94.8 (87.1-98.8)	99.6 (95.2-100)	100 (93.6-100)	0.2740	0.7802	94.5 (88.6-97.1)	98.2 (92.6-100)	100 (98.4-100)	0.0528	0.2415

Supplemental Table 11. Significant cell subset activation marker frequency differences.

	White			Black					
Cell-Marker	Ctrl vs SLE INACT	Ctrl vs SLE ACT	SLE INACT vs SLE ACT	Ctrl vs SLE INACT	Ctrl vs SLE ACT	SLE INACT vs SLE ACT	White Ctrl vs Black Ctrl	White SLE INACT vs Black SLE INACT	White SLE ACT vs Black SLE ACT
**Granulocytes**									
CD38+	0.9400	0.5680	0.5680	0.3740	**0.0475**	0.1810	0.9290	0.2900	0.1190
CD11c+	0.3260	0.8700	0.3270	0.3280	0.0829	0.3980	**0.0410**	**0.0065**	0.2710
CD11b+	1.0000	0.4070	0.4340	0.7890	0.7390	0.6690	0.7190	0.5420	0.7880
**B cells**									
CD38+	0.4500	0.1650	0.4140	0.8590	0.4090	0.4810	0.1830	0.7620	0.5180
CD11c+	0.8800	0.2530	0.2210	0.7900	0.2830	0.3600	0.4770	0.5450	0.7900
CD11b+	0.7620	0.5680	0.3690	0.4770	0.3220	0.5260	0.2860	0.5970	0.6210
CD27+	0.8210	0.7440	0.9350	0.6570	0.3220	0.5260	0.7220	0.6500	0.5690
**Non-switched Memory B cells**									
CD38+	0.1400	0.1780	0.8700	0.1310	0.0692	0.8050	0.2860	0.3440	0.5690
CD11c+	0.2120	0.4620	**0.0496**	0.5940	0.5630	0.9440	0.7900	0.6500	0.1190
CD11b+	0.1730	0.5950	0.5670	0.6250	0.2650	0.5970	0.9290	0.2550	0.8490
**Memory B cells/Plasmablasts**									
CD11c+	0.3260	0.6830	0.1650	1.0000	0.3640	0.5260	0.0506	0.5450	**0.0135**
CD11b+	1.0000	0.5670	0.5140	0.5340	0.5630	0.8330	0.5340	0.7620	0.6210
**DN B Cells**									
CD38+	0.5450	**0.0338**	0.1210	0.6570	0.8690	0.6470	1.0000	0.8210	0.1020
CD11c+	0.2410	0.0864	0.4140	0.0621	0.4090	0.5260	0.7900	0.6500	0.2710
CD11b+	0.7620	0.1910	0.1650	0.6570	0.6200	0.8880	0.4240	0.7050	0.3050
**Naïve B Cells**									
CD38+	0.5450	0.2200	0.5670	0.9290	0.2150	0.1050	0.1310	0.4500	0.4250
CD11c+	0.4730	0.9670	0.5390	0.9290	0.2830	0.3240	0.9290	0.3450	0.1590

CD11b+	0.8800	0.5680	0.5140	0.5340	0.3640	0.5260	0.2140	0.5970	0.6210
**Transitional B Cells**									
CD11c+	0.6790	1.0000	0.6270	0.1700	0.3520	0.7940	0.5150	0.6280	0.7450
CD11b+	0.3070	0.6210	0.1410	0.8590	0.1600	0.2050	0.4770	0.1210	0.2380
**T Cells**									
CD38+	0.0588	0.5140	0.5140	1.0000	0.2480	0.4390	0.5940	0.2900	0.2390
HLA-DR+	0.2260	0.0864	0.2210	0.4240	**0.0166**	0.1210	0.5340	0.9400	0.6210
CD11b+	1.0000	0.5680	0.8060	0.7900	0.5090	0.6220	0.2140	0.4500	0.6210
CD27+	0.5450	0.2880	0.6240	0.7900	0.8040	0.9440	0.5940	0.3640	0.1600
CD38+HLA-DR+	0.1510	**0.0412**	0.1910	0.0756	**0.0013**	0.0573	0.4770	0.9400	0.4250
**NKT Cells**									
CD38+	0.2570	0.1020	0.3910	0.1550	**0.0064**	0.0573	0.3740	0.8800	0.9700
HLA-DR+	0.7330	0.1300	0.1910	0.3280	**0.0105**	0.0907	0.4240	0.8200	0.8490
CD11c+	0.4960	**0.0113**	0.2210	0.5940	0.8690	0.6210	0.5340	0.6770	0.0623
CD11b+	0.6770	0.8700	0.4130	0.1310	0.4820	0.6980	0.2860	0.2570	0.8490
CD27+	0.6230	0.7130	0.8380	0.6890	0.5910	0.7250	0.7220	0.2260	0.0742
**CD4+ T Cells**									
CD38+	**0.0052**	0.2530	0.2880	0.8590	0.9340	1.0000	0.3280	0.1740	0.4250
HLA-DR+	0.4500	0.0500	0.1020	0.3740	**0.0166**	0.0910	0.9290	0.8800	0.7900
CD11b+	0.7620	0.5680	0.8700	0.7900	0.5090	0.4390	0.4240	0.4960	0.5690
CD27+	0.1310	0.0500	1.0000	1.0000	0.6200	0.2910	0.7220	0.2570	**0.0109**
CD38+HLA-DR+	0.3260	**0.0090**	0.0864	0.1830	**0.0039**	0.0783	0.8590	0.7620	0.9090
**CD8+ T Cells**									
CD38+	0.1510	0.5140	1.0000	0.7220	0.0575	0.2310	0.1550	0.5450	0.1190
HLA-DR+	0.4500	0.3690	0.5680	0.6570	**0.0082**	0.0910	0.8590	0.7620	0.4250
CD11b+	0.9400	0.8060	0.6830	0.4770	0.1600	0.2310	0.1830	0.4060	0.4250
CD27+	0.8210	0.7440	0.7440	0.6570	0.3640	0.5730	0.1310	0.4960	0.6760
CD38+HLA-DR+	0.2570	0.5140	0.6240	0.0621	**0.0010**	**0.0201**	0.4770	0.7050	0.2390
**DN T Cells**									
CD38+	0.1510	**0.0412**	0.6240	**0.0077**	**0.0023**	0.0671	0.8590	0.5970	0.1600

HLA-DR+	0.2260	0.1110	0.4140	0.2860	0.1370	0.0671	0.2860	0.3260	0.9090
CD11b+	0.1510	0.3690	0.6240	0.3280	0.6200	0.9440	**0.0129**	0.3260	0.5690
CD27+	0.4500	1.0000	0.7440	0.3740	0.6200	0.7250	0.1550	0.0588	**0.0304**
CD38+HLA-DR+	**0.0211**	**0.0090**	0.2880	0.3740	**0.0105**	**0.0411**	0.2860	0.5450	0.4250
**CD4+ Naïve/Effector T Cells**									
CD38+	0.1310	0.3690	0.8700	0.4770	0.1860	0.7780	0.8590	0.3640	0.7320
HLA-DR+	0.3640	0.3270	0.9020	0.9290	**0.0208**	**0.0092**	0.7220	0.2900	0.4250
CD11b+	0.7620	0.6240	0.9350	0.7220	0.6200	0.4810	0.4240	0.4500	0.5690
CD27+	0.1510	0.2210	0.8380	0.8590	0.4090	0.6220	0.1830	0.5970	0.1590
CD38+HLA-DR+	0.7620	0.2210	0.4870	0.7900	0.0986	0.1930	0.7220	0.6230	0.7610
**CD4+ Memory T Cells**									
CD38+	0.3260	0.5140	0.0724	0.7900	**0.0166**	0.0910	0.3740	0.5970	0.6760
HLA-DR+	0.2900	0.0864	0.0500	0.3280	**0.0208**	0.1590	0.1830	0.2570	0.5690
CD11b+	0.8800	0.6240	0.7440	0.8590	0.5090	0.5730	0.3280	0.5970	0.4250
CD27+	0.5970	0.2530	0.5140	0.6570	0.3220	0.1590	0.7220	0.8800	**0.0056**
CD38+HLA-DR+	1.0000	**0.0338**	**0.0338**	0.3740	**0.0039**	0.0573	0.7220	0.5970	0.9700
**CD8+ Naïve/Effector T Cells**									
CD38+	0.5450	0.6830	0.7440	1.0000	0.2480	0.2910	0.1830	0.7050	0.1190
HLA-DR+	0.6500	0.2210	0.5680	0.7900	**0.0390**	0.2050	0.9290	0.8800	0.6210
CD11b+	0.8800	0.5140	0.8060	0.2480	0.0986	0.2310	0.1310	0.4960	0.2390
CD27+	0.9400	1.0000	0.9350	0.7900	0.3220	0.5730	0.1830	0.6500	0.5690
CD38+HLA-DR+	0.0963	0.6240	0.3270	0.0506	0.7410	0.1210	0.1830	0.4500	0.3050
**CD8+ Memory T Cells**									
CD38+	0.1740	0.1420	0.3690	**0.0263**	**0.0010**	0.0783	0.7900	0.7620	0.3820
HLA-DR+	0.4500	0.2880	0.5680	0.1550	**0.0050**	0.1390	0.4240	0.3260	0.2100
CD11b+	0.7050	0.6830	0.8060	0.9290	0.6800	0.5730	0.5940	0.6500	0.7320
CD27+	0.3260	0.9350	0.3270	0.7220	0.8040	0.9440	0.3740	0.8800	0.6760
CD38+HLA-DR+	0.1990	0.1910	0.6240	**0.0077**	**0.0017**	0.0573	0.7220	0.2260	0.1380
**NK Cells**									
CD38+	0.7620	0.3690	0.5130	0.5940	0.0985	**0.0486**	0.8590	0.4960	0.8490

HLA-DR+	0.8210	0.8060	0.5140	0.6570	0.1370	0.3240	0.5940	0.5970	0.6210
CD11c+	0.7340	0.2210	0.1420	0.4240	0.4570	0.7250	0.9290	0.4500	0.4250
CD11b+	0.7340	0.6830	0.8700	0.7900	0.3210	0.3770	0.9290	0.9100	0.2670
CD27+	0.2260	0.4140	0.4140	0.5050	0.8360	0.4600	0.7220	0.0963	0.6210
**Monocytes**									
CD38+	0.1120	0.4140	**0.0412**	0.2140	**0.0593**	0.2310	**0.0129**	0.4060	0.2710
**Classical Monocytes**									
CD38+	0.3260	0.2530	0.4140	0.9290	0.6800	0.9440	0.1310	0.4960	0.5690
**Intermediate Monocytes**									
CD38+	0.7910	0.9350	0.7750	0.5940	0.6800	0.6470	0.0560	**0.0155**	**0.0167**
**Non-Classical Monocytes**									
CD38+	0.7050	0.1210	0.3270	0.3740	**0.0318**	0.3600	**0.0263**	0.4960	0.2390
**Basophils**									
CD11c+	0.7050	0.4620	0.7750	0.9290	0.2150	0.2310	0.5940	0.9400	0.3050
**DCs**									
CD38+	0.3260	0.1640	**0.0499**	0.9290	0.3640	0.4810	0.4770	0.6500	**0.0303**
**pDCs**									
CD38+	0.1090	0.2890	0.8950	0.2050	**0.0184**	0.1970	0.7550	0.7160	0.3490

Supplemental Table 12. TLR gene list

lps	lps	lps	lps	lps	lps	lps	lps	lps	lps	polyIC	polyIC	polyIC	polyIC	r848	R848	tlr7	tlr9
ACADM	CCND3	CTNNB1	FOS	ID2	LCP2	NDUFV2	PSMA2	SERPINB9	TMEM154	ACSL3	DDX60L	JUN	RPL11	ADAR	IRF7	BANK1	CAST
ACP5	CCNI	CTSB	FOSB	IER5	LDHA	NFAT5	PSMA6	SLC11A1	TMSB10/TMSB4X	ADAM10	DISC1	JUNB	RPL12	AIF1	IRF9	BCL2	CCL3
ACSL1	CCNL1	CTSC	FOXO1	IFI16	LGALS1	NFE2L2	PSMB10	SLC16A3	TNFAIP2	ADAR	DUSP1	KLF4	RPL13A	APOBEC3A	ISG15	BCL2A1	CCL5
ADAR	CCPG1	CTSL	FPR1	IFI35	LGALS3	NFIL3	PSMB8	SLC2A3	TNFAIP3	AIMP1	EEF1G	KLF6	RPL15	ATF4	ISG20	CCL3	CCR7
ADGRE2	CCR1	CXCL16	FTH1	IFI44	LGALS9	NFKB1	PSMB9	SLC31A2	TNFAIP6	ANXA1	EGR1	KYNU	RPL17	ATF6	JUN	CCND3	CD300A
AFF3	CCR7	CXCL2	FUT8	IFI44L	LIMS1	NFKBIA	PSME1	SLC66A3	TNFAIP8	ATF4	EIF2AK2	LGALS9	RPL19	ATP5F1A	LAP3	CCR7	CD300E
AHNAK	CD14	CXCL3	FYB1	IFI6	LIPA	NFKBIZ	PSME2	SMAD2	TNFRSF1B	ATP5MG	EIF2S3	LTA4H	RPL3	ATP5F1C	LYN	CD300A	CD69
AHR	CD22	CXCL8	FYN	IFITM1	LITAF	NLRP3	PSMG4	SMARCE1	TNFSF10	AUTS2	EIF5A	LTB	RPL30	ATP5PF	LYST	CD44	CD83
AKAP13	CD300LF	CXCR4	G0S2	IFITM2	LMO4	NOTCH2	PTGS2	SNAP23	TNFSF13B	B2M	EREG	LY6E	RPS13	ATP6V1B2	MCL1	CD69	CD86
ALDH2	CD36	CYBA	GABARAP	IFNGR2	LRBA	NR3C1	PTPRC	SOCS3	TOR3A	BAX	ETFB	LYN	RPS2	BCL2	MSN	CD79A	CERS6
ALOX5AP	CD37	CYBB	GADD45B	IGHM	LRP1	NR4A2	PYCARD	SOD1	TPI1	BCL2	EZR	LYZ	RPSA	BCL2A1	MX1	CD83	CXCL2
ANXA1	CD4	CYCS	GAS5	IGKC	LRRFIP1	NT5C3A	QKI	SOD2	TPT1	BIRC2	FAU	M6PR	SAP18	BST2	MX2	CD86	CXCL3
ANXA5	CD44	CYTIP	GBP1	IGSF6	LRRK2	OAS1	RAB6A	SP140	TRAF3	BIRC3	FCN1	MX1	SARS1	CASP4	NAMPT	CEBPB	CXCL8
ANXA7	CD48	DDX17	GBP2	IKZF1	LST1	OLR1	RAN	SPCS1	TREM1	BIRC5	FLI1	MYC	SAT1	CCL3	NFE2L2	CREB5	DUSP1
APEX1	CD53	DDX27	GCA	IKZF3	LTA4H	OSCAR	RARA	SPI1	TRIM22	BST2	FOS	NAAA	SLC25A4	CCL3L1	NFKB1	CXCL2	EGR1
APOBEC3A	CD55	DDX3X	GCH1	IL10RA	LY86	PALM2AKAP2	RASGEF1B	SPTLC2	TSC22D3	BTLA	FPR1	NAMPT	SNRPD1	CCL4L1/CCL4L2	NFKBIA	CXCL3	FCER2
AREG	CD58	DDX6	GDI2	IL10RB	LY96	PARK7	RB1	SSB	TSPO	CCL3	GADD45B	NEDD9	SNU13	CCL5	NFKBIZ	CXCL8	FCMR
ARL4C	CD63	DICER1	GMFG	IL15	LYN	PARP14	RB1CC1	SSBP1	TXN	CCL3L1	GBP1	NFIL3	SOCS3	CCR1	NLRP3	DENND4C	GADD45B
ARPC1B	CD68	DNAJC3	GOLGA4	IL1B	LYZ	PARP8	RBMS1	SSR1	TXNDC11	CCL5	GBP2	NFKB1	SOD2	CCR7	OAS1	DMXL2	GCH1
ASAP1	CD69	DNMT1	GPD2	IL2RG	M6PR	PAX5	REL	ST6GAL1	TXNIP	CCND3	GCH1	NFKBIA	SP110	CD44	PARK7	EIF3J	HMOX1
ASCC3	CD74	DOCK10	GPR183	IL4R	MAFB	PBRM1	RELB	STAP1	TYMP	CCR7	HIF1A	NFKBIZ	SRGN	CD83	PAX5	ETS2	IFI16
ASH1L	CD83	DOCK8	GSK3B	INPP5D	MAP1LC3B	PDCD4	RFX7	STAT1	TYROBP	CCT5	HLA-A	NLRP3	SRSF2	CD86	PKM	FCER1G	IFI35
ATF4	CD86	DUSP1	GSTP1	INSIG1	MAP3K8	PDE3B	RGS2	STAT2	UBE2I	CCT7	HLA-B	NMI	SSB	CEBPB	PLSCR1	FCMR	IFI44L
ATG7	CD9	EAF2	GZMA	IQGAP1	MAP4K4	PDE4B	RHOB	STAT3	UBE2J1	CD14	HLA-C	NOP58	ST6GAL1	CLEC4E	PMAIP1	GNG2	IFITM1
ATP2B1	CDC42EP3	EEF2	GZMB	IRAK3	MARCKS	PDIA3	RIPK2	STAT4	UCP2	CD36	HLA-DMB	NR4A2	ST8SIA4	CLEC7A	PRDM1	HIVEP2	IL10RA

ATP5MC1	CEBPB	EGR1	H3-3A/H3-3B	IRF1	MARCKSL1	PDIA4	RNF19A	STIM1	UTRN	CD44	HLA-E	NUP153	STAT1	CREB5	PRDX1	IFI35	IL1B
ATP6AP1	CEBPD	EGR2	HADHB	IRF7	MCL1	PDIA6	RNF213	STMN1	VCAN	CD47	HSPA9	OAS1	STAT2	CSF1R	PSMB2	IFI44	IRF7
ATP6V0D1	CEMIP2	EIF2AK2	HCK	IRF8	MDH1	PECAM1	RORA	STX11	VIM	CD69	HSPD1	OLR1	SYK	CXCL2	PTGS2	IFI44L	ISG15
ATRX	CFD	EIF2AK3	HDAC9	IRF9	MEF2C	PELI1	RPLP0	STXBP2	VSIR	CD74	IFI16	OXA1L	TANK	CXCL3	PTPRC	IFITM1	LAMP2
B4GALT5	CFLAR	ELAVL1	HERC5	ISG15	MIF	PID1	RPS14	SUMO1	VTI1B	CD83	IFI44	PARP14	TET2	CXCL8	PYCARD	IL10RA	LYST
BACH2	CFP	ELL2	HERPUD1	ISG20	MOB1A	PIK3AP1	RPS19	SUMO2	WARS1	CD86	IFI44L	PDHB	TGFB1	DENND4C	RAB7A	IL1B	MCL1
BAX	CKAP4	EMB	HHEX	ITGA4	MSN	PIM2	RPS2	SWAP70	WTAP	CDK14	IFI6	PLAAT4	THBS1	DNAJC3	RAP1A	INPP5D	MX1
BCL2	CLEC4E	ENO1	HIF1A	ITGAE	MT-CO1	PKIG	RPS5	TAGAP	XAF1	CEBPB	IFITM1	PLAUR	THOC2	EIF2AK2	REL	IRF7	MX2
BCL2A1	CLEC7A	ENTPD1	HIVEP2	ITGAM	MT2A	PKM	S100A12	TALDO1	XBP1	CEBPD	IFITM3	PMAIP1	THRAP3	ETS2	RELB	ISG15	NAMPT
BID	CLIC4	EP300	HLA-A	ITGB1	MTDH	PLAAT4	S100A8	TANK	YBX1	CFLAR	IFNGR1	PPP1R15A	TLR2	FCER1G	RIPK2	ISG20	NFKBIZ
BIRC2	CLPTM1L	ERCC1	HLA-DMA	ITGB2	MTSS1	PLAUR	S100A9	TAPBP	YBX3	CLEC4E	IL10RA	PPP2CA	TLR4	FCER2	SFPQ	JUNB	PTGS2
BIRC3	CMPK2	EREG	HLA-DMB	IVNS1ABP	MX1	PLEK	SAMHD1	TCF4	YWhiteAE	CLEC7A	IL15	PSMA3	TNFAIP2	FCGR2A	SOD2	MX1	PYCARD
BTG1	CNST	ERGIC1	HLA-DOB	JAK2	MX2	PLPP5	SAMSN1	TCF7L2	YWhiteAQ	CMPK2	IL1B	PSMB8	TNFAIP3	GBP1	SP100	MX2	S100A8
C1QA	CORO1A	ERP44	HLA-DQA1	JAZF1	MXD1	PLSCR1	SAP18	TENT5C	YY1	COX20	IL2RG	PSMB9	TNFAIP6	GNG2	SPI1	NCF4	SOCS3
C5AR1	COX4I1	ETS2	HLA-DQB1	JCHAIN	MYC	PMAIP1	SAT1	TFDP2	ZBTB7A	COX7A2L	IL4R	PSME2	TNFSF10	HCK	STAP1	NFKB1	SPTLC2
CALHM6	CREB3L2	ETV6	HLA-DRB5	JUN	MYH9	PNOC	SDHB	TFEC	ZC3HAV1	COX7B	IMMP2L	PTBP2	TNFSF13B	HERC5	STAT1	NFKBIA	STAT1
CALM1 (includes others)	CREBBP	FABP5	HM13	JUNB	NAMPT	POU2F2	SEC11A	TFRC	ZEB1	CSNK1A1	IQGAP2	PTGS2	TOP1	HERPUD1	STAT2	PTPN6	STAT2
CALR	CRTAP	FCER1G	HMGB1	JUND	NCF1	PPIA	SEC24D	TGFB1	ZFC3H1	CTNNB1	IRF1	PTPN2	UBE3A	HHEX	STMN1	RPL26	TGFB1
CASP1	CSF1R	FCER2	HMGB2	KLF10	NCF2	PPP1CB	SEC61A1	THBS1	ZFHX3	CXCL2	IRF7	PTPRJ	USP25	HIVEP2	TLR2	RPL32	TIMP1
CASP4	CSF2RA	FCGRT	HMOX1	KLF2	NCL	PPP1R15A	SELL	TIMP1	ZFP36	CXCL3	IRF9	REL	VASP	HSPB1	TLR4	SOD2	TLR4
CAV1	CSF2RB	FGL2	HSH2D	KLF3	NCOA1	PPP1R16B	SELPLG	TIMP2	ZFP36L2	CXCL8	ISG15	RELB	VIM	IFI16	TMEM154	STAT1	TNFRSF1B
CCL3	CSF3R	FLI1	HSP90B1	KLF4	NCOA2	PRDM1	SEPTIN1	TKT	ZYX	CXCR4	ISG20	RGS2	WDFY4	IFI44L	TNFAIP3	STAT2	TNFSF10
CCL3L1	CST3	FNDC3A	HSPA8	KLF6	NCOA3	PRDX1	SERPINA1	TLR2		CYC1	ITGAM	RNF114	XAF1	IFITM1	TNFSF10	TMEM154	TNFSF13B
CCL5	CSTB	FNDC3B	HSPB1	KYNU	NDFIP1	PRDX5	SERPINB1	TLR4		DDX3X	JAK2	RPL10A	YARS1	IFITM3	TNFSF13B	TNFAIP3	
													ZBP1	IL15	TRIM22		
													ZC3HAV1	IL1B	WARS1		
													ZFP36	IRAK3	XBP1		

YWhite
AB

Supplemental Table 13. Fold change in phospho-proteins following B cell stimulation with various stimuli. Median metal intensity and IQR shown for unstimulated/basal conditions. Median fold change and IQR over the unstimulated/basal conditions shown for all other stimuli.

B Cells										
	Median (Interquartile Range)					Median (Interquartile Range)				
	**White Controls**	**White SLE INACT**	**White SLE ACT**	**KW p-value**	**FDR q-value**	**Black Controls**	**Black SLE INACT**	**Black SLE ACT**	**KW p-value**	**FDR q-value**
**cCASP3**										
Basal	6.56 (6.19-7.2)	7.29 (6.77-10.5)	9.99 (7.24-12)	0.1920	0.8207	7.16 (6.24-8.27)	6.91 (6.42-8.98)	6.87 (6.72-10.8)	0.6920	0.8387
IFNα	1.13 (1-1.19)	1.31 (1.05-1.39)	1.78 (0.979-4.38)	0.4500	0.8363	1.11 (1.05-1.49)	1.67 (1.14-3.38)	1.27 (1.11-1.49)	0.3860	0.7772
LPS (TLR4)	1.02 (1-1.07)	1 (0.81-1.16)	1.06 (0.536-1.18)	0.9470	0.9918	1.07 (0.927-1.17)	1.12 (1.01-1.28)	1.01 (0.988-1.1)	0.4560	0.7772
R848 (TLR7/8)	1.02 (0.951-1.05)	0.961 (0.856-1.12)	1.08 (0.912-1.47)	0.8000	0.926	1.15 (1.07-1.68)	1.03 (0.966-1.21)	1.04 (0.951-1.07)	0.1610	0.7317
CpG (TLR9)	1.05 (0.935-1.08)	0.942 (0.693-1.03)	1.02 (0.882-1.08)	0.3120	0.8207	1.53 (1.19-2)	0.976 (0.954-1.03)	1.04 (0.964-1.09)	**0.0050**	0.2263
PMA-Iono	0.963 (0.916-1.12)	1.1 (1.05-1.13)	1.25 (1.04-2.11)	0.3020	0.8207	1.13 (1.04-1.32)	1.16 (1.06-1.72)	1.14 (1.06-1.42)	0.9820	0.9918
**pCREB**										
Basal	19 (16.2-25.1)	18.1 (13-22.2)	21.9 (20.1-31.6)	0.4460	0.8363	20.1 (13.8-20.9)	27.5 (16.8-43.5)	18.5 (11.8-25.2)	0.4500	0.7772
IFNα	1.05 (0.989-1.1)	1.27 (1.09-1.41)	1.43 (1.06-1.75)	0.1160	0.8207	1.23 (1.14-1.5)	1.11 (0.994-1.26)	1.18 (1.07-1.53)	0.3300	0.7772
LPS (TLR4)	1.04 (0.944-1.16)	1.11 (1-1.25)	1.08 (0.908-1.32)	0.7040	0.926	1.11 (1.08-1.52)	1.02 (0.946-1.12)	1.05 (0.926-1.1)	0.0802	0.7254
R848 (TLR7/8)	1.15 (1.13-1.19)	1.23 (1.02-1.63)	1.43 (0.806-2.05)	0.8570	0.9539	1.49 (1.35-2.47)	1.09 (0.982-1.26)	1.42 (1.21-1.76)	**0.0409**	0.5577
CpG (TLR9)	1.02 (0.947-1.09)	1.03 (0.934-1.15)	0.949 (0.602-1.15)	0.7250	0.926	1.09 (1.02-1.24)	0.948 (0.834-1.04)	1.02 (0.943-1.1)	0.1200	0.7254

PMA-Iono	1.87 (1.59-2.64)	2.69 (2.39-4.13)	2.1 (1.63-3.88)	0.3120	0.8207	2.17 (2.13-4.61)	2.34 (1.52-2.88)	2.67 (2.33-3.2)	0.2930	0.7772
**pERK1/2**										
Basal	5.27 (4.71-6.86)	5.78 (5.07-9.83)	4.8 (4.02-7.34)	0.7030	0.926	5.02 (4.29-5.58)	4.86 (4.59-6.93)	4.78 (3.99-6.08)	0.8240	0.8642
IFNα	0.981 (0.907-1.14)	1.14 (1.11-1.28)	1.25 (0.978-1.46)	0.3860	0.8363	1.12 (0.985-1.88)	1.17 (1.06-2.16)	1.07 (0.946-1.14)	0.3790	0.7772
LPS (TLR4)	1.01 (0.894-1.08)	1.14 (0.876-1.21)	1.29 (1.05-1.51)	0.1830	0.8207	1.1 (1.04-1.24)	1.06 (1.01-1.15)	1.02 (1.01-1.05)	0.2100	0.7772
R848 (TLR7/8)	1.04 (0.964-1.14)	1.15 (1.04-1.36)	0.927 (0.868-1.3)	0.4390	0.8363	1.39 (1.24-1.75)	1.26 (1.04-1.63)	1.3 (1.2-2.57)	0.3640	0.7772
CpG (TLR9)	1.01 (0.989-1.16)	0.98 (0.879-1.13)	1.15 (0.843-1.19)	0.7890	0.926	1.06 (0.953-1.31)	1.03 (0.888-1.1)	0.986 (0.963-1.02)	0.4790	0.7772
PMA-Iono	5.77 (4.01-6.67)	5.16 (3.56-10.9)	5.53 (3.52-6.96)	0.9720	0.9918	9.04 (5.55-10.4)	6.34 (5.22-8.38)	6.84 (5.16-7.82)	0.4670	0.7772
**p-p38**										
Basal	10.4 (8.64-19.6)	9.45 (7.68-10.4)	9.65 (6.64-13.2)	0.5660	0.882	6.5 (5.78-10)	9.7 (6.05-15.7)	6.34 (5.62-10.7)	0.4900	0.7772
IFNα	1.05 (0.957-1.14)	1.2 (0.987-1.41)	1.41 (1.11-1.79)	**0.0371**	0.8207	1.18 (1.03-1.27)	1.08 (0.947-1.25)	1.2 (1.04-1.27)	0.7870	0.8585
LPS (TLR4)	1.16 (1.04-1.22)	1.3 (1.07-1.39)	1.85 (1.38-1.95)	**0.0137**	0.7472	1.39 (1.17-1.83)	1.13 (1.05-1.28)	1.11 (1.03-1.36)	0.3550	0.7772
R848 (TLR7/8)	1.31 (1.19-1.68)	1.47 (1.24-2.03)	2.07 (1.91-2.32)	0.0755	0.8207	2.34 (1.85-3.08)	1.49 (1.18-1.75)	2.57 (1.81-3.01)	0.1170	0.7254
CpG (TLR9)	1.03 (1.02-1.05)	0.957 (0.926-1.01)	1.12 (1.07-1.26)	0.1210	0.8207	1.12 (1.01-1.58)	1 (0.852-1.1)	1.01 (0.943-1.11)	0.1970	0.7772
PMA-Iono	1.52 (1.28-2.21)	1.7 (1.55-2.5)	2.91 (2.46-3.97)	0.1920	0.8207	2.53 (1.99-2.81)	1.43 (1.29-1.85)	1.69 (1.26-2.57)	0.1140	0.7254
**p-PLCg2**										
Basal	9.66 (5.48-12)	10.3 (5.98-12.9)	10.4 (6.62-12.1)	0.9700	0.9918	8.81 (7.78-11)	8.16 (6.99-9.86)	9 (7.38-10.2)	0.8220	0.8642
IFNα	0.925 (0.909-1.01)	0.953 (0.915-1.1)	0.962 (0.917-0.993)	0.7470	0.926	0.997 (0.959-1.01)	0.967 (0.888-1.02)	1.01 (0.939-1.05)	0.6220	0.8272

LPS (TLR4)	0.964 (0.961-1.04)	0.936 (0.92-1.12)	1.02 (0.986-1.06)	0.4020	0.8363	1 (0.985-1.02)	1.01 (0.994-1.07)	0.976 (0.923-1)	0.3100	0.7772
R848 (TLR7/8)	1.02 (1-1.04)	1 (0.908-1.06)	0.968 (0.91-1.13)	0.7470	0.926	1.03 (0.995-1.08)	0.971 (0.897-1.01)	0.967 (0.905-1.01)	0.1300	0.7254
CpG (TLR9)	1.02 (0.87-1.11)	0.96 (0.94-1.01)	1.04 (0.946-1.08)	0.7420	0.926	1.01 (0.978-1.05)	1.01 (0.871-1.06)	0.972 (0.853-1.03)	0.5130	0.7772
PMA-Iono	0.923 (0.887-1.1)	0.996 (0.914-1.07)	1.06 (0.964-1.26)	0.2710	0.8207	0.944 (0.92-0.97)	0.895 (0.853-0.993)	0.929 (0.888-0.978)	0.6660	0.8387
**pSTAT1**										
Basal	3.9 (2.71-5.02)	6.03 (5.48-7.28)	7.92 (6.06-13.2)	0.0606	0.8207	3.7 (2.36-5.31)	3.7 (3.01-5.63)	6.04 (3.86-8.54)	0.4830	0.7772
IFNα	10.1 (5.93-12.5)	8.83 (3.1-11.4)	2.48 (1.4-8.3)	0.2630	0.8207	9.61 (6.25-13.8)	8.63 (4.05-14.1)	4.85 (2.36-13.2)	0.7170	0.8501
LPS (TLR4)	1 (0.808-1.18)	0.933 (0.841-1.13)	0.823 (0.285-1.57)	0.7520	0.926	1.06 (0.951-1.2)	1.17 (1.09-1.31)	1.08 (0.991-1.22)	0.5030	0.7772
R848 (TLR7/8)	0.988 (0.893-1.08)	0.981 (0.811-1.04)	1.06 (0.267-1.26)	0.3630	0.8363	1.09 (0.988-1.3)	0.973 (0.934-1.11)	1 (0.903-1.21)	0.5770	0.8154
CpG (TLR9)	0.971 (0.833-1.07)	0.892 (0.772-1.16)	0.787 (0.246-0.921)	0.7270	0.926	1.07 (0.96-1.51)	0.993 (0.771-1.08)	1.01 (0.899-1.1)	0.6370	0.8272
PMA-Iono	1.1 (0.972-1.23)	1.35 (1.01-1.49)	0.939 (0.734-1.35)	0.4600	0.8363	1.2 (0.959-1.5)	1.46 (0.981-1.63)	1.37 (1.07-1.46)	0.7690	0.8559
**pSTAT3**										
Basal	8.45 (6.46-12)	8.6 (7.11-13.1)	13.3 (9.72-13.5)	0.2710	0.8207	7.41 (5.82-9.04)	5.74 (4.86-9.23)	9.82 (5.92-12.3)	0.4710	0.7772
IFNα	4.38 (2.42-5.59)	4.31 (2.09-5.33)	3.81 (1.97-4.16)	0.9820	0.9918	4.34 (3.6-5.92)	3.43 (2.2-6.22)	2.04 (1.6-2.96)	0.1330	0.7254
LPS (TLR4)	1.02 (0.953-1.07)	1.02 (0.895-1.09)	1.07 (0.778-1.27)	0.8150	0.926	1.07 (0.99-1.37)	1.01 (0.952-1.12)	1.05 (0.894-1.19)	0.6850	0.8387
R848 (TLR7/8)	1.04 (0.963-1.13)	1.02 (0.956-1.1)	0.973 (0.645-1.09)	0.5460	0.882	0.998 (0.938-1.28)	0.99 (0.909-0.997)	1.03 (0.928-1.11)	0.5980	0.8154
CpG (TLR9)	0.982 (0.892-1.11)	0.973 (0.879-0.995)	0.828 (0.67-1.02)	0.4770	0.8392	0.973 (0.922-1.24)	0.914 (0.835-1)	0.961 (0.864-1.06)	0.5560	0.8154
PMA-Iono	0.955 (0.864-1.01)	1.03 (0.967-1.11)	1.08 (0.924-1.32)	0.1690	0.8207	1 (0.884-1.09)	1.06 (0.865-1.19)	0.951 (0.883-1.07)	0.7550	0.8559

**pSTAT5**										
Basal	9.21 (6.71-10.3)	10.6 (6.61-13.2)	10.9 (9.59-17.6)	0.3020	0.8207	7.88 (6.99-11.3)	9.97 (7.72-12.3)	11.5 (9.14-17)	0.3760	0.7772
IFNα	1.9 (1.66-2.12)	2.16 (1.77-2.49)	1.42 (1.09-2.92)	0.4940	0.842	3.14 (2.52-4.16)	3.35 (1.92-3.96)	1.23 (1.07-2)	**0.0083**	0.2263
LPS (TLR4)	1.1 (0.98-1.15)	1.03 (0.9-1.08)	1.02 (0.953-1.18)	0.7700	0.926	1.03 (0.956-1.09)	1.07 (1.04-1.13)	0.993 (0.961-1.11)	0.2960	0.7772
R848 (TLR7/8)	1.08 (1.02-1.27)	0.965 (0.9-1.06)	1.08 (0.924-1.2)	0.2070	0.8207	1.06 (1.03-1.1)	1.06 (0.979-1.19)	0.992 (0.966-1.07)	0.3900	0.7772
CpG (TLR9)	0.999 (0.931-1.1)	0.971 (0.829-1.02)	0.934 (0.904-1.03)	0.4550	0.8363	1.1 (0.983-1.51)	1.02 (0.951-1.05)	0.975 (0.899-1.06)	0.3250	0.7772
PMA-Iono	1.06 (0.979-1.07)	1.08 (1.02-1.28)	1.08 (1.01-1.34)	0.7340	0.926	1.11 (1.02-1.26)	1.18 (1.12-1.39)	1.26 (1.12-1.34)	0.5970	0.8154
**Syk**										
Basal	43.9 (30.8-52.6)	48.7 (34.6-52.1)	42.9 (37.9-58)	0.9740	0.9918	40.9 (34.7-50.9)	41.9 (33.3-52.3)	45.1 (37.4-59.1)	0.7540	0.8559
IFNα	1.01 (0.929-1.07)	1.29 (1.06-1.43)	1.49 (0.937-1.6)	0.1080	0.8207	1.19 (1.07-1.37)	1.17 (1.01-1.42)	1.16 (1.06-1.37)	0.9500	0.9776
LPS (TLR4)	1.05 (0.942-1.09)	1.04 (0.936-1.16)	0.961 (0.801-1.05)	0.5550	0.882	1.05 (1.01-1.13)	1.05 (0.988-1.09)	1.02 (0.983-1.07)	0.5050	0.7772
R848 (TLR7/8)	1.04 (0.939-1.06)	0.942 (0.906-1.03)	0.824 (0.8-1.01)	0.1630	0.8207	1.29 (1.04-1.49)	0.961 (0.878-1.01)	1.07 (0.987-1.16)	**0.0227**	0.4127
CpG (TLR9)	1.08 (0.904-1.25)	0.96 (0.919-0.989)	0.919 (0.825-1.08)	0.4320	0.8363	1.08 (1.01-1.37)	0.97 (0.895-1.06)	0.995 (0.982-1.03)	0.1610	0.7317
PMA-Iono	1.04 (0.94-1.14)	1.16 (1.05-1.26)	1.12 (0.97-1.52)	0.3160	0.8207	1.15 (1.06-1.22)	1.17 (1.03-1.31)	1.23 (1.14-1.52)	0.3680	0.7772

Supplemental Table 14. Fold change in phospho-proteins following CD4+ T cell stimulation with various stimuli. Median metal intensity and IQR shown for unstimulated/basal conditions. Median fold change and IQR over the unstimulated/basal conditions shown for all other stimuli.

CD4+ T Cells										
	Median (Interquartile Range)					Median (Interquartile Range)				
	**White Controls**	**White SLE INACT**	**White SLE ACT**	**KW p-value**	**FDR q-value**	**Black Controls**	**Black SLE INACT**	**Black SLE ACT**	**KW p-value**	**FDR q-value**
**cCASP3**										
Basal	5.53 (4.64-6.11)	5.94 (5.22-7.82)	6.36 (5.81-7.42)	0.2480	0.8817	5.71 (5.01-5.91)	5.84 (4.89-6.67)	5.6 (5.15-9.55)	0.7680	0.9109
IFNα	1.05 (0.993-1.08)	1.21 (1.06-1.25)	1.3 (1.13-1.47)	0.0803	0.7299	1.1 (1.01-1.25)	1.38 (1.16-1.59)	1.12 (1.04-1.23)	0.0580	0.2925
LPS (TLR4)	1.03 (0.964-1.07)	1.01 (0.94-1.08)	0.958 (0.938-1.01)	0.5510	0.9712	1.06 (0.996-1.1)	1.02 (0.998-1.07)	1.02 (0.978-1.07)	0.5970	0.8683
R848 (TLR7/8)	0.974 (0.915-1.04)	0.973 (0.929-1.04)	1 (0.914-1.03)	0.9770	0.9969	1.04 (1-1.21)	0.982 (0.963-1.07)	1.01 (0.958-1.04)	0.3050	0.7561
CpG (TLR9)	0.989 (0.932-1.06)	0.96 (0.914-1.01)	0.984 (0.956-1.04)	0.8050	0.9969	1.19 (1-1.34)	1.02 (0.968-1.1)	1.03 (0.984-1.05)	0.1750	0.5965
PMA-Iono	1.01 (0.973-1.07)	1.05 (1-1.14)	1.17 (1.07-1.37)	0.0679	0.7299	1.09 (1.05-1.22)	1.14 (1.12-1.32)	1.14 (1.04-1.23)	0.4870	0.8683
**pCREB**										
Basal	9.4 (7.58-13)	9.57 (8.2-12.8)	10.5 (7.85-18.5)	0.6420	0.9969	7.36 (6.05-12.7)	10.8 (7.63-16.3)	7.98 (5.46-12.5)	0.2100	0.6737
IFNα	1.02 (0.97-1.17)	1.18 (1.08-1.32)	1.01 (0.682-1.35)	0.2930	0.8817	1.15 (1.02-1.57)	1.1 (1.05-1.64)	1.11 (1.09-1.37)	0.9870	1
LPS (TLR4)	1.04 (0.983-1.15)	1.1 (1.06-1.15)	1.06 (0.856-1.17)	0.5450	0.9712	1.2 (1.12-1.27)	1.11 (0.974-1.15)	1.06 (1.05-1.17)	0.1200	0.4363
R848 (TLR7/8)	1.02 (0.953-1.17)	1.04 (0.985-1.08)	1.03 (0.982-1.14)	0.9830	0.9969	1.24 (1.08-1.35)	0.984 (0.931-1.06)	1.06 (1.05-1.15)	**0.0066**	0.1745

CpG (TLR9)	0.982 (0.963-1.11)	1 (0.968-1.09)	1.04 (0.935-1.08)	0.9770	0.9969	1.18 (1.13-1.32)	0.99 (0.872-1.11)	1.05 (0.988-1.07)	**0.0128**	0.1745
PMA-Iono	3.62 (2.76-4.89)	3.03 (2.01-4.71)	3.59 (1.96-4.26)	0.6910	0.9969	4.64 (4.03-6.05)	3.8 (2.16-4.83)	4.18 (2.98-7.22)	0.3190	0.7564
**pERK1/2**										
Basal	4.37 (3.68-5.63)	5.46 (4.86-6.21)	4.39 (4.3-6.09)	0.5040	0.9712	3.41 (2.79-4.51)	3.86 (3.04-4.73)	3.45 (3.14-5.06)	0.8990	1
IFNα	1.02 (0.978-1.03)	1.04 (0.989-1.13)	1 (0.943-1.25)	0.8740	0.9969	0.998 (0.963-1.27)	1.11 (1.03-1.25)	1.07 (0.983-1.16)	0.7340	0.9098
LPS (TLR4)	1.01 (0.974-1.07)	1 (0.968-1.03)	1 (0.967-1.06)	0.9870	0.9969	1.02 (1-1.03)	1 (0.958-1.03)	0.989 (0.973-1.04)	0.7700	0.9109
R848 (TLR7/8)	1.04 (0.965-1.09)	0.985 (0.907-0.998)	0.993 (0.924-1.07)	0.2700	0.8817	1.03 (1.01-1.13)	0.968 (0.948-0.996)	1.01 (0.953-1.04)	0.0893	0.3746
CpG (TLR9)	1.01 (0.97-1.05)	0.899 (0.85-0.965)	0.993 (0.979-1.01)	**0.0296**	0.7299	1.08 (1.01-1.21)	0.958 (0.898-1.01)	0.971 (0.937-1.04)	0.0590	0.2925
PMA-Iono	4.78 (3.32-6.94)	3.13 (2.29-3.65)	3.58 (1.74-4.74)	0.4900	0.9712	8.2 (3.21-10.7)	4.75 (3.23-5.89)	4.61 (4.14-6.16)	0.6050	0.8683
**p-p38**										
Basal	3.94 (3.4-5.57)	4.63 (3.8-5.23)	4.46 (3.76-4.95)	0.8000	0.9969	3.06 (2.91-4.19)	3.06 (2.75-4.1)	3.59 (3.3-4.39)	0.5220	0.8683
IFNα	1.05 (0.98-1.12)	1.03 (0.959-1.25)	1.04 (1.01-1.34)	0.8900	0.9969	1.08 (1.03-1.12)	1.2 (1.13-1.31)	1.03 (0.973-1.14)	0.0518	0.2925
LPS (TLR4)	1.11 (1.03-1.19)	1.16 (1.08-1.18)	1.08 (1.02-1.15)	0.5090	0.9712	1.14 (1.07-1.18)	1.13 (1.09-1.18)	1.13 (1.04-1.14)	0.6880	0.9098
R848 (TLR7/8)	1.09 (1.06-1.15)	1.04 (0.985-1.07)	1.02 (1.01-1.05)	0.1430	0.8817	1.11 (1.06-1.16)	1.08 (1.03-1.09)	1.01 (0.97-1.14)	0.3900	0.8275
CpG (TLR9)	1.03 (0.967-1.15)	0.964 (0.896-1.02)	0.957 (0.917-0.991)	0.0577	0.7299	1.07 (1.03-1.19)	0.962 (0.893-1.01)	0.967 (0.935-1.04)	**0.0096**	0.1745
PMA-Iono	3.29 (2.57-4.07)	1.96 (1.57-2.71)	2.5 (1.76-2.85)	0.2740	0.8817	3.29 (2.25-4.45)	2.62 (1.93-3.06)	2.31 (1.61-3)	0.2690	0.6986
**p-PLCg2**										
Basal	1.47 (1.38-1.76)	1.7 (1.57-2.14)	1.94 (1.72-2.69)	0.3190	0.8817	1.34 (1.26-1.48)	1.44 (1.28-1.86)	1.36 (1.17-2.27)	0.6700	0.9098

IFNα	1.27 (1.12-1.3)	1.47 (1.31-1.61)	1.38 (1.1-1.55)	0.2230	0.8817	1.5 (1.4-1.54)	1.43 (1.29-1.63)	1.22 (1.06-1.53)	0.2270	0.6746
LPS (TLR4)	1 (0.956-1.04)	0.996 (0.926-1.08)	0.9 (0.861-1.03)	0.6690	0.9969	1.01 (1-1.05)	1.04 (0.978-1.06)	1.07 (0.957-1.1)	0.7160	0.9098
R848 (TLR7/8)	1.03 (0.97-1.11)	1 (0.817-1.03)	0.903 (0.892-1.06)	0.3880	0.8817	1.13 (1.01-1.3)	0.979 (0.933-1)	1.02 (0.929-1.08)	0.0806	0.3663
CpG (TLR9)	1.03 (0.939-1.06)	0.983 (0.881-1.04)	1 (0.822-1.01)	0.2630	0.8817	1.15 (1.03-1.25)	1.02 (0.942-1.11)	1.03 (0.95-1.05)	0.0544	0.2925
PMA-Iono	1.09 (0.971-1.21)	1.12 (1.03-1.23)	1.04 (1.02-1.36)	0.9820	0.9969	1.11 (1.09-1.18)	1.17 (1.12-1.35)	1.19 (0.961-1.3)	0.6550	0.9098
**pSTAT1**										
Basal	13.6 (10.2-23.2)	24.6 (15.9-37.1)	20.1 (14.7-31.9)	0.3870	0.8817	23.7 (16.6-32.3)	25.7 (18.3-28.6)	26.5 (15.1-34.6)	0.9800	1
IFNα	7.67 (3.7-8.99)	3.72 (2.76-8.09)	3.12 (1.92-5.07)	0.3310	0.8817	4.29 (3.32-5.95)	3.56 (2.13-5.07)	2.84 (1.69-5.07)	0.5640	0.8683
LPS (TLR4)	0.974 (0.898-1.12)	1.05 (0.94-1.09)	0.974 (0.906-1.03)	0.5520	0.9712	1.07 (0.925-1.14)	1.15 (1.06-1.2)	1.03 (0.946-1.14)	0.4020	0.8275
R848 (TLR7/8)	0.978 (0.84-1.13)	0.987 (0.898-1.07)	1.01 (0.774-1.14)	0.5720	0.9749	1.06 (0.906-1.23)	1.02 (0.915-1.12)	1.06 (0.96-1.48)	0.9940	1
CpG (TLR9)	0.88 (0.68-1.19)	0.993 (0.97-1.22)	0.968 (0.82-1.03)	0.9500	0.9969	1.04 (0.871-1.16)	0.994 (0.819-1.22)	1 (0.908-1.25)	0.6020	0.8683
PMA-Iono	1.23 (1.06-1.46)	1.2 (1-1.38)	0.99 (0.848-1.53)	0.9110	0.9969	1.15 (0.965-1.49)	1.38 (0.985-1.57)	1.36 (1.03-1.76)	0.8860	1
**pSTAT3**										
Basal	10.1 (6.14-13.7)	11.3 (9.72-12.4)	12.1 (6.58-17.3)	0.8800	0.9969	13.3 (10-15.9)	8.77 (6.58-12.2)	8.56 (7.3-15.2)	0.5380	0.8683
IFNα	4.31 (3.97-6.07)	2.97 (2.3-4.93)	2.24 (0.922-7.38)	0.3290	0.8817	3.47 (2.47-5.98)	3.08 (1.73-5.79)	2.57 (1.17-3.68)	0.3830	0.8275
LPS (TLR4)	1.01 (0.973-1.04)	1 (0.934-1.08)	1.05 (0.889-1.09)	0.8250	0.9969	0.984 (0.92-1.03)	1.03 (0.999-1.11)	0.96 (0.945-1.02)	0.1180	0.4363
R848 (TLR7/8)	1.01 (0.958-1.05)	0.966 (0.911-1.04)	1.14 (1.01-1.44)	0.1130	0.8804	0.995 (0.91-1.04)	0.995 (0.93-1.04)	0.956 (0.859-1.03)	0.5460	0.8683
CpG (TLR9)	1.03 (0.991-1.05)	0.943 (0.913-1.08)	1.03 (0.968-1.15)	0.7360	0.9969	0.951 (0.867-0.972)	1.07 (0.987-1.24)	0.951 (0.891-0.99)	0.0513	0.2925

PMA-Iono	0.903 (0.863-0.952)	0.883 (0.836-0.928)	0.999 (0.919-1.14)	0.2520	0.8817	0.841 (0.722-0.968)	0.962 (0.888-0.989)	0.857 (0.808-0.91)	0.2530	0.6899
**pSTAT5**										
Basal	6.66 (5.5-7.73)	10.3 (8.21-12)	12.3 (8.39-15.3)	**0.0225**	0.7299	5.4 (5.18-10)	9 (6.5-10.9)	8.67 (6.95-16.3)	0.5190	0.8683
IFNα	4.33 (2.75-5.68)	3.6 (3.05-5.3)	1.97 (1.5-4.09)	0.3770	0.8817	6.89 (5.21-11.7)	4.83 (3.2-7.34)	1.72 (1.23-5.35)	**0.0283**	0.2925
LPS (TLR4)	1.02 (0.983-1.03)	0.977 (0.937-1.01)	0.917 (0.889-1.09)	0.3640	0.8817	1.03 (0.987-1.04)	1.02 (1.01-1.06)	1.02 (0.913-1.05)	0.7850	0.9109
R848 (TLR7/8)	0.992 (0.951-1.01)	0.964 (0.92-0.977)	1 (0.868-1.1)	0.4350	0.949	0.999 (0.959-1.1)	0.995 (0.946-1.05)	1.01 (0.91-1.06)	0.9200	1
CpG (TLR9)	1.01 (0.976-1.04)	0.929 (0.896-0.959)	0.956 (0.886-1.05)	0.2780	0.8817	1.07 (0.987-1.16)	1.03 (0.978-1.1)	1 (0.87-1.09)	0.4400	0.8275
PMA-Iono	1.04 (1.01-1.07)	1.07 (1.03-1.1)	1.04 (0.994-1.16)	0.8460	0.9969	1.11 (1.02-1.17)	1.18 (1.14-1.36)	1.14 (1.03-1.32)	0.2350	0.6746
**Syk**										
Basal	6.13 (5.98-6.96)	6.36 (5.48-7.53)	6.59 (5.62-7.88)	0.8570	0.9969	6.55 (6.33-7.41)	6.54 (5.73-7.22)	6.24 (5.74-7.04)	0.7230	0.9098
IFNα	1.02 (1.01-1.03)	1.12 (1.06-1.22)	1.42 (1.03-1.5)	0.0577	0.7299	1.11 (0.993-1.21)	1.19 (1.09-1.53)	1.15 (1.07-1.23)	0.4400	0.8275
LPS (TLR4)	1.03 (1.02-1.08)	1.03 (1-1.1)	0.997 (0.931-1.12)	0.7510	0.9969	1.07 (1.04-1.1)	1.04 (1.01-1.12)	1.02 (1.01-1.06)	0.4300	0.8275
R848 (TLR7/8)	1.02 (0.998-1.14)	1.01 (0.921-1.04)	1.04 (0.887-1.18)	0.6180	0.9969	1.14 (1.05-1.24)	1.01 (0.974-1.02)	1.05 (0.99-1.09)	**0.0412**	0.2925
CpG (TLR9)	1.02 (0.924-1.09)	0.964 (0.919-1)	1.04 (0.898-1.06)	0.6800	0.9969	1.19 (1.06-1.24)	0.991 (0.937-1.09)	0.986 (0.979-1.03)	**0.0119**	0.1745
PMA-Iono	1.04 (0.997-1.1)	1.11 (1.08-1.17)	1.2 (1.06-1.77)	0.2000	0.8817	1.23 (1.14-1.29)	1.27 (1.12-1.31)	1.21 (1.15-1.34)	0.9510	1

Supplemental Table 15. Fold change in phospho-proteins following CD8+ T cell stimulation with various stimuli. Median metal intensity and IQR shown for unstimulated/basal conditions. Median fold change and IQR over the unstimulated/basal conditions shown for all other stimuli.

CD8+ T Cells										
	Median (Interquartile Range)					Median (Interquartile Range)				
	**White Controls**	**White SLE INACT**	**White SLE ACT**	**KW p-value**	**FDR q-value**	**Black Controls**	**Black SLE INACT**	**Black SLE ACT**	**KW p-value**	**FDR q-value**
**cCASP3**										
Basal	5.36 (5.28-5.78)	5.25 (4.9-7.3)	6.72 (5.34-7.62)	0.3850	0.9712	5.15 (4.52-5.49)	5.56 (5.04-6.54)	5.57 (4.79-11.2)	0.3870	0.6627
IFNα	1 (1-1.1)	1.21 (1.18-1.37)	1.31 (0.971-1.76)	0.1840	0.9712	1.14 (1.01-1.39)	1.52 (1.18-1.65)	1.31 (1.12-1.33)	0.2590	0.6421
LPS (TLR4)	0.998 (1-1.09)	1.04 (1.03-1.2)	0.963 (0.902-1.02)	0.3600	0.9712	1.04 (1.01-1.06)	1.07 (1-1.12)	1.03 (1.01-1.04)	0.6510	0.8493
R848 (TLR7/8)	0.962 (0.998-1.05)	1.04 (1.01-1.07)	0.964 (0.876-1.06)	0.7700	0.9712	1.06 (1.01-1.2)	1.01 (0.976-1.04)	1.01 (0.994-1.07)	0.3120	0.6627
CpG (TLR9)	1.04 (1.02-1.1)	0.993 (1-1.08)	0.978 (0.975-1.05)	0.9140	0.9712	1.21 (1.05-1.51)	1.01 (0.946-1.07)	1.03 (0.961-1.09)	0.0573	0.3094
PMA-Iono	1.05 (1.04-1.11)	1.1 (1.1-1.19)	1.15 (1.07-1.42)	0.2410	0.9712	1.16 (1.12-1.21)	1.14 (1.09-1.42)	1.25 (1.12-1.33)	0.8720	0.9482
**pCREB**										
Basal	16 (15.7-20.4)	14.5 (13.7-17.8)	15.9 (11.5-20.3)	0.6900	0.9712	12.3 (9.33-15.6)	14.7 (11.6-22.3)	9.38 (8.24-17.5)	0.2220	0.6054
IFNα	1.04 (1.04-1.11)	1.09 (1.09-1.41)	1.08 (0.824-1.45)	0.4540	0.9712	1.2 (1.04-1.4)	1.11 (1.02-1.51)	1.15 (1.1-1.24)	0.8840	0.9482
LPS (TLR4)	0.989 (1-1.06)	1.06 (1.05-1.14)	1.16 (0.975-1.18)	0.2350	0.9712	1.08 (1.05-1.15)	1.05 (1.01-1.14)	1.08 (1.04-1.17)	0.6540	0.8493
R848 (TLR7/8)	0.939 (0.951-1.04)	0.957 (0.96-1)	1.07 (0.969-1.12)	0.2360	0.9712	1.14 (1.11-1.22)	1.01 (0.919-1.06)	1.08 (1.04-1.12)	**0.0072**	0.1023
CpG (TLR9)	0.977 (0.996-1.04)	0.978 (0.999-1.07)	1.03 (1.02-1.19)	0.7430	0.9712	1.23 (1.13-1.38)	1.04 (0.882-1.08)	1.11 (0.99-1.14)	**0.0131**	0.1236

PMA-Iono	1.83 (1.77-2.43)	2.07 (1.99-3.03)	2.41 (1.72-2.99)	0.7820	0.9712	3.08 (2.9-3.74)	2.8 (1.88-3.06)	3.14 (2.21-5.16)	0.3100	0.6627
**pERK1/2**										
Basal	4.07 (3.93-5.31)	4.6 (4.28-6.12)	4.11 (3.79-5.66)	0.6280	0.9712	2.91 (2.3-4.19)	3.34 (2.66-3.99)	3.08 (2.64-4.51)	0.9040	0.9482
IFNα	1 (1-1.07)	1.01 (1-1.07)	0.971 (0.929-1.15)	0.9060	0.9712	0.994 (0.93-1.12)	1.11 (1.06-1.2)	1.05 (0.982-1.13)	0.2490	0.6421
LPS (TLR4)	1.04 (1.02-1.11)	1 (1-1.09)	1 (0.967-1.01)	0.3690	0.9712	1.02 (0.998-1.06)	1.02 (1.01-1.06)	1.02 (0.955-1.06)	0.8960	0.9482
R848 (TLR7/8)	0.998 (1-1.04)	1 (1-1.03)	0.95 (0.91-0.98)	0.2480	0.9712	1.06 (1.01-1.08)	0.99 (0.951-1.03)	1.02 (0.968-1.05)	0.1780	0.5636
CpG (TLR9)	1.03 (1.03-1.05)	0.935 (0.944-1.01)	0.979 (0.914-1)	**0.0173**	0.9435	1.09 (1.01-1.23)	0.991 (0.901-1.08)	1 (0.932-1.03)	0.0624	0.3094
PMA-Iono	4.26 (3.74-6.25)	2.62 (2.51-3.44)	3.25 (1.73-4.06)	0.5550	0.9712	6.54 (3.29-9.58)	4.7 (3.21-5.56)	3.91 (3.1-5.52)	0.5920	0.8493
**p-p38**										
Basal	3.88 (3.84-6.8)	4.74 (4.58-5.4)	4.06 (4.02-5.3)	0.9090	0.9712	2.91 (2.78-4.01)	2.94 (2.58-4.01)	3.43 (3.09-4.08)	0.5400	0.8415
IFNα	1.08 (1.07-1.12)	1.04 (1.03-1.1)	1.03 (1-1.11)	0.7880	0.9712	1.03 (0.908-1.13)	1.16 (1.13-1.18)	1.02 (0.97-1.13)	0.0862	0.3616
LPS (TLR4)	1.25 (1.23-1.34)	1.12 (1.1-1.21)	1.12 (1.11-1.23)	0.4590	0.9712	1.07 (1.03-1.13)	1.08 (1.06-1.14)	1.07 (1.01-1.1)	0.8270	0.9482
R848 (TLR7/8)	1.05 (1.04-1.14)	1.02 (1-1.11)	1.08 (0.941-1.1)	0.7980	0.9712	1.12 (1.08-1.17)	1.04 (0.991-1.08)	0.978 (0.967-1.09)	0.0804	0.3616
CpG (TLR9)	1 (1-1.09)	0.976 (1-1.05)	1.02 (0.973-1.03)	0.8960	0.9712	1.1 (1.03-1.13)	1.02 (0.973-1.06)	0.968 (0.933-1.01)	**0.0136**	0.1236
PMA-Iono	2.82 (2.64-3.82)	1.85 (1.83-2.93)	2.14 (1.72-3.89)	0.4150	0.9712	2.9 (2.12-4.39)	2.18 (1.78-2.64)	2.22 (1.59-2.97)	0.3420	0.6627
**p-PLCg2**										
Basal	1.52 (1.49-1.86)	1.87 (1.87-2.53)	1.78 (1.53-2.71)	0.4400	0.9712	1.33 (1.25-1.51)	1.66 (1.26-2.06)	1.53 (1.25-2.79)	0.3830	0.6627
IFNα	1.19 (1.18-1.29)	1.15 (1.12-1.51)	1.16 (1.05-1.41)	0.9460	0.9716	1.37 (1.27-1.52)	1.38 (1.15-1.48)	1.1 (0.856-1.23)	0.0560	0.3094

LPS (TLR4)	1.06 (1.06-1.22)	0.992 (0.995-1.01)	1.02 (0.935-1.14)	0.0565	0.9712	1.05 (1.03-1.09)	1.02 (0.952-1.11)	0.976 (0.827-1.02)	0.1610	0.5636
R848 (TLR7/8)	1.02 (1.01-1.06)	0.916 (0.923-1.02)	1.01 (0.946-1.08)	0.2010	0.9712	1.14 (1.05-1.23)	1.04 (0.936-1.06)	0.979 (0.854-1.01)	**0.0075**	0.1023
CpG (TLR9)	0.978 (0.994-1.02)	0.979 (0.989-1.1)	1.01 (0.985-1.07)	0.8150	0.9712	1.15 (1.11-1.36)	0.957 (0.904-1.04)	0.926 (0.837-0.986)	**0.0029**	0.1023
PMA-Iono	1.08 (1.05-1.22)	1.06 (1.03-1.28)	1.14 (1.04-1.22)	0.5550	0.9712	1.2 (1.14-1.27)	1.27 (1.14-1.39)	1.2 (1.04-1.27)	0.3910	0.6627
**pSTAT1**										
Basal	10.7 (10-15.4)	19.1 (19-23.6)	15.2 (13.4-18.9)	0.2920	0.9712	13.9 (11-20.6)	14.5 (8.95-18.3)	19.4 (10.3-24.8)	0.8250	0.9482
IFNα	7.36 (7.2-7.64)	4.03 (3.72-8.16)	2.38 (2.05-3.85)	0.2200	0.9712	5.83 (4.3-8.37)	4.13 (2.85-9.16)	3.29 (1.91-5.19)	0.3530	0.6627
LPS (TLR4)	0.985 (0.994-1.07)	1.02 (1.01-1.1)	0.964 (0.928-1.04)	0.7950	0.9712	1.03 (0.933-1.1)	1.07 (1.06-1.19)	1.01 (0.986-1.18)	0.4560	0.7315
R848 (TLR7/8)	0.979 (0.988-1.07)	0.988 (0.989-1.04)	1.02 (0.842-1.08)	0.4760	0.9712	1.08 (0.966-1.15)	1.02 (0.972-1.14)	1.05 (0.917-1.28)	0.7070	0.8764
CpG (TLR9)	0.962 (1-1.05)	1 (1-1.09)	0.919 (0.872-1.01)	0.9260	0.9712	1.01 (0.935-1.03)	0.997 (0.893-1.17)	1.08 (0.92-1.15)	0.8620	0.9482
PMA-Iono	1.19 (1.18-1.28)	1.19 (1.17-1.26)	1.07 (0.974-1.53)	0.9620	0.9716	1.15 (0.935-1.37)	1.38 (0.968-1.68)	1.27 (1.12-1.73)	0.6430	0.8493
**pSTAT3**										
Basal	5.82 (4.89-8.59)	6.63 (6.34-8.64)	6.42 (5.13-10.1)	0.7110	0.9712	7.69 (3.98-10)	6.25 (4.51-8.72)	6.55 (5.48-8.52)	0.8620	0.9482
IFNα	6.28 (5.9-8.15)	3.95 (3.84-7.16)	2.36 (1.62-10.6)	0.4290	0.9712	5.22 (3.84-11.3)	3.72 (3.03-7.75)	2.88 (1.46-6.48)	0.3420	0.6627
LPS (TLR4)	0.994 (1-1.03)	1.02 (1-1.15)	1 (0.908-1.07)	0.5660	0.9712	0.961 (0.916-1.02)	1.03 (0.999-1.07)	0.992 (0.941-1.04)	0.1170	0.4558
R848 (TLR7/8)	0.988 (1-1.11)	1.02 (1.01-1.04)	1.07 (1-1.09)	0.7660	0.9712	0.963 (0.853-0.985)	1 (0.93-1.09)	0.947 (0.932-1)	0.3110	0.6627
CpG (TLR9)	1.01 (1-1.05)	1 (1-1.06)	1 (0.959-1.03)	0.9030	0.9712	0.93 (0.905-1.02)	1.08 (0.979-1.22)	0.95 (0.876-0.979)	**0.0404**	0.2754
PMA-Iono	0.945 (0.948-0.959)	0.962 (0.967-1.06)	0.997 (0.966-1.22)	0.1880	0.9712	0.908 (0.809-0.976)	1 (0.958-1.04)	0.936 (0.845-1)	0.1690	0.5636

**pSTAT5**										
Basal	6.2 (5.7-8.18)	8.26 (8.14-13)	12.9 (6.53-14.3)	0.2420	0.9712	5.17 (5-8.74)	8.97 (7.19-12.9)	7.48 (6.63-18.3)	0.4010	0.6627
IFNα	3.17 (2.95-3.58)	2.94 (2.8-4.66)	1.95 (1.3-2.28)	0.2960	0.9712	4.72 (3.66-7.61)	3.38 (2.16-4.89)	1.35 (1.19-3.16)	**0.0268**	0.2088
LPS (TLR4)	0.988 (0.995-1.07)	0.994 (1-1.05)	1.02 (0.928-1.13)	0.8570	0.9712	1.03 (0.983-1.07)	1.03 (0.965-1.07)	1 (0.931-1.04)	0.6350	0.8493
R848 (TLR7/8)	0.983 (0.984-0.991)	0.989 (1-1.02)	1.02 (0.904-1.07)	0.3820	0.9712	0.979 (0.941-1.18)	0.962 (0.946-0.981)	1.02 (0.941-1.11)	0.6280	0.8493
CpG (TLR9)	0.956 (0.979-1.01)	0.985 (0.986-0.996)	0.994 (0.811-1.04)	0.8900	0.9712	0.987 (0.901-1.13)	0.974 (0.944-1.06)	1.01 (0.918-1.04)	0.9830	0.9989
PMA-Iono	1.03 (1.03-1.08)	1.12 (1.11-1.15)	1.18 (1.05-1.27)	0.1000	0.9712	1.12 (0.934-1.18)	1.18 (1.1-1.28)	1.24 (1.08-1.37)	0.5880	0.8493
**Syk**										
Basal	6.77 (6.76-7.56)	7.4 (7.16-8.16)	7.94 (6.19-8.15)	0.8740	0.9712	7.08 (6.67-8.42)	6.88 (6.27-7.46)	6.89 (6.3-8.02)	0.6780	0.86
IFNα	1.01 (1-1.12)	1.24 (1.2-1.32)	1.25 (1.07-1.31)	0.0798	0.9712	1.14 (1.02-1.33)	1.27 (1.11-1.58)	1.13 (1.1-1.22)	0.3750	0.6627
LPS (TLR4)	1.04 (1.02-1.1)	1.04 (1.03-1.08)	1.05 (0.882-1.13)	0.8360	0.9712	1.08 (1.06-1.09)	1.05 (0.997-1.13)	1.04 (1.02-1.05)	0.2210	0.6054
R848 (TLR7/8)	0.974 (0.977-1.05)	0.958 (0.962-0.994)	0.985 (0.885-1.05)	0.9160	0.9712	1.06 (1.04-1.22)	1 (0.964-1.06)	1.01 (0.992-1.07)	0.1860	0.5636
CpG (TLR9)	1.01 (1-1.08)	0.987 (0.996-1.03)	0.948 (0.893-1.02)	0.7350	0.9712	1.22 (1.07-1.31)	0.974 (0.957-1.05)	0.997 (0.963-1.05)	**0.0047**	0.1023
PMA-Iono	1.07 (1.04-1.12)	1.16 (1.15-1.21)	1.07 (1.04-1.47)	0.4800	0.9712	1.26 (1.18-1.29)	1.24 (1.13-1.33)	1.16 (1.15-1.33)	0.9890	0.9989

Supplemental Table 16. **Fold change in phospho-proteins following dendritic cell stimulation with various stimuli.** Median metal intensity and IQR shown for unstimulated/basal conditions. Median fold change and IQR over the unstimulated/basal conditions shown for all other stimuli.

Dendritic Cells										
	Median (Interquartile Range)					Median (Interquartile Range)				
	**White Controls**	**White SLE INACT**	**White SLE ACT**	**KW p-value**	**FDR q-value**	**Black Controls**	**Black SLE INACT**	**Black SLE ACT**	**KW p-value**	**FDR q-value**
**cCASP3**										
Basal	15.6 (10.9-25.4)	17 (11.8-21.1)	11.3 (8.02-16.6)	0.1280	0.6121	11.7 (10.8-15.1)	19.5 (12.6-24)	20.1 (9.59-28.5)	0.7100	0.9303
IFNα	0.914 (0.842-1.06)	1.01 (0.812-1.22)	1.12 (1.08-1.77)	0.1980	0.6121	1.05 (0.843-1.41)	0.983 (0.762-1.38)	1.25 (1.04-1.88)	0.3090	0.8426
LPS (TLR4)	0.879 (0.715-0.997)	1.02 (0.637-1.14)	1.03 (0.88-1.51)	0.5260	0.9766	1.08 (0.964-1.3)	1.08 (0.818-1.18)	0.863 (0.768-1.22)	0.8540	0.9303
R848 (TLR7/8)	0.952 (0.873-1.34)	0.81 (0.65-0.843)	1.22 (0.861-1.32)	**0.0173**	0.6121	1.2 (0.576-1.34)	0.97 (0.832-1.36)	0.994 (0.87-1.2)	0.9500	0.9736
CpG (TLR9)	0.909 (0.72-1.06)	0.868 (0.792-0.984)	0.957 (0.801-1.37)	0.4620	0.9691	1.27 (0.88-1.32)	0.928 (0.812-1.15)	0.785 (0.732-0.975)	0.1580	0.6288
PMA-Iono	0.822 (0.759-0.95)	0.925 (0.746-1.14)	1.38 (1.03-2.43)	**0.0289**	0.6121	1.39 (1.29-1.48)	1.03 (0.618-1.22)	1.19 (0.853-1.52)	0.1960	0.6288
**pCREB**										
Basal	50.7 (42.6-83.6)	60.5 (42.1-71.7)	62.3 (46.5-71.4)	0.9700	0.9797	56.6 (43.8-68.7)	63 (45.8-102)	58.7 (44.4-77.1)	0.7580	0.9303
IFNα	1.01 (0.895-1.22)	1.25 (1.18-1.72)	0.873 (0.793-1.38)	0.1380	0.6121	1.1 (0.988-1.36)	1.15 (0.909-1.28)	1.15 (1.07-1.68)	0.6130	0.9303
LPS (TLR4)	1.07 (0.957-1.36)	1.37 (1.15-1.49)	1.07 (0.905-1.41)	0.6830	0.9766	1.29 (1.24-1.58)	1.22 (1.03-1.33)	1.21 (1.09-1.29)	0.3840	0.9022
R848 (TLR7/8)	1.01 (0.904-1.26)	1.11 (0.973-1.24)	1.08 (0.742-1.27)	0.9110	0.9766	1.51 (1.36-1.75)	1.04 (0.922-1.2)	1.24 (1.07-1.38)	**0.0216**	0.589
CpG (TLR9)	0.89 (0.85-1.14)	0.89 (0.764-0.951)	0.976 (0.804-1.06)	0.8760	0.9766	1.07 (1-1.23)	0.983 (0.783-1.16)	1.07 (0.921-1.18)	0.4350	0.9184

PMA-Iono	1.29 (1.24-1.47)	1.46 (1.37-1.63)	1.38 (1.29-1.61)	0.1920	0.6121	1.75 (1.59-1.98)	1.54 (1.18-1.61)	1.87 (1.58-1.97)	0.1180	0.6288
**pERK1/2**										
Basal	10 (7.92-119)	17.4 (13.2-111)	10.7 (8.12-40.8)	0.4520	0.9691	9.84 (6.7-20)	9.33 (6.43-12.8)	9.24 (5.38-13.4)	0.7010	0.9303
IFNα	1.18 (1.06-1.52)	1.31 (1.19-2.51)	1.5 (0.949-1.84)	0.4370	0.9691	1.36 (0.96-1.85)	2.17 (1.78-2.93)	1.31 (0.922-3.37)	0.5130	0.9184
LPS (TLR4)	1.24 (1-1.54)	1.92 (1.5-2.35)	1.47 (0.976-2.34)	0.1290	0.6121	3.48 (1.74-5.88)	2.4 (1.41-4.21)	4.16 (1.45-5.41)	0.8030	0.9303
R848 (TLR7/8)	1.4 (0.976-1.62)	1.67 (0.956-2.23)	1.25 (1.07-1.32)	0.9180	0.9766	3.51 (1.57-5.69)	2.49 (1.77-4.87)	2.53 (1.98-7.34)	0.8850	0.9303
CpG (TLR9)	1.03 (0.864-1.23)	0.624 (0.357-0.923)	1.04 (0.645-1.13)	0.0792	0.6121	1.38 (0.631-1.73)	0.85 (0.746-1.32)	1.07 (0.945-1.75)	0.6480	0.9303
PMA-Iono	4.37 (2.41-5.81)	3.57 (2.19-3.99)	3.97 (2.99-5.24)	0.5720	0.9766	5.45 (4.09-9.61)	6.79 (4.77-8.03)	6.2 (3.56-12.5)	0.8610	0.9303
**p-p38**										
Basal	21.4 (14.1-58)	23.5 (17.7-44.5)	28.8 (21.5-36.1)	0.8530	0.9766	18.2 (16.8-28.2)	23.9 (14.2-34.4)	31.8 (23.5-44.4)	0.1790	0.6288
IFNα	1.19 (0.8-1.31)	1.15 (0.815-2.07)	1.19 (0.908-1.52)	0.8800	0.9766	1.05 (0.859-1.36)	0.901 (0.626-1.23)	1.02 (0.527-1.47)	0.7860	0.9303
LPS (TLR4)	3.03 (2.12-3.9)	2.95 (2.67-3.64)	2.47 (2.06-3.09)	0.5020	0.9766	3.66 (2.33-6.1)	2.66 (2.11-3.84)	2.27 (2-2.72)	0.2310	0.6999
R848 (TLR7/8)	2.6 (2.32-3.21)	2.94 (2.37-3.96)	2.82 (2.23-3.38)	0.7500	0.9766	4.05 (2.85-6.33)	2.96 (1.98-3.85)	2.47 (2.05-2.9)	0.1220	0.6288
CpG (TLR9)	1.17 (0.948-1.55)	0.849 (0.608-1.33)	0.801 (0.628-1.08)	0.1990	0.6121	1.19 (1.04-1.35)	0.852 (0.547-1.13)	0.932 (0.523-1.23)	0.1950	0.6288
PMA-Iono	2.24 (1.69-2.6)	2.51 (1.87-3.36)	2.2 (1.72-2.48)	0.7090	0.9766	3.09 (2.01-3.37)	1.87 (1.25-2.87)	1.59 (1.11-2.03)	0.0653	0.6288
**p-PLCg2**										
Basal	20.6 (12.8-23.4)	16.4 (13.5-18.7)	16.5 (15.1-17.9)	0.3180	0.7541	15.7 (14.8-17.8)	12.3 (10.5-12.6)	11.7 (9.28-14.3)	**0.0352**	0.6288
IFNα	1.03 (0.919-1.15)	1.18 (0.921-1.48)	1.27 (0.781-1.35)	0.8800	0.9766	0.93 (0.912-0.957)	1.06 (0.997-1.27)	1.2 (0.816-1.46)	0.0652	0.6288

LPS (TLR4)	1.09 (0.977-1.19)	0.937 (0.888-1.06)	0.832 (0.765-1)	0.1360	0.6121	0.963 (0.806-1.05)	1.1 (0.987-1.38)	1.09 (0.816-1.21)	0.1420	0.6288
R848 (TLR7/8)	0.929 (0.839-1.05)	0.986 (0.96-1.03)	0.872 (0.749-0.938)	0.0817	0.6121	0.941 (0.903-1.04)	1.14 (0.958-1.28)	0.886 (0.81-1.22)	0.1550	0.6288
CpG (TLR9)	0.867 (0.713-1.12)	0.96 (0.927-0.981)	0.952 (0.699-1.01)	0.8430	0.9766	1.03 (1.02-1.17)	1.2 (0.983-1.32)	1.18 (0.804-1.2)	0.5060	0.9184
PMA-Iono	0.874 (0.852-0.925)	0.896 (0.796-1.24)	0.886 (0.633-1.06)	0.8900	0.9766	0.775 (0.663-0.964)	0.979 (0.774-1.13)	0.96 (0.836-1.04)	0.5020	0.9184
**pSTAT1**										
Basal	21.8 (18.5-32.6)	27.6 (11.4-31.7)	25.6 (20.8-42.5)	0.7190	0.9766	27.7 (25.2-37.2)	26.3 (23.1-33.6)	33.2 (27.2-36.8)	0.5140	0.9184
IFNα	3.81 (3.63-4.73)	8.01 (4.04-11.6)	2.67 (1.34-4.89)	0.1490	0.6121	3.18 (2.74-3.62)	3.78 (3.34-4.88)	4.23 (1.98-6.23)	0.7320	0.9303
LPS (TLR4)	0.922 (0.683-1.03)	1.06 (0.964-1.29)	0.967 (0.355-1.06)	0.1850	0.6121	0.961 (0.895-1.03)	1.23 (0.98-1.32)	1.05 (0.91-1.13)	0.1720	0.6288
R848 (TLR7/8)	0.757 (0.644-1.03)	0.925 (0.843-1.02)	0.91 (0.439-1.05)	0.1400	0.6121	0.985 (0.901-1.12)	1.16 (0.945-1.53)	0.887 (0.855-1.03)	0.1910	0.6288
CpG (TLR9)	0.827 (0.674-1.01)	0.979 (0.897-1.18)	0.822 (0.394-1.04)	0.4930	0.9766	0.865 (0.816-0.994)	1.02 (0.903-1.36)	1.07 (0.979-1.08)	0.1660	0.6288
PMA-Iono	1.01 (0.794-1.22)	1.31 (1.13-1.55)	1.04 (0.788-1.15)	0.1700	0.6121	1.06 (0.955-1.15)	1.24 (0.975-1.49)	1.19 (0.929-1.36)	0.7860	0.9303
**pSTAT3**										
Basal	11.6 (8.99-15)	15.4 (11-20.7)	15.3 (9.13-19.9)	0.5450	0.9766	12.2 (7.92-16.7)	9.95 (6.65-13.3)	11 (7.82-13.8)	0.6860	0.9303
IFNα	6.31 (4.94-7.26)	4.08 (2.27-6.67)	1.45 (0.971-8.38)	0.3050	0.7541	7.18 (3.29-10.2)	4.47 (2.76-5.83)	2.74 (1.31-3.82)	0.1150	0.6288
LPS (TLR4)	1.14 (0.851-1.18)	0.995 (0.803-1.11)	1.09 (0.975-1.16)	0.7380	0.9766	1.03 (0.96-1.14)	1.16 (0.912-1.41)	0.974 (0.781-1.07)	0.3030	0.8426
R848 (TLR7/8)	0.831 (0.705-1.1)	0.97 (0.907-1.12)	0.951 (0.659-1.29)	0.7880	0.9766	1.06 (0.937-1.29)	1.01 (0.879-1.25)	1.11 (1.04-1.22)	0.8070	0.9303
CpG (TLR9)	0.998 (0.755-1.06)	1.02 (0.841-1.18)	0.856 (0.773-1.07)	0.7510	0.9766	1.03 (0.879-1.15)	1.1 (0.847-1.2)	0.983 (0.833-1.28)	0.9640	0.9736
PMA-Iono	0.913 (0.804-0.99)	0.98 (0.905-1.21)	0.998 (0.76-1.2)	0.7560	0.9766	0.939 (0.787-1.04)	1.05 (0.824-1.22)	1.09 (1.01-1.18)	0.3660	0.9022

**pSTAT5**										
Basal	15.1 (14.1-17.8)	15.3 (13.1-20)	17.4 (14.6-18.5)	0.7520	0.9766	15.8 (13.3-19.1)	15.1 (12.7-23.9)	20.3 (16.2-22.2)	0.3280	0.8519
IFNα	2.74 (2.07-3.46)	3.42 (2.07-4.7)	1.36 (1.24-3.61)	0.2940	0.7541	4.54 (4.23-5.57)	2.14 (1.54-4.56)	1.88 (1.35-2.32)	**0.0195**	0.589
LPS (TLR4)	0.972 (0.786-1.18)	0.985 (0.798-1.17)	0.957 (0.67-0.971)	0.3100	0.7541	0.967 (0.914-1.03)	1.04 (0.894-1.38)	0.973 (0.692-1.15)	0.5440	0.9272
R848 (TLR7/8)	0.97 (0.912-1.15)	0.919 (0.787-1.21)	1.02 (0.818-1.13)	0.8800	0.9766	0.885 (0.82-0.914)	0.956 (0.718-1.2)	1.08 (0.805-1.21)	0.5220	0.9184
CpG (TLR9)	0.877 (0.781-1.1)	0.94 (0.923-1.07)	0.974 (0.666-1.01)	0.7470	0.9766	0.998 (0.952-1.04)	0.889 (0.838-1.15)	1.02 (0.867-1.19)	0.8870	0.9303
PMA-Iono	0.999 (0.861-1.05)	1.06 (0.995-1.28)	0.961 (0.852-1.1)	0.1260	0.6121	1.04 (0.983-1.17)	1.11 (0.978-1.41)	1.13 (0.941-1.32)	0.8150	0.9303
**Syk**										
Basal	39.5 (35.7-60.6)	45 (36.7-60.9)	44.2 (30.9-53.7)	0.8740	0.9766	49.9 (44.2-59.2)	49.2 (35.8-78.5)	45.1 (34.8-58.9)	0.7160	0.9303
IFNα	0.968 (0.831-1.24)	1.21 (1.09-1.93)	1.45 (0.876-1.87)	0.2020	0.6121	1.13 (1.05-1.3)	1.36 (1.02-1.52)	1.12 (0.938-1.54)	0.8370	0.9303
LPS (TLR4)	0.972 (0.917-1.1)	1.03 (0.892-1.21)	0.995 (0.886-1.08)	0.9490	0.9766	1.07 (1.06-1.13)	1.09 (0.993-1.12)	1.05 (0.975-1.18)	0.8300	0.9303
R848 (TLR7/8)	1.01 (0.895-1.16)	1.02 (0.927-1.06)	0.951 (0.897-1.11)	0.9390	0.9766	1.03 (0.838-1.33)	0.986 (0.876-1.05)	1.01 (0.935-1.19)	0.6750	0.9303
CpG (TLR9)	0.852 (0.781-1.13)	1.01 (0.953-1.13)	0.888 (0.842-0.965)	0.2740	0.7541	0.911 (0.762-1.14)	1.02 (0.915-1.16)	1.1 (0.943-1.31)	0.3970	0.9022
PMA-Iono	1.02 (0.871-1.2)	1.3 (1.28-1.62)	1.47 (1.06-1.54)	**0.0492**	0.6121	1.25 (1.19-1.31)	1.23 (0.988-1.42)	1.35 (1.15-1.55)	0.4790	0.9184

Supplemental Table 17. Fold change in phospho-proteins following plasmacytoid dendritic cell stimulation with various stimuli. Median metal intensity and IQR shown for unstimulated/basal conditions. Median fold change and IQR over the unstimulated/basal conditions shown for all other stimuli.

Plasmacytoid Dendritic Cells										
	Median (Interquartile Range)					Median (Interquartile Range)				
	**White Controls**	**White SLE INACT**	**White SLE ACT**	**KW p-value**	**FDR q-value**	**Black Controls**	**Black SLE INACT**	**Black SLE ACT**	**KW p-value**	**FDR q-value**
**cCASP3**										
Basal	8.98 (8.04-12.7)	12.5 (10.8-13.8)	10.4 (8.9-12)	0.1860	0.8493	10.7 (9.64-11.8)	11.4 (6.97-15.3)	12.2 (9.25-13.9)	0.5710	0.9625
IFNα	1.01 (0.88-1.17)	1.29 (0.937-1.42)	1.1 (0.851-1.28)	0.4070	0.8493	1.17 (1.05-1.31)	1.27 (1.02-1.61)	1.67 (0.985-1.98)	0.4150	0.9625
LPS (TLR4)	0.926 (0.837-1.01)	0.949 (0.556-1.2)	1.1 (0.908-1.32)	0.3340	0.8493	0.907 (0.771-1.1)	0.996 (0.791-1.08)	1.11 (0.9-1.2)	0.5490	0.9625
R848 (TLR7/8)	0.971 (0.923-1.14)	0.988 (0.842-1.03)	1.04 (0.962-1.27)	0.6280	0.8493	1.06 (0.837-1.33)	1.07 (0.78-1.54)	0.94 (0.779-1.33)	0.8770	0.9625
CpG (TLR9)	0.976 (0.659-1.09)	0.932 (0.703-1.08)	0.949 (0.927-1.12)	0.9960	1	1.06 (0.706-1.24)	1.07 (0.884-1.22)	0.882 (0.76-1.07)	0.7110	0.9625
PMA-Iono	0.897 (0.726-1.12)	1.16 (0.985-1.42)	1.27 (0.941-1.34)	0.0932	0.8493	1.23 (1-1.39)	1.2 (0.917-1.62)	1.31 (0.996-1.7)	0.9760	0.9858
**pCREB**										
Basal	14.3 (8.97-28.6)	18 (14.8-27)	12.1 (7.11-25.8)	0.5730	0.8493	10.2 (9.1-13.8)	22 (13.6-42.1)	23.6 (10.1-31.8)	0.2120	0.9565
IFNα	0.984 (0.725-1.27)	1.13 (0.485-1.52)	1.89 (0.942-2.09)	0.3200	0.8493	1.67 (0.995-2.24)	0.914 (0.581-1.78)	1.46 (0.712-3.81)	0.2960	0.9625
LPS (TLR4)	0.839 (0.509-1.06)	0.766 (0.447-1.27)	1.76 (1.04-2.77)	**0.0423**	0.8493	1.09 (0.886-1.27)	1 (0.645-1.47)	0.833 (0.598-1.28)	0.7940	0.9625
R848 (TLR7/8)	1.53 (0.706-1.66)	0.997 (0.646-2.25)	1.4 (0.901-4.92)	0.4270	0.8493	2.72 (1.76-3.99)	2.16 (1.55-2.66)	2.35 (1.6-6.16)	0.6590	0.9625
CpG (TLR9)	0.998 (0.624-1.48)	0.638 (0.277-1.14)	1.03 (0.821-1.51)	0.4810	0.8493	1.19 (0.858-2.6)	0.882 (0.645-2.04)	1.2 (0.853-1.86)	0.8570	0.9625

PMA-Iono	2.91 (1.69-3.44)	2.6 (1.08-3.08)	1.8 (1.7-5.9)	0.6080	0.8493	5.97 (3.09-8.73)	2.43 (1.82-5.21)	3.25 (1.75-6.69)	0.3130	0.9625
**pERK1/2**										
Basal	5.35 (4.54-7.25)	5.76 (4.16-7.58)	7.39 (3.78-13.2)	0.9680	0.9961	4.7 (3.93-5.02)	6 (4.19-10.8)	5.45 (3.23-6.51)	0.4650	0.9625
IFNα	1.16 (1.04-1.29)	1.21 (0.879-1.56)	0.94 (0.888-1.86)	0.8320	0.99	1.18 (0.963-1.61)	1.06 (0.693-1.65)	1.22 (0.743-2.13)	0.7860	0.9625
LPS (TLR4)	1.18 (0.896-1.28)	0.929 (0.846-1.08)	1.32 (0.468-1.5)	0.4510	0.8493	1.05 (0.894-1.22)	1.04 (0.758-1.92)	0.601 (0.5-0.718)	**0.0070**	0.1549
R848 (TLR7/8)	1.33 (1.16-1.64)	1.65 (1.29-2.79)	1.79 (1.02-2.2)	0.6000	0.8493	3.82 (1.19-7.44)	2.95 (1.26-5.75)	3.18 (1.86-4.16)	0.9000	0.9625
CpG (TLR9)	1.07 (0.954-1.16)	1.01 (0.762-1.19)	0.565 (0.361-1.22)	0.6500	0.8493	1.16 (0.961-1.2)	0.62 (0.536-1.11)	1.09 (0.715-1.51)	0.3590	0.9625
PMA-Iono	2.25 (1.85-5.5)	3.26 (2.41-5.12)	2.39 (1.34-3.59)	0.3880	0.8493	4.39 (3.06-4.76)	3.32 (2.36-5.72)	2.15 (2.01-3.25)	0.1910	0.9565
**p-p38**										
Basal	5.75 (4.19-11.6)	7.69 (6.46-9.15)	4.75 (4.41-13)	0.8350	0.99	4.32 (3.37-5.78)	3.55 (2.82-10.2)	4.51 (3.66-5.47)	0.6370	0.9625
IFNα	0.85 (0.679-1.14)	0.773 (0.58-1.45)	0.838 (0.465-1.34)	0.9160	0.9954	1.29 (0.816-1.54)	1.17 (0.55-1.57)	0.705 (0.433-1.05)	0.4900	0.9625
LPS (TLR4)	0.988 (0.531-1.39)	0.832 (0.671-1.33)	1.2 (1.14-1.58)	0.4180	0.8493	1.24 (1.03-1.33)	0.98 (0.683-1.44)	1.08 (0.71-1.28)	0.7090	0.9625
R848 (TLR7/8)	3.69 (1.8-5.6)	4.3 (2.65-6.7)	5.49 (3.97-9)	0.4750	0.8493	15.4 (5.89-22)	13.2 (7.42-17.4)	14.3 (6.75-19.1)	0.8290	0.9625
CpG (TLR9)	1.02 (0.854-1.15)	0.724 (0.377-0.879)	0.784 (0.653-0.81)	0.1460	0.8493	1 (0.909-3.11)	1.14 (0.755-1.98)	1.08 (0.525-2.59)	0.8080	0.9625
PMA-Iono	2.23 (1.43-2.52)	2.16 (1.96-3.16)	1.99 (1.17-2.88)	0.9280	0.9954	3.42 (2.71-4.79)	1.94 (0.963-2.31)	3.02 (1.6-5.31)	0.2220	0.9565
**p-PLCg2**										
Basal	7.22 (4.03-10.2)	8.1 (5.63-9.35)	7.7 (5.33-9.17)	0.9490	0.9954	6.52 (6.03-8.18)	4.19 (3.64-4.75)	5.84 (4.39-7.76)	**0.0179**	0.1627
IFNα	1.29 (1.08-1.48)	0.964 (0.891-1.3)	1.06 (0.746-1.73)	0.6500	0.8493	0.807 (0.753-0.915)	1.32 (1.01-1.89)	0.894 (0.543-1.18)	**0.0137**	0.1549

LPS (TLR4)	1.21 (1.13-1.41)	0.806 (0.54-1.23)	0.875 (0.618-1.13)	0.2520	0.8493	0.752 (0.59-0.929)	1.2 (0.872-2)	0.812 (0.603-1.19)	0.1070	0.7295
R848 (TLR7/8)	1.05 (0.942-1.25)	0.985 (0.742-1.33)	0.872 (0.531-1)	0.4090	0.8493	0.772 (0.636-0.922)	1.14 (0.966-1.32)	0.805 (0.729-1.04)	**0.0142**	0.1549
CpG (TLR9)	1.04 (1.01-1.19)	0.906 (0.668-1.25)	1.02 (0.913-1.05)	0.6540	0.8493	0.978 (0.853-1.13)	1.21 (0.962-1.61)	0.895 (0.689-1.48)	0.3160	0.9625
PMA-Iono	1.18 (0.974-1.35)	0.874 (0.751-1.22)	1.42 (1.06-1.47)	0.3050	0.8493	0.778 (0.726-0.897)	1.5 (1.15-2.04)	0.646 (0.461-0.96)	**0.0039**	0.1549
**pSTAT1**										
Basal	24.3 (14-31.4)	29.4 (20.4-34.6)	30.4 (24.9-39)	0.4700	0.8493	28.5 (23.3-33.8)	31.4 (25.8-36.6)	20.3 (17.1-38.1)	0.6510	0.9625
IFNα	6.49 (4.19-8.58)	7 (3.54-11.1)	3.15 (1.72-7.52)	0.4660	0.8493	6.21 (4.16-7.82)	6.6 (5.35-9.41)	8.49 (2.42-12.3)	0.9640	0.9858
LPS (TLR4)	1.08 (0.801-1.15)	1.25 (0.847-1.46)	0.895 (0.523-0.977)	0.2630	0.8493	0.995 (0.828-1.15)	1.21 (0.913-1.25)	0.862 (0.592-1.16)	0.3200	0.9625
R848 (TLR7/8)	1.01 (0.799-1.13)	0.926 (0.664-1.02)	0.829 (0.451-1.52)	0.6210	0.8493	0.99 (0.843-1.13)	0.912 (0.732-1.13)	1.41 (0.758-1.69)	0.8110	0.9625
CpG (TLR9)	0.965 (0.614-1.27)	1.09 (0.999-1.17)	0.807 (0.511-1.34)	0.7630	0.9458	0.783 (0.728-0.978)	0.83 (0.737-0.951)	0.962 (0.669-1.36)	0.4950	0.9625
PMA-Iono	1.2 (1.04-1.27)	1.21 (0.92-1.82)	0.709 (0.596-1.04)	0.1410	0.8493	1.09 (0.996-1.23)	1.11 (0.811-1.2)	1.05 (0.798-1.37)	0.8390	0.9625
**pSTAT3**										
Basal	6.83 (6.2-10.2)	6.53 (5.7-9.34)	8.05 (7.32-16.4)	0.4160	0.8493	5.99 (4.63-7.49)	5.04 (3.95-7.88)	6.95 (5.15-8.54)	0.7320	0.9625
IFNα	9.76 (8.19-15.8)	11 (7.19-12.8)	4.14 (3.04-10.4)	0.2370	0.8493	14.2 (9.29-20.4)	10.7 (7.59-19.1)	6.42 (3.98-13.7)	0.2280	0.9565
LPS (TLR4)	0.789 (0.681-0.989)	0.907 (0.779-1.38)	0.689 (0.464-0.922)	0.4100	0.8493	0.936 (0.674-1.07)	1.25 (1.17-1.66)	0.824 (0.794-1.24)	**0.0424**	0.3304
R848 (TLR7/8)	0.88 (0.605-1.36)	1.04 (0.882-1.38)	0.81 (0.654-1.43)	0.5360	0.8493	0.998 (0.8-1.13)	0.964 (0.795-1.3)	1.09 (0.922-1.33)	0.8130	0.9625
CpG (TLR9)	0.852 (0.739-0.931)	1.02 (0.666-1.52)	0.798 (0.527-1.57)	0.7450	0.9449	1.13 (0.921-1.17)	0.995 (0.784-1.2)	0.984 (0.837-1.3)	0.9680	0.9858
PMA-Iono	1.07 (1.03-1.16)	1.02 (0.76-1.48)	0.801 (0.383-1.2)	0.4480	0.8493	1.04 (0.73-1.15)	1.02 (0.779-1.05)	1 (0.518-1.28)	0.8930	0.9625

**pSTAT5**										
Basal	11 (9.99-15.1)	11.1 (8.94-16.9)	12.5 (8.77-19.7)	0.8840	0.9954	9.81 (8.41-11.6)	8.83 (7.14-18)	10.5 (8.51-20.7)	0.6440	0.9625
IFNα	2.93 (2.12-3.73)	3.21 (1.84-6.9)	2.24 (1.03-3.46)	0.4720	0.8493	5.99 (4.48-7.06)	5.22 (3.23-7.37)	2.06 (1.09-3.3)	**0.0120**	0.1549
LPS (TLR4)	0.951 (0.74-1.03)	0.982 (0.829-1.31)	0.78 (0.494-1.07)	0.4940	0.8493	0.865 (0.803-0.904)	1.12 (0.699-1.21)	0.907 (0.811-1.01)	0.6720	0.9625
R848 (TLR7/8)	1.11 (0.933-1.13)	0.958 (0.784-1.14)	0.789 (0.499-1.07)	0.4180	0.8493	0.93 (0.822-1.04)	0.803 (0.655-1.11)	0.906 (0.714-1.21)	0.7330	0.9625
CpG (TLR9)	0.955 (0.81-1.05)	1.01 (0.712-1.24)	0.83 (0.645-0.869)	0.5710	0.8493	0.788 (0.708-1.07)	0.965 (0.898-1.26)	1.01 (0.828-1.28)	0.3660	0.9625
PMA-Iono	1.07 (0.871-1.31)	1.04 (0.846-1.53)	1.03 (0.918-1.19)	0.9490	0.9954	1.04 (0.92-1.11)	1.6 (0.944-1.96)	1.15 (0.742-1.41)	0.4210	0.9625
**Syk**										
Basal	37.7 (32.9-65.1)	56.4 (38.7-60)	38.8 (28.5-57.7)	0.5460	0.8493	54.7 (46.3-61.5)	55.3 (32.6-79)	39.4 (33.9-50.3)	0.5780	0.9625
IFNα	1.02 (0.844-1.22)	1.41 (1.19-1.57)	1.05 (0.953-1.54)	0.2140	0.8493	1.16 (0.972-1.58)	1.2 (0.954-1.65)	1.28 (1.12-1.5)	0.8020	0.9625
LPS (TLR4)	0.933 (0.877-1.05)	0.901 (0.877-1.08)	0.941 (0.785-1.24)	0.8770	0.9954	1.03 (0.981-1.09)	1.14 (0.985-1.3)	1.17 (0.777-1.52)	0.8180	0.9625
R848 (TLR7/8)	0.984 (0.844-1.17)	0.964 (0.806-1.08)	0.838 (0.663-0.867)	0.3540	0.8493	1.02 (0.849-1.11)	0.996 (0.81-1.05)	1.25 (0.722-1.46)	0.6730	0.9625
CpG (TLR9)	0.953 (0.905-0.989)	0.866 (0.809-0.94)	0.889 (0.844-1.07)	0.2330	0.8493	0.986 (0.958-1.14)	0.894 (0.744-1.41)	1.1 (0.934-1.19)	0.6890	0.9625
PMA-Iono	1.03 (0.945-1.26)	1.28 (1.22-1.51)	1.08 (0.922-1.31)	0.4640	0.8493	1.24 (1.21-1.3)	1.4 (0.979-1.61)	1.75 (1.38-2.08)	0.1560	0.9454

Supplemental Table 18. **Fold change in phospho-proteins following natural killer (NK) cell stimulation with various stimuli.** Median metal intensity and IQR shown for unstimulated/basal conditions. Median fold change and IQR over the unstimulated/basal conditions shown for all other stimuli.

NK Cells										
	Median (Interquartile Range)					Median (Interquartile Range)				
	**White Controls**	**White SLE INACT**	**White SLE ACT**	**KW p-value**	**FDR q-value**	**Black Controls**	**Black SLE INACT**	**Black SLE ACT**	**KW p-value**	**FDR q-value**
**cCASP3**										
Basal	8.45 (7.45-9.93)	9.36 (7.81-11.4)	13.8 (9.73-14.2)	0.1120	0.953	8.72 (7.59-10)	10.2 (9.03-11.5)	10.6 (9.56-18.1)	0.1630	0.6408
IFNα	1.05 (0.971-1.31)	1.36 (1.06-1.58)	0.93 (0.835-1.47)	0.1930	0.953	1.5 (1.13-1.73)	1.32 (1.17-1.97)	1.28 (1.05-1.84)	0.7630	0.9156
LPS (TLR4)	1.02 (0.911-1.13)	0.972 (0.863-1.6)	0.917 (0.708-0.97)	0.3820	0.953	1.09 (1.07-1.23)	1.04 (0.999-1.12)	0.9 (0.809-1.13)	0.2210	0.6969
R848 (TLR7/8)	1 (0.935-1.13)	0.979 (0.85-1.07)	0.915 (0.808-1.1)	0.8600	0.9969	1.13 (1.04-1.38)	1.07 (0.988-1.18)	0.92 (0.861-1.11)	0.1780	0.6408
CpG (TLR9)	1.04 (0.985-1.09)	1.05 (0.92-1.3)	0.823 (0.707-1.11)	0.5080	0.953	1.26 (1.1-1.5)	1.04 (0.745-1.21)	1.05 (0.924-1.3)	0.1790	0.6408
PMA-Iono	1.07 (0.997-1.17)	1.34 (1.24-1.57)	1.03 (0.98-1.45)	0.0540	0.953	1.28 (1.19-1.51)	1.28 (1.16-1.41)	1.39 (1.16-1.54)	0.8960	0.922
**pCREB**										
Basal	43.2 (35.1-57)	40 (29.9-60.7)	44.3 (26.2-50.1)	0.9630	0.9969	43.2 (35.6-48.1)	50.5 (32.9-61.3)	40.6 (34.4-49.4)	0.8080	0.9174
IFNα	0.988 (0.934-1.05)	1.2 (1.04-1.61)	1.25 (1.02-1.71)	0.1180	0.953	1.05 (0.935-1.14)	1.16 (0.98-1.41)	1.13 (0.964-1.47)	0.4950	0.7893
LPS (TLR4)	1.07 (0.952-1.28)	1.31 (1.2-1.55)	1.21 (1.03-1.6)	0.3780	0.953	1.54 (1.12-1.62)	1.19 (1.15-1.67)	1.27 (1.02-1.79)	0.9680	0.9777
R848 (TLR7/8)	1.08 (1.01-1.18)	1.24 (1.03-1.33)	1.15 (1.13-1.26)	0.3470	0.953	1.29 (1.17-1.59)	1.23 (1.06-1.34)	1.48 (1.22-1.78)	0.6330	0.8597
CpG (TLR9)	1.05 (0.931-1.13)	1.11 (0.795-1.18)	0.999 (0.881-1.17)	0.9870	0.9969	1.15 (1.01-1.29)	0.951 (0.865-1)	1.03 (0.92-1.22)	0.1280	0.6408

PMA-Iono	1.2 (1.18-1.39)	1.39 (1.17-1.75)	1.37 (1.26-1.87)	0.3370	0.953	1.37 (1.24-1.53)	1.68 (1.42-1.83)	1.54 (1.19-1.85)	0.4840	0.7893
**pERK1/2**										
Basal	8.91 (4.94-34)	9.6 (7.35-16.4)	5.19 (4.57-15.8)	0.3590	0.953	5.69 (4.17-6.96)	6.69 (4.89-13.5)	7.4 (4.23-15.5)	0.6800	0.8625
IFNα	0.874 (0.756-1.29)	1.56 (0.908-1.71)	1.22 (1.04-1.46)	0.1430	0.953	1.05 (0.65-1.42)	1.43 (1.18-1.78)	1.14 (0.834-1.33)	0.2970	0.733
LPS (TLR4)	1.81 (1.16-2.16)	2.02 (1.55-3.25)	2.93 (1.74-4.96)	0.5080	0.953	5.89 (1.6-8.99)	2.2 (1.53-5.04)	1.78 (1-4.77)	0.3790	0.7656
R848 (TLR7/8)	1.24 (1.11-1.92)	1.46 (1.09-2.38)	2.55 (1.41-2.77)	0.0997	0.953	1.03 (0.857-5.9)	2.73 (1.64-4.31)	3.21 (1.6-6.98)	0.5110	0.7893
CpG (TLR9)	1.12 (0.951-2.33)	0.844 (0.62-1.23)	1.26 (1.09-1.47)	0.1700	0.953	1.01 (0.836-1.29)	1.11 (0.818-1.52)	1.12 (0.569-1.29)	0.8740	0.922
PMA-Iono	4.26 (3.58-5.86)	2.88 (2.31-5.55)	4.18 (2.87-5.75)	0.6010	0.953	5.71 (2.32-6.51)	4.63 (2.68-5.41)	2.91 (2.21-4.78)	0.6030	0.8433
**p-p38**										
Basal	13.5 (9.71-33.9)	12.8 (9.27-16.4)	8.82 (7.49-15.8)	0.4200	0.953	7.41 (6-8.96)	8.88 (6.27-12.9)	13.3 (8.14-17)	0.3150	0.733
IFNα	0.945 (0.813-1.04)	1.13 (0.94-1.29)	1 (0.97-1.18)	0.3030	0.953	0.976 (0.55-1.32)	1.14 (0.992-1.63)	0.915 (0.814-1.24)	0.4500	0.7893
LPS (TLR4)	3.74 (2.44-4.39)	5.44 (4.21-7.04)	5.09 (4.62-5.47)	**0.0197**	0.6954	8.69 (4.33-12)	6.02 (4.97-7.95)	4.16 (1.91-7.64)	0.2870	0.733
R848 (TLR7/8)	3.41 (2.56-4.95)	4.56 (3.65-6.22)	4.82 (4.12-7.71)	0.2520	0.953	4.41 (2.3-9.17)	6.17 (4.74-10.1)	6.27 (2.96-6.91)	0.7600	0.9156
CpG (TLR9)	0.985 (0.946-1.14)	1.03 (0.856-1.25)	1.07 (1-1.31)	0.5550	0.953	1.18 (1.1-1.39)	1.03 (0.904-1.16)	0.851 (0.72-1.33)	0.1720	0.6408
PMA-Iono	1.86 (1.72-2.27)	2.19 (1.72-2.65)	2.1 (1.49-2.55)	0.7330	0.9635	2.39 (1.25-3.14)	2.38 (1.94-3.12)	1.34 (1.04-2.13)	0.1480	0.6408
**p-PLCg2**										
Basal	29 (14-34.1)	22.4 (17.2-27.9)	19.8 (17.6-25.6)	0.7370	0.9635	14.6 (12.8-25.6)	15 (7.91-26.4)	23.5 (13.8-35.4)	0.3790	0.7656
IFNα	0.881 (0.802-0.956)	1.01 (0.802-1.31)	0.669 (0.633-1.5)	0.6590	0.953	0.809 (0.469-0.983)	0.935 (0.818-1.34)	0.713 (0.523-0.848)	**0.0228**	0.4145

LPS (TLR4)	1 (0.925-1.07)	0.918 (0.774-1.17)	1.01 (0.76-1.38)	0.8360	0.9969	0.82 (0.677-1.08)	0.928 (0.768-1.1)	0.836 (0.711-1.08)	0.6500	0.8597
R848 (TLR7/8)	0.964 (0.863-1.15)	0.932 (0.84-0.989)	1.06 (0.791-1.34)	0.6070	0.953	0.992 (0.611-1.19)	1.03 (0.734-1.33)	0.936 (0.782-1.26)	0.8940	0.922
CpG (TLR9)	0.836 (0.783-1.13)	0.976 (0.786-1.13)	0.864 (0.81-1.4)	0.7420	0.9635	1 (0.891-1.34)	0.988 (0.91-1.22)	0.822 (0.686-1.05)	0.2770	0.733
PMA-Iono	0.858 (0.791-0.922)	0.888 (0.661-0.984)	0.849 (0.682-1.08)	0.9310	0.9969	0.527 (0.423-0.638)	0.93 (0.772-1.3)	0.613 (0.349-0.69)	**0.0048**	0.1309
**pSTAT1**										
Basal	15.8 (9.61-18.6)	22.5 (10.2-27.2)	17.3 (14.3-36.9)	0.7380	0.9635	21.8 (12.6-28.2)	18.7 (15.3-32)	24.9 (18.1-28.8)	0.8300	0.9174
IFNα	6.22 (3.73-10.9)	8.22 (2.81-9.78)	6.19 (1.6-7.83)	0.5270	0.953	3.5 (3.07-5.28)	4.24 (3.64-6.12)	4.38 (1.41-7.28)	0.7890	0.9156
LPS (TLR4)	0.962 (0.822-1.12)	1.01 (0.901-1.32)	0.957 (0.914-1.39)	0.7780	0.9868	0.879 (0.784-0.983)	1.03 (0.955-1.3)	0.791 (0.632-1.02)	0.1500	0.6408
R848 (TLR7/8)	0.936 (0.695-1.26)	1.12 (0.845-1.42)	0.955 (0.708-1.2)	0.3990	0.953	0.863 (0.674-0.992)	1.09 (0.789-1.2)	1.02 (0.899-1.18)	0.5700	0.8181
CpG (TLR9)	1.03 (0.714-1.3)	1.11 (0.955-1.32)	0.946 (0.636-1.19)	0.6450	0.953	1.17 (0.933-1.43)	1.1 (0.855-1.33)	0.97 (0.87-1.01)	0.1490	0.6408
PMA-Iono	1.09 (0.963-1.45)	1.26 (0.962-1.53)	1.14 (0.948-1.61)	0.8670	0.9969	0.769 (0.507-0.883)	1.23 (0.892-1.45)	1 (0.73-1.18)	0.1660	0.6408
**pSTAT3**										
Basal	6.93 (6.55-9.09)	10.3 (8.12-14.7)	9.16 (7.7-12.7)	0.2530	0.953	5.32 (4.35-7.6)	5.45 (4.27-6.14)	7.32 (4.98-8)	0.4820	0.7893
IFNα	9.02 (6.03-9.69)	3.69 (2.07-7.51)	2.75 (1.03-12.1)	0.2350	0.953	8.58 (6.19-14)	4.55 (3.73-11.6)	4.07 (1.89-8.04)	0.2590	0.733
LPS (TLR4)	1.05 (0.949-1.33)	0.949 (0.802-1.32)	0.932 (0.824-1.05)	0.4030	0.953	1.08 (0.937-1.15)	1.17 (0.916-1.33)	1.11 (0.981-1.39)	0.8410	0.9174
R848 (TLR7/8)	1.03 (0.821-1.23)	0.876 (0.727-1.04)	0.822 (0.64-1.11)	0.5400	0.953	0.996 (0.911-1.07)	1.14 (1.02-1.28)	1 (0.916-1.19)	0.3310	0.733
CpG (TLR9)	1.04 (0.918-1.16)	0.955 (0.842-1.04)	0.933 (0.623-1.11)	0.6400	0.953	1.27 (0.96-1.48)	1.03 (0.852-1.25)	1 (0.924-1.16)	0.6620	0.8597
PMA-Iono	1.04 (0.892-1.09)	0.824 (0.676-0.876)	0.828 (0.607-0.952)	**0.0255**	0.6954	0.769 (0.714-0.869)	1.11 (0.896-1.5)	0.97 (0.839-1.09)	0.0862	0.6408

**pSTAT5**										
Basal	14.1 (10.9-18.4)	15 (12.2-18.1)	14.3 (11.1-15.3)	0.9800	0.9969	9.06 (8.5-13)	12.1 (6.74-13.8)	19.5 (12.9-27.4)	**0.0415**	0.4527
IFNα	2.54 (1.94-3.08)	2.05 (1.62-4.22)	1.61 (0.953-3.46)	0.6640	0.953	4.35 (3.09-5.99)	3 (1.94-4.5)	1.03 (0.828-1.39)	**0.0012**	0.0654
LPS (TLR4)	0.981 (0.939-1.05)	0.914 (0.814-0.984)	1.09 (0.797-1.12)	0.5350	0.953	0.941 (0.869-1.04)	0.92 (0.88-1.21)	0.949 (0.681-1.15)	0.7730	0.9156
R848 (TLR7/8)	0.944 (0.86-1.01)	0.917 (0.817-1.06)	0.998 (0.894-1.24)	0.6380	0.953	0.967 (0.776-1.14)	0.99 (0.846-1.2)	0.892 (0.682-1.01)	0.3360	0.733
CpG (TLR9)	0.935 (0.876-1.01)	1.03 (0.874-1.14)	0.879 (0.753-1.29)	0.5550	0.953	1.11 (0.997-1.36)	1.05 (0.934-1.35)	0.932 (0.84-1.09)	0.2300	0.6969
PMA-Iono	0.956 (0.912-1.1)	0.943 (0.84-1.27)	1.03 (0.833-1.14)	0.9640	0.9969	0.86 (0.774-0.957)	1.09 (0.968-1.53)	0.963 (0.828-1.06)	**0.0376**	0.4527
**Syk**										
Basal	31.9 (30.1-38)	33.8 (27.7-44.7)	35.1 (31-40.1)	0.8900	0.9969	42.7 (41.2-44.3)	37.3 (29-45.9)	36.3 (33.9-45.4)	0.4660	0.7893
IFNα	1.02 (0.94-1.06)	1.12 (1.03-1.42)	1.2 (1.04-1.21)	0.1980	0.953	1 (0.964-1.06)	1.09 (0.92-1.42)	1.21 (0.964-1.46)	0.5580	0.8181
LPS (TLR4)	0.994 (0.952-1.1)	1.07 (0.914-1.32)	1 (0.902-1.06)	0.8980	0.9969	1.02 (0.906-1.05)	1.06 (0.968-1.25)	0.961 (0.916-1.15)	0.4140	0.7893
R848 (TLR7/8)	1 (0.952-1.08)	1.09 (0.916-1.19)	0.951 (0.912-0.993)	0.4020	0.953	0.947 (0.896-1.01)	1.05 (0.879-1.16)	0.992 (0.923-1.15)	0.5210	0.7893
CpG (TLR9)	0.998 (0.945-1.03)	0.965 (0.859-1.18)	1 (0.878-1.09)	0.8960	0.9969	1.01 (0.953-1.18)	0.912 (0.878-1.15)	0.999 (0.951-1.07)	0.5040	0.7893
PMA-Iono	0.991 (0.954-1.06)	1.13 (0.944-1.24)	1.18 (0.963-1.34)	0.4680	0.953	0.96 (0.891-1.07)	1.15 (0.994-1.35)	1.08 (0.948-1.3)	0.1880	0.6408

Supplemental Table 19. Fold change in phospho-proteins following monocyte stimulation with various stimuli. Median metal intensity and IQR shown for unstimulated/basal conditions. Median fold change and IQR over the unstimulated/basal conditions shown for all other stimuli.

Monocytes										
	Median (Interquartile Range)					Median (Interquartile Range)				
	**White Controls**	**White SLE INACT**	**White SLE ACT**	**KW p-value**	**FDR q-value**	**Black Controls**	**Black SLE INACT**	**Black SLE ACT**	**KW p-value**	**FDR q-value**
**cCASP3**										
Basal	9.37 (7.61-11.2)	9.99 (9.19-11.7)	10.4 (8.7-10.6)	0.7630	0.9854	9.22 (8.61-10.4)	9.24 (8.49-10.5)	9.64 (8.64-12.7)	0.7470	0.9318
IFNα	1.07 (1.06-1.12)	1.19 (1.02-1.26)	1.19 (1.06-1.25)	0.3180	0.8831	1.14 (1.04-1.17)	1.23 (1.13-1.63)	1.13 (1.04-1.25)	0.1700	0.8429
LPS (TLR4)	1.06 (1.02-1.12)	1.01 (0.985-1.08)	0.97 (0.951-1.02)	0.0918	0.8817	1.11 (1.07-1.15)	1.12 (1.05-1.15)	1.04 (1.02-1.11)	0.1580	0.8429
R848 (TLR7/8)	1.02 (0.968-1.05)	0.977 (0.932-1.07)	0.995 (0.93-1.02)	0.6400	0.9776	1.11 (1.09-1.12)	1.01 (0.96-1.04)	1.02 (0.989-1.04)	**0.0284**	0.5163
CpG (TLR9)	1.02 (0.936-1.09)	0.982 (0.941-1.02)	0.973 (0.956-0.996)	0.4700	0.9234	1.08 (1.04-1.11)	0.979 (0.965-1.06)	1.01 (0.945-1.03)	0.0694	0.6062
PMA-Iono	1.13 (1.04-1.22)	1.16 (1.08-1.33)	1.11 (1.07-1.22)	0.6680	0.9776	1.3 (1.18-1.46)	1.38 (1.25-1.61)	1.25 (1.22-1.43)	0.5460	0.9006
**pCREB**										
Basal	56.2 (47.6-80.7)	59.1 (45.9-71.2)	64.3 (45.9-75.6)	0.8320	0.9865	58.8 (52-73.3)	67.6 (44.4-100)	54.8 (47.5-66.9)	0.5620	0.9006
IFNα	1.09 (1.05-1.1)	1.26 (1.11-1.35)	1.26 (0.869-1.38)	0.2470	0.8831	1.19 (1.15-1.33)	1.06 (0.984-1.44)	1.29 (1.21-1.33)	0.2320	0.9006
LPS (TLR4)	1.03 (1.01-1.09)	1.15 (1.08-1.22)	1.07 (1.06-1.26)	0.0839	0.8817	1.17 (1.09-1.4)	1.2 (1.05-1.26)	1.26 (1.1-1.35)	0.6510	0.9118
R848 (TLR7/8)	1.05 (0.969-1.15)	1.09 (0.96-1.17)	1.04 (0.917-1.14)	0.9390	1	1.31 (1.13-1.46)	1.13 (0.997-1.19)	1.25 (1.17-1.35)	0.0923	0.6293
CpG (TLR9)	1.02 (0.963-1.1)	0.972 (0.938-1.02)	1.02 (0.914-1.07)	0.6010	0.9776	1.07 (1.03-1.18)	0.972 (0.832-1.12)	1.01 (0.985-1.09)	0.2240	0.9006

PMA-Iono	1.2 (1.13-1.26)	1.4 (1.28-1.47)	1.35 (1.29-1.47)	**0.0413**	0.8817	1.49 (1.3-1.69)	1.47 (1.28-1.63)	1.59 (1.4-1.9)	0.4720	0.9006
**pERK1/2**										
Basal	9.11 (6.1-99.6)	18 (12.9-31.4)	8.66 (7.46-32.3)	0.3860	0.8831	8.33 (7.02-11.6)	9.42 (6.53-11.6)	7.09 (4.61-14.6)	0.6520	0.9118
IFNα	1.2 (1.09-1.35)	1.38 (1.29-2)	1.27 (1.03-1.65)	0.3160	0.8831	1.84 (1.46-2.32)	1.54 (1.3-1.98)	1.21 (1.09-1.52)	0.3120	0.9006
LPS (TLR4)	1.72 (1.35-1.9)	1.75 (1.43-2.22)	1.61 (1.15-3.87)	0.8130	0.9854	4.16 (2.54-7.22)	1.65 (1.42-4.27)	2.05 (1.45-4.85)	0.4780	0.9006
R848 (TLR7/8)	1.26 (1.12-1.45)	1.16 (1.07-1.47)	1.18 (0.924-1.69)	0.9180	1	3.69 (1.28-6.5)	1.73 (1.27-3.82)	2.41 (1.75-4.72)	0.5440	0.9006
CpG (TLR9)	1.08 (0.965-1.18)	0.956 (0.856-1.23)	1.01 (0.787-1.11)	0.6910	0.9776	1.39 (1.28-1.55)	1.09 (0.939-1.2)	1.05 (0.935-1.22)	0.0778	0.6062
PMA-Iono	4.88 (2.54-6.03)	2.72 (2.09-4.81)	4.18 (2.69-5.33)	0.4780	0.9234	5.7 (4.81-11.3)	4.94 (4.34-6.84)	6.38 (4.06-8.66)	0.7890	0.9318
**p-p38**										
Basal	20.6 (15.6-68.8)	24 (16-36.1)	21.6 (19.1-25.3)	0.9940	1	16.8 (12.4-22.2)	16.3 (8.92-29.6)	15.6 (12.6-22.6)	0.9900	0.9999
IFNα	1.18 (1.16-1.22)	1.19 (1.09-1.57)	1.07 (0.922-1.27)	0.5220	0.9413	1.59 (1.25-1.83)	1.45 (1.13-1.62)	1.36 (1.17-1.47)	0.6030	0.9006
LPS (TLR4)	3.83 (3.52-4.46)	4.49 (3.32-5.38)	3.91 (3.18-4.17)	0.5350	0.9413	6.26 (3.92-7.88)	5.06 (3.22-6.6)	5.82 (3.28-7.13)	0.7520	0.9318
R848 (TLR7/8)	4.03 (3.37-4.62)	4.66 (3.21-5.64)	3.97 (3.4-4.78)	0.7170	0.9776	6.25 (3.9-8.73)	5.28 (3.2-6.79)	6.31 (3.76-7.23)	0.7060	0.9318
CpG (TLR9)	1.17 (1.08-1.37)	1.1 (0.905-1.32)	1.06 (1.05-1.14)	0.2680	0.8831	1.41 (1.19-1.75)	1.14 (0.836-1.45)	1.25 (1.15-1.43)	0.3240	0.9006
PMA-Iono	2.48 (1.8-3.24)	2.32 (2.07-3.06)	2.77 (2.27-2.86)	0.9870	1	2.88 (2.35-4.03)	3.19 (2.05-4.21)	3.51 (2.36-4.2)	0.9720	0.9999
**p-PLCg2**										
Basal	7.57 (4.5-9)	6.37 (5.78-8.09)	6.27 (5.19-6.51)	0.8940	1	4.62 (4.01-5.83)	4.35 (3.73-5.04)	3.59 (3.05-4.71)	0.3820	0.9006
IFNα	1.05 (1.01-1.07)	1.18 (1.07-1.62)	1.04 (0.928-1.11)	0.1280	0.8831	1.08 (1-1.27)	1.23 (1.11-1.44)	1.07 (1.04-1.13)	0.3560	0.9006

LPS (TLR4)	1.08 (0.97-1.12)	0.991 (0.928-1.09)	0.947 (0.933-0.997)	0.2680	0.8831	0.968 (0.94-1.02)	1.03 (1.01-1.09)	0.989 (0.92-1.01)	0.1540	0.8429
R848 (TLR7/8)	1.03 (1-1.11)	0.99 (0.931-1.02)	0.982 (0.938-1)	0.2000	0.8831	1.06 (1.02-1.09)	0.98 (0.917-1.03)	0.989 (0.961-1.01)	0.1990	0.9006
CpG (TLR9)	1 (0.961-1.07)	1.03 (0.926-1.12)	0.948 (0.909-0.956)	0.1940	0.8831	1.03 (1.01-1.08)	1.02 (0.942-1.06)	0.983 (0.92-1.03)	0.3020	0.9006
PMA-Iono	1.04 (0.996-1.07)	1.01 (0.972-1.15)	1.02 (0.966-1.09)	0.9180	1	1.03 (0.952-1.11)	1.07 (0.971-1.22)	1.11 (1.06-1.17)	0.5970	0.9006
**pSTAT1**										
Basal	16.8 (11.5-28.2)	25.6 (16.8-33.4)	21 (17.2-26.1)	0.7030	0.9776	21.1 (17.1-30.3)	25.4 (19.2-30.3)	21.9 (15.1-26.7)	0.8030	0.9318
IFNα	6.63 (4.23-9.95)	6.35 (3.64-10.8)	2.94 (2.01-8.74)	0.4130	0.8831	6.88 (4.09-8.34)	5.6 (3.8-8.28)	5.75 (2.22-9.87)	0.9250	0.9892
LPS (TLR4)	0.981 (0.8-1.03)	1.14 (1.05-1.23)	0.961 (0.837-1.11)	0.0970	0.8817	1.03 (0.978-1.13)	1.07 (1.02-1.15)	1.08 (0.997-1.13)	0.8480	0.955
R848 (TLR7/8)	0.952 (0.752-1.02)	1.03 (0.913-1.1)	0.995 (0.712-1.02)	0.4910	0.9234	0.95 (0.88-1.02)	1.01 (0.914-1.08)	1.02 (0.945-1.12)	0.7640	0.9318
CpG (TLR9)	0.952 (0.829-1.07)	0.996 (0.897-1.11)	0.834 (0.768-1.03)	0.3830	0.8831	0.986 (0.966-1.02)	1.02 (0.939-1.09)	1.03 (0.917-1.1)	0.5110	0.9006
PMA-Iono	1.23 (1.14-1.5)	1.33 (1.19-1.87)	1.19 (1.08-1.42)	0.3610	0.8831	1.44 (1.23-1.46)	1.35 (1.16-1.71)	1.28 (1.26-1.79)	0.7720	0.9318
**pSTAT3**										
Basal	10.5 (8.03-11.1)	14 (11.2-18.6)	14.3 (9.58-26.7)	0.4040	0.8831	8.87 (6.79-10.7)	8.35 (6.39-9.67)	9.34 (8.32-13.1)	0.5100	0.9006
IFNα	8.94 (6.54-10.9)	4.61 (3.35-7.98)	2.26 (1.19-10.8)	0.2080	0.8831	12.4 (8.47-15.5)	4.98 (3.82-13.6)	2.29 (1.83-6.44)	**0.0410**	0.559
LPS (TLR4)	1.04 (0.979-1.08)	1.06 (0.913-1.14)	0.95 (0.942-0.977)	0.2170	0.8831	1.03 (0.999-1.05)	1.07 (1.03-1.14)	0.966 (0.937-1.01)	**0.0248**	0.5163
R848 (TLR7/8)	1.01 (0.974-1.08)	0.945 (0.885-1.08)	0.962 (0.871-1.02)	0.4210	0.8831	1.03 (0.99-1.1)	1.01 (0.967-1.08)	0.987 (0.892-1.07)	0.3490	0.9006
CpG (TLR9)	1.05 (0.985-1.06)	1.02 (0.953-1.05)	0.97 (0.92-1.01)	0.4160	0.8831	1.08 (1.06-1.11)	1.04 (0.962-1.1)	0.991 (0.925-1.03)	0.0569	0.6062
PMA-Iono	1.04 (0.975-1.08)	1.05 (1.01-1.11)	1.04 (0.994-1.08)	0.7870	0.9854	1.09 (0.989-1.12)	1.18 (1.06-1.26)	1.11 (0.983-1.19)	0.5080	0.9006

**pSTAT5**										
Basal	9.87 (8.47-11.6)	10.4 (9.38-13.2)	11.8 (9.69-12.2)	0.7050	0.9776	8.02 (7.51-8.86)	9.35 (7.14-13.5)	10.2 (7.77-16.7)	0.5130	0.9006
IFNα	3.3 (2.91-3.75)	3.56 (2.16-5)	1.56 (1.31-4.49)	0.3740	0.8831	8.06 (5.92-9.23)	4.36 (2.15-6.48)	1.55 (1.31-1.69)	**0.0005**	**0.0273**
LPS (TLR4)	1.05 (1.03-1.06)	1.04 (0.996-1.12)	1.06 (0.956-1.16)	0.8930	1	1.04 (1.01-1.08)	1.07 (1.03-1.08)	1.03 (0.981-1.1)	0.8580	0.955
R848 (TLR7/8)	0.994 (0.945-1.01)	1.01 (0.946-1.07)	1.03 (0.916-1.08)	0.7800	0.9854	1.02 (0.984-1.08)	1.01 (0.977-1.03)	1.05 (0.951-1.17)	0.9720	0.9999
CpG (TLR9)	1 (0.976-1.09)	1 (0.97-1.04)	0.952 (0.926-0.975)	0.2710	0.8831	1.06 (1.01-1.1)	1.02 (0.929-1.07)	1 (0.955-1.12)	0.6110	0.9006
PMA-Iono	1.06 (1.02-1.08)	1.17 (1.13-1.34)	1.16 (1.08-1.32)	**0.0124**	0.6763	1.33 (1.28-1.4)	1.35 (1.29-1.45)	1.43 (1.25-1.51)	0.5220	0.9006
**Syk**										
Basal	39.8 (35-65.9)	43.8 (39.3-57.5)	48.1 (36-55.5)	0.9770	1	54 (43.6-60.5)	58.1 (40.7-80.5)	44.2 (40.8-57.5)	0.5530	0.9006
IFNα	1.02 (0.908-1.08)	1.19 (1.07-1.54)	1.21 (0.942-1.41)	0.0951	0.8817	1.13 (1.04-1.31)	1.05 (0.955-1.35)	1.17 (1.05-1.25)	0.9120	0.9892
LPS (TLR4)	1.01 (0.927-1.05)	1.02 (0.983-1.09)	1.02 (0.893-1.1)	0.7040	0.9776	1.04 (0.994-1.08)	1.01 (0.915-1.04)	1.01 (0.953-1.06)	0.6780	0.9245
R848 (TLR7/8)	1.02 (0.921-1.06)	0.948 (0.919-1.04)	0.999 (0.913-1.04)	0.8100	0.9854	1.03 (0.972-1.24)	0.997 (0.933-1.02)	1.03 (0.985-1.09)	0.2950	0.9006
CpG (TLR9)	1.01 (0.981-1.05)	0.987 (0.947-1.03)	1.02 (0.881-1.04)	0.6750	0.9776	1.02 (0.945-1.08)	0.946 (0.918-1.02)	1.02 (0.971-1.06)	0.4640	0.9006
PMA-Iono	1.09 (1.06-1.16)	1.24 (1.07-1.38)	1.3 (1.06-1.42)	0.3180	0.8831	1.15 (1.08-1.19)	1.16 (0.961-1.29)	1.22 (1.05-1.47)	0.5860	0.9006

Supplemental Table 20. **Fold change in phospho-proteins following granulocyte stimulation with various stimuli.** Median metal intensity and IQR shown for unstimulated/basal conditions. Median fold change and IQR over the unstimulated/basal conditions shown for all other stimuli.

Granulocytes										
	Median (Interquartile Range)					Median (Interquartile Range)				
	**White Controls**	**White SLE INACT**	**White SLE ACT**	**KW p-value**	**FDR q-value**	**Black Controls**	**Black SLE INACT**	**Black SLE ACT**	**KW p-value**	**FDR q-value**
**cCASP3**										
Basal	8.82 (7.9-9.61)	8.35 (7.97-10.7)	9.21 (8.6-9.7)	0.7900	0.8833	8.18 (7.84-9.88)	9.05 (8.79-10.3)	9.07 (8.75-11.2)	0.5360	0.8226
IFNα	1 (0.962-1.06)	1.1 (1.04-1.16)	1.03 (1.01-1.06)	0.1890	0.8833	1.03 (0.994-1.09)	1.15 (1.04-1.35)	1.07 (1.02-1.31)	0.3170	0.7171
LPS (TLR4)	1.03 (0.994-1.04)	1.02 (0.992-1.07)	0.985 (0.967-1.03)	0.5350	0.8833	1.06 (1.03-1.07)	1.04 (0.999-1.09)	1.02 (1.01-1.03)	0.1990	0.6783
R848 (TLR7/8)	0.997 (0.969-1.02)	0.997 (0.947-1.03)	0.999 (0.971-1.03)	0.9940	1	1.04 (1.03-1.07)	0.996 (0.968-1.02)	1 (0.983-1.03)	**0.0219**	0.5945
CpG (TLR9)	1.02 (0.977-1.05)	0.984 (0.94-1.02)	0.992 (0.978-0.999)	0.3800	0.8833	1.02 (1.01-1.04)	0.988 (0.961-1.02)	0.986 (0.953-1.01)	0.1680	0.6763
PMA-Iono	1.08 (1.03-1.2)	1.2 (1.14-1.28)	1.14 (1.11-1.3)	0.3330	0.8833	1.39 (1.29-1.45)	1.28 (1.19-1.44)	1.41 (1.25-1.49)	0.7270	0.8924
**pCREB**										
Basal	24.5 (22.5-36.9)	26.9 (22.2-33.2)	25.3 (21.5-28.4)	0.8130	0.8833	25.2 (21.3-32)	23 (18.5-37.8)	17.9 (13.8-28.6)	0.2770	0.7171
IFNα	0.999 (0.887-1.07)	0.997 (0.871-1.24)	1.08 (0.961-1.23)	0.4830	0.8833	1.06 (0.975-1.11)	1.07 (0.927-1.24)	1.24 (1.12-1.38)	0.1110	0.6763
LPS (TLR4)	1.54 (1.17-1.69)	1.29 (1.17-1.78)	1.47 (1.36-1.49)	0.8800	0.923	1.34 (1.25-1.42)	1.44 (1.3-1.5)	1.43 (1.15-1.94)	0.7000	0.8924
R848 (TLR7/8)	1.31 (1.05-1.59)	1.18 (0.983-1.4)	1.29 (1.26-1.37)	0.5450	0.8833	1.41 (1.31-1.59)	1.3 (1.19-1.41)	1.36 (1.12-1.85)	0.7230	0.8924

CpG (TLR9)	0.981 (0.931-1.04)	0.923 (0.894-1.02)	0.973 (0.919-1.07)	0.4840	0.8833	1.13 (0.948-1.19)	0.981 (0.862-1.1)	1 (0.944-1.08)	0.2640	0.7171
PMA-Iono	1.44 (1.14-1.49)	1.26 (1.06-1.51)	1.43 (1.39-1.49)	0.7120	0.8833	1.21 (0.975-1.54)	1.27 (1.07-1.44)	1.42 (1.08-2.02)	0.4010	0.7711
**pERK1/2**										
Basal	5.45 (4.68-6.86)	6.63 (6.12-8.59)	5.47 (4.84-8.32)	0.4900	0.8833	5.35 (4.24-6.23)	4.77 (3.68-6.99)	4.5 (3.7-6.88)	0.9300	0.9642
IFNα	1.04 (0.995-1.11)	1.16 (1.02-1.28)	1.01 (0.964-1.09)	0.3750	0.8833	1.09 (0.981-1.31)	1.12 (1.04-1.3)	1.12 (1.04-1.29)	0.8790	0.9642
LPS (TLR4)	1.1 (1.03-1.19)	1.21 (1.14-1.31)	1.12 (0.984-1.52)	0.4340	0.8833	1.25 (1.15-1.59)	1.22 (1.11-1.33)	1.08 (1.04-1.16)	0.1540	0.6763
R848 (TLR7/8)	1.04 (0.994-1.09)	1 (0.944-1.05)	1 (0.959-1.07)	0.8100	0.8833	1.16 (1.12-1.27)	1.16 (1.09-1.21)	1.08 (1.04-1.14)	0.1860	0.6763
CpG (TLR9)	1.02 (0.967-1.04)	0.949 (0.876-0.977)	0.975 (0.958-1.03)	0.1590	0.8833	1.06 (1.03-1.12)	0.999 (0.918-1.05)	0.988 (0.947-1)	**0.0254**	0.5945
PMA-Iono	3.46 (2.83-4.97)	3.37 (2.45-4.6)	2.69 (2.1-3.95)	0.6820	0.8833	4.84 (3.92-6.26)	3.46 (3.16-4.31)	3.36 (3.07-4.3)	0.1490	0.6763
**p-p38**										
Basal	30 (23.1-38.1)	27.3 (24.3-28.8)	28.3 (26.6-35.3)	0.6000	0.8833	25.7 (22-28.1)	24.7 (19.5-29.4)	22.5 (17.7-23.8)	0.3440	0.7171
IFNα	1.02 (0.943-1.11)	1.05 (0.941-1.08)	0.94 (0.899-0.997)	0.5540	0.8833	1.04 (1.01-1.06)	1.1 (1.06-1.13)	1.08 (1.03-1.15)	0.2950	0.7171
LPS (TLR4)	2.7 (2.42-2.89)	3.39 (2.73-4.08)	2.48 (1.57-2.78)	0.1060	0.8833	3.07 (2.69-3.38)	3.05 (2.31-3.69)	3.25 (2.9-3.78)	0.7130	0.8924
R848 (TLR7/8)	2.42 (2.14-2.76)	1.96 (1.81-3.31)	2.17 (1.63-2.27)	0.5590	0.8833	2.86 (2.65-3.15)	2.7 (1.92-3.34)	3.04 (2.5-3.65)	0.4100	0.7711
CpG (TLR9)	0.975 (0.913-1.06)	0.981 (0.909-1.04)	0.964 (0.922-0.984)	0.7760	0.8833	1.02 (0.998-1.12)	0.985 (0.815-1.05)	0.998 (0.972-1.04)	0.2450	0.7171
PMA-Iono	1.73 (1.46-1.94)	1.85 (1.67-2.11)	1.73 (1.54-1.91)	0.6840	0.8833	2 (1.61-2.17)	1.85 (1.27-2.15)	2.05 (1.92-2.16)	0.5430	0.8226
**p-PLCg2**										

Basal	8.07 (6.07-9.78)	6.48 (5.92-7.08)	6.31 (5.98-7.96)	0.5450	0.8833	6.29 (5.56-7.28)	6.4 (5.55-9.48)	5.41 (5.23-6.74)	0.6020	0.8874
IFNα	0.99 (0.962-1.01)	0.957 (0.898-1.02)	0.981 (0.964-1.07)	0.6840	0.8833	0.992 (0.931-1.02)	1.01 (0.946-1.05)	1.01 (0.877-1.04)	0.8960	0.9642
LPS (TLR4)	0.967 (0.937-0.989)	0.965 (0.942-0.981)	0.957 (0.936-0.995)	0.9400	0.9673	1.01 (0.942-1.08)	0.968 (0.929-1.04)	0.965 (0.954-1.01)	0.6900	0.8924
R848 (TLR7/8)	0.992 (0.913-1.02)	0.971 (0.955-0.986)	0.961 (0.938-0.995)	0.7330	0.8833	1.03 (0.966-1.05)	0.97 (0.957-1.08)	0.986 (0.956-1.01)	0.4900	0.8226
CpG (TLR9)	0.993 (0.954-1.04)	0.965 (0.929-1.02)	0.981 (0.97-0.993)	0.7470	0.8833	1.03 (1.01-1.05)	0.998 (0.942-1.03)	0.992 (0.96-1.01)	0.3130	0.7171
PMA-Iono	0.964 (0.953-1.03)	1 (0.956-1.2)	0.979 (0.944-1.03)	0.8100	0.8833	1.12 (1.03-1.21)	1.06 (1.02-1.15)	1.1 (1.05-1.15)	0.7690	0.8924
**pSTAT1**										
Basal	4.23 (3.41-5.26)	5.73 (4.31-7.16)	4.26 (3.9-4.93)	0.4630	0.8833	3.77 (3.56-5.53)	4.59 (3.67-7.06)	4.68 (3.32-6.48)	0.8080	0.9181
IFNα	5.39 (2.53-6.19)	4.96 (2.58-6.33)	2.34 (1.25-4.28)	0.3670	0.8833	4.98 (4.14-7.87)	4.05 (3.47-8.08)	3.53 (1.56-4.53)	0.1780	0.6763
LPS (TLR4)	1.02 (0.978-1.09)	1.16 (1.05-1.31)	0.991 (0.952-1.1)	0.0708	0.8833	1.09 (1.03-1.15)	1.1 (1.04-1.14)	0.998 (0.979-1.06)	0.1390	0.6763
R848 (TLR7/8)	0.982 (0.871-1.07)	1.01 (0.916-1.11)	0.961 (0.912-1.03)	0.7520	0.8833	1.1 (1.04-1.16)	1.05 (1-1.15)	0.987 (0.958-1.05)	0.3290	0.7171
CpG (TLR9)	0.969 (0.897-1.1)	0.976 (0.918-1.12)	0.967 (0.955-0.992)	0.6200	0.8833	0.981 (0.955-1.02)	0.995 (0.968-1.07)	0.947 (0.903-0.995)	0.0887	0.6763
PMA-Iono	1.15 (1.07-1.23)	1.47 (1.29-1.64)	1.12 (1.07-1.18)	**0.0456**	0.8833	1.33 (1.27-1.45)	1.31 (1.2-1.44)	1.15 (1.01-1.47)	0.4820	0.8226
**pSTAT3**										
Basal	87.4 (73.7-102)	84.1 (76.5-86.1)	88.5 (85.1-99.4)	0.4790	0.8833	73.7 (62.1-78.3)	75.7 (61.2-88.8)	67.9 (51.8-83.5)	0.7610	0.8924
IFNα	1.72 (1.27-2.04)	1.57 (1.32-2.05)	1.24 (0.931-2.09)	0.5270	0.8833	2.02 (1.86-2.35)	1.67 (1.4-2)	1.18 (1.1-1.57)	**0.0436**	0.5945

LPS (TLR4)	0.95 (0.92-0.979)	0.941 (0.909-1)	0.97 (0.841-0.981)	0.7510	0.8833	0.982 (0.913-0.999)	0.963 (0.94-1.03)	0.957 (0.955-0.988)	0.9270	0.9642
R848 (TLR7/8)	0.974 (0.93-1)	1 (0.923-1.02)	0.962 (0.954-1)	0.7890	0.8833	1.01 (0.96-1.02)	0.94 (0.898-0.968)	0.977 (0.957-1.01)	0.1260	0.6763
CpG (TLR9)	0.984 (0.936-1.04)	1 (0.94-1.05)	0.968 (0.923-0.992)	0.4120	0.8833	1.01 (0.993-1.02)	0.996 (0.944-1.04)	1.02 (0.996-1.04)	0.6370	0.8924
PMA-Iono	0.94 (0.874-0.944)	0.888 (0.832-0.908)	0.905 (0.852-0.929)	0.6560	0.8833	0.953 (0.852-1.03)	0.91 (0.875-0.952)	0.906 (0.859-1.04)	0.9370	0.9642
**pSTAT5**										
Basal	67.5 (59.1-90.4)	63.6 (55.7-74.3)	59.3 (53.9-77.2)	0.5590	0.8833	70.9 (68.1-74.2)	78.3 (67.8-91.1)	75.1 (69.8-80.6)	0.4450	0.809
IFNα	1 (0.949-1.09)	0.992 (0.923-1.07)	0.9 (0.788-1.1)	0.7430	0.8833	1.13 (1.02-1.19)	1.04 (0.805-1.1)	1.06 (0.956-1.1)	0.3550	0.7171
LPS (TLR4)	0.973 (0.918-0.997)	0.971 (0.947-1.01)	0.947 (0.902-0.998)	0.7360	0.8833	0.981 (0.939-1.05)	0.986 (0.97-1.04)	0.992 (0.964-1.03)	0.9900	0.9999
R848 (TLR7/8)	0.996 (0.95-1.01)	0.992 (0.954-1)	0.959 (0.927-0.998)	0.8000	0.8833	1.04 (0.941-1.05)	0.988 (0.908-1.02)	0.978 (0.966-1)	0.5320	0.8226
CpG (TLR9)	1 (0.932-1.04)	0.998 (0.97-1.02)	0.977 (0.879-0.997)	0.6330	0.8833	1.03 (1.01-1.05)	0.987 (0.962-1)	0.976 (0.959-1)	0.1320	0.6763
PMA-Iono	0.986 (0.951-1.03)	1.06 (1.01-1.11)	0.976 (0.95-1)	**0.0358**	0.8833	1.07 (0.972-1.19)	0.994 (0.975-1.05)	1.11 (0.954-1.19)	0.5130	0.8226
**Syk**										
Basal	25.2 (24.1-26.7)	24.4 (23-26.2)	24.4 (24.1-26.1)	0.7040	0.8833	23.3 (20.7-25)	24.4 (19.4-29.8)	22.9 (18.7-26.1)	0.7190	0.8924
IFNα	0.945 (0.938-0.969)	1.04 (1.01-1.13)	1.01 (0.921-1.06)	0.0986	0.8833	0.995 (0.941-1.02)	1.09 (0.928-1.19)	1.13 (1.03-1.14)	0.1480	0.6763

LPS (TLR4)	0.985 (0.943-1)	0.958 (0.925-1.09)	0.954 (0.923-1.02)	0.8260	0.8833	0.969 (0.922-1)	0.952 (0.925-1.03)	0.921 (0.91-0.999)	0.7460	0.8924
R848 (TLR7/8)	0.982 (0.952-1.02)	0.951 (0.943-0.96)	0.973 (0.935-1.01)	0.6400	0.8833	0.999 (0.979-1.05)	0.95 (0.893-0.97)	0.971 (0.931-0.995)	**0.0405**	0.5945
CpG (TLR9)	0.953 (0.934-1.02)	0.969 (0.942-0.993)	1.02 (0.983-1.02)	0.7760	0.8833	1.01 (0.988-1.04)	0.949 (0.92-1.02)	1 (0.949-1.03)	0.2730	0.7171
PMA-Iono	0.94 (0.921-0.989)	0.976 (0.911-1.04)	0.967 (0.933-1.01)	0.7800	0.8833	0.952 (0.927-1.01)	0.921 (0.847-0.965)	0.969 (0.906-1.06)	0.2560	0.7171

Supplemental Table 21. Demographics for the scRNA-seq replication study (GSE135779).

Names	Age	Sex	Race	Ethnicity	SLEDAI	sledai_class	study_group	PBMC_total
aHD2	50	F	White	NH	ND	CTRL	White CTRL	5754
aHD5	46	F	White	NH	ND	CTRL	White CTRL	7501
cHD1	7	F	White	NH	ND	CTRL	White CTRL	12170
cHD10	18	F	White	NH	ND	CTRL	White CTRL	5700
cHD11	17	F	White	NH	ND	CTRL	White CTRL	6199
cHD3	13	F	White	NH	ND	CTRL	White CTRL	8176
aSLE3	36	F	White	not reported	0	SLE.INACT	White SLE.INACT	6825
cSLE2	18	F	White	NH	0	SLE.INACT	White SLE.INACT	4819
cSLE4	17	F	White	NH	0	SLE.INACT	White SLE.INACT	5826
aSLE7	47	F	White	NH	6	SLE.ACT	White SLE.ACT	2964
cSLE22	17	F	White	NH	4	SLE.ACT	White SLE.ACT	3466
aHD1	39	F	Black	NH	ND	CTRL	Black CTRL	6586
aHD6	47	F	Black	NH	ND	CTRL	Black CTRL	10907
cHD5	12	F	Black	NH	ND	CTRL	Black CTRL	5058
cHD7	8	F	Black	NH	ND	CTRL	Black CTRL	6461
cHD8	8	F	Black	NH	ND	CTRL	Black CTRL	6826
cHD9	14	M	Black	NH	ND	CTRL	Black CTRL	6792
aSLE8	62	F	Black	NH	2	SLE.INACT	Black SLE.INACT	5777
cSLE30	10	F	Black	NH	0	SLE.INACT	Black SLE.INACT	7036
cSLE7	17	F	Black	NH	0	SLE.INACT	Black SLE.INACT	3575
cSLE8	16	F	Black	NH	2	SLE.INACT	Black SLE.INACT	5712
aSLE1	37	F	Black	NH	15	SLE.ACT	Black SLE.ACT	3855
cSLE11	17	F	Black	NH	8	SLE.ACT	Black SLE.ACT	8711
cSLE16	13	F	Black	NH	6	SLE.ACT	Black SLE.ACT	4995
cSLE19	16	F	Black	NH	6	SLE.ACT	Black SLE.ACT	6182
cSLE21	13	F	Black	NH	6	SLE.ACT	Black SLE.ACT	2379
cSLE23	17	F	Black	NH	6	SLE.ACT	Black SLE.ACT	3037

cSLE26	17	F	Black	NH	6	SLE.ACT	Black SLE.ACT	5319
cSLE27	15	F	Black	NH	19	SLE.ACT	Black SLE.ACT	5450

Supplemental Table 22. Comparisons of plasma soluble mediator levels in White (White) and Black (Black) controls and systemic lupus erythematosus (SLE) with inactive (INACT) and active (ACT) disease activity.

	Mean MFI (IQR)					Mean MFI (IQR)				
Soluble Mediator	White Controls	White SLE INACT	White SLE ACT	KW p-value	FDR Q-value	Black Controls	Black SLE INACT	Black SLE ACT	KW p-value	FDR Q-value
BLyS	91 (74.375-95.5)	81.5 (73.25-93.875)	75 (70-83)	0.5260	0.8376	83.5 (76.5-90.125)	87 (85-117)	91 (80.25-119.75)	0.4380	0.4913
CXCL13	43.5 (38.375-71)	57.5 (39.75-124.375)	44.5 (36-94)	0.8310	0.9205	41.5 (30.875-51.125)	58 (42-101)	94 (52.75-268.25)	0.0700	0.4552
Eotaxin	290 (66.25-927.5)	222.25 (152-301.5)	132 (90-460.5)	0.5940	0.8376	40.5 (19.25-179.75)	29 (21-55.5)	46 (29.5-98.625)	0.3790	0.4913
GM-CSF	6 (5-7.5)	10 (6.5-12.75)	8 (7-9)	**0.0450**	0.5909	6.5 (6-7.25)	7 (6-8.5)	9 (7-18.25)	0.1880	0.4657
GRO-α	43 (36.5-50)	44.5 (42.125-70.375)	42 (39-74)	0.6250	0.8376	39.25 (36.5-62.25)	58.5 (41.5-85.5)	60.5 (48.75-82.75)	0.2870	0.48
IFN-α	180 (158.875-183)	151.5 (134.5-209)	185 (140.5-224)	0.6900	0.8376	131.25 (121.375-217.75)	189 (144-227)	178 (130.75-213.375)	0.5350	0.5546
IFN-γ	30.5 (22.75-40)	32.5 (25.25-49.25)	37 (30-55)	0.4920	0.8376	24.5 (20.5-35.875)	45 (29.5-48)	44.5 (40-90.25)	0.1740	0.4657
IL-1α	352.5 (328.75-405)	356.5 (317.125-377)	321.5 (297-360)	0.6500	0.8376	363.5 (328.375-407.75)	387 (312-431.5)	352 (325-376.25)	0.4430	0.4913
IL-1β	9 (6.75-10)	10 (8.25-10.75)	12 (10-13)	0.3160	0.8365	8 (7-9.75)	10 (8-12)	18 (10.75-22.875)	0.0982	0.4552
IL-1RA	59 (36.125-121)	43 (37.375-59.25)	61 (54-68)	0.4430	0.8376	65 (45-91.25)	59 (48-90.5)	106 (60-134.25)	0.4240	0.4913
IL-10	16 (11.75-22.5)	18 (16.25-20.625)	20 (17-33)	0.2110	0.8311	17.25 (14.375-18.625)	26 (18-43)	27.5 (16.75-74.5)	0.1320	0.4657
IL-12p70	9 (7.875-10)	9.5 (8.25-11.5)	11 (8-15.5)	0.5480	0.8376	7.5 (6.75-10.5)	10.5 (9-11)	12 (10.5-38.25)	0.0922	0.4552
IL-13	10 (9-12)	10.5 (10-12.875)	12 (8.5-16)	0.7060	0.8376	10.75 (9-16.75)	15 (12-19)	19 (12.5-27.875)	0.3070	0.48
IL-15	11 (10-17)	19.75 (13.25-36.5)	17 (15-19.5)	0.1950	0.8311	13 (9-23.5)	17 (13-19)	22 (15-51)	0.2370	0.4668
IL-17A	8 (6.875-9.5)	15 (9.5-24.5)	11.5 (11-17)	**0.0281**	0.5534	9.5 (7.75-10.75)	10.5 (8-15)	12 (9.5-33.5)	0.4270	0.4913
IL-18	29.5 (20.25-36.5)	31.25 (25.125-46.125)	39 (18-82)	0.6490	0.8376	19 (14.875-28)	25 (20-29)	72 (24.5-168.75)	0.0975	0.4552

IL-2	7.5 (5.75-11)	18.5 (10.75-24)	13.5 (9-20.5)	**0.0259**	0.5534	8.5 (6-11.5)	11 (7-12)	10.5 (9-18.5)	0.3970	0.4913
IL-21	55.5 (39.5-124)	51.5 (40-68.5)	56 (37-67)	0.8720	0.9205	37 (29.25-82)	45.5 (35.5-105)	48 (34.25-66.375)	0.6090	0.6151
IL-22	17 (14.375-27)	24 (18.25-39.375)	27 (15-31.5)	0.4810	0.8376	17.75 (14.875-21.875)	19 (18-29)	24 (18-56.375)	0.3290	0.48
IL-23	14 (11.5-16)	14.5 (13.25-22.25)	16 (12-27.5)	0.7230	0.8376	11.75 (9.875-21)	16 (15-17)	17 (13.75-37.25)	0.3150	0.48
IL-27	9 (7.5-17)	13.5 (9.25-24.875)	15 (11-19.5)	0.3300	0.8365	10.75 (7.25-15)	16 (10-19)	22 (13.5-46.25)	0.1630	0.4657
IL-31	15 (12.75-19)	17.5 (15.875-24.125)	17 (14-19)	0.3970	0.8376	13.25 (11.75-18.25)	16 (15-20)	18 (13-35.625)	0.4450	0.4913
IL-4	23 (12.25-33)	15.5 (12.5-20.5)	25 (20-25.5)	0.2990	0.8365	18 (15.75-24.25)	27 (21-28)	31 (24-43.5)	0.1170	0.4609
IL-5	8 (7-9)	12.25 (8.875-14.625)	11 (8-15)	0.1160	0.6753	7.5 (7-8.75)	9 (9-14)	11 (9.5-20)	0.1040	0.4552
IL-6	11 (10.5-13.5)	12 (10.25-14)	12 (11-14)	0.7010	0.8376	12 (10.125-13.25)	10.5 (10-18)	18 (11.5-25.875)	0.0868	0.4552
IL-7	8 (8-9)	9.5 (8.25-10.875)	9 (8-10.5)	0.5850	0.8376	7 (7-9.25)	7 (7-8)	10 (8.5-12.75)	**0.0493**	0.4552
IL-8	26 (18.375-37)	26.5 (21.5-34)	25 (17-39.5)	0.9700	0.9797	20.75 (16.75-51.25)	30 (20-41.5)	47 (23.5-57)	0.3220	0.48
IL-9	12 (9.75-14.5)	18.75 (12-24.375)	11.5 (10-25.5)	0.2330	0.8344	13.75 (10.75-22)	11 (10-17)	25.5 (15.75-34.125)	0.0977	0.4552
IP-10	74 (61.75-103)	62.5 (49.5-73.75)	61 (47-196)	0.5990	0.8376	65.25 (37.75-102.5)	44 (41-72)	61 (50-167)	0.4830	0.5142
MCP-1	59 (45.75-139)	102 (71.75-149.125)	66.5 (42-375)	0.8680	0.9205	54.5 (36.25-131.25)	33 (22-95)	149 (69.75-564.75)	**0.0224**	0.4552
MIP-1α	105 (53.5-334)	404.25 (226.125-517.25)	389 (74-607)	0.1600	0.7878	66.25 (51.25-247.875)	244.5 (109.5-301.5)	130 (99.75-877)	0.2290	0.4668
MIP-1β	118.5 (96.75-226)	98 (87.625-161.875)	87 (71-93)	0.1200	0.6753	85.25 (68.75-204.5)	104 (85-192)	162 (118.5-241.375)	0.4490	0.4913
RANTES	2279 (1831.75-2938)	1976 (1565.5-2362.75)	2238.5 (1751-2786)	0.8880	0.9205	1599 (1245.625-2664.125)	2072 (1739-4729)	1834 (1367.5-2925.125)	0.3090	0.48
sCD40L	223.5 (141-560.5)	590.5 (353.75-738.5)	399.5 (157.5-972)	0.3590	0.8365	118.75 (77.25-353)	253 (116-465)	196 (133.5-1179.5)	0.4170	0.4913

SCF	69.5 (36.5-78)	39.5 (29.625-51.625)	74.5 (59-112)	0.1010	0.6753	58.5 (26.375-92)	67 (51-89)	95 (69.25-131.5)	0.2000	0.4657
SDF-1α	800 (741.125-860)	640.25 (632.25-786.625)	741.5 (720.5-795)	0.5020	0.8376	700.5 (630.75-774.375)	765.5 (702-990.5)	757 (674.75-863.75)	0.2710	0.48
sICAM-1	6815 (6215.25-7714.5)	3968.5 (2837.25-4617.875)	7465 (5121-7841.5)	0.0624	0.6145	5908 (4090.375-6942.625)	8052.5 (4763-9687)	7577 (6132.75-8394.625)	0.2010	0.4657
TNF-α	22 (19.5-26)	28 (20.875-31)	25 (19-31)	0.3610	0.8365	23.25 (20.75-28)	27 (23-39.5)	39.5 (30.25-49.75)	0.1870	0.4657
TNF-β	25 (20.875-29)	33 (27-43.375)	30 (25.5-47)	0.3350	0.8365	20.5 (17.375-36.375)	26.5 (22.5-40.5)	30.5 (24.5-78.625)	0.2190	0.4668

Supplemental Table 23. Significance (p-values) of plasma soluble mediator levels across disease groups.

	White			Black					
**Soluble Mediator**	**Ctrl vs SLE INACT**	**Ctrl vs SLE ACT**	**SLE INACT vs SLE ACT**	**Ctrl vs SLE INACT**	**Ctrl vs SLE ACT**	**SLE INACT vs SLE ACT**	**White Ctrl vs Black Ctrl**	**White SLE INACT vs Black SLE INACT**	**White SLE ACT vs Black SLE ACT**
BLyS	0.5950	0.2330	0.6230	0.3350	0.2150	0.8790	0.5640	0.2700	**0.0365**
CXCL13	0.5140	0.8950	0.7440	0.1240	**0.0318**	0.3230	0.5310	0.9350	0.2390
Eotaxin	0.4140	0.4020	0.6240	0.6300	0.6800	0.1190	**0.0343**	**0.0055**	0.0742
GM-CSF	**0.0211**	0.0616	0.5370	0.4620	0.1040	0.1940	0.3040	0.2850	0.4910
GRO-α	0.4140	0.4800	0.5950	0.2470	0.1370	0.6480	0.9230	0.5670	0.2390
IFN-α	0.8060	0.3540	0.5680	0.2900	0.4570	0.6210	0.8470	0.4140	0.7900
IFN-γ	0.5670	0.3100	0.3690	0.1940	0.0985	0.3420	0.5310	0.5140	0.3420
IL-1α	0.6830	0.4020	0.5130	0.2680	0.5630	0.3050	0.8470	0.3270	0.5180
IL-1β	0.3880	0.1990	0.2840	0.3080	0.0820	0.0870	0.8080	0.8690	0.2230
IL-1RA	0.4140	0.8950	0.1770	0.9620	0.4090	0.1840	0.8470	0.1650	0.1020
IL-10	0.3680	0.0848	0.3460	0.0537	0.1070	0.9700	0.5960	0.1020	0.5680
IL-12p70	0.6210	0.2470	0.6220	0.1480	0.0749	0.1380	0.3560	0.6220	0.1820
IL-13	0.3830	0.5940	0.7100	0.3110	0.1860	0.3610	0.5580	**0.0484**	0.1100
IL-15	0.0783	0.1830	0.9350	0.7720	0.1260	0.1960	0.9610	0.3900	0.3820
IL-17A	**0.0138**	**0.0337**	0.8380	0.6290	0.2000	0.4460	0.3080	0.1880	0.8790
IL-18	0.3070	0.6270	0.8700	0.3600	0.0630	0.1100	0.3860	0.1530	0.3820
IL-2	**0.0126**	**0.0422**	0.4870	0.3320	0.2470	0.4460	0.8090	**0.0491**	0.7040
IL-21	0.5670	0.7570	1.0000	0.2900	0.5910	0.7040	0.4130	0.6530	0.6210
IL-22	0.1910	0.5650	0.7130	0.3120	0.1600	0.5680	0.7720	0.6820	0.6480
IL-23	0.6230	0.4510	0.6820	0.3850	0.1600	0.4030	0.5960	0.7430	0.5180
IL-27	0.1400	0.2870	0.9670	0.3860	0.0756	0.2390	1.0000	0.9350	0.2700
IL-31	0.1640	0.5350	0.5360	0.3600	0.2140	0.8190	0.4400	0.5950	0.7030

IL-4	0.5950	0.7230	0.0656	0.1770	0.0826	0.1830	0.9620	**0.0159**	0.1380
IL-5	**0.0442**	0.1540	0.5940	0.1890	**0.0380**	0.4010	0.9220	0.5390	0.5680
IL-6	0.5110	0.4510	0.8050	0.6640	0.0626	0.0676	0.9610	0.8370	0.1690
IL-7	0.3010	0.5890	0.6490	0.7940	**0.0446**	**0.0383**	0.1820	**0.0311**	0.3980
IL-8	0.8700	0.8250	0.9020	0.5640	0.1600	0.3230	1.0000	0.6240	0.1710
IL-9	0.0597	0.6900	0.4130	0.7340	0.0829	0.0622	0.3590	0.2020	0.1020
IP-10	0.2530	0.8250	0.6530	0.8850	0.5630	0.1830	0.5960	0.6230	0.8490
MCP-1	0.5680	0.8600	0.8060	0.1490	0.1170	**0.0109**	0.7730	0.0723	0.2240
MIP-1α	0.0604	0.2000	0.6240	0.1020	0.1860	0.7320	0.5640	0.1650	0.7900
MIP-1β	0.3690	**0.0469**	0.2050	0.4410	0.2150	0.6210	0.2900	0.6240	**0.0109**
RANTES	0.6830	0.8950	0.6830	0.1490	0.5090	0.3050	0.3360	0.6240	0.3820
sCD40L	0.1420	0.4530	0.5950	0.2480	0.2470	0.9700	0.4410	0.1020	0.7320
SCF	0.1020	0.5660	0.0549	0.5010	0.1170	0.1840	0.8470	**0.0143**	0.4700
SDF-1α	0.4620	0.2000	0.5680	0.1490	0.4090	0.2710	0.2480	0.0724	0.7900
sICAM-1	0.0338	0.8950	0.0604	0.1780	0.0829	0.6760	0.2900	**0.0114**	0.4250
TNF-α	0.1640	0.3530	0.6530	0.3360	0.0982	0.2540	0.6640	0.5400	0.1020
TNF-β	0.1640	0.2510	1.0000	0.1940	0.1160	0.4940	0.6650	0.6230	0.7040

Supplemental Table 24. Fold change (stimulated over unstimulated fluorescence intensity) of cytokine levels following TLR stimulation (R848/CpG/LPS cocktail).

	Median Fold Change (IQR)					Median Fold Change (IQR)				
**Soluble Mediator**	**White Controls**	**White SLE INACT**	**White SLE ACT**	**KW p-value**	**FDR q-value**	**Black Controls**	**Black SLE INACT**	**Black SLE ACT**	**KW p-value**	**FDR q-value**
BLyS	1.22 (1.18-1.3)	1.22 (1.16-1.31)	1.16 (1.13-1.36)	0.9000	0.9526	1.23 (1.06-1.36)	1.22 (1.09-1.28)	1.12 (0.971-1.44)	0.8400	0.8935
CXCL13	1.63 (1.46-2.24)	1.44 (0.914-1.53)	1.11 (0.995-2.2)	0.1330	0.2698	1.89 (1.33-3.56)	1.35 (1.12-1.95)	0.938 (0.875-1.4)	0.1580	0.6499
Eotaxin	1.21 (1.11-1.32)	1.18 (0.928-1.37)	1.35 (1.22-1.75)	0.2210	0.3873	1.63 (1.14-1.98)	2.02 (1.65-2.22)	1.55 (1.29-1.88)	0.2340	0.709
GM-CSF	2.47 (2-3.9)	2.03 (1.74-2.82)	2.46 (2.09-3.99)	0.4210	0.5121	2.83 (1.63-3.44)	2.33 (1.81-2.98)	2.33 (1.88-3.49)	0.9970	1
GRO-α	1.5 (1.27-1.79)	0.707 (0.382-1.7)	2.44 (1.59-3.3)	**0.0193**	0.1875	1.39 (0.958-2.61)	1.37 (1.3-2.47)	1.47 (1.37-1.72)	0.8300	0.8935
IFN-α	9.61 (5.51-12.2)	3.87 (3.09-5.43)	4.78 (2.23-9.01)	0.0957	0.2698	7.83 (4.72-14.8)	10.4 (6.99-12.6)	4.92 (2.35-7.14)	0.1650	0.6499
IFN-γ	21.3 (14.6-23.8)	14.1 (3.48-28.7)	3.43 (2.62-6.56)	**0.0072**	0.1875	16 (4.94-30.2)	10.2 (5-14.3)	2.37 (1.44-5.66)	**0.0363**	0.3978
IL-1α	3.87 (2.5-4.36)	2.08 (1.58-3.63)	3.87 (2.77-4.79)	0.1370	0.2698	4.12 (3.19-5.16)	2.93 (2.19-3.49)	3.09 (2.2-4.17)	0.4750	0.8309
IL-1β	220 (170-243)	141 (63.9-240)	166 (127-239)	0.3170	0.446	169 (104-186)	172 (147-212)	87.5 (70.9-119)	**0.0362**	0.3978
IL-1RA	22.4 (12.9-32.1)	9.67 (5.78-15.1)	31.5 (15.2-57.5)	0.0618	0.2698	13.7 (10.2-23.2)	21.6 (11.3-36.5)	8.55 (6.26-10.4)	0.0532	0.4191
IL-10	15.5 (11.7-32.3)	3.48 (1.83-12.3)	43.9 (14-64.6)	**0.0238**	0.1875	25.2 (14.7-38.7)	10.5 (7.98-20.3)	6.78 (3.32-13)	0.1850	0.6499
IL-12p70	254 (187-331)	156 (56-463)	117 (57.8-217)	0.2350	0.3873	58.1 (44.3-177)	94.6 (50.9-277)	81.8 (11.5-93.1)	0.2520	0.709
IL-13	2.06 (1.78-2.28)	2.38 (1.69-2.94)	1.77 (1.45-2.27)	0.3830	0.5121	1.4 (1.24-1.99)	1.75 (1.56-2.31)	1.63 (1.04-2.1)	0.6750	0.8309
IL-15	1.41 (1.22-1.8)	1.82 (1.43-2.63)	1.64 (1.55-2.02)	0.4160	0.5121	1.86 (1.05-2.29)	1.67 (1.34-2.26)	1.3 (1-2.21)	0.8620	0.8935
IL-17A	1.23 (1.2-1.5)	1.09 (1-1.16)	1.4 (1.24-1.5)	**0.0195**	0.1875	1.36 (1.19-1.45)	1.38 (1.04-1.48)	1.08 (0.992-1.46)	0.5520	0.8309
IL-18	6.22 (4.72-7.27)	4.3 (2.27-6.89)	2.85 (2.37-3.29)	**0.0112**	0.1875	5.72 (2.72-8.51)	4.51 (3.02-7.05)	2.1 (1.34-3.2)	**0.0242**	0.3978

IL-2	2.68 (2.35-2.81)	2.2 (1.85-2.76)	2.38 (2.16-3.07)	0.6410	0.7014	2.42 (1.73-2.93)	2.56 (2.19-2.97)	2.13 (1.57-2.55)	0.3240	0.7976
IL-21	2.02 (1.65-2.57)	2.13 (1.58-2.33)	1.99 (1.87-2.28)	0.9780	0.9878	2.35 (1.41-2.57)	2.18 (1.8-2.43)	1.58 (1.36-1.78)	0.1170	0.5761
IL-22	5.06 (4.02-6.31)	4.78 (2.26-6.2)	3.86 (2.6-5.3)	0.4290	0.5121	4.03 (3.24-5.01)	4.26 (2.84-5.05)	3.04 (2-4.3)	0.4670	0.8309
IL-23	17.2 (12.6-27.5)	13.5 (7.96-20.5)	10.8 (6.48-14.8)	0.2690	0.3983	13.2 (9.22-17.3)	11.8 (7.48-17.6)	8.25 (4.06-11.7)	0.3510	0.8133
IL-27	1.42 (1.27-1.86)	2.16 (1.58-2.69)	1.58 (1.29-2.03)	0.2360	0.3873	1.45 (0.984-1.98)	1.7 (1.42-2.08)	1.49 (0.954-1.63)	0.7080	0.8451
IL-31	1.3 (1.18-1.55)	1.39 (1.2-1.71)	1.17 (0.984-1.94)	0.5940	0.6685	1.58 (1.11-2.16)	1.71 (1.24-1.91)	1.36 (1.11-1.48)	0.5110	0.8309
IL-4	2.1 (1.71-2.86)	2.22 (2.11-2.62)	1.53 (1.21-2.34)	0.2730	0.3983	2.09 (1.08-3.73)	1.92 (1.55-2.18)	1.75 (1.3-2.06)	0.6040	0.8309
IL-5	2.69 (2.3-2.85)	2.49 (2.26-3.17)	2.84 (2.35-3.11)	0.9190	0.9526	2.51 (1.29-2.92)	2.72 (2.03-2.87)	2 (1.74-2.91)	0.5710	0.8309
IL-6	86.8 (60.7-118)	34.7 (16.2-55.2)	75.6 (56.4-131)	0.1160	0.2698	68.5 (44.6-98.1)	78.7 (51.4-113)	22 (16.7-65.5)	0.1160	0.5761
IL-7	1.43 (1.3-1.49)	1.22 (1.04-1.39)	1.5 (1.36-1.68)	0.1340	0.2698	1.51 (1.08-1.69)	1.71 (1.11-2.31)	1.71 (1.05-1.92)	0.6310	0.8309
IL-8	10.6 (4.95-17.8)	2.29 (1.16-9.41)	9.82 (4.34-53.3)	0.1270	0.2698	5.62 (4.47-9.07)	12.9 (3.92-19.2)	10.6 (4.81-14.1)	0.5220	0.8309
IL-9	4.14 (2.17-5.59)	2.02 (1.52-4.23)	4.43 (2.64-11.8)	0.1190	0.2698	4.15 (2.93-4.8)	4.88 (2.84-6.29)	4.19 (3.19-4.57)	0.4880	0.8309
IP-10	5.64 (4.94-6.19)	4.08 (3.46-5.15)	4.12 (2.02-5.23)	0.1370	0.2698	4.2 (2.55-5.36)	4.12 (2.48-6.14)	2.81 (1.65-3.84)	0.2880	0.7563
MCP-1	2.03 (0.951-5.97)	0.53 (0.412-3.73)	2.93 (1.71-4.58)	0.4150	0.5121	1.34 (0.872-9.22)	2.48 (1.08-10.4)	1.55 (0.741-4.38)	0.5740	0.8309
MIP-1α	46.4 (23.5-73.1)	14.1 (7.5-28.6)	67.3 (39.5-107)	0.0936	0.2698	38.6 (21.6-49.6)	52.5 (36.5-76.9)	20.1 (7.81-39.5)	0.0761	0.4996
MIP-1β	33.2 (6.95-59.6)	4.25 (1.96-38.4)	62.7 (18.9-91.2)	0.0891	0.2698	43.2 (17.2-52.6)	39.5 (20.1-61.2)	14 (3.48-43.5)	0.1980	0.6499
RANTES	1.14 (0.833-1.3)	0.907 (0.749-1.06)	1.17 (0.789-1.4)	0.5570	0.6453	0.996 (0.844-1.36)	1.04 (0.693-1.4)	1.12 (0.988-1.51)	0.6640	0.8309
sCD40L	1.16 (1.06-1.34)	1.15 (1.02-1.33)	1.32 (1.24-1.41)	0.2050	0.3845	1.07 (0.967-1.43)	1.18 (1.05-1.29)	1.11 (0.997-1.13)	0.5860	0.8309
SCF	1.55 (1.33-1.87)	1.57 (1.42-1.69)	1.21 (1.01-1.41)	0.1250	0.2698	1.26 (0.974-1.79)	1.58 (1.08-1.9)	1.22 (0.909-1.7)	0.6560	0.8309

SDF-1α	1.57 (1.25-1.7)	1.03 (0.889-1.49)	1.67 (1.46-1.92)	**0.0494**	0.2698	1.37 (1.32-1.84)	1.34 (1.08-2.02)	1.54 (1.27-2.08)	0.7980	0.8935
sICAM-1	0.968 (0.899-1.08)	1.1 (1.01-1.16)	1.13 (1.08-1.19)	0.0643	0.2698	0.955 (0.789-1.21)	1.05 (0.992-1.16)	1.12 (0.958-1.2)	0.7850	0.8935
TNF-α	134 (119-144)	105 (90.8-116)	97.2 (85-122)	**0.0341**	0.2239	96.7 (62.2-144)	87.1 (66.8-106)	42.3 (19.3-68.9)	**0.0404**	0.3978
TNF-β	1.31 (1.21-1.45)	1.57 (1.38-1.72)	1.53 (1.19-1.75)	0.2590	0.3983	1.08 (0.935-1.55)	1.59 (1.03-1.71)	1.4 (1.2-1.48)	0.6060	0.8309

Supplemental Table 25. Significance (p-values) of soluble mediator fold change differences following TLR stimulation across disease groups.

	White			Black					
**Soluble Mediator**	**Ctrl vs SLE INACT**	**Ctrl vs SLE ACT**	**SLE INACT vs SLE ACT**	**Ctrl vs SLE INACT**	**Ctrl vs SLE ACT**	**SLE INACT vs SLE ACT**	**White Ctrl vs Black Ctrl**	**White SLE INACT vs Black SLE INACT**	**White SLE ACT vs Black SLE ACT**
BLyS	0.9400	0.6570	0.7220	0.9290	0.8690	0.4810	0.7900	0.7050	0.3640
CXCL13	0.0538	0.1830	0.5940	0.4770	0.1600	0.0783	0.6570	0.4960	0.2480
Eotaxin	0.8210	0.1310	0.1310	0.2140	0.9340	0.1050	0.1100	**0.0032**	0.4090
GM-CSF	0.3250	0.8590	0.2140	0.9650	0.9340	0.9720	0.7220	0.7910	0.6200
GRO-α	0.1990	0.0756	**0.0077**	0.8590	0.5630	0.6730	0.7220	0.0963	0.2150
IFN-α	**0.0284**	0.1830	0.7220	0.7220	0.2480	0.0573	0.9290	**0.0102**	0.9340
IFN-γ	0.4960	**0.0004**	0.1100	0.6570	**0.0390**	**0.0242**	0.5940	0.8800	0.3220
IL-1α	0.0963	0.6570	0.0914	0.2860	0.2830	1.0000	0.5340	0.1990	0.3640
IL-1β	0.1990	0.2480	0.4770	0.5940	0.0829	**0.0137**	0.1100	0.4500	**0.0132**
IL-1RA	0.0963	0.3280	**0.0330**	0.3280	0.1600	**0.0201**	0.3280	0.0696	**0.0132**
IL-10	**0.0284**	0.3280	**0.0209**	0.4770	0.0829	0.2310	0.7220	0.0588	**0.0475**
IL-12p70	0.3640	0.0756	0.5340	0.2860	0.6800	0.1050	**0.0059**	0.5450	0.1860
IL-13	0.3260	0.4240	0.2480	0.4240	0.8690	0.4810	0.0914	0.2900	0.5090
IL-15	0.1860	0.3740	1.0000	0.9290	0.8040	0.5490	0.5340	0.7620	0.5090
IL-17A	**0.0220**	0.6860	**0.0137**	0.8940	0.4320	0.3060	0.9290	0.2080	0.1600
IL-18	0.1740	**0.0007**	0.3740	0.5940	**0.0208**	**0.0242**	0.5940	0.7050	0.2480
IL-2	0.4270	0.8240	0.4240	0.5940	0.5360	0.1130	0.5050	0.4730	0.1070
IL-21	0.8800	0.7900	0.9290	0.7900	0.2150	**0.0290**	0.9290	0.5450	**0.0390**
IL-22	0.4060	0.1830	0.7900	0.7900	0.3640	0.2600	0.2140	0.7620	0.4090
IL-23	0.2900	0.1310	0.4770	0.9290	0.1860	0.2600	0.3740	0.5450	0.4570
IL-27	0.1210	0.7560	0.1970	0.5340	0.9340	0.4390	0.7220	0.2900	0.4570
IL-31	0.5710	0.5940	0.3280	0.7900	0.4570	0.2600	0.4240	0.7050	0.6800

IL-4	0.6770	0.3280	0.0914	0.8590	0.3640	0.4390	0.8590	0.1120	1.0000
IL-5	0.8800	0.5340	0.9290	0.4240	0.9340	0.3240	0.5630	1.0000	0.4090
IL-6	0.0696	0.7220	0.0914	0.7220	0.0829	0.0783	0.3740	0.1310	0.0986
IL-7	0.1980	0.3050	0.0681	0.4770	0.4570	0.5030	0.6570	0.1400	0.8040
IL-8	0.1310	0.5940	0.0621	0.2860	0.4090	0.6730	0.3740	0.0588	0.6200
IL-9	0.1990	0.4770	**0.0410**	0.3280	0.8690	0.2910	0.7220	0.0696	0.5630
IP-10	0.0963	0.0914	0.6570	0.7220	0.3220	0.1210	0.1100	0.8210	0.3220
MCP-1	0.2260	0.5940	0.3280	0.5940	0.6800	0.2910	0.7900	0.1510	0.4090
MIP-1α	0.1120	0.2860	0.0621	0.3280	0.1600	**0.0346**	0.4240	**0.0343**	**0.0208**
MIP-1β	0.1310	0.2860	0.0506	1.0000	0.1170	0.1390	0.6570	**0.0413**	0.0829
RANTES	0.4060	0.7900	0.3280	0.7900	0.4090	0.4810	0.8590	0.7050	0.8690
sCD40L	0.9700	0.0914	0.1550	0.7900	0.9340	0.2050	0.5940	1.0000	**0.0030**
SCF	0.9400	0.0912	0.0618	0.6250	0.9340	0.3240	0.2480	0.8210	0.8040
SDF-1α	0.0963	0.5340	**0.0164**	0.6570	0.5630	0.6220	0.9290	0.0821	0.6200
sICAM-1	0.0696	**0.0410**	0.4240	0.6570	0.4570	0.9440	1.0000	0.6500	0.4090
TNF-α	**0.0126**	0.0621	0.9290	0.6570	**0.0390**	**0.0290**	0.1310	0.4500	**0.0166**
TNF-β	0.0963	0.3740	0.5940	0.3510	0.5630	0.5730	0.4500	0.5970	0.4090

Supplemental Table 26. Fold change (stimulated over unstimulated fluorescence intensity) of cytokine levels following PMA/ionomycin stimulation.

Soluble Mediator	Median Fold Change (IQR)			KW p-value	FDR q-value	Median Fold Change (IQR)			KW p-value	FDR q-value
	White Controls	White SLE INACT	White SLE ACT			Black Controls	Black SLE INACT	Black SLE ACT		
BLyS	1.28 (1-1.64)	1.21 (1.15-1.39)	1.22 (1.12-1.67)	0.9350	0.9692	1.03 (0.95-1.19)	1.37 (1.3-1.55)	1.34 (1.05-1.5)	0.0797	0.2232
CXCL13	2.33 (1.88-3.22)	1.95 (1.68-2.77)	2.42 (1.42-7.57)	0.9050	0.9635	3.23 (2.29-6.53)	3.26 (2.66-5.26)	3.2 (2.32-3.64)	0.9910	1
Eotaxin	0.867 (0.678-1)	0.908 (0.737-1.15)	1.06 (0.65-1.23)	0.6310	0.8018	1.09 (0.827-1.67)	1.93 (1.56-2.1)	1.88 (1.42-1.99)	**0.0479**	0.221
GM-CSF	35.2 (21.9-57.4)	66.7 (16.8-96.4)	16 (12.2-61.2)	0.4990	0.7353	21.2 (6.5-38.3)	18.5 (13.8-65.1)	21.4 (11-39.6)	0.6860	0.7111
GRO-α	3.72 (2.02-4.06)	3.2 (0.757-9.66)	5.04 (3.38-6.75)	0.3780	0.6474	2.79 (1.91-3.8)	4.05 (3.09-5.1)	2.82 (2.22-3.76)	0.3930	0.4838
IFN-α	1.23 (1.17-1.36)	1.13 (1.01-1.42)	1.61 (1.44-1.73)	0.1220	0.4977	1.24 (1.14-1.28)	1.56 (1.11-1.74)	1.43 (1.26-1.52)	0.4570	0.5294
IFN-γ	108 (82.5-114)	128 (39.9-155)	60.1 (30-69.2)	0.1340	0.4977	89.3 (46.7-112)	71.5 (55.2-124)	42 (22.7-69.6)	0.0688	0.2232
IL-1α	2.01 (1.78-2.08)	1.53 (1.03-2)	2.15 (1.85-2.81)	0.2980	0.6059	2.08 (1.55-2.16)	2.13 (1.72-2.43)	2.2 (1.62-2.24)	0.6540	0.6984
IL-1β	5.52 (4.48-8.06)	7.33 (5.16-12)	3.18 (2.43-9.59)	0.1390	0.4977	3.6 (1.42-5.91)	4.39 (3.39-5.5)	3.23 (2.27-4.91)	0.4510	0.5294
IL-1RA	24.7 (13.3-39.2)	12.4 (7.23-24.4)	32.5 (20.6-37.5)	0.2580	0.6059	22.6 (19.6-29.8)	31.7 (18.6-53.8)	10.9 (7.75-17.3)	**0.0294**	0.221
IL-10	215 (135-303)	95.5 (51.5-203)	54.4 (35.5-102)	**0.0340**	0.4464	140 (65.9-171)	171 (119-243)	45.2 (24.5-73.7)	**0.0242**	0.221
IL-12p70	4.45 (2.76-7.44)	4.54 (3.4-5.61)	4.36 (2.04-6.2)	0.8830	0.9635	3.72 (2.3-5.78)	5.62 (3.33-8.42)	2.8 (1.49-4.6)	0.1580	0.3271
IL-13	53.9 (41-77.5)	74.7 (36.6-149)	23 (12.9-84.3)	0.1930	0.5543	33.5 (13.1-46.6)	60.7 (22.2-134)	58.3 (21.2-68.9)	0.2940	0.4289
IL-15	1.72 (1.25-3.07)	2.83 (1.48-3.46)	2.72 (1.84-4.29)	0.6600	0.8124	1.21 (0.983-1.86)	1.98 (1.51-2.82)	1.93 (1.3-3.61)	0.1910	0.3271
IL-17A	619 (399-806)	1050 (364-1610)	322 (98.5-854)	0.4320	0.6807	985 (510-1530)	1570 (914-2170)	662 (303-916)	0.0780	0.2232
IL-18	22.2 (17.2-28.4)	21.7 (11.8-24.5)	13.6 (6.29-18.1)	0.0505	0.4973	23.6 (15.2-26.4)	19.6 (16.9-24.7)	6.09 (4.59-18.2)	**0.0449**	0.221

IL-2	1060 (939-1230)	954 (816-1030)	794 (566-1060)	0.1910	0.5543	905 (467-1010)	970 (841-1050)	804 (302-890)	0.1830	0.3271
IL-21	8.58 (4.75-9.72)	7.93 (5.27-10.3)	8.11 (4.56-10.2)	0.9980	1	6.15 (2.23-9.39)	8.62 (6.82-13)	4.29 (2.46-8.66)	0.2550	0.4018
IL-22	9.89 (5.69-31.9)	52.1 (10.3-172)	5.21 (3.21-10.5)	0.1160	0.4977	21.3 (6.09-32.6)	28.3 (15.9-135)	10.4 (6.24-19.4)	0.1690	0.3271
IL-23	2.87 (1.47-4.1)	4.58 (2.61-6.86)	3.98 (3.5-7.18)	0.3080	0.6059	1.58 (1.02-2.65)	3.01 (2.56-5.35)	3.56 (2.76-4.69)	0.0505	0.221
IL-27	1.51 (1.28-3.27)	3.22 (2.43-4.07)	2.73 (2.16-3.16)	0.3230	0.6059	1.22 (1.05-1.63)	3.17 (2.15-3.32)	2.08 (1.3-2.65)	**0.0119**	0.221
IL-31	2.11 (1.42-2.65)	2.24 (1.98-3.47)	3.15 (2.04-4.24)	0.5040	0.7353	1.51 (0.955-1.79)	3.06 (2.34-4.17)	3.25 (2.01-3.79)	**0.0122**	0.221
IL-4	4.66 (2.47-5.98)	6.14 (5.49-11.5)	3.17 (1.72-4.05)	**0.0143**	0.3565	3.61 (1.87-4.47)	3.96 (2.61-4.52)	2.93 (2.05-4.42)	0.6560	0.6984
IL-5	5.31 (3.86-7.15)	8.03 (5.73-11.1)	5.28 (2.66-10.4)	0.2870	0.6059	5.29 (2.1-6.39)	5.87 (3.96-10.1)	3.35 (2.95-8.78)	0.3930	0.4838
IL-6	10.4 (6.4-12.4)	7.82 (4.65-22.8)	5.45 (4.92-13.1)	0.6880	0.8212	21.6 (13.1-24.2)	19.3 (12.4-28.4)	6.45 (3.33-29.6)	0.3880	0.4838
IL-7	1.17 (1-1.28)	1.12 (1.02-1.59)	1.46 (1.19-1.73)	0.4000	0.6565	1 (0.686-1.13)	1.56 (1.29-2.1)	1.5 (1.18-1.95)	**0.0228**	0.221
IL-8	7.7 (3.71-14.7)	2.72 (1.09-7.92)	17.9 (1.94-34.2)	0.1970	0.5543	3.66 (3.03-5.29)	12.5 (3.67-17.7)	7.64 (5.14-13.2)	0.1100	0.2549
IL-9	3.87 (1.74-5.37)	2.96 (1.35-3.61)	6.08 (2.28-10.5)	0.2770	0.6059	2.64 (2.27-3.86)	4.97 (3.07-6.39)	3.69 (2.51-4.81)	0.1890	0.3271
IP-10	1.65 (1.25-1.81)	2.7 (1.86-3.73)	1.62 (1.44-2.72)	0.2680	0.6059	1.64 (1.12-2.3)	2.29 (1.95-2.44)	2.01 (1.56-2.37)	0.2360	0.3873
MCP-1	5.44 (2.65-10.9)	2.61 (1.21-10.6)	3.44 (2.4-20.1)	0.5430	0.7639	4.25 (2.73-14)	17.6 (2.49-40.1)	3.79 (1.4-8.89)	0.3430	0.4795
MIP-1α	39.3 (19.5-61.7)	14.1 (7.43-28.2)	51.5 (33.4-66.9)	0.1290	0.4977	27.3 (12.1-36.2)	46.6 (34.6-73.8)	18.9 (5.11-37.2)	0.0568	0.2232
MIP-1β	19.6 (5.9-41.4)	4.54 (1.77-30.3)	25 (12.2-41.5)	0.3410	0.6105	28.1 (15.6-40.6)	33.8 (13.5-57)	12.4 (3.82-27.9)	0.1820	0.3271
RANTES	0.847 (0.663-1.02)	0.853 (0.689-1.12)	0.764 (0.497-1.13)	0.7800	0.9037	0.729 (0.641-0.973)	0.887 (0.661-1.1)	1.02 (0.946-1.18)	0.2870	0.4289
sCD40L	2.23 (2.04-2.67)	2.13 (1.61-2.54)	2.27 (2.16-2.37)	0.8090	0.9105	1.74 (1.02-1.98)	1.97 (1.79-2.82)	1.33 (1.14-1.97)	0.0850	0.2232
SCF	2.6 (2.28-3.04)	2.25 (1.91-3.12)	2.07 (1.25-3.22)	0.6100	0.8009	1.74 (1.11-2.23)	2.42 (2.26-2.57)	1.88 (1.41-2.67)	**0.0492**	0.221
SDF-1α	1.38 (1.08-1.56)	1.38 (0.893-1.91)	1.51 (1.24-2.06)	0.5880	0.7987	1.38 (1.18-1.58)	1.49 (1.24-1.77)	1.71 (1.31-1.93)	0.6030	0.6786

sICAM-1	0.867 (0.697-0.973)	1.1 (0.939-1.17)	1.1 (1-1.2)	**0.0181**	0.3565	0.899 (0.604-1.06)	0.968 (0.924-1.08)	1.04 (0.901-1.14)	0.3530	0.4795
TNF-α	104 (90-135)	155 (83.7-187)	47 (38.1-105)	0.1090	0.4977	89 (64.3-103)	104 (72.3-124)	56.3 (30.6-71.9)	0.0925	0.2277
TNF-β	1.72 (1.21-2.09)	2.49 (2.05-3.35)	2.67 (2.22-2.94)	0.0821	0.4977	1.18 (0.975-2.19)	2.29 (2.09-2.8)	2.15 (1.78-2.64)	0.0638	0.2232

Supplemental Table 27. Significance (p-values) of soluble mediator fold change differences following PMA/ionomycin stimulation across disease groups.

	White			Black					
**Soluble Mediator**	**Ctrl vs SLE INACT**	**Ctrl vs SLE ACT**	**SLE INACT vs SLE ACT**	**Ctrl vs SLE INACT**	**Ctrl vs SLE ACT**	**SLE INACT vs SLE ACT**	**White Ctrl vs Black Ctrl**	**White SLE INACT vs Black SLE INACT**	**White SLE ACT vs Black SLE ACT**
BLyS	1.0000	0.6570	0.8590	**0.0209**	0.1370	0.5730	0.2140	0.3640	0.5090
CXCL13	0.5970	1.0000	0.8590	0.9290	1.0000	0.8330	0.3280	0.0821	0.8040
Eotaxin	0.4060	0.4240	0.9290	**0.0263**	0.0693	0.2910	0.1830	**0.0025**	**0.0132**
GM-CSF	0.3260	0.2860	0.4770	0.3280	0.6800	0.7780	0.2480	0.4960	0.9340
GRO-α	1.0000	0.1100	0.4240	0.1830	0.8040	0.3240	0.5940	0.5450	0.1370
IFN-α	0.4060	0.0621	0.1100	0.5340	0.2150	0.2600	0.6570	0.2570	0.0829
IFN-γ	0.5450	0.0506	0.1310	0.8590	0.1170	**0.0242**	0.4240	0.4060	0.4570
IL-1α	0.3640	0.2860	0.1830	0.3740	0.5090	0.7780	0.5940	0.1990	0.4090
IL-1β	0.2900	0.2480	0.0621	0.4770	0.9340	0.1810	0.2480	**0.0256**	0.6200
IL-1RA	0.1740	0.7900	0.1550	0.2860	0.1170	**0.0112**	0.7220	0.0588	0.1370
IL-10	0.0821	**0.0129**	0.3740	0.2860	0.1370	**0.0075**	0.1310	0.2900	0.6200
IL-12p70	0.7050	0.6570	0.7900	0.4240	0.7410	**0.0317**	0.6570	0.2260	0.4570
IL-13	0.5450	0.1830	0.0914	0.1100	0.5630	0.3600	0.0756	0.4960	0.6800
IL-15	0.3840	0.5050	0.9290	0.0619	0.1860	0.9440	0.1190	0.4500	0.4570
IL-17A	0.4060	0.4770	0.2480	0.1310	0.6200	**0.0290**	0.3280	0.1990	0.5090
IL-18	0.4960	**0.0164**	0.0914	0.5940	**0.0475**	**0.0290**	0.6570	0.8210	0.6200
IL-2	0.2260	0.0756	0.5940	0.2860	0.5630	0.0671	0.0914	0.5970	0.5630
IL-21	1.0000	0.9290	1.0000	0.2140	1.0000	0.1210	0.5340	0.5450	0.3220
IL-22	0.1510	0.2480	0.0756	0.2480	0.7410	0.0573	1.0000	0.9400	0.3220
IL-23	0.1990	0.1830	0.8940	**0.0410**	**0.0258**	0.8880	0.1830	0.6500	0.3640
IL-27	0.1310	0.5940	0.4240	**0.0044**	0.1370	0.0782	0.1200	0.5450	0.3220
IL-31	0.4500	0.2480	0.7220	**0.0045**	**0.0208**	0.6730	0.1830	0.4960	0.6200

IL-4	0.0588	0.1550	**0.0077**	0.5340	0.9340	0.3600	0.1830	**0.0082**	0.8040
IL-5	0.0889	1.0000	0.3740	0.2860	0.6200	0.2310	0.5340	0.3840	0.8690
IL-6	0.7620	0.3280	0.7220	0.9290	0.6200	0.1050	0.2860	0.1120	0.7410
IL-7	0.7620	0.1300	0.4770	**0.0128**	**0.0202**	0.7780	0.1950	0.1400	0.7100
IL-8	0.1990	0.4240	0.1100	0.0914	**0.0475**	0.7780	0.2140	0.0588	0.3640
IL-9	0.6500	0.2860	0.1100	0.0619	0.2830	0.4810	0.2860	0.1300	0.3220
IP-10	0.0963	0.6570	0.3740	0.1550	0.3220	0.2310	0.8240	0.6500	0.5630
MCP-1	0.3260	0.9290	0.3740	0.4240	0.4090	0.1810	1.0000	0.0696	0.5090
MIP-1α	0.1510	0.4770	0.0621	0.0506	0.7410	**0.0346**	0.1310	**0.0343**	**0.0208**
MIP-1β	0.2570	0.5340	0.2140	0.4770	0.2150	0.0910	0.7900	**0.0284**	0.2480
RANTES	0.6500	0.8590	0.4770	0.5340	0.1170	0.3600	0.8590	0.9400	0.3640
sCD40L	0.6500	0.8590	0.5340	0.1100	0.8040	**0.0346**	**0.0410**	0.9400	**0.0050**
SCF	0.5200	0.3740	0.5940	**0.0164**	0.4090	0.1050	**0.0455**	0.3640	0.8040
SDF-1α	0.8210	0.3740	0.3740	0.5340	0.3640	0.5730	0.9290	0.4960	0.8690
sICAM-1	**0.0156**	**0.0164**	0.8590	0.4240	0.1600	0.4810	0.9290	0.1990	0.4570
TNF-α	0.4500	0.0756	0.0756	0.2860	0.2150	**0.0411**	0.1550	0.3260	0.8690
TNF-β	**0.0450**	0.0753	0.8590	**0.0263**	0.0693	0.6220	0.3740	0.8800	0.3640

Supplemental Table 28. Fold change (stimulated over unstimulated fluorescence intensity) of cytokine levels following PHA stimulation.

	Median Fold Change (IQR)					Median Fold Change (IQR)				
**Soluble Mediator**	**White Controls**	**White SLE INACT**	**White SLE ACT**	**KW p-value**	**FDR q-value**	**Black Controls**	**Black SLE INACT**	**Black SLE ACT**	**KW p-value**	**FDR q-value**
BLyS	1.03 (0.982-1.17)	1.06 (0.979-1.15)	1.13 (1.09-1.18)	0.4430	0.6477	1.01 (0.929-1.21)	1.06 (1.01-1.28)	1.07 (0.919-1.14)	0.7060	0.7867
CXCL13	1.49 (1.31-1.67)	1.31 (1.04-1.46)	1.44 (1.15-1.75)	0.3950	0.6477	1.66 (1.25-1.93)	1.67 (1.17-1.98)	1.27 (1.09-1.61)	0.6130	0.752
Eotaxin	0.958 (0.882-1.13)	0.856 (0.82-1.12)	1.1 (0.965-1.26)	0.4790	0.6739	1.32 (1.05-1.67)	1.66 (1.47-2.1)	1.56 (1.25-1.66)	0.2490	0.5162
GM-CSF	1.82 (1.43-3.66)	2.14 (1.48-3.95)	1.83 (1.48-2.38)	0.8840	0.9411	1.79 (1.08-1.88)	1.97 (1.51-2.91)	1.88 (1.06-3.9)	0.4470	0.6411
GRO-α	13.1 (9.97-16.4)	6.35 (2.07-11.6)	3.87 (2.32-6.23)	0.0960	0.3825	12.5 (5.62-19.1)	11.8 (4.94-19.6)	4.62 (2.47-11.8)	0.2700	0.5318
IFN-α	1.19 (1.08-1.32)	0.936 (0.728-1.15)	1.2 (0.968-1.37)	0.0560	0.3825	1.08 (1.06-1.1)	1.06 (0.945-1.5)	1.23 (1.09-1.44)	0.3000	0.5443
IFN-γ	2.65 (2.25-3.85)	3.35 (1.98-4.6)	1.72 (1.59-2.77)	0.4440	0.6477	3.47 (2.43-4.23)	3.01 (2.57-3.86)	2.25 (1.03-2.77)	0.1180	0.5162
IL-1α	2.82 (2.28-2.98)	1.77 (1.3-2.71)	2.44 (2.23-2.76)	0.2220	0.5712	2.93 (2.29-3.58)	3.05 (2.27-3.42)	2.8 (1.66-3)	0.6300	0.752
IL-1β	6.44 (5.07-19)	5.56 (1.24-9.54)	2.24 (1.7-2.75)	0.0971	0.3825	4.86 (2.87-7.69)	3.73 (2.91-12.4)	2.62 (1.53-3.65)	0.2120	0.5162
IL-1RA	40.6 (25.1-61.6)	11.1 (7.16-23.2)	34 (22.3-64.3)	0.0919	0.3825	31 (21.4-41.5)	30.9 (22.9-66.8)	11.6 (8.03-17.1)	**0.0126**	0.4037
IL-10	40.9 (19.3-54.2)	6.56 (3.14-11.6)	6.15 (2.84-21)	**0.0078**	0.3072	22.1 (16.6-33.3)	33.4 (14.9-52.4)	7.16 (3.32-14.2)	**0.0205**	0.4037
IL-12p70	1.47 (1.16-1.71)	1.59 (1.24-1.81)	1.25 (1.08-1.62)	0.5830	0.7233	1.14 (0.929-1.3)	1.55 (1.38-2.04)	1.3 (0.971-1.49)	**0.0327**	0.4116
IL-13	7.38 (5.24-11.9)	7.96 (3.63-11.7)	3.15 (1.41-9.4)	0.2710	0.5852	4.7 (2.9-5.97)	8.95 (4.31-20.4)	4.98 (1.97-10.8)	0.3340	0.5695
IL-15	1.15 (0.93-1.37)	1.19 (0.949-1.5)	1.56 (1.21-1.91)	0.2980	0.5852	1.15 (1.05-1.39)	1.23 (0.906-1.84)	1.47 (0.846-1.86)	0.8880	0.8969
IL-17A	39.8 (26-58.5)	56.8 (12.4-202)	13 (3.06-34)	0.2300	0.5712	73.1 (44.9-106)	136 (52.3-278)	18.3 (9.18-94.9)	0.1080	0.5162

IL-18	2.41 (1.7-2.77)	2.13 (1.77-2.31)	1.89 (1.59-2.61)	0.5730	0.7233	2.11 (1.62-2.54)	2.35 (2.03-2.92)	1.48 (1.14-2.06)	0.0532	0.4116
IL-2	9.55 (4.74-17.7)	4.17 (2.16-6.04)	15.2 (4.56-22.1)	0.2850	0.5852	5.44 (4.25-9.31)	12.3 (9.63-35.1)	11.9 (2.35-25)	0.2420	0.5162
IL-21	1.76 (1.4-2.42)	1.62 (1.22-1.91)	2.09 (1.56-2.58)	0.2830	0.5852	1.84 (1.26-2.1)	2.25 (1.86-2.41)	1.82 (1.25-2.06)	0.2020	0.5162
IL-22	2.59 (1.68-3.55)	3.75 (1.83-13.3)	1.91 (1.07-2.88)	0.3270	0.5855	4.01 (3.27-11.9)	7.33 (5.37-13.8)	2.89 (1.86-15.5)	0.3470	0.5695
IL-23	1.62 (1.45-2.75)	2.04 (1.61-2.43)	1.5 (1.12-2.17)	0.6050	0.7233	2.03 (1.52-2.39)	2.05 (1.35-3.83)	2.6 (1.43-3.6)	0.8240	0.8541
IL-27	1.14 (0.908-1.2)	1.53 (0.981-1.64)	1.25 (0.978-1.58)	0.6060	0.7233	0.909 (0.684-0.918)	1.14 (0.882-1.65)	1.22 (0.734-1.33)	0.1900	0.5162
IL-31	1.15 (1.02-1.33)	1.11 (1.04-1.55)	1.22 (0.935-1.42)	0.9770	0.9868	1.16 (1.02-1.34)	1.13 (1.02-1.91)	1.25 (1.06-1.56)	0.6890	0.7867
IL-4	1.91 (1.41-2.1)	2.13 (1.64-2.46)	1.45 (1.04-1.96)	0.2070	0.5712	1.49 (0.963-2.19)	2 (1.47-2.17)	1.02 (0.959-2.06)	0.3760	0.5924
IL-5	1.67 (1.36-1.85)	1.88 (1.69-2.05)	1.92 (1.47-2.28)	0.5220	0.709	1.4 (1.09-1.83)	1.93 (1.78-2.18)	1.29 (1.06-2.24)	0.1070	0.5162
IL-6	18.2 (12.2-24.5)	10.1 (5.11-24.3)	3.88 (2.85-5.56)	**0.0317**	0.3825	27.9 (15.1-36.7)	23.3 (14.6-30)	13 (5.49-26.5)	0.3040	0.5443
IL-7	1.14 (1-1.21)	1.24 (0.98-1.56)	1.29 (1.11-1.54)	0.4420	0.6477	1.19 (1-1.36)	1.44 (1.28-1.57)	1.26 (1-1.87)	0.2370	0.5162
IL-8	16.1 (5.09-19.1)	1.69 (1.12-9.93)	15.3 (4.01-53.9)	0.0755	0.3825	7.06 (5.51-8.61)	20.6 (6.04-24.5)	9.49 (8.07-13.5)	0.1600	0.5162
IL-9	4.94 (2.35-6.84)	1.72 (1.31-4.35)	5.88 (2.82-11.7)	0.0826	0.3825	4.34 (2.83-5.01)	6.82 (3.17-7.31)	4.19 (3.75-5.51)	0.2270	0.5162
IP-10	3.25 (1.91-3.76)	2.78 (2.13-3.09)	2.38 (1.27-3.15)	0.6720	0.7785	2.99 (1.46-3.29)	3.31 (2.63-3.66)	2.51 (1.49-3.39)	0.5710	0.7497
MCP-1	6.4 (3.21-18.8)	1.68 (1.2-14)	4.01 (2.6-5.52)	0.3540	0.6063	5.34 (2.69-22.3)	5.58 (2.89-36.3)	4.85 (1.29-6.86)	0.4720	0.6411
MIP-1α	47.5 (23.2-86.4)	9.47 (5.35-27.4)	56.2 (36.8-74.9)	0.0784	0.3825	31 (14.3-42.8)	53.7 (35.8-73.7)	18.7 (6.57-41.9)	0.0627	0.4116
MIP-1β	38.8 (7.84-73.9)	3.56 (2.06-41.4)	50.7 (24.1-86.8)	0.0835	0.3825	37.4 (17.2-64.1)	49.3 (25.5-63.7)	17.7 (6.44-43.3)	0.1600	0.5162
RANTES	1.48 (1.08-1.52)	0.974 (0.785-1.42)	1.07 (0.974-1.35)	0.3120	0.5852	1.18 (0.916-1.47)	1.24 (0.955-2.02)	1.07 (1.02-1.77)	0.7190	0.7867
sCD40L	1.57 (1.32-1.64)	1.21 (1.16-1.35)	1.34 (1.21-1.55)	0.1240	0.444	1.28 (1.04-1.52)	1.32 (1.23-1.7)	1.06 (0.963-1.21)	**0.0422**	0.4116

SCF	1.15 (1-1.24)	1.17 (1-1.35)	1.2 (1.04-1.4)	0.8400	0.9191	1.04 (0.905-1.17)	1.21 (0.901-1.5)	0.992 (0.894-1.25)	0.4650	0.6411
SDF-1α	2.05 (1.73-2.74)	1.56 (1.2-1.98)	1.9 (1.81-2.1)	0.2320	0.5712	2.08 (1.82-2.71)	2.51 (1.77-2.71)	1.84 (1.39-2.65)	0.7670	0.8165
sICAM-1	1.12 (1.04-1.18)	1.13 (0.974-1.17)	1.08 (0.96-1.16)	0.7690	0.8655	1.13 (0.869-1.25)	1 (0.838-1.11)	1.07 (0.945-1.13)	0.6050	0.752
TNF-α	17 (13.2-20.7)	12.9 (5.54-16.1)	4.89 (2.93-17.4)	0.1630	0.535	10.1 (8.05-11.3)	12.9 (8.39-19.6)	8.17 (4.49-15.2)	0.4100	0.6212
TNF-β	1.18 (1.03-1.35)	1.26 (0.991-1.82)	1.21 (1.13-1.4)	0.9590	0.9868	0.996 (0.789-1.14)	1.13 (0.964-1.85)	1.29 (1.01-1.66)	0.2090	0.5162

Supplemental Table 29. Significance (p-values) of soluble mediator fold change differences following PHA stimulation across disease groups.

	White			Black					
Soluble Mediator	Ctrl vs SLE INACT	Ctrl vs SLE ACT	SLE INACT vs SLE ACT	Ctrl vs SLE INACT	Ctrl vs SLE ACT	SLE INACT vs SLE ACT	White Ctrl vs Black Ctrl	White SLE INACT vs Black SLE INACT	White SLE ACT vs Black SLE ACT
BLyS	0.8500	0.2480	0.2860	0.3280	0.8040	0.7250	0.5940	0.4960	0.2310
CXCL13	0.1730	0.5940	0.4770	0.9650	0.4570	0.3600	0.6570	0.3440	0.8040
Eotaxin	0.3640	0.4770	0.3280	0.1310	0.3640	0.2910	0.0914	**0.0012**	**0.0258**
GM-CSF	0.8210	0.7220	0.6570	0.1680	0.4090	0.9160	0.2480	0.5970	0.8690
GRO-α	0.1510	**0.0263**	0.7900	0.9290	0.1860	0.1590	0.7900	0.4500	0.5630
IFN-α	**0.0343**	0.8590	0.0506	1.0000	0.0986	0.3240	0.1830	0.1310	0.6800
IFN-γ	0.9400	0.1830	0.3740	0.7220	0.1370	**0.0486**	0.6570	0.8800	0.5630
IL-1α	0.1310	0.7900	0.1550	0.7220	0.3640	0.5260	0.6570	0.1990	1.0000
IL-1β	0.3640	**0.0129**	0.5340	0.8240	0.1600	0.1210	0.2860	0.5450	0.9010
IL-1RA	**0.0413**	0.7220	0.1100	0.5340	0.0575	**0.0039**	0.3280	**0.0156**	0.0693
IL-10	**0.0082**	**0.0077**	0.9290	0.5940	**0.0475**	**0.0092**	0.2860	**0.0233**	1.0000
IL-12p70	0.7050	0.5050	0.3070	**0.0112**	0.2830	0.0977	0.0504	0.7340	0.7410
IL-13	0.7620	0.1100	0.2480	0.1550	0.9340	0.2600	0.0914	0.7620	0.6800
IL-15	0.9400	0.2130	0.1420	0.7560	0.6200	0.7780	0.7900	0.7050	0.4330
IL-17A	0.4960	0.0914	0.2140	0.4240	0.2830	**0.0346**	0.1550	0.2260	0.3220
IL-18	0.4060	0.3280	0.9290	0.3740	0.1860	**0.0167**	0.3740	0.3260	0.2480
IL-2	0.1310	0.7900	0.2480	0.0621	0.6800	0.3600	0.4770	**0.0082**	0.8690
IL-21	0.3640	0.5340	0.1100	0.1310	1.0000	0.1210	0.5340	0.1120	0.1600
IL-22	0.4060	0.4240	0.1550	0.2860	0.5090	0.2050	0.1310	0.1990	0.1170
IL-23	0.6230	0.4770	0.3740	0.6890	0.5090	0.9440	0.7900	0.8500	0.2830
IL-27	0.2900	0.5930	0.8590	0.0828	0.2150	0.4600	0.0827	0.5970	0.3220
IL-31	0.9400	0.9650	0.7900	0.5630	0.3640	0.9720	0.7220	0.7620	0.5630
IL-4	0.4500	0.2480	0.0914	0.3280	0.8040	0.1810	0.4240	0.6500	0.7410
IL-5	0.2260	0.5050	1.0000	**0.0410**	0.8690	0.1130	0.3740	0.5200	0.6790

IL-6	0.2570	**0.0077**	0.1550	0.7220	0.2150	0.1810	0.5340	0.1510	0.0522
IL-7	0.7900	0.1780	0.4230	0.0617	0.4050	0.5490	0.8560	0.2560	0.8360
IL-8	0.0963	0.5940	**0.0330**	0.0914	0.1600	0.3600	0.2860	**0.0156**	0.4570
IL-9	0.1510	0.3740	**0.0330**	0.1310	0.5630	0.1810	0.4770	**0.0284**	0.2830
IP-10	0.6500	0.3740	0.6570	0.4240	0.9340	0.3240	0.6570	0.4060	0.8040
MCP-1	0.1740	0.4240	0.4770	0.7220	0.2830	0.3240	0.8590	0.1740	0.8690
MIP-1α	**0.0494**	0.8590	0.0621	0.1550	0.3640	**0.0242**	0.2140	**0.0284**	**0.0318**
MIP-1β	**0.0494**	0.5940	0.0756	0.7220	0.1600	0.0783	1.0000	**0.0284**	0.1600
RANTES	0.1510	0.3740	0.4770	0.4770	0.5090	0.7780	0.3280	0.2570	0.3220
sCD40L	**0.0343**	0.4770	0.3280	0.6570	0.2480	**0.0060**	0.2860	0.2260	**0.0318**
SCF	0.7620	0.6570	0.5940	0.2480	0.6200	0.3980	0.1970	0.8210	0.4090
SDF-1α	0.1310	0.6570	0.1830	0.9290	0.6200	0.4810	0.7220	0.1120	1.0000
sICAM-1	0.8210	0.4240	0.7220	0.3280	0.5630	0.6220	1.0000	0.1510	0.7410
TNF-α	0.1990	0.0621	0.6250	0.2140	0.7410	0.2910	**0.0129**	0.8210	1.0000
TNF-β	0.8800	0.7220	0.8590	0.1830	0.0829	0.8880	0.1100	0.8500	0.9340

SUPPLEMENTAL REFERENCES

1. Young MD, and Behjati S. SoupX removes ambient RNA contamination from droplet based single-cell RNA sequencing data. *bioRxiv.* 2020:303727.
2. Hao Y, Hao S, Andersen-Nissen E, et al. Integrated analysis of multimodal single-cell data. *Cell.* 2021;184(13):3573-87.e29.
3. McGinnis CS, Patterson DM, Winkler J, et al. MULTI-seq: sample multiplexing for single-cell RNA sequencing using lipid-tagged indices. *Nature Methods.* 2019;16(7):619-26.
4. Mulè MP, Martins AJ, and Tsang JS. Normalizing and denoising protein expression data from droplet-based single cell profiling. *Nature Communications.* 2022;13(1):2099.
5. Korsunsky I, Millard N, Fan J, et al. Fast, sensitive and accurate integration of single-cell data with Harmony. *Nature Methods.* 2019;16(12):1289-96.
6. Chaussabel D, and Baldwin N. Democratizing systems immunology with modular transcriptional repertoire analyses. *Nat Rev Immunol.* 2014;14(4):271-80.
7. Banchereau R, Hong S, Cantarel B, et al. Personalized Immunomonitoring Uncovers Molecular Networks that Stratify Lupus Patients. *Cell.* 2016;165(3):551-65.
8. Finak G, McDavid A, Yajima M, et al. MAST: a flexible statistical framework for assessing transcriptional changes and characterizing heterogeneity in single-cell RNA sequencing data. *Genome Biology.* 2015;16(1):278.
9. Price AL, Butler J, Patterson N, et al. Discerning the ancestry of European Americans in genetic association studies. *PLoS Genet.* 2008;4(1):e236.
10. Hochberg MC. Updating the American College of Rheumatology revised criteria for the classification of systemic lupus erythematosus. *Arthritis Rheum.* 1997;40(9):1725.
11. Petri M, Orbai AM, Alarcon GS, et al. Derivation and validation of the Systemic Lupus International Collaborating Clinics classification criteria for systemic lupus erythematosus. *Arthritis Rheum.* 2012;64(8):2677-86.
12. Slight-Webb S, Smith M, Bylinska A, et al. Autoantibody-positive healthy individuals with lower lupus risk display a unique immune endotype. *J Allergy Clin Immunol.* 2020;146(6):1419-33.
13. Nehar-Belaid D, Hong S, Marches R, et al. Mapping systemic lupus erythematosus heterogeneity at the single-cell level. *Nat Immunol.* 2020;21(9):1094-106.
14. Wolf FA, Angerer P, and Theis FJ. SCANPY: large-scale single-cell gene expression data analysis. *Genome Biol.* 2018;19(1):15.
15. van Duin D, Mohanty S, Thomas V, et al. Age-associated defect in human TLR-1/2 function. *J Immunol.* 2007;178(2):970-5.
